# Mono- vs. Bis-Guanyl Hydrazone-Decorated Tricyclic Scaffolds: Effects on G-Quadruplex Binding and Selectivity

**DOI:** 10.3390/ijms27125282

**Published:** 2026-06-10

**Authors:** Chiara Platella, Alice Maiocchi, Giovanni Cipolla, Rosa Gaglione, Angela Arciello, Pierfausto Seneci, Domenica Musumeci, Alessandra Silvani, Clelia Giannini, Daniela Montesarchio

**Affiliations:** 1 Department of Chemical Sciences, University of Naples Federico II, Via Cintia 21, 80126 Naples, Italy; chiara.platella@unina.it (C.P.); rosa.gaglione@unina.it (R.G.); angela.arciello@unina.it (A.A.); domenica.musumeci@unina.it (D.M.); 2 Department of Chemistry, University of Milan, Via Golgi 19, 20133 Milan, Italy; alice.maiocchi@unimi.it (A.M.); giovanni.cipolla@studenti.unimi.it (G.C.); pierfausto.seneci@unimi.it (P.S.); alessandra.silvani@unimi.it (A.S.); 3 Institute of Biostructure and Bioimaging (IBB), National Research Council (CNR), 80145 Naples, Italy

**Keywords:** guanyl hydrazones, tricyclic aromatic compounds, G-quadruplex ligands, cancer

## Abstract

Mono- and bis-guanyl hydrazone-functionalized tricyclic compounds were here designed and investigated as putative G-quadruplex ligands in the context of anticancer drug development. The G-quadruplex on Controlled Pore Glass (G4-CPG) assay, a fast and easy screening method based on affinity chromatography for identifying potential G-quadruplex binders, together with biophysical techniques such as circular dichroism and fluorescence spectroscopy, demonstrated a higher selectivity of mono- with respect to disubstituted derivatives in recognizing G-quadruplexes from telomeric and oncogenic DNA regions vs. duplexes. Among the mono-substituted compounds, higher G-quadruplex selectivity was found for those containing the pyrido [3,4-*b*]indole and dibenzofuran scaffolds compared to the 9H-fluorene, 9H-carbazole, and dibenzothiophene ones. Molecular docking studies suggested that the investigated ligands bound the hybrid telomeric G-quadruplex model by adopting a coplanar arrangement of the core and guanyl hydrazone moieties, both stacked on the 5′-G-quartet, while in the interaction with the parallel oncogenic G-quadruplex model the guanyl hydrazone moieties pointed towards the grooves/loops. Finally, biological assays highlighted the higher potential of mono-guanyl hydrazone-derivatized tricyclic compounds as selective anticancer agents, showing higher anticancer activity and selectivity of action than the bis-guanyl hydrazone derivatives.

## 1. Introduction

In the quest to identify agents with enhanced biological activity against cancer cells, over the past decade a growing interest has been focused on the discovery and development of small molecules capable of interacting with nucleic acids. Among these, ligands able to recognize and bind non-canonical secondary structures, such as G-quadruplexes [1], have emerged as promising candidates due to their potential biological relevance.

Among non-canonical nucleic acid architectures, G-quadruplexes—i.e., tetrahelical structures formed by stacked G-tetrads interconnected through Hoogsteen hydrogen bonds and stabilized by monovalent cations—have attracted considerable attention due to the strict correlation between G-quadruplex folding/unfolding and telomere maintenance/oncogene expression regulation at both transcriptional and translational levels [2].

Despite extensive efforts in the development of G-quadruplex-selective ligands, as reflected by the vast body of literature in this field [3,4,5], identifying a small molecule characterized by both high affinity and selectivity toward specific G-quadruplex structures remains a major challenge, mainly due to non-specific binding to either the most similar molecular entities to G-quadruplexes which can be found in cells, i.e., the abundant duplex DNA or RNA, or off-target proteins. Typically, G-quadruplex ligands feature planar polyaromatic cores that enable π–π stacking interactions with terminal G-tetrads, complemented by positively charged or polar substituents that interact with loops and grooves [4,6]. Although many structural classes—such as fused aromatic polycycles, macrocycles, and modular ligands—have demonstrated promising G-quadruplex-binding capabilities, most candidates still suffer from limited selectivity, poor pharmacokinetic properties, or excessive molecular complexity.

In this context, increasing efforts have been devoted to understanding how two key structural features, i.e., symmetry and substitution pattern with polar groups of the central molecular framework, affect G-quadruplex binding modes, affinity, and selectivity.

Molecular symmetry may play a critical role in defining a specific binding mode as well as modulating the relative affinity and selectivity towards G-quadruplexes [7]. In particular, bis-substituted ligands can enhance affinity through multivalent interactions, but their higher molecular complexity may also introduce steric constraints that reduce the conformational flexibility required for selective recognition of G-quadruplex structures. Conversely, mono-substituted derivatives may in some cases provide improved selectivity by minimizing steric hindrance and enabling more precise interactions with specific G-quadruplex topologies.

As far as the polar substitution pattern is concerned, the presence of one or two positively charged substituents at physiological pH appears to enable multi-site interactions with the G-quadruplex loops, grooves, and/or flanking residues [8]. Fine-tuning the polarity, rigidity, and size of these substituents may therefore represent an effective strategy to modulate interactions with distinct G-quadruplex topologies.

Overall, analysis of previous studies on the G-quadruplex thermal stabilization efficiency and recognition selectivity by the most relevant G-quadruplex-targeting organic ligands has led to the general conclusion that aromatic modules containing electron-withdrawing atoms or groups—such as nitrogen, halogens, or cationic functions—enhance G-quadruplex binding through π–π stacking interactions, while positively charged side chains can have significant effects on improving G-quadruplex binding and stabilization [9,10,11,12,13]. The optimal length of these side chains depends on the ligand scaffold and can modulate the strength of binding to G-quadruplexes by engaging interactions with their loops and grooves.

Building on these considerations and our previous findings in the field [7,14], in the present study we selected a series of aromatic tricyclic scaffolds to investigate the influence of molecular symmetry and substitution degree on the recognition of specific G-quadruplex structures. In choosing the type of polar substitution, we were inspired by the emerging role of aryl guanyl hydrazones in medicinal chemistry due to their target-tunable properties [15]. The presence of multiple tautomeric forms for guanyl hydrazones allows for extensive charge delocalization across several nitrogen atoms, resulting in a flexible charge distribution. This electronic adaptability may contribute to more effective accommodation of the molecules within nucleic acid binding sites, thereby supporting their potential as effective ligands for these biological targets [7,16,17,18].

Herein, we describe the G-quadruplex-binding properties of mono- and bis-guanyl hydrazone-functionalized tricyclic scaffolds as determined by using a combined approach including an affinity chromatography-based screening, biophysical analyses, and computational studies, accompanied by biological assays aimed at preliminarily investigating the anticancer activity and selectivity of these compounds.

## 2. Results and Discussion

### 2.1. Synthesis of Mono- and Bis-Guanyl Hydrazone-Functionalized Tricyclic Compounds

**Figure 1:**
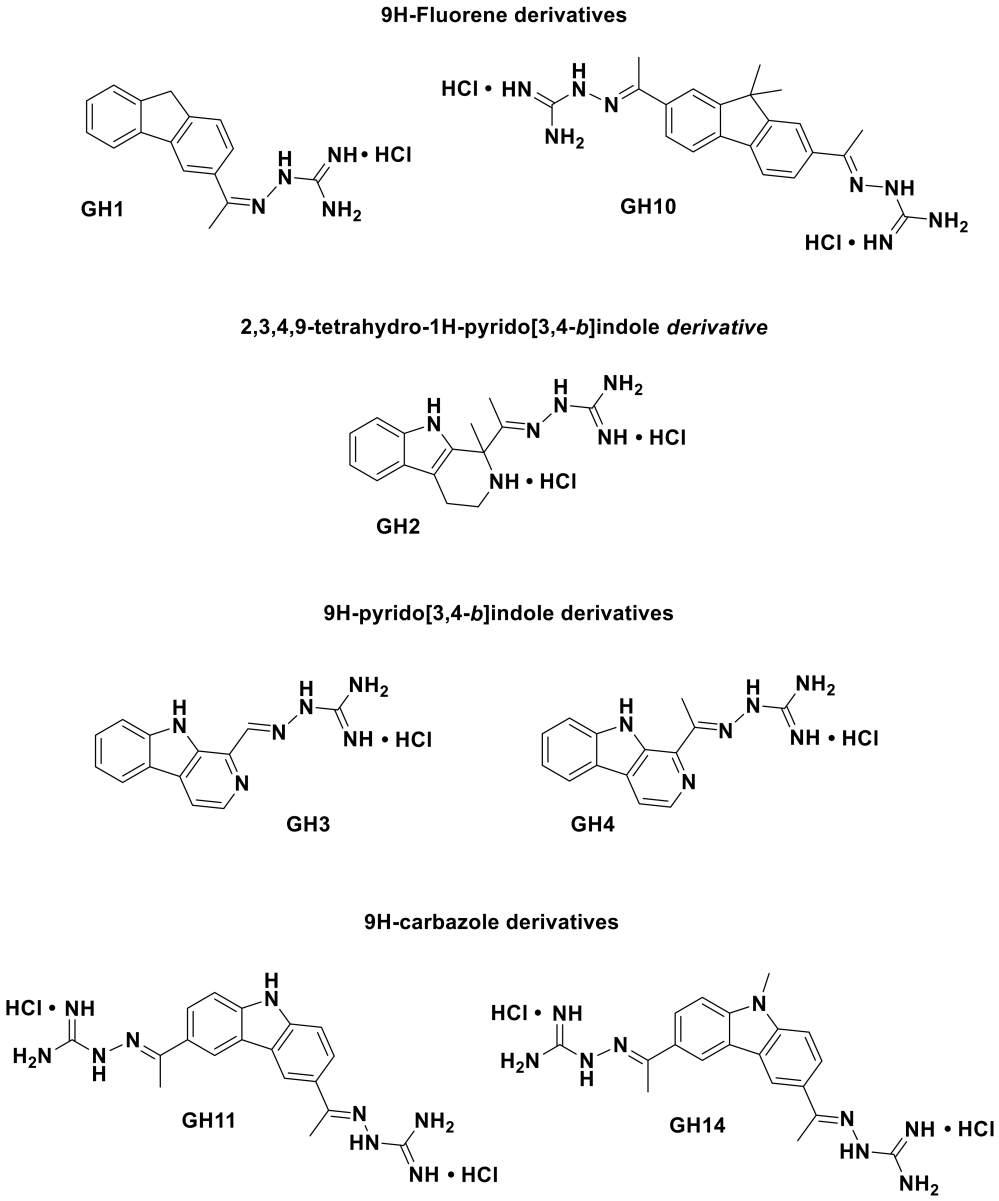

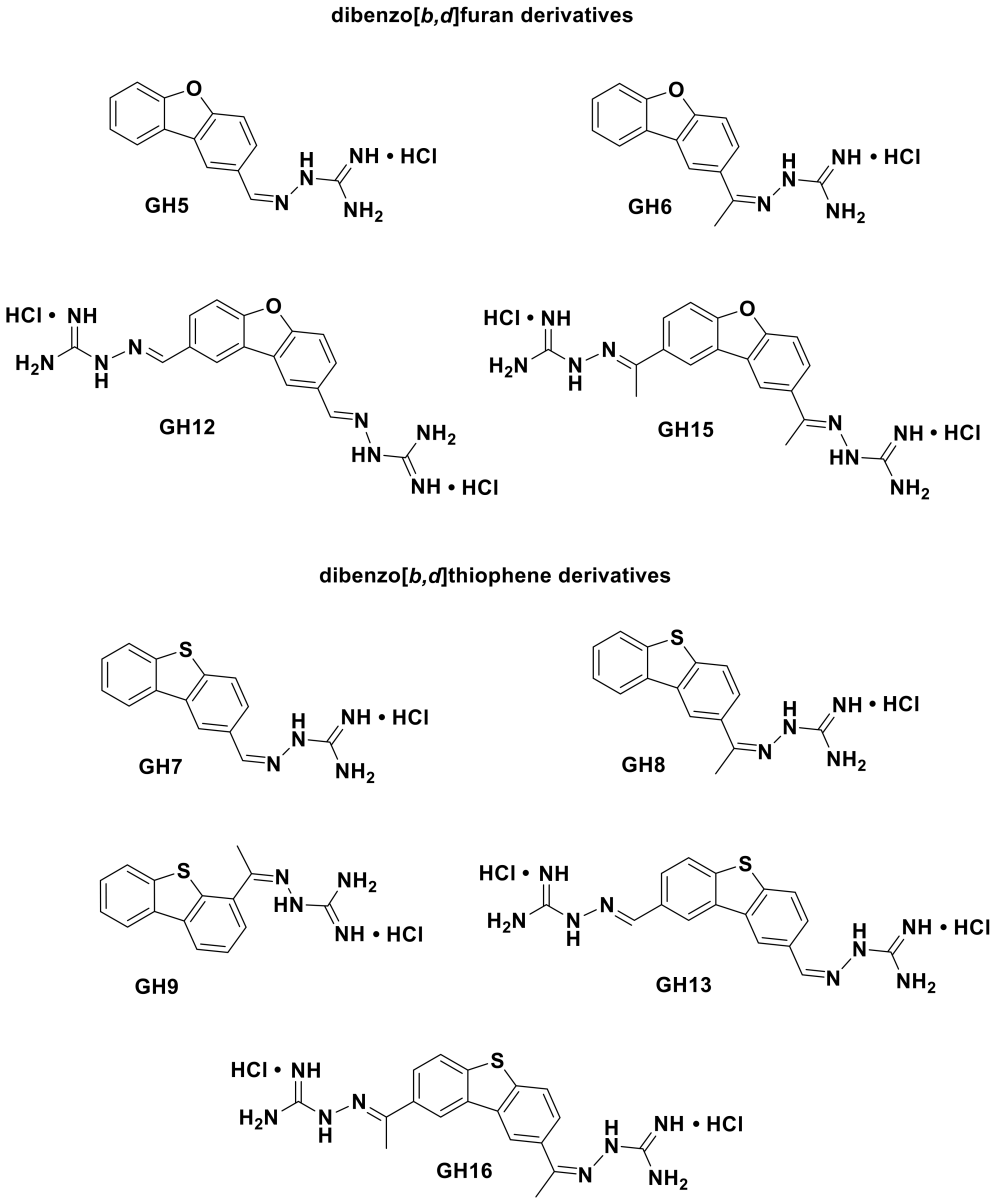
Synthesized compounds, grouped according to the different tricyclic cores.

The investigated novel compounds, whose syntheses are reported below, can be divided into derivatives bearing one (**GH1**–**GH9**) or two (**GH10**–**GH16**) guanyl hydrazone groups and further differentiated into six families, based on their tricyclic cores (Figure 1): 9H-fluorene (**GH1**, **GH10**), 2,3,4,9-tetrahydro-1H-pyrido [3,4-*b*]indole (**GH2**), 9H-pyrido [3,4-*b*]indole (**GH3**, **GH4**), 9H-carbazole (**GH11**, **GH14**), dibenzo[*b*,*d*]furan (**GH5**, **GH6**, **GH12**, **GH15**), and dibenzo[*b*,*d*]thiophene (**GH7**, **GH8**, **GH9**, **GH13**, **GH16**). The following guanyl hydrazones carried an additional methyl group at the hydrazone carbon atom: **GH1**, **GH2**, **GH4**, **GH6**, **GH8**, **GH9**, **GH10**, **GH11**, **GH14**, **GH15**, **GH16**.

#### 2.1.1. Synthesis of Mono-Guanyl Hydrazone-Functionalized Derivatives **GH1**–**GH9**

**Scheme 1:**
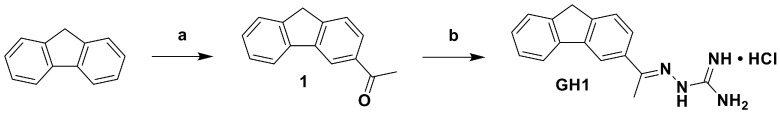
Synthesis of **GH1**. Reaction conditions: (a) AcCl, AlCl_3_, dry DCM, N_2_, r.t., 4 h, 71%; (b) AG·HCl, EtOH, cat. 1 M HCl, 80 °C, 7 h, 43%.

For the synthesis of **GH1** (Scheme 1), a Friedel–Crafts acylation on 9H-fluorene was first performed. The low reactivity of the starting scaffold led to the selective formation of the mono-acylated intermediate **1** in good yield. Condensation with aminoguanidine hydrochloride (AG·HCl) was then carried out using a catalytic amount of HCl and refluxing the reaction mixture in EtOH to promote sufficient acetyl group reactivity and reagent solubility. The fluorene derivative **GH1** was thus obtained in moderate overall yield, after purification by reverse-phase column chromatography.

**Scheme 2:**
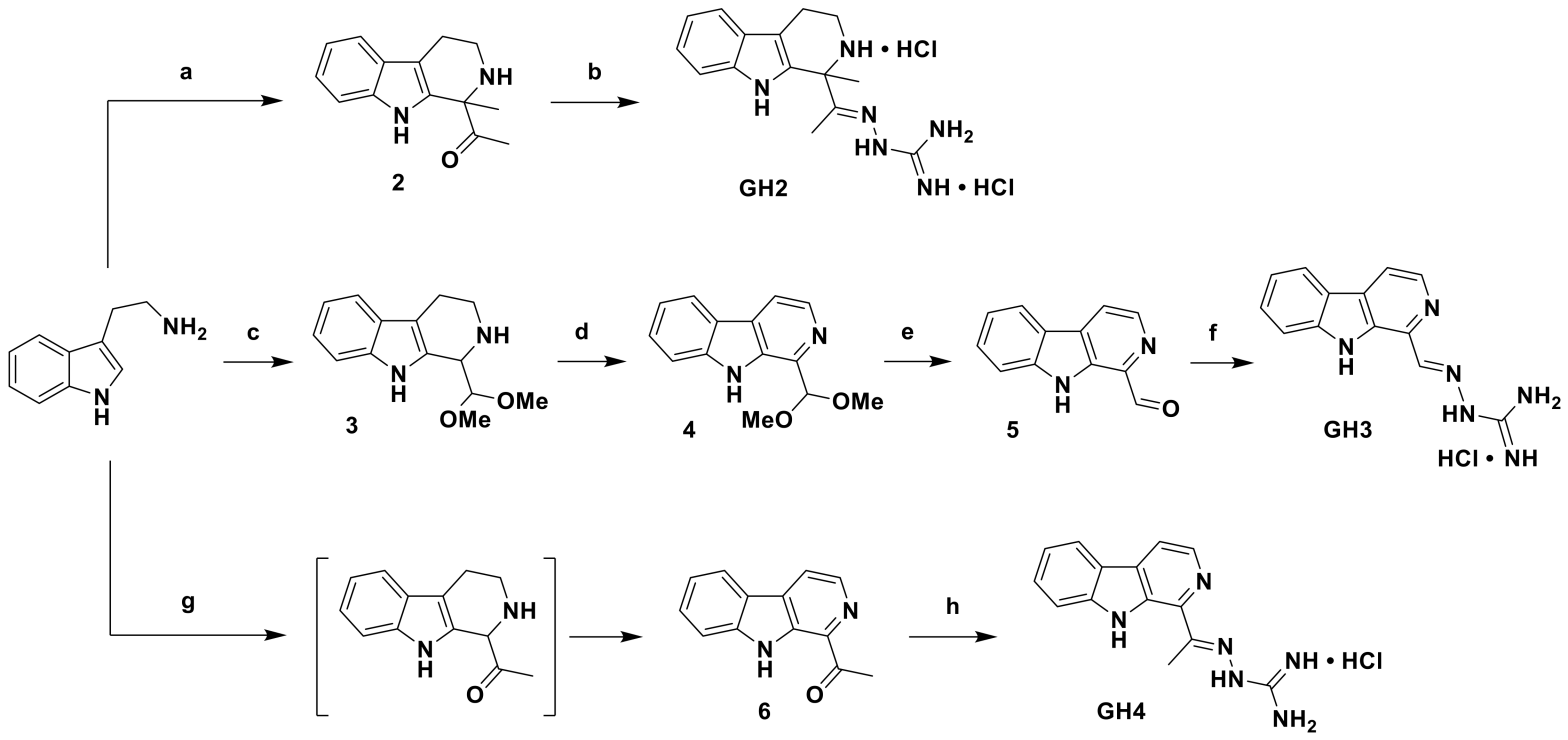
Synthesis of **GH2**–**GH4**. Reaction conditions: (a) butane-2,3-dione, TFA, MeOH, 60 °C, 7 h, 63%; (b) AG·HCl, cat. 1 M HCl, EtOH, 80 °C, 14 h, 46%; (c) 2,2-dimethoxyacetaldehyde, TFA, DCM, r.t., overnight, 48%; (d) KMnO_4_, THF, r.t., 25 h, 47%; (e) AcOH, 1,4-dioxane, H_2_O, N_2_, 100 °C, 1 h, 76%; (f) AG·HCl, EtOH, cat. 1 M HCl, 80 °C, 7 h, 40%; (g) (1) pyruvaldehyde (40% in H_2_O), H_2_SO_4_, H_2_O, r.t., overnight, (2) KMnO_4_, THF, r.t., overnight, 12% over 2 steps; (h) AG·HCl, cat. 1 M HCl, EtOH, 80 °C, 7 h, 85%.

**GH2** was obtained by coupling tryptamine with butane-2,3-dione (Scheme 2, step a), giving intermediate **2** in good yield, followed by condensation with AG·HCl (step b). The reaction was slow, likely due to steric hindrance around the carbonyl group and poor solubility of **2** in its protonated form, but after refluxing for 14 h the desired tetrahydro-β-carboline derivative **GH2** could be obtained, even if in moderate yield.

Reaction of tryptamine with dimethoxyacetaldehyde (step c) gave intermediate **3**, which was aromatized by treatment with KMnO_4_ (step d) to afford acetal **4** in moderate yield. Mild acid hydrolysis (step e) provided the corresponding aldehyde **5** in good yield. Condensation with AG·HCl (step f) produced the β-carboline derivative **GH3** in good yield and purity after trituration in Et_2_O and reverse-phase column chromatography.

The methyl analogue **GH4** was synthesized by cyclization of tryptamine with pyruvaldehyde followed by aromatization (step g), giving ketone **6** in low yield. Condensation with AG·HCl (step h) provided **GH4** in good yield and excellent purity after Et_2_O trituration, without requiring chromatographic column purifications.

**Scheme 3:**
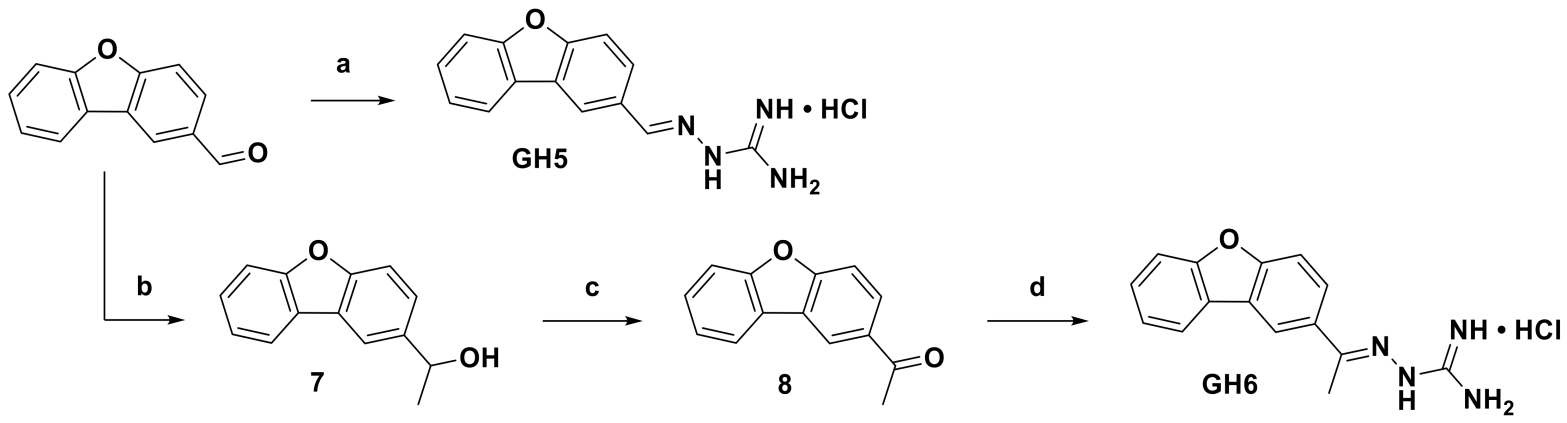
Synthesis of **GH5** and **GH6**. Reaction conditions: (a) AG·HCl, EtOH, cat. 1 M HCl, 80 °C, 8 h, 54%; (b) MeMgBr, dry THF, N_2_, 0 °C to r.t., 2.5 h, 86%; (c) DMP, dry DCM, 0 °C to r.t., overnight, 77%; (d) AG·HCl, EtOH, cat. 1 M HCl, 80 °C, 7 h, 70%.

The synthesis of the dibenzofuran derivative **GH5** is outlined in Scheme 3. Commercially available dibenzo[*b*,*d*]furan-2-carbaldehyde was condensed with AG·HCl (step a) to afford **GH5** in moderate yield. For the synthesis of the methyl analogue **GH6**, addition of methylmagnesium bromide to the same aldehyde furnished racemic secondary alcohol **7** in very good yield (step b). Oxidation of **7** with Dess–Martin periodinane (DMP) afforded the acetyl derivative **8** in good yield (step c). Subsequent condensation of key intermediate **8** with AG·HCl (step d) provided **GH6** in good yield and purity.

**Scheme 4:**
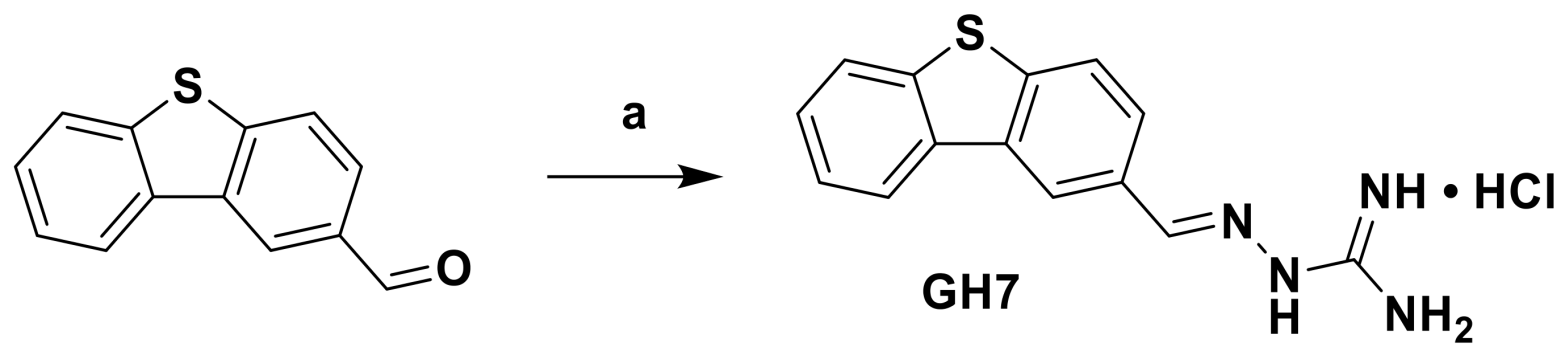
Synthesis of **GH7**. Reaction conditions: (a) AG·HCl, cat. 1 M HCl, EtOH, 80 °C, 7 h, 86%.

The dibenzothiophene derivative **GH7** was synthesized starting from commercially available dibenzo[*b*,*d*]thiophene-2-carbaldehyde via condensation with AG·HCl (Scheme 4). After solvent removal, the residue was washed with diisopropyl ether to remove unreacted starting material, affording **GH7** in good yield and purity.

**Scheme 5:**
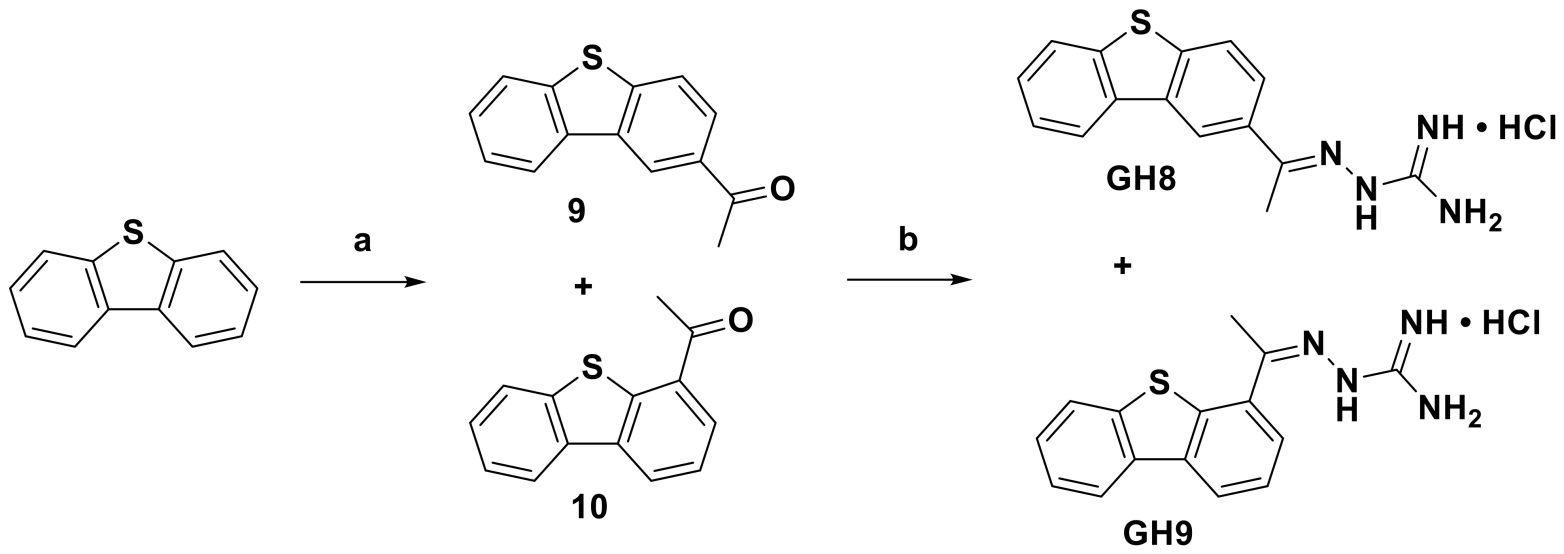
Synthesis of **GH8** and **GH9**. Reaction conditions: (**a**) AlCl_3_, AcCl, dry DCM, N_2_, r.t., overnight, 27% (**9**), 14% (**10**); (**b**) AG·HCl, EtOH, cat. 1 M HCl, 80 °C, 8 h, 73% (**GH8**), 78% (**GH9**).

The synthesis of the methyl analogue **GH8** and its isomer **GH9** is depicted in Scheme 5. Friedel–Crafts acylation of dibenzothiophene with acetyl chloride and AlCl_3_ (step a) afforded the acetylated intermediate **9** in low, unoptimized yield, due to the concomitant formation of its isomer **10** and recovery of unreacted starting material. Subsequent condensation of both acetyl intermediates **9** and **10** with AG·HCl under standard conditions (step b) provided derivatives **GH8** and **GH9**, respectively, in good yields.

#### 2.1.2. Synthesis of Bis-Guanyl Hydrazone-Functionalized Derivatives **GH10**–**GH16**

**Scheme 6:**
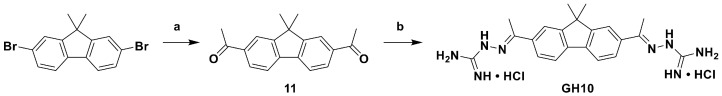
Synthesis of **GH10**. Reaction conditions: (a) (1) *n*-BuLi, dry THF, N_2_, −78 °C to −30 °C, 1 h, (2) *N*-methoxy-*N*-methylacetamide, −78 °C to r.t., 18 h, 58%; (b) AG·HCl, EtOH, cat. 1 M HCl, 80 °C, 4 h, 96%.

The synthesis of dimethylfluorene derivative **GH10** is outlined in Scheme 6. Lithium–halogen exchange of commercially available 2,7-dibromo-9,9-dimethylfluorene upon treatment with *n*-BuLi, followed by electrophilic quenching with *N*-methoxy-*N*-methylacetamide (step a), afforded the diacetyl intermediate **11**. Subsequent condensation with AG·HCl under standard conditions (step b) provided **GH10** in excellent yield. The product was isolated with adequate purity by simple washings with organic solvents, without requiring chromatographic column purifications.

**Scheme 7:**
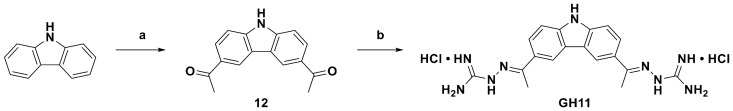
Synthesis of **GH11**. Reaction conditions: (a) AlCl_3_, AcCl, dry DCM, N_2_, 0 °C to 40 °C, 4 h, 30%; (b) AG·HCl, EtOH, cat. 1 M HCl, 80 °C, 8 h, 70%.

The synthesis of the carbazole derivative **GH11** is depicted in Scheme 7. Friedel–Crafts acylation of commercially available carbazole with acetyl chloride and AlCl_3_ (step a) afforded the bis-acetylated intermediate **12** in moderate yield, with selective formation of the disubstituted product. Subsequent condensation of **12** with AG·HCl under standard conditions (step b) gave **GH11** in good yield and purity after washings with MeOH.

**Scheme 8:**
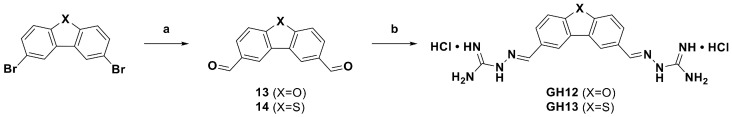
Synthesis of **GH12** and **GH13**. Reaction conditions: (a) (1) *n*-BuLi, dry Et_2_O, N_2_, −78 °C to −30 °C, 1 h, (2) dry DMF, r.t., 3 h, 76% (**13**), 74% (**14**); (b) AG·HCl, EtOH, cat. 1 M HCl, 80 °C, 8 h, 71% (**GH12**), 97% (**GH13**).

The synthesis of symmetric dibenzofuran and dibenzothiophene derivatives **GH12** and **GH13** is shown in Scheme 8. Bis-formylation of 2,8-dibromodibenzo[*b*,*d*]furan and 2,8-dibromodibenzo[*b*,*d*]thiophene was achieved via *n*-BuLi-mediated metal–halogen exchange followed by quenching with DMF, affording intermediate **13** and **14** in good yields (step a). Subsequent condensation of these dialdehydes with AG·HCl under standard conditions (step b) provided **GH12** and **GH13**, respectively.

**Scheme 9:**
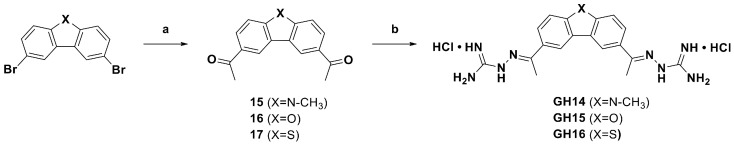
Synthesis of **GH14**-**GH16**. Reaction conditions: (a) (1) *n*-BuLi, dry THF, N_2_, −78 °C to r.t., 1 h, (2) *N*-methyl,*N*-ethoxy*-N*-methylacetamide, −78 °C to r.t., 3 h, 48% (**15**), 37% (**16**), 42% (**17**); (b) AG·HCl, EtOH, cat. 1 M HCl, 80 °C, 8 h, 92% (**GH14**), 93% (**GH15**),72% (**GH16**).

The synthesis of symmetric, methyl derivatives **GH14**–**GH16** is shown in Scheme 9. Bis-acetylations of 3,6-dibromo-9-methyl-9H-carbazole, 2,8-dibromodibenzo[*b,d*]furan, and 2,8-dibromodibenzo[*b*,*d*]thiophene were achieved via *n*-BuLi-mediated metal–halogen exchange followed by quenching with *N*-methoxy-*N*-methylacetamide, affording bis-acetyl intermediates **15**–**17** in moderate, unoptimized yields (step a). Subsequent condensation of **15**–**17** with AG·HCl under standard conditions (step b) furnished **GH14**–**GH16** in good yield and purity after simple washings with dichloromethane.

In conclusion to this section, we would like to emphasize that once the synthetic accessibility of the target derivatives had been demonstrated, no extensive optimization of the reaction conditions or investigation of regioselectivity was undertaken.

### 2.2. Screening of the Guanyl Hydrazones Library by the G-quadruplex on Controlled Pore Glass (G4-CPG) Assay

The library of the above shown synthetic compounds was firstly evaluated by the G4-CPG assay [19]. Due to the poor solubility of guanyl hydrazones **GH1**–**GH16** in pure water at the mM concentration typically used to prepare the stock solutions, these were dissolved in pure DMSO. Then, the compounds were evaluated for their solubility and stability at the concentration and in the washing/releasing solutions used in the G4-CPG assay. All the tested compounds proved to be fully soluble and stable in the assay experimental conditions.

The UV spectra of the investigated compounds in the washing solution (50 mM KCl, 10% DMSO, 10% EtOH) are reported in Figure S1, which clearly emphasizes that, as expected, the compounds carrying the same tricyclic core (as grouped in Figure 1) gave similar UV profiles.

For each compound, tests were carried out to evaluate first the unspecific binding on nude CPG support, and then the ability to bind the oligonucleotide sequences forming G-quadruplex and duplex structures bound on the CPG solid support. Notably, no unspecific binding was observed for all tested compounds (Table 1).

Overall, all compounds showed a significant interaction with both the hybrid telomeric G-quadruplex tel26 and the parallel oncogenic G-quadruplex c-myc (Table 1). Their ability to selectively recognize one of the two specific G-quadruplex topologies was promptly evaluated by calculating the ratio between the percentage of ligand bound to the hybrid G-quadruplex tel26 and the ligand bound to the parallel G-quadruplex c-myc (Table 1). The ligands here tested showed selectivity indexes in the range 0.74–1.2, proving in the assay conditions a slight preference of **GH1**, **GH2**, **GH3,** and **GH7** for tel26 over c-myc G-quadruplex (selectivity index > 1.0) and of **GH5**, **GH8**, **GH9**, **GH10**, **GH11**, **GH12**, **GH14,** and **GH15** for c-myc over tel26 G-quadruplex (selectivity index < 1.0).

Then, based on the ratio between the percentage of ligand bound to the G-quadruplex models and the ligand bound to the control duplex, a selectivity index indicating the ability of each ligand to discriminate between these structures was calculated (Table 1 and Figure S2). Notably, many of the tested ligands proved to be able to selectively bind the G-quadruplex models with respect to the control duplex. Particularly, compounds carrying only one guanyl hydrazone group showed lower percentages of ligand bound to the control duplex ds27, and thus higher selectivity indexes, compared to the compounds bearing two guanyl hydrazone groups (Table 1 and Figure S2).

More in detail, the ligands with a methyl group installed at the hydrazone carbon atom overall showed a slightly higher selectivity towards G-quadruplex sequences compared with their corresponding unmethylated congeners (**GH16** > **GH13**, **GH6** > **GH5**, **GH15** > **GH12** and **GH4** > **GH3**). Moreover, among dibenzo[*b,d*]thiophene derivatives, *para*-substituted **GH8** showed higher selectivity than *ortho*-substituted **GH9** towards G-quadruplex structures.

Based on these data, nine monosubstituted compounds (**GH1**, **GH2**, **GH3**, **GH4**, **GH5**, **GH6**, **GH7**, **GH8**, and **GH9**) were selected out of the sixteen starting compounds for further consideration, and advanced to more in-depth studies. In addition, the disubstituted analogue of the most G-quadruplex-selective ligand **GH6**, i.e., **GH15**, was also included in the successive investigation as a control.

Considering their selectivity index values, the order of selectivity among the ten selected ligands, going from the most to the least selective compound for the hybrid G-quadruplex tel26, was as follows: **GH6** > **GH2** > **GH4** > **GH3** = **GH1** > **GH5** > **GH7** > **GH8** > **GH9** > **GH15**. In turn, the order of selectivity, from the most to the least selective compound for the parallel G-quadruplex c-myc, was as follows: **GH6** > **GH5** > **GH2** > **GH4** > **GH3** > **GH1** > **GH8** > **GH7** = **GH9** > **GH15**.

To summarize, our results indicated that the monosubstituted guanyl hydrazone-based ligands showed approximately the following order of selectivity for both target G-quadruplex structures: dibenzofuran derivatives > pyrido [3,4-*b*]indole derivatives > 9H-fluorene derivative > dibenzothiophene derivatives.

### 2.3. CD Titrations and Melting Experiments

The G-quadruplex-forming oligonucleotides, as well as the control duplex, were prepared for the CD experiments by annealing the DNA solutions at 2 μM concentration, in 20 mM KCl, 5 mM potassium phosphate buffer (pH 7) for tel26 and ds12 or 10 mM Tris-HCl buffer (pH 7) for pu22. Particularly, pu22 was analyzed in 10 mM Tris-HCl buffer, considering that in the 20 mM KCl, 5 mM potassium phosphate buffer used for tel26 and ds12 or even in the presence of lower amounts of potassium ions (such as 2.5 or 1 mM) the pu22 G-quadruplex was so stable that its melting temperature (T_m_) value, as well as the related ΔT_m_ values in the presence of each ligand, could not be accurately determined.

In the selected experimental conditions, the CD profiles of the analyzed sequences (Figure S3, left panels) confirmed that, in line with the literature, tel26 mainly folded into a hybrid 2-type G-quadruplex, featured by a positive CD band with maximum centered at 290 nm and a shoulder at 270 nm [20], pu22 into a parallel G-quadruplex, characterized by a positive band centered at 263 nm, and a negative one with minimum at 240 nm [21], and ds12 into a B-DNA duplex structure, having a positive band with a maximum at 281 nm along with a negative band with a minimum at 253 nm [22]. Then, the three oligonucleotides were titrated with increasing amounts (up to 2 molar equivalents) of each ligand, and the corresponding CD spectra were recorded after each addition (Figures S4–S13, left panels).

CD titration experiments of tel26 showed that all ligands produced an increase of the 290 nm band intensity, with the only exception of **GH2** and **GH15** (Figures S5A and S13A, respectively), and the most marked effect was observed for **GH9** (Figure S12A). Additionally, a reduction in the 270 nm shoulder intensity was observed, with the strongest effects found in the case of **GH9** and **GH15** (Figures S12A and S13A, respectively). Moreover, a red shift of the 290 nm maximum band of tel26 was detected in the titration with **GH15** (Figure S13A).

On the other hand, in the CD titration experiments of pu22 all compounds caused both an intensity increase of the 263 nm maximum band and of the 240 nm minimum band (Figures S4–S13, panels B).

Finally, most ligands did not produce any significant variation in the CD profile of the control duplex (Figures S4–S13, panels C). Indeed, only slight changes were observed, such as a very small increase of the 281 nm maximum band in the titration with **GH7** (Figure S10C), as well as a decrease of the 253 nm minimum band with **GH3**, **GH4,** and **GH9** (Figures S6C, S7C and S12C), while an increase of the minimum band was found with **GH15** (Figure S13C). Additionally, an increase in intensity in the 300–320 nm region was observed for the ds12/**GH15** system (Figure S13C). The slight changes in CD spectra related to the control duplex titrations suggested a weak binding and/or potential groove binding without conformational changes or a subtle ligand intercalation of the tested compounds.

Overall, the above results, indicating a stronger affinity of the investigated compounds for G-quadruplex structures than to duplex DNA, well correlated with those obtained by the G4-CPG assay. Moreover, the highest effects observed on the duplex DNA upon treatment with **GH7**, **GH9,** and **GH15** were in good agreement with the UV-based binding assay data (Table 1 and Figure S2), since these ligands were among the compounds with the highest binding percentage towards the duplex-functionalized CPG support in the investigated series.

In parallel, the effects of the studied ligands on the thermal stability of the target DNAs were evaluated by CD melting experiments on all DNA/ligand mixtures, in 20 mM KCl, 5 mM potassium phosphate buffer (pH 7) for tel26 and ds12 or in 10 mM Tris-HCl buffer (pH 7) for pu22, by measuring the ligand-induced change in the melting temperature (ΔT_m_) of the G-quadruplex models and the control duplex. CD melting curves of tel26, pu22, and ds12 in the absence or presence of each ligand (DNA/ligand 1:2 ratio) were recorded by following the CD signal at the wavelength of the maximum intensity (290, 263, and 253 nm for tel26, pu22, and ds12, respectively).

**Figure 2:**
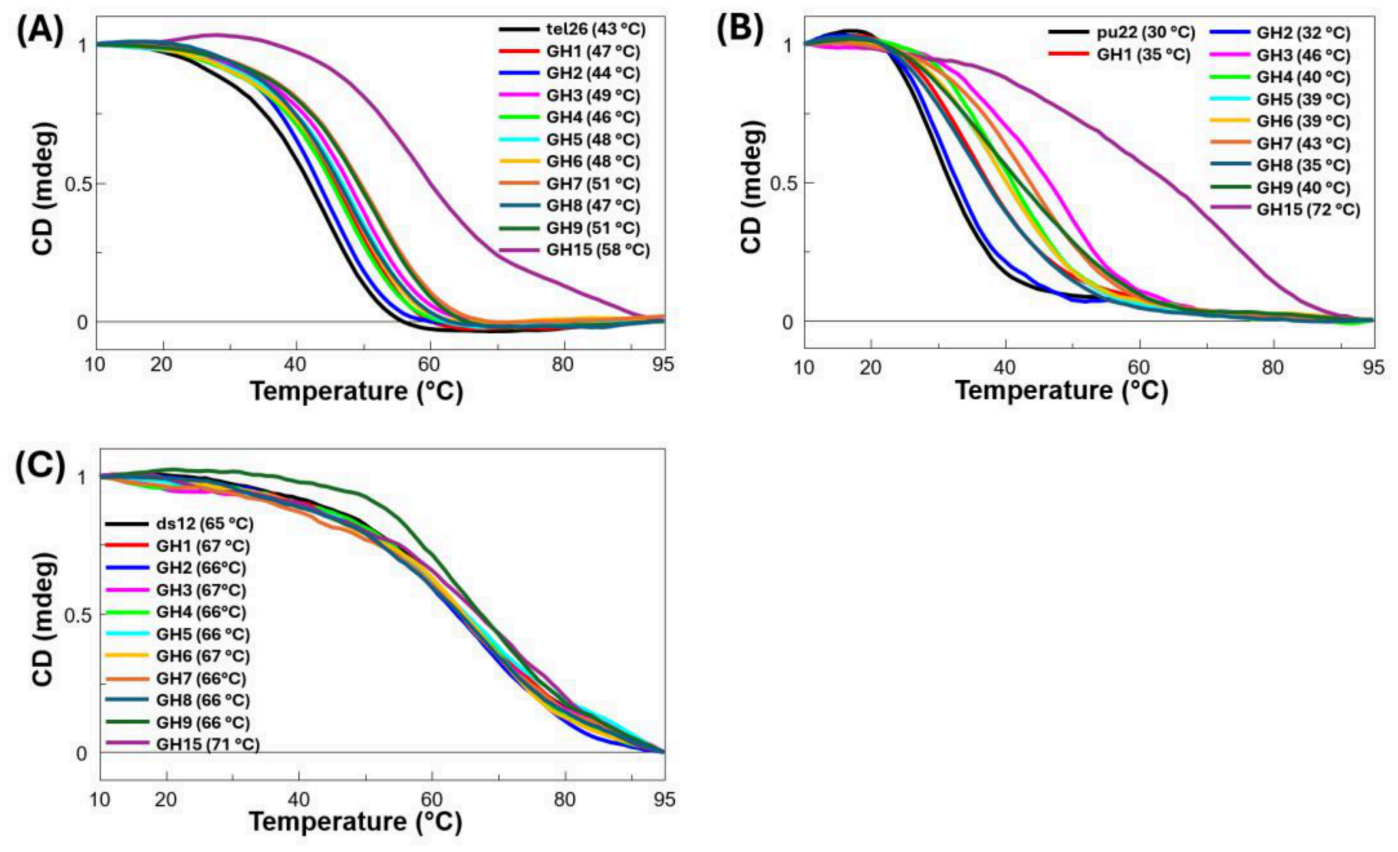
Normalized CD melting curves for (**A**) tel26, (**B**) pu22, and (**C**) ds12 in the presence of the investigated ligands (2 equiv) in 20 mM KCl, 5 mM potassium phosphate buffer (pH 7) for tel26 and ds12, recorded at 290 and 253 nm respectively, and in 10 mM Tris-HCl buffer (pH 7) for pu22, recorded at 263 nm. The melting temperature (T_m_) values for the free DNA or the 1:2 DNA/ligand mixtures are reported in brackets.

Melting temperature (T_m_) values of 43, 30, and 65 °C were found for free tel26, pu22, and ds12, respectively (Figure S3, right panels). The T_m_ and ΔT_m_ values for all the studied DNA/ligand systems (Figure 2 and Figure S4–S13, right panels) are summarized in Table 2.

All ligands, with the only exception of **GH2**, displayed high stabilizing ability on both G-quadruplex targets. Indeed, ΔT_m_ values in the range +3–+15 °C were found in the case of tel26/ligand mixtures, while ΔT_m_ values in the range +5–+42 °C were found in the case of pu22/ligand mixtures.

The strongest stabilizing effects on tel26 and pu22 G-quadruplexes were detected for the control ligand **GH15**. However, this compound also strongly stabilized the model duplex, thus confirming the low G-quadruplex vs. duplex selectivity found for this disubstituted compound in the G4-CPG assay. Notably, all the other ligands produced no stabilization on the control duplex, since all the determined ΔT_m_ values in these cases were within the experimental error (+1–+2 °C). Taken together, our results indicated the monosubstituted compounds as selective and effective G-quadruplex ligands.

### 2.4. Fluorescence Spectroscopy Studies

Aiming at a deeper investigation of the interaction between tel26, pu22, and ds12 and the selected ligands, fluorescence spectroscopy titrations were performed to determine the corresponding binding constants and stoichiometries. In these experiments, the ligand concentration is kept constant and increasing amounts of oligonucleotide are added. Then, the fraction of bound ligand is calculated from the fluorescence intensity values obtained and plotted as a function of DNA concentration. Finally, fitting of these data with an independent and equivalent site model [23] allows for calculating the binding constant (K_b_) and binding stoichiometry (n) values.

Before performing the titration experiments, fluorescence spectra of the free ligands were recorded (Figure S14). The highest fluorescence intensities were found for **GH2**, **GH3**, **GH4**, **GH7,** and **GH15**. Moreover, considering that for all ligands, with the only exception of **GH3** and **GH4**, the excitation wavelengths used in the titration experiments fall in the same range of the oligonucleotide absorption spectra, control titrations of the 20 mM KCl, 5 mM potassium phosphate buffer (pH 7) with free tel26, pu22, and ds12 were performed. Only for tel26 and pu22 G-quadruplexes significant fluorescence intensities were found in the same range of emission of the ligands (Figure S15). Thus, the intrinsic fluorescence intensities of the G-quadruplexes were taken into account for the following titration experiments by subtracting the fluorescence contribution of the free DNA sequences from the fluorescence values obtained for the mixtures of tel26 or pu22 with the selected ligands, excluding **GH3** and **GH4**. However, considering that operating a subtraction of the DNA contribution assumes perfect linearity between DNA fluorescence and its concentration—so not taking into account the potential quenching/enhancement upon interaction between DNA and ligand—the binding parameters resulting from fitting the isotherms of fluorescence titrations using this simplified model should be considered only as rough estimates of real binding constants and stoichiometries.

Successively, fluorescence titration experiments were performed at a fixed concentration (2.0 μM) of ligand by adding increasing amounts of tel26, pu22, and ds12 (up to 10 μM).

By evaluating the titrations before subtracting the oligonucleotide contribution, an enhancement in fluorescence intensity was observed for most ligands (**GH1**, **GH4**, **GH5**, **GH6**, **GH8, GH9,** and **GH15**) upon addition of increasing concentrations of both tel26 and pu22 (Figures S16A,B,D,E, Figures S19–S21 panels A,B,D,E, and Figures S23–S25 panels A,B,D,E respectively). On the other hand, **GH2** showed a quenching in its fluorescence intensity when titrated with tel26 (Figure S17A,D), while an enhancement in fluorescence intensity up to 1 μM DNA concentration followed by a quenching were observed when titrated with pu22 (Figure S17B,E). Finally, **GH3** and **GH7** showed a significant fluorescence quenching followed by a slight enhancement along with a blue shift of the maximum of fluorescence intensity when titrated with both tel26 and pu22 (Figures S18A,B,D,E and S22A,B,D,E respectively).

By analyzing all the fluorescence data after proper subtraction of DNA contribution, the following results were obtained. As far as the titrations with tel26 are concerned, only for **GH9** a fluorescence enhancement was observed (Figure S24D, red curve), no significant intensity variation was observed for **GH8** (Figure S23D, red curve), whereas all remaining ligands showed a quenching in their fluorescence intensity upon addition of increasing amounts of tel26 (Figures S16–S22 and S25, panels D, see red curves).

In the case of the titrations with pu22, **GH1** and **GH8** showed a fluorescence enhancement (Figures S16E and S23E, respectively, see red curves), **GH2**, **GH5**, **GH7,** and **GH15** displayed a quenching of fluorescence intensity (Figures S17E, S20E, S22E, and S25E, respectively, see red curves), while for **GH6** and **GH9** no variation in their fluorescence intensities were observed upon subtraction of the free pu22 fluorescence contribution (Figures S21E and S24E, see red curves).

Finally, for titrations involving the control duplex no subtraction was required, since the duplex did not give any relevant intrinsic fluorescence. By observing the variations of fluorescence of the ligand upon addition of ds12, only **GH1** showed a slight enhancement in its fluorescence intensity (Figure S16C,F), while **GH4**, **GH6**, **GH8,** and **GH9** did not show any significant variation (Figures S19C,F, S21C,F, S23C,F, and S24C,F). On the other hand, **GH2**, **GH5**, **GH7,** and **GH15** displayed a quenching of the fluorescence intensity upon increasing the duplex concentration (Figures S17C,F, S20C,F, S22C,F, and S25C,F). Similarly to its behavior when treated with the G-quadruplexes structures, **GH3** showed a significant quenching, followed by a slight increase in fluorescence intensity (Figure S18C,F). Moreover, a blue shift of fluorescence maximum was observed for both **GH3** and **GH7** (Figures S18C and S22C).

Overall, except for **GH2**, **GH3,** and **GH7**, no significant variation of fluorescence intensity was found for the remaining ligands when titrated with the control duplex compared to their corresponding titrations with G-quadruplex structures, thus suggesting a stronger affinity of the ligands for the G-quadruplex targets than for the control duplex.

**Figure 3:**
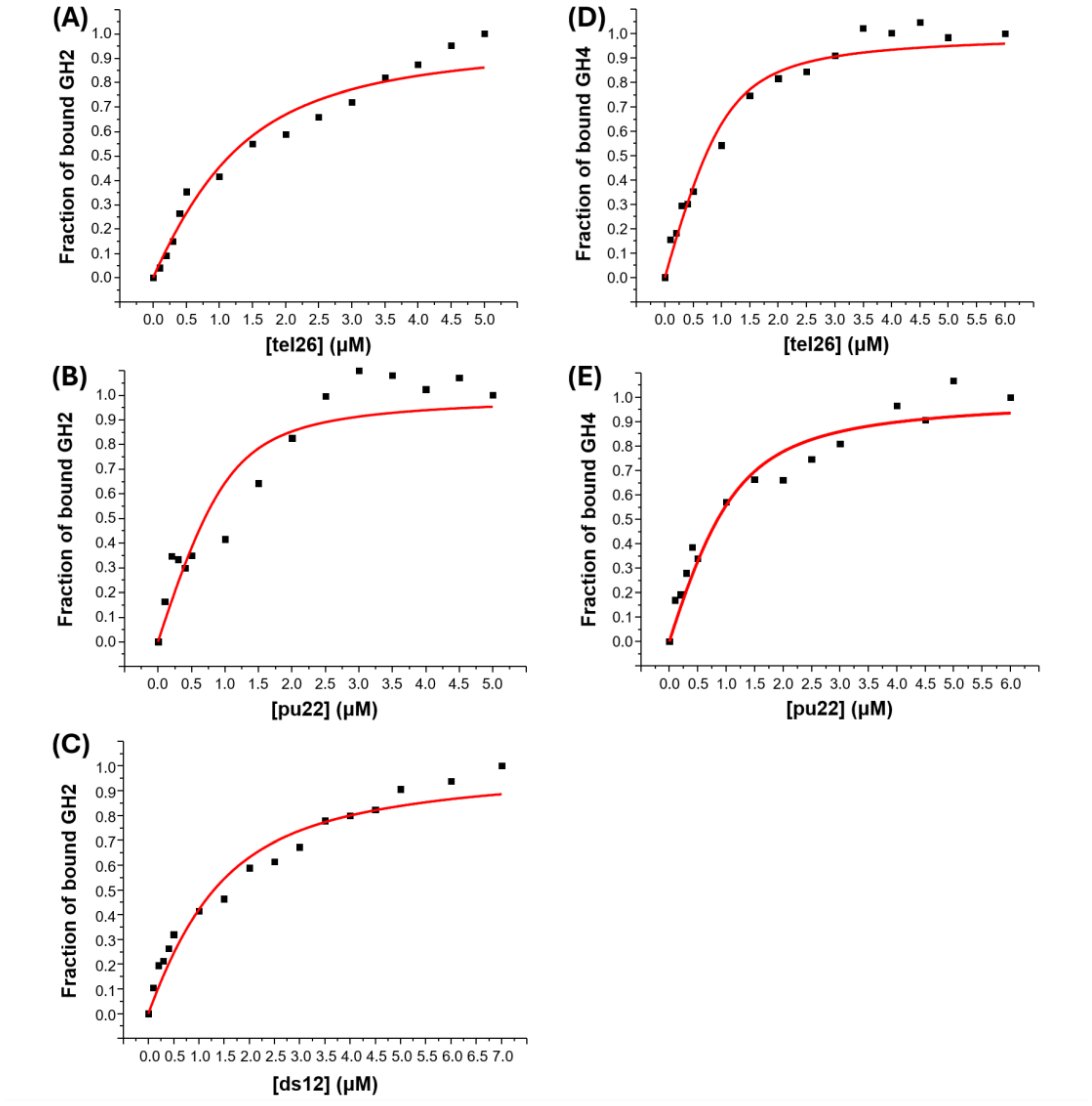
(**Left**) panels: Representative binding curves obtained by plotting the fraction of bound **GH2** to (**A**) tel26, (**B**) pu22, and (**C**) ds12 as a function of DNA concentration. (**Right**) panels: Representative binding curves obtained by plotting the fraction of bound **GH4** to (**D**) tel26 and (**E**) pu22 as a function of DNA concentration. The black squares represent experimental data; the red line represents the best fit obtained using an independent and equivalent-site model.

**Figure 4:**
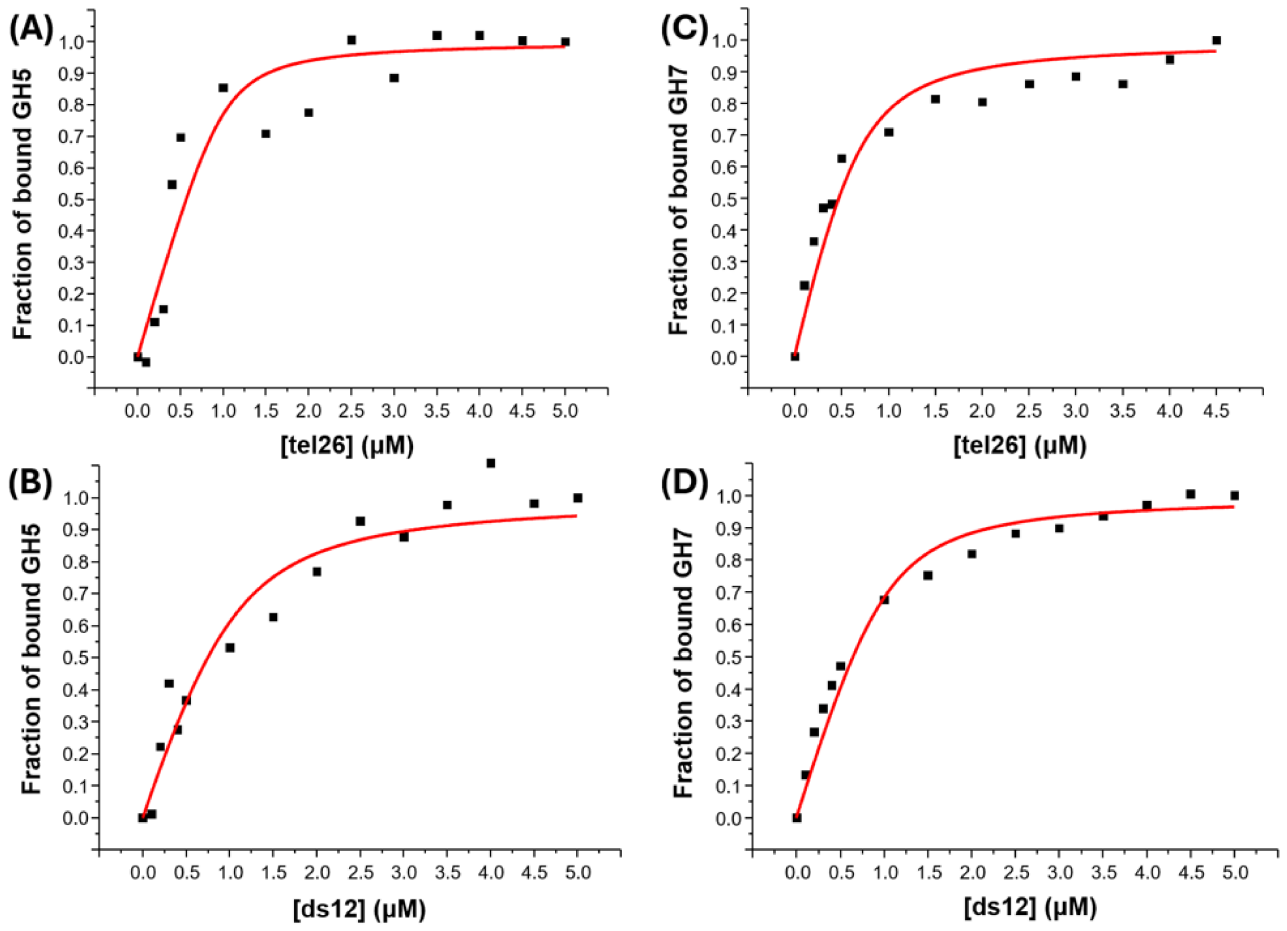
(**Left**) panels: Representative binding curves obtained by plotting the fraction of bound **GH5** to (**A**) tel26 and (**B**) ds12 as a function of DNA concentration. (**Right**) panels: Representative binding curves obtained by plotting the fraction of bound **GH7** to (**C**) tel26 and (**D**) ds12 as a function of DNA concentration. The black squares represent experimental data; the red line represents the best fit obtained using an independent and equivalent-site model.

Unfortunately, not for all DNA/ligand systems a well-defined fluorescence intensity increase or decrease after subtraction of the fluorescence contribution of free DNA was observed, thus not allowing a quantitative analysis for all the systems investigated by fluorescence spectroscopy titrations. Indeed, it was possible to obtain binding constants and stoichiometries only in the case of tel26-pu22-ds12/**GH2**, tel26-pu22**/GH4**, tel26-ds12/**GH5,** and tel26-ds12**/GH7** systems, for which a fitting of the experimental points following a defined hyperbolic trend could be performed by exploiting the independent and equivalent-site model. The fitting curves related to the fluorescence data of **GH2** and **GH4** with tel26 and pu22 G-quadruplexes and ds12 duplex are shown in Figure 3, while those related to **GH5** and **GH7** with tel26 and ds12 are shown in Figure 4. In parallel, the K_b_ and n values for all the studied DNA/ligand systems are summarized in Table 3.

From the obtained K_b_ values, it emerged that all these ligands strongly interacted with tel26 and pu22 G-quadruplexes; however, binding constants of the same order of magnitude as obtained with the G-quadruplexes were found in the interaction of **GH2**, **GH5,** and **GH7** with ds12. Notably, no binding towards the ds12 was in turn observed for **GH4**, which was found to be the strongest and most selective ligand among the ones for which K_b_ values could be obtained by fluorescence spectroscopy titrations. A 1:2 binding stoichiometry was found for all DNA/ligand systems, with the only exception of tel26/**GH7** system, for which a 1:3 binding stoichiometry was observed. The DNA/ligand stoichiometry higher than 1:2—which is typically observed for G-quadruplex ligands targeting the two outer G-quartets—found for the tel26/**GH7** system compared to the other systems could be ascribed to the higher lipophilicity of the dibenzothiophene core compared to pyrido [3,4-*b*]indole and dibenzofuran ones, which can result in targeting more than two binding sites within G-quadruplex structures.

Finally, comparison of both the affinity and selectivity trends observed by analysis of the binding constants determined by fluorescence titration for the above four ligands towards tel26, pu22, and ds12 proved that the obtained data essentially match the trends found with the G4-CPG assay. This analysis thus confirmed that, among the monosubstituted investigated compounds, the pyrido [3,4-*b*]indole and dibenzofuran derivatives are more selective G-quadruplex ligands than the dibenzothiophene derivatives.

### 2.5. Docking Studies

Aiming at investigating more in detail the binding mode of the here described mono- and bis-guanyl hydrazone-derivatized tricyclic compounds towards G-quadruplex and duplex models, molecular docking studies were carried out. **GH6** and **GH15** were selected as models for mono- and bis-guanyl hydrazone-derivatized tricyclic compounds, respectively, considering that they experimentally showed the highest difference in terms of G-quadruplex vs. duplex selectivity index, based on the G4-CPG assay results, among the ligand pairs substituted with one or two guanyl hydrazone moieties here investigated. Moreover, **GH1**, **GH4,** and **GH8** were also included, having the same pendant group as **GH6** but differing for their cores (9H-fluorene, pyrido [3,4-*b*]indole and dibenzothiophene, respectively, vs. dibenzofuran), to evaluate the differences produced by the central scaffold in binding the targets as well as the control.

As G-quadruplex and duplex models, PDB-deposited structures containing either accessible outer G-quartets and grooves, for the G-quadruplex models, or accessible intercalative binding sites and grooves, for the duplex model, were selected. These structures were, in detail, those corresponding to hybrid-2 tel26 G-quadruplex (PDB 5MVB) [24], parallel pu22 G-quadruplex (PDB 2L7V) [25] and B-like duplex ds12 (PDB prepared from 1NAJ) [26,27].

**Figure 5:**
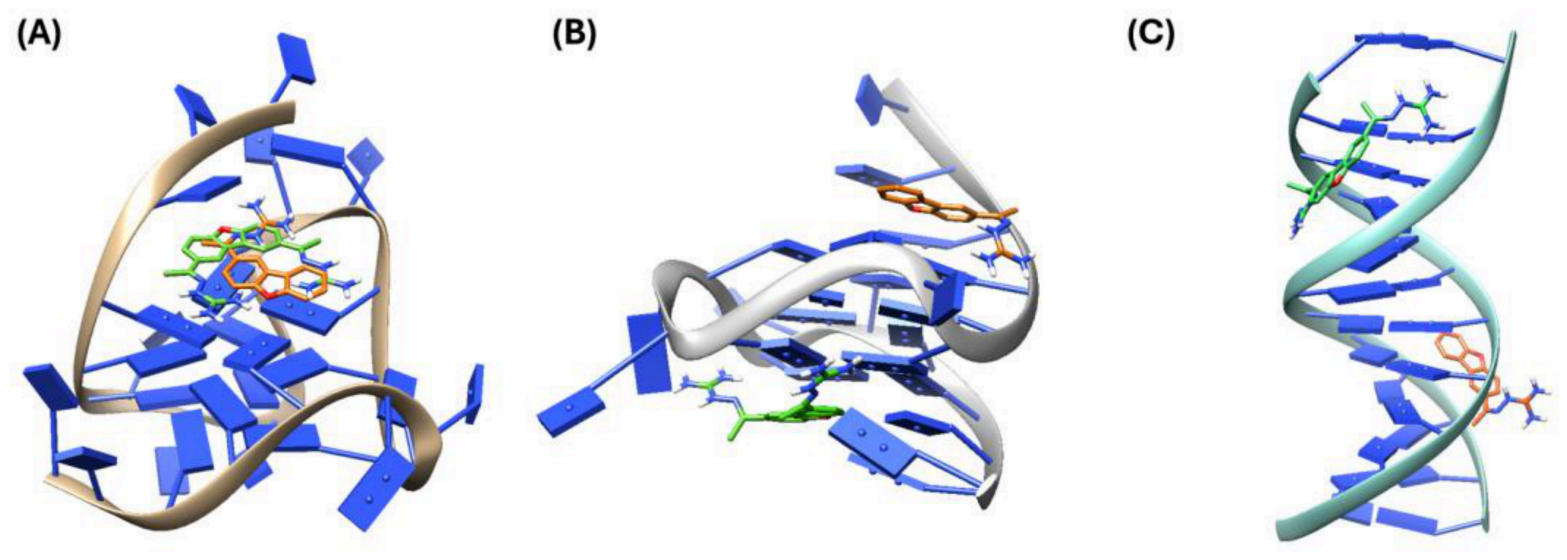
Binding modes of **GH6** and **GH15** when docked to (**A**) tel26 G-quadruplex (PDB 5MVB), (**B**) pu22 G-quadruplex (PDB 2L7V), and (**C**) ds12 duplex (from PDB 1NAJ). Ligands and G-quadruplexes/duplex are represented as sticks and ribbon, respectively. **GH6** and **GH15** are colored orange and green, respectively. The 5′- and 3′-end of the G-quadruplexes are at the top and bottom, respectively.

In the case of tel26 G-quadruplex, both **GH6** and **GH15** targeted the 5′-end G-quartet (Figure 5A). On the other hand, in the case of pu22 G-quadruplex, **GH6** bound the 5′-end G-quartet, whereas **GH15** preferentially targeted the 3′-end G-quartet (Figure 5B). Additionally, in the case of ds12 duplex, two different binding sites were found for the two ligands; indeed, **GH6** and **GH15** bound two different regions of the minor groove of the duplex (Figure 5C).

In detail, the core and guanyl hydrazones of **GH6** and **GH15** were almost coplanar when the two ligands interacted with the upper G-quartet of the hybrid tel26 G-quadruplex (Figure 5A). In turn, in the interaction with the parallel pu22 G-quadruplex only the ligand cores were located on top of the G-quartets, while the guanyl hydrazone moieties of both ligands pointed towards the grooves/loops (Figure 5B). As far as the ds12 duplex is concerned, for **GH6,** the pendant group pointed in the opposite direction with respect to the minor groove, whereas for **GH15** both pendant groups pointed towards the minor groove, with one of them being in close proximity to the intercalation site (Figure 5C).

Notably, all the observed binding modes correlated well with the effects observed on the G-quadruplexes and duplex by CD analysis. Indeed, the CD profiles of both the G-quadruplexes were significantly affected by ligand binding (Figures S9A,B and S13A,B), indicative of a tight interaction with the G-quartets as observed in Figure 5A,B. In parallel, no effect on the CD profile of the duplex was detected upon addition of **GH6** (Figure S9C), suggesting a complementary binding within the duplex grooves as found in Figure 5C, while significant variation in the CD signal of ds12 duplex was observed upon treatment with **GH15** (Figure S13C), suggesting in this case a tight interaction of one pendant group of the ligand with an intercalation site of the duplex, as depicted in Figure 5C.

In detail, the core of **GH6** was stacked onto G12 and G16 (Figure S26A), while the core of **GH15** was sandwiched between A3 and G12 of tel26 G-quadruplex, and its guanyl hydrazone group was engaged in electrostatic interactions with the phosphate group of G16 (Figure S26B). As far as the interactions with pu22 G-quadruplex are concerned, the core of **GH6** was stacked on G7 and G11 and its guanyl hydrazone group formed an H-bond with the O4′ of G8 (Figure S27A), while the core of **GH15** was stacked on G13 and its two guanyl hydrazone moieties were involved in two H-bonds and an electrostatic interaction with G13, G18, and A25 (Figure S27B). Finally, in the case of ds12 duplex, the guanyl hydrazone group of **GH6** formed an electrostatic interaction with the phosphate group of A18 and an H-bond with O3′ of A17 (Figure S28A). In turn, **GH15** gave hydrophobic interactions with G2, C23, and G24 located at the intercalation site and its guanyl hydrazone groups formed two H-bonds with the O4′ of C3 and G22 (Figure S28B).

The binding energies obtained for these binding poses (Table 4) confirmed that, compared to mono-substituted **GH6**, the bis-substituted **GH15** showed higher affinity towards G-quadruplexes but also higher affinity for the control duplex, in line with the lower G-quadruplex vs. duplex selectivity of **GH15** than **GH6** experimentally derived by the G4-CPG assay and CD studies.

Upon comparison of the binding energies (Table 4) of **GH1**, **GH4**, and **GH8** with **GH6** and **GH15**, as experimentally found it emerged that: (i) regardless of the kind of central scaffold, mono-substituted compounds have lower G-quadruplex and duplex affinities compared to the bis-substituted **GH15**, and (ii) pyrido [3,4-*b*]indole- and dibenzofuran-based ligands have lower affinity for the control duplex, and thus higher G-quadruplex selectivity, than 9H-fluorene- and dibenzothiophene-based ones. The latter insight is a consequence of the different binding modes observed for the mono-substituted **GH1**, **GH4**, **GH6**, and **GH8**. Indeed, in the case of tel26 G-quadruplex, these ligands shared a similar binding site, i.e., the 5′-end G-quartet, wherein they adopted different poses (Figure S29A). In the case of pu22 G-quadruplex, **GH1**, **GH4**, and **GH6** targeted the 5′-end G-quartet with different binding poses, while **GH8** showed a preference for the 3′-end G-quartet (Figure S29B). Finally, in the case of the ds12 duplex **GH4** and **GH6** showed a similar binding pose in the minor groove, whereas **GH1** targeted the duplex intercalation site and **GH8** the minor groove on the opposite side of **GH4** and **GH6**, thus resulting in higher binding energies for both **GH1** and **GH8** than **GH4** and **GH6** (Figure S29C).

### 2.6. Biological Assays

The anticancer activity of all the mono-guanyl hydrazone-derivatized tricyclic compounds and of the bis-guanyl hydrazone compounds **GH15**, included as a reference, was evaluated by MTT assays on human MCF7 breast cancer cells and, in parallel, on HaCaT normal cells used as control cell line.

All the selected derivatives showed dose-dependent cytotoxic effects upon 72 h incubation with the tested cell lines (Figures S30 and S31) and IC_50_ values ≤ 8 μM (Table 5), with the highest activity on cancer cells (IC50 ≤ 4 μM) found for **GH1**, **GH3**, **GH4**, **GH5**, **GH6**, **GH7,** and **GH9** and the lowest for **GH8**, **GH15**, and particularly **GH2** (Table 5).

The highest cancer vs. normal cells selectivity was found for **GH4**, with **GH3**, **GH7**, **GH8,** and **GH9** also being somehow selective. In contrast, no selectivity was observed for **GH1**, **GH2**, **GH5**, **GH6,** and **GH15** (Table 5).

Overall, these preliminary data indicated that the bis-guanyl hydrazone-derivatized compound **GH15** showed a low anticancer activity as well as a low selectivity for MCF7 cancer cells, whereas some mono-guanyl hydrazone-derivatized tricyclic compounds in the investigated series may be of interest in view of developing selective anticancer agents. Notably, at the dose of 3.12 μM, **GH4** and **GH9** reduced the cell viability of MCF7 cancer cells by 20 and 40%, respectively, while being completely inactive on HaCaT cells.

## 3. Materials and Methods

### 3.1. Chemical Synthesis

#### 3.1.1. General Information

Oven-dried glassware was used to carry out all the chemical reactions, performed using anhydrous solvents under a nitrogen atmosphere. Solvents were purchased from Sigma Aldrich and used as such. Chemical reagents were obtained from Sigma Aldrich (Burlington, MA, USA), Fluorochem (Cork, Ireland), and TCI (Zwijndrecht, Belgium). All the intermediates and final products were purified by flash chromatography using high purity grade silica gel (Merck Grade, pore size 60 Å, 230–400 mesh particle size, Sigma-Aldrich, Burlington, MA, USA) as a stationary phase. Alternatively, purification was carried out on a BIOTAGE^®^ system using Biotage Sfar Duo cartridges (Biotage, Uppsala, Sweden) (4, 10 or 25 g) for direct phase chromatography or Biotage Sfar Duo C_18_ cartridges (Biotage, Uppsala, Sweden) (6, 12 or 30 g) for reverse phase chromatography. Thin layer chromatography (TLC) entailing Merck-precoated 60F_254_ plates allowed monitoring the reaction mixtures by using UV light at 254 nm as a direct detection method, or by charring either with a phosphomolybdic acid ethanolic solution, or a potassium permanganate or a ninhydrin solution. Mass spectra were recorded on a Thermo Fisher (Waltham, MA, USA) LCQ Fleet Ion Trap Mass Spectrometer equipped with UltiMate™ 3000 high-performance liquid chromatography (HPLC) system. HRMS spectra were obtained using a Synapt G2-Si QTof mass spectrometer (Waters, Milford, MA, USA) with ZsprayTM ESI-probe for electrospray ionization (Waters, Milford, MA, USA). ^1^H-NMR and ^13^C-NMR spectra were recorded on Bruker DRX-400 or Bruker DRX-300 instruments (Billerica, MA, USA) in CDCl_3_ or DMSO-d6, according to the compound solubility. Chemical shifts (δ) for proton and carbon signals are quoted relatively to tetramethylsilane as an internal standard and expressed in parts per million (ppm).

#### 3.1.2. General Method for Guanyl Hydrazone Preparation

AG·HCl (1.96 equiv for bis-carbonyl compounds or 0.98 equiv for mono-carbonyl compounds) was added to a vigorously stirred solution of the corresponding carbonyl compound (1.0 equiv) in EtOH. After addition of few drops of a 1 M HCl solution, the resulting mixture was refluxed under stirring and monitored by TLC until completion of the reaction (eluent: 9:1 DCM/MeOH; visualization with ninhydrin). Then the mixture was concentrated under reduced pressure affording a solid residue, which was precipitated from organic solvent, filtered and, if necessary, purified by reverse-phase column chromatography.

#### 3.1.3. Synthesis of 1-(9H-Fluoren-3-yl)ethan-1-one (**1**)

AlCl_3_ (642 mg, 4.81 mmol, 4.0 equiv) was suspended in anhydrous DCM (3 mL) and the resulting mixture cooled to 0 °C under nitrogen atmosphere. Then, AcCl (0.205 mL, 2.89 mmol, 2.4 equiv) was added dropwise. 9H-Fluorene (200 mg, 1.20 mmol, 1.0 equiv) was solubilized in anhydrous DCM (7 mL) and added dropwise to the reaction mixture. The resulting solution was taken at r.t. under stirring for 4 h monitoring by TLC (eluent mixture: 8:2 *n*-Hex/EtOAc, developed in 2,4-dinitrophenylhydrazine). After reaction completion, the reaction was quenched by addition of 1 M HCl (2 mL). The aqueous phase was extracted with DCM (3 × 15 mL) and the combined organic phases were washed with water (40 mL), dried over anhydrous Na_2_SO_4_, filtered, and concentrated under reduced pressure. The resulting crude was purified by direct flash column chromatography (eluent mixture: 15:1 petroleum ether/EtOAc) to afford 178 mg (0.85 mmol, 71% yield) of pure intermediate **1** as an orange solid.

^1^H NMR (400 MHz, CDCl_3_) δ 8.11 (s, 1H), 7.98 (d, *J* = 8.0 Hz, 1H), 7.84–7.76 (m, 2H), 7.56 (d, *J* = 7.1 Hz, 1H), 7.45–7.32 (m, 2H), 3.90 (s, 2H), 2.64 (s, 3H). The spectroscopic data are consistent with those reported in the literature [28].

#### 3.1.4. Synthesis of 2-(1-(9H-Fluoren-3-yl)ethylidene)hydrazine-1-carboximidamide Hydrochloride (**GH1**)

The reaction was performed according to the general method. Ketone **1** (0.85 mmol, 1 equiv), dissolved in EtOH (8.5 mL), was reacted with AG·HCl (90 mg, 0.83 mmol, 0.98 eq) at reflux for 7 h. Then, the reaction mixture was taken to dryness under reduced pressure, obtaining a yellow solid which was triturated with Et_2_O and then purified by BIOTAGE^®^ (Biotage, Uppsala, Sweden) reverse-phase chromatography (eluent mixture: H_2_O/ACN from 0% to 100% ACN), recovering 111 mg (0.37 mmol, 43% yield) of pure target **GH1** as a white solid.

^1^H NMR (400 MHz, DMSO-*d*_6_) δ 11.03 (s, 1H), 8.21 (s, 1H), 8.00 (dd, *J* = 8.1, 1.7 Hz, 1H), 7.99–7.90 (m, 2H), 7.72 (bs), 7.62 (d, *J* = 7.0 Hz, 1H), 7.41 (td, *J* = 7.4, 1.3 Hz, 1H), 7.35 (td, J = 7.4, 1.3 Hz, 1H), 3.97 (s, 2H), 2.40 (s, 3H).

^13^C NMR (101 MHz, DMSO-*d*_6_) δ 155.9, 152.0, 143.7, 143.1, 142.5, 140.5, 135.4, 127.3, 126.9, 125.8, 125.3, 123.4, 120.5, 119.7, 36.4, 14.6.

HRMS ESI (positive mode): [M+H]^+^ 265.2453.

#### 3.1.5. Synthesis of 1-(1-Methyl-2,3,4,9-tetrahydro-1H-pyrido [3,4-*b*]indol-1-yl)ethan-1-one (**2**)

TFA (0.096 mL, 1.25 mmol, 1.0 equiv) and butane-2,3-dione (0.122 mL, 1.38 mmol, 1.1 equiv) were added to a solution of tryptamine (200 mg, 1.25 mmol, 1.0 equiv) in MeOH (10.4 mL) under nitrogen atmosphere. The solution was taken at 60 °C under stirring for 7 h, monitoring by TLC (eluent mixture: 1:1 *n*-Hex/Acetone). Then, the mixture was cooled to r.t. and concentrated under reduced pressure. The resulting residue was poured in a separatory funnel containing a satd. aq. solution of NaHCO_3_ (30 mL) and DCM (30 mL). After phase separation the aqueous phase was extracted with DCM (3 × 30 mL). The combined organic phases were dried over anhydrous Na_2_SO_4_ and then concentrated under reduced pressure. The resulting crude was purified by direct-phase flash column chromatography (eluent mixture from 99:1 to 95:5 DCM/MeOH), obtaining pure **2** (76 mg, 0.33 mmol, 63% yield) as a brown foam.

^1^H NMR (400 MHz, CDCl_3_) δ 8.41 (s, 1H), 7.51 (dd, *J* = 8.0, 1.2 Hz, 1H), 7.35 (d, *J* = 8.0 Hz, 1H), 7.18 (ddd, *J* = 8.0, 7.1, 1.2 Hz, 1H), 7.11 (ddd, *J* = 8.0, 7.1, 1.2 Hz, 1H), 3.33 (m, 1H), 3.02 (m, 1H), 2.77 (m, 2H), 2.35 (s, 3H), 1.57 (s, 3H). The spectroscopic data are consistent with those reported in literature [29].

#### 3.1.6. Synthesis of 2-(1-(1-Methyl-2,3,4,9-tetrahydro-1H-pyrido [3,4-*b*]indol-1-yl)ethylidene)hydrazine-1-carboximidamide Dihydrochloride (**GH2**)

The reaction was performed according to the general method. Ketone **2** (52 mg, 0.228 mmol, 1.00 equiv), dissolved in EtOH (2.5 mL), was reacted with AG·HCl (24.6 mg, 0.223 mmol, 0.98 equiv) and taken at reflux for 14 h. After reaction completion, the mixture was concentrated under reduced pressure and the resulting solid was triturated with Et_2_O (4 × 2.5 mL), DCM (2.5 mL) and EtOAc (3 × 2.5 mL), obtaining a brown solid. The residue was purified by BIOTAGE^®^ (Biotage, Uppsala, Sweden) reverse-phase chromatography (eluent mixture: H_2_O/ACN from 0% to 100% ACN), recovering pure **GH2** as a brown solid (37 mg, 0.104 mmol, 46% yield).

^1^H NMR (400 MHz, DMSO-*d*_6_) δ 11.65 (s, 1H), 11.31 (s, 1H), 10.21 (s, 1H), 7.85 (bs), 7.50 (d, *J* = 7.9 Hz, 1H), 7.43 (d, *J* = 8.2 Hz, 1H), 7.22–7.14 (m, 1H), 7.09–7.01 (m, 1H), 3.54 (m, 1H), 3.33 (m, 1H), 3.06 (m, 1H), 2.91 (m, 1H), 2.09 (s, 3H), 1.93 (s, 3H).

^13^C NMR (101 MHz, DMSO-*d*_6_) δ 156.2, 151.7, 136.6, 129.2, 125.3, 122.5, 119.2, 118.5, 111.6, 107.3, 61.5, 24.0, 18.1, 14.3.

HRMS ESI (positive mode): [M+H]^+^ 285.1827.

#### 3.1.7. Synthesis of 1-(Dimethoxymethyl)-2,3,4,9-tetrahydro-1H-Pyrido [3,4-*b*]indole (**3**)

2,2-Dimethoxyacetaldehyde (0.56 mL, 3.74 mmol, 1.2 equiv) was added to tryptamine (500 mg, 3.12 mmol, 1.0 equiv), dissolved in DCM (31 mL) under stirring and nitrogen atmosphere, followed by a dropwise addition of TFA (1.6 mL, 0.94 mmol, 0.3 equiv). The resulting solution was stirred at r.t. overnight, monitoring by TLC (eluent mixture: 98:2 DCM/MeOH). Then, the mixture was poured in a separatory flask containing 10% NaHCO_3_ aq. solution (30 mL), and after phase separation the aqueous phase was extracted with DCM (3 × 30 mL). The combined organic phases were dried over anhydrous Na_2_SO_4_ and then concentrated under reduced pressure. The crude product was purified by direct-phase flash column chromatography (eluent mixture: 95:5 DCM/MeOH), obtaining pure **3** as a brown foam (370 mg, 1.78 mmol, 48% yield).

^1^H NMR (400 MHz, CDCl_3_) δ 8.49 (s, 1H), 7.50 (d, *J* = 7.8 Hz, 1H), 7.34 (d, J = 8.1 Hz, 1H), 7.16 (m, 1H), 7.09 (m, 1H), 4.48 (d, J = 6.6 Hz, 1H), 4.20 (d, *J* = 6.6 Hz, 1H), 3.59 (s, 3H), 3.52 (s, 3H), 3.38 (m, 1H), 3.04 (m, 1H), 2.89–2.70 (m, 2H), 2.25 (s, 1H). The spectroscopic data are consistent with those reported in the literature [30].

#### 3.1.8. Synthesis of 1-(Dimethoxymethyl)-9H-pyrido [3,4-*b*]indole (**4**)

KMnO_4_ (180 mg, 1.14 mmol, 4.0 equiv) was added to a vigorously stirred solution of 1-(dimethoxymethyl)-2,3,4,9-tetrahydro-1H-pyrido [3,4-*b*]indole **3** (70 mg, 0.284 mmol, 1.0 equiv) in THF (1.4 mL) under nitrogen atmosphere. The mixture was taken at r.t. for 25 h, monitoring by TLC (eluent mixture: 3:7 *n*-Hex/EtOAc). Then, the reaction mixture was filtered over a Celite pad, washing with EtOAc (125 mL). The crude product was purified by direct-phase flash column chromatography (eluent mixture: 3:7 *n*-Hex/EtOAc), obtaining pure **4** as a colorless oil (32 mg, 0.133 mmol, 47% yield).

^1^H NMR (400 MHz, CDCl_3_) δ 9.15 (s, 1H), 8.45 (d, *J* = 5.3 Hz, 1H), 8.13 (dd, *J* = 7.9, 1.0 Hz, 1H), 7.95 (d, *J* = 5.3 Hz, 1H), 7.61–7.53 (m, 2H), 7.29 (ddd, *J* = 7.9, 6.5, 1.6 Hz, 1H), 5.76 (s, 1H), 3.53 (s, 6H). The spectroscopic data are consistent with those reported in the literature [30].

#### 3.1.9. Synthesis of 9H-Pyrido [3,4-*b*]indole-1-carbaldehyde (**5**)

1-(Dimethoxymethyl)-9H-pyrido [3,4-*b*]indole **4** (103 mg, 0.43 mmol, 1 equiv) was loaded into a two-neck flask under nitrogen atmosphere. 1,4-Dioxane (1.0 mL), water (0.5 mL), and AcOH (1.8 mL, 32 mmol, 75 equiv) were subsequently added under vigorous stirring, and the resulting mixture was refluxed for 1 h, monitoring by TLC using the eluent mixture: 7:3 *n*-Hex/EtOAc. To quench the reaction, a satd. aq. solution of NaHCO_3_ (5 mL) was added until neutrality. After phase separation the aqueous phase was extracted with EtOAc (3 × 30 mL), the combined organic phases were dried over anhydrous Na_2_SO_4_ and then concentrated under reduced pressure. The crude product was purified by direct-phase flash column chromatography (eluent mixture: 99:1 DCM/MeOH), obtaining pure **5** as a yellow solid (63 mg, 0.32 mmol, 76% yield).

^1^H NMR (400 MHz, CDCl_3_) δ 10.37 (s, 1H), 10.07 (s, 1H), 8.65 (d, *J* = 5.0 Hz, 1H), 8.18 (m, 2H), 7.68–7.57 (m, 2H), 7.48–7.30 (m, 1H). The spectroscopic data are consistent with those reported in the literature [30].

#### 3.1.10. Synthesis of 2-((9H-Pyrido [3,4-*b*]indol-1-yl)methylene)hydrazine-1-carboximidamide Hydrochloride (**GH3**)

The reaction was carried out according to the general method. 9H-pyrido [3,4-*b*]indole-1-carbaldehyde **5** (30.0 mg, 0.153 mmol, 1.00 equiv), dissolved in EtOH (1.5 mL), was reacted with AG·HCl (16.5 mg, 0.149 mmol, 0.98 equiv) and taken at reflux. After 7 h the reaction mixture was concentrated under reduced pressure, and the resulting solid was triturated with Et_2_O (3 × 2.5 mL). The residue was purified by BIOTAGE^®^ (Biotage, Uppsala, Sweden) reverse-phase chromatography (eluent mixture: H_2_O/ACN from 1% to 100% ACN), recovering pure target **GH3** as a yellow solid (18 mg, 0.062 mmol, 40% yield).

^1^H NMR (400 MHz, DMSO-*d*_6_) δ 12.48 (s, 1H), 11.85 (s, 1H), 8.68 (s, 1H), 8.52 (d, *J* = 5.3 Hz, 1H), 8.41 (d, *J* = 5.3 Hz, 1H), 8.38 (d, *J* = 8.0 Hz, 1H), 7.88 (d, *J* = 8.0 Hz, 1H), 7.73–7.63 (m, 1H), 7.39–7.35 (m, 1H).

^13^C NMR (101 MHz, DMSO-*d*_6_) δ 155.1, 141.9, 135.9, 133.6, 132.3, 131.1, 129.7, 122.3, 120.7, 120.2, 116.6.

HRMS ESI (positive mode): [M+2H]^2+^ 127.1011; [M+H]^+^ 253.1211.

#### 3.1.11. Synthesis of 1-(9H-Pyrido [3,4-*b*]indol-1-yl)ethan-1-one (**6**)

H_2_SO_4_ (0.15 mL, 2.81 mmol, 0.9 equiv) was dissolved in water (16.0 mL) at 0 °C. Then, the solution temperature was raised to r.t., and tryptamine (500 mg, 3.12 mmol, 1.0 equiv) and pyruvaldehyde (40% in H_2_O, 0.51 mL, 3.12 mmol, 1.0 equiv) were sequentially added. The mixture was stirred at r.t. overnight, monitoring by TLC (eluent mixture: 1:1 DCM/EtOAc). A total of 10% NaOH aq. solution (3 mL) was added until pH = 8, leading to the precipitation of a yellow solid, which was filtered and washed with water (10 mL). The mother liquor was extracted with DCM (3 × 20 mL), the combined organic phases were dried over anhydrous Na_2_SO_4,_ and the solvent was removed under reduced pressure. The resulting crude was filtered over a silica plug, washing with 1:1 DCM/EtOAc (50 mL). The solvent was evaporated and the resulting solid, directly dissolved in THF (1.0 mL) under nitrogen atmosphere, was treated with KMnO_4_ (186 mg, 1.18 mmol, 0.4 equiv). The reaction mixture was taken under stirring at r.t. overnight, monitoring by TLC (eluent mixture: 1:1 DCM/EtOAc), and then filtered over a Celite plug, washing with EtOAc (25 mL). The crude product was purified by filtering over a silica plug, washing with 1:1 DCM/EtOAc (25 mL) and obtaining, after drying, pure **6** as a yellow solid (84 mg, 0.40 mmol, 12% yield over 2 steps).

^1^H NMR (400 MHz, CDCl_3_) δ 10.30 (s, 1H), 8.56 (d, *J* = 5.0 Hz, 1H), 8.18–8.14 (m, 2H), 7.69–7.53 (m, 2H), 7.34 (ddd, *J* = 8.0, 6.2, 1.9 Hz, 1H), 2.92 (s, 3H). The spectroscopic data are consistent with those reported in the literature [31].

#### 3.1.12. Synthesis of 2-(1-(9H-Pyrido [3,4-*b*]indol-1-yl)ethylidene)hydrazine-1-carboximidamide Hydrochloride (**GH4**)

The reaction was carried out according to the general method. 1-(9H-pyrido [3,4-*b*]indol-1-yl)ethan-1-one **6** (17 mg, 0.081 mmol, 1.00 equiv), dissolved in EtOH (0.8 mL), was reacted with AG·HCl (8 mg, 0.079 mmol, 0.98 equiv) and taken at reflux. After 7 h the reaction was complete, the mixture was concentrated under reduced pressure, and the resulting solid was triturated with diisopropyl ether (3 × 2.5 mL), recovering pure target **GH4** as a yellow solid (21 mg, 0.069 mmol, 85% yield).

^1^H NMR (400 MHz, DMSO-*d*_6_) δ 12.11 (s, 1H), 12.03 (s, 1H), 8.68 (d, *J* = 5.8 Hz, 1H), 8.58 (d, *J* = 5.8 Hz, 1H), 8.48 (d, *J* = 8.0 Hz, 1H), 8.33 (s, 3H), 7.89 (d, *J* = 8.0 Hz, 1H), 7.75 (t, *J* = 7.5 Hz, 1H), 7.42 (t, *J* = 7.5 Hz, 1H), 2.82 (s, 3H).

^13^C NMR (101 MHz, DMSO-*d*_6_) δ 156.2, 143.4, 134.3, 133.3, 132.3, 132.0, 131.1, 122.9, 121.3, 119.6, 116.9, 113.4, 16.5 (one quaternary carbon was not detected).

HRMS ESI (positive mode): [M+2H]^2+^ 133.9985; [M+H]^+^ 267.1388.

#### 3.1.13. Synthesis of 2-(Dibenzo[*b,d*]furan-2-ylmethylene)hydrazine-1-carboximidamide Hydrochloride (**GH5**)

The reaction was performed according to the general method. Dibenzofuran-2-carbaldehyde (100 mg, 0.509 mmol, 1.0 equiv), dissolved in EtOH (5.0 mL), was reacted with AG·HCl (55 mg, 0.498 mmol, 0.98 equiv) and taken at reflux. After 8 h the reaction was complete and the reaction mixture was concentrated under reduced pressure giving a white solid. This residue was triturated with DCM (3 × 3.0 mL), filtered and purified via BIOTAGE^®^ (Biotage, Uppsala, Sweden) reverse-phase chromatography (eluent mixture: H_2_O/ACN from 0% to 100% ACN), recovering pure target dibenzofuran **GH5** (79 mg, 0.274 mmol, 54% yield) as a white solid.

^1^H-NMR (400 MHz, DMSO-*d*_6_) δ 12.11 (bs, 1H), 8.67 (d, *J* = 1.7 Hz, 1H), 8.36 (s, 1H), 8.16 (dd, *J* = 7.7, 0.8 Hz, 1H), 8.09 (dd, *J* = 8.6, 1.7 Hz, 1H), 7.83 (bs, 3H), 7.79 (d, *J* = 8.6 Hz, 1H), 7.76 (d, *J* = 8.3 Hz, 1H), 7.58 (td, *J* = 8.4, 7.9, 1.3 Hz, 1H), 7.47 (td, *J* = 7.5, 1.0 Hz, 1H).

^13^C-NMR (101 MHz, DMSO-*d*_6_) δ 157.1, 156.5, 156.0, 147.0, 129.5, 128.6, 127.6, 124.5, 124.0, 123.7, 121.7, 121.3, 112.5, 112.4.

HRMS ESI (positive mode): [M+H]^+^ 253.1089.

#### 3.1.14. Synthesis of 1-(Dibenzo[*b,d*]furan-2-yl)ethan-1-ol (**7**)

Dibenzofuran-2-carbaldehyde (100 mg, 0.509 mmol, 1.0 equiv) was dissolved in dry THF (3 mL) at 0 °C under nitrogen atmosphere and the resulting solution was treated with MeMgBr (0.2 mL, 1.22 mmol, 1.2 equiv). The reaction mixture was brought at r.t., stirred for 2.5 h monitoring by TLC (eluent mixture: 8:2 *n*-Hex-EtOAc), and finally quenched upon addition of satd. aq. NH_4_Cl solution (5 mL). After extraction of the aqueous phase with DCM (3 × 15 mL), the combined organic phases were dried over anhydrous Na_2_SO_4_ and concentrated under reduced pressure. The resulting crude was purified via direct flash column chromatography (eluent mixture from 9:1 to 8:2 *n*-Hex-EtOAc), to afford pure alcohol **7** (93 mg, 0.438 mmol, 86% yield) as a white solid.

^1^H NMR (400 MHz, CDCl_3_) δ 7.98–7.89 (m, 2H), 7.62–7.39 (m, 4H), 7.34 (t, *J* = 7.5 Hz, 1H), 5.10–4.99 (m, 1H), 1.58 (d, *J* = 1.3 Hz, 3H). The spectroscopic data are consistent with those reported in the literature [32].

#### 3.1.15. Synthesis of 1-(Dibenzo[*b,d*]furan-2-yl)ethan-1-one (**8**)

Alcohol **7** (93 mg, 0.437 mmol, 1.0 equiv), dissolved in dry DCM (2.1 mL) at 0 °C under a nitrogen atmosphere, was treated with solid DMP (222 mg, 0.525 mmol, 1.2 equiv) and the resulting mixture was taken under stirring at r.t. overnight. The reaction was monitored by TLC (eluent mixture: 8:2 *n*-Hex-EtOAc, developed in 2,4 dinitrophenylhydrazine) and, after completion, quenched by addition of satd. aq. Na_2_S_2_O_3_ (3 mL) and aq. NaHCO_3_ (3 mL). Next, the aqueous phase was extracted with DCM (3 × 15 mL), the combined organic phases were dried over anhydrous Na_2_SO_4_ and then concentrated under reduced pressure. The resulting crude was purified via direct flash column chromatography (eluent mixture: 9:1 *n*-Hex/EtOAc), affording pure ketone **8** (71 mg, 0.337 mmol, 77% yield) as a white solid.

^1^H-NMR (400 MHz, CDCl_3_) δ 8.57 (d, *J* = 1.9 Hz, 1H), 8.09 (dd, *J* = 8.6, 1.9 Hz, 1H), 7.99 (d, *J* = 8.3 Hz, 1H), 7.58 (d, *J* = 7.8 Hz, 2H), 7.50 (t, *J* = 7.0 Hz, 1H), 7.39 (t, *J* = 7.5 Hz, 1H), 2.71 (s, 3H). The spectroscopic data are consistent with those reported in the literature [32].

#### 3.1.16. Synthesis of 2-(1-(Dibenzo[*b,d*]furan-2-yl)ethylidene)hydrazine-1-carboximidamide Hydrochloride (**GH6**)

The reaction was performed according to the described general method. Ketone **8** (57 mg, 0.271 mmol, 1.0 equiv), dissolved in EtOH (2.7 mL), was reacted with AG·HCl (29 mg, 0.26 mmol, 0.98 equiv) at reflux for 7 h. After reaction completion, the mixture was taken to dryness obtaining a white crude. This residue was triturated with DCM and filtered, to obtain pure target **GH6** (57 mg, 0.188 mmol, 70% yield) as a white solid without further purification.

^1^H NMR (400 MHz, DMSO-*d*_6_) δ 11.20 (s, 1H), 8.72 (s, 1H), 8.20–8.12 (m, 2H), 7.80 (bs, 3H), 7.68 (d, *J* = 8.4 Hz, 2H), 7.51 (t, *J* = 7.8 Hz, 1H), 7.40 (t, *J* = 7.5 Hz, 1H), 2.44 (s, 3H).

^13^C-NMR (101 MHz, DMSO-*d*_6_) δ 156.7, 156.5, 156.4, 152.0, 132.7, 128.4, 127.1, 124.1, 124.0, 123.8, 121.8, 120.5, 112.3, 111.8, 15.4.

HRMS ESI (positive mode): [M+H]^+^ 267.1248.

#### 3.1.17. Synthesis of 2-(Dibenzo[*b,d*]thiophen-2-ylmethylene)hydrazine-1-carboximidamide (**GH7**)

The reaction was performed according to the general method. Dibenzo[*b,d*]thiophene-2-carbaldehyde (50 mg, 0.23 mmol, 1.00 equiv) was dissolved in EtOH (2.3 mL) and then reacted with AG·HCl (26 mg, 0.22 mmol, 0.98 equiv) at reflux for 7 h. After reaction completion, the mixture was taken to dryness and the resulting solid was triturated with diisopropyl ether (2 × 2.5 mL), obtaining pure target **GH7** as a white solid (61 mg, 0.2 mmol, 86% yield) without further purification.

^1^H NMR (400 MHz, DMSO-*d*_6_) δ 12.14 (s, 1H), 8.85 (d, *J* = 1.5 Hz, 1H), 8.49–8.41 (m, 1H), 8.35 (s, 1H), 8.11 (d, *J* = 8.4 Hz, 1H), 8.10–8.02 (m, 2H), 7.86 (s, 3H), 7.61–7.52 (m, 2H).

^13^C NMR (101 MHz, DMSO-*d*_6_) δ 155.4, 146.6, 140.7, 139.0, 135.4, 134.7, 130.4, 127.5, 125.9, 125.0, 123.3, 123.3, 122.3, 121.3.

HRMS ESI (positive mode): [M+H]^+^ 269.0862.

#### 3.1.18. Synthesis of 2-Acetyl Dibenzothiophene and 4-Acetyl Dibenzothiophene (**9** and **10**)

AlCl_3_ (578 mg, 4.34 mmol, 2 equiv) was suspended in dry DCM (18 mL) in a two-neck flask under an N_2_ atmosphere. AcCl (0.3 mL, 4.34 mmol, 2 equiv), and, after 10 min, dibenzothiophene (400 mg, 2.17 mmol, 1 equiv), were sequentially added to the mixture, and the reaction mixture was taken under stirring at r.t. overnight, monitored by TLC (eluent mixture: 5:5 *n*-Hex/DCM). After reaction completion, the mixture was quenched by addition of a few drops of 1 M HCl and the aqueous phase was extracted with DCM (3 × 25 mL). The combined organic phases were dried over anhydrous Na_2_SO_4_ and concentrated under reduced pressure. The crude was purified by direct flash column chromatography (eluent mixture: 1:1 *n*-Hex/DCM) to afford pure compound **9** (135 mg, 0.597 mmol, 27% yield) as a white solid. Compound **10** was also isolated as a by-product in 14% yield.

Compound **9**: ^1^H NMR (400 MHz, CDCl_3_) δ 8.74 (d, *J* = 1.1 Hz, 1H), 8.28–8.19 (m, 1H), 8.04 (dd, *J* = 8.4, 1.7 Hz, 1H), 7.90 (d, *J* = 9.0 Hz, 1H), 7.88–7.84 (m, 1H), 7.56–7.45 (m, 2H), 2.73 (s, 3H). The spectroscopic data are consistent with those reported in the literature [33].

Compound **10**: ^1^H NMR (400 MHz, CDCl_3_) δ 8.35 (d, *J* = 7.8 Hz, 1H), 8.16 (d, *J* = 2.3 Hz, 1H), 8.08 (d, *J* = 7.6 Hz, 1H), 7.93 (d, *J* = 6.9 Hz, 1H), 7.55 (t, *J* = 7.7 Hz, 1H), 7.52–7.43 (m, 2H), 2.77 (s, 3H). The spectroscopic data are consistent with those reported in the literature [34].

#### 3.1.19. Synthesis of 2-(1-(Dibenzo[*b*,*d*]thiophen-2-yl)ethylidene)hydrazine-1-carboximidamide Hydrochloride (**GH8**)

The reaction was performed according to the general method. Ketone **9** (25 mg, 0.110 mmol, 1 equiv), dissolved in EtOH (1.1 mL), was reacted with AG·HCl (11 mg, 0.105 mmol, 0.98 equiv) at reflux for 8 h. After reaction completion, the mixture was taken to dryness obtaining a white solid. This residue was triturated with DCM (2 × 2.5 mL) followed by filtration, to obtain pure target **GH8** (25 mg, 0.078 mmol, 73% yield) as a white solid.

^1^H NMR (400 MHz, DMSO-*d*_6_) δ 11.12 (s, 1H), 8.92 (d, *J* = 1.8 Hz, 1H), 8.62–8.52 (m, 1H), 8.20 (dd, *J* = 8.6, 1.8 Hz, 1H), 8.14–8.02 (m, 2H), 8.01–7.6 (bs), 7.62–7.51 (m, 2H), 2.50 (overlapped).

^13^C NMR (101 MHz, DMSO-*d*_6_) δ 156.5, 152.1, 140.5, 139.4, 135.7, 135.5, 134.2, 127.8, 126.0, 125.3, 123.6, 123.3, 123.0, 120.7, 15.3.

HRMS ESI (positive mode): [M+H]^+^ 283.1018.

#### 3.1.20. Synthesis of 2-(1-(Dibenzo[*b,d*]thiophen-4-yl)ethylidene)hydrazine-1-carboximidamide Hydrochloride (**GH9**)

The reaction was performed according to the general method. Ketone **10** (94 mg, 0.415 mmol, 1 equiv), dissolved in EtOH (4 mL), was reacted with AG·HCl (45 mg, 0.407 mmol, 0.98 equiv) at reflux for 8 h. After reaction completion, the mixture was taken to dryness obtaining a white solid. This residue was triturated with DCM (3 × 3.5 mL) followed by filtration, to obtain pure target **GH9** (103 mg, 0.323 mmol, 78% yield) as a white solid.

^1^H NMR (400 MHz, DMSO-*d*_6_) δ 11.78 (s, 1H), 8.52 (d, *J* = 7.9 Hz, 1H), 8.48–8.39 (m, 1H), 8.12–8.03 (m, 1H), 8.02 (d, *J* = 7.3 Hz, 1H), 7.68 (t, *J* = 7.8 Hz, 1H), 7.62–7.50 (m, 2H), 2.60 (s, 3H).

^13^C NMR (101 MHz, DMSO-*d*_6_) δ 156.6, 152.5, 140.4, 136.6, 135.1, 134.6, 131.3, 127.8, 125.4, 125.4, 123.9, 123.1, 122.5, 15.4.

HRMS ESI (positive mode): [M+H]^+^ 283.1037.

#### 3.1.21. Synthesis of 1,1′-(9,9-Dimethyl-9H-Fluorene-2,7-diyl)bis(ethan-1-one) (**11**)

2,7-Dibromo-9,9-dimethyl-9H-fluorene (83 mg, 0.24 mmol, 1 equiv) was dissolved in dry THF (2 mL) under nitrogen atmosphere. The resulting solution was cooled to −78 °C and *n*-BuLi (1.6 M in THF, 0.50 mL, 0.83 mmol, 3.5 equiv) was added dropwise under stirring. The resulting mixture was allowed to warm up to −30 °C within 1 h. Then, the mixture was cooled again to −78 °C and *N*-methoxy-*N*-methyl acetamide (0.10 mL, 0.95 mmol, 4 equiv) was added dropwise. The reaction was allowed to reach r.t. within 3 h and monitored by TLC (eluent mixture: 8:2 *n*-Hex/EtOAc, developed in 2,4-dinitrophenylhydrazine). After reaction completion, the mixture was treated with water (3 mL) and then taken to dryness. The resulting solid residue was dissolved in DCM (15 mL) and water (15 mL) was added. The aqueous phase was extracted with DCM (3 × 15 mL), the combined organic phases were dried over anhydrous Na_2_SO_4_, filtered, and concentrated under reduced pressure. The obtained crude was purified by direct flash column chromatography (eluent mixture: from 9:1 to 8:2 *n*-Hex/EtOAc) to afford pure intermediate **11** (39 mg, 0.14 mmol, 58% yield) as a yellow solid.

^1^H NMR (400 MHz, CDCl_3_) δ 8.08 (s, 2H), 8.00 (dd, *J* = 8.0, 1.6 Hz, 2H), 7.88–7.82 (m, 2H), 2.68 (s, 6H), 1.56 (s, 6H).

^13^C NMR (101 MHz, CDCl_3_) δ 198.0, 155.2, 142.7, 137.3, 128.4, 122.7, 121.1, 47.4, 28.0.

ESI positive mode: [M+H]^+^ 279.1316.

#### 3.1.22. Synthesis of 2,2′-((9,9-Dimethyl-9H-Fluorene-2,7-diyl)bis(ethan-1-yl-1-ylidene))bis(Hydrazine-1-carboximidamide) Dihydrochloride (**GH10**)

The reaction was performed according to the general method. Diketone **11** (10 mg, 0.036 mmol, 1 equiv) was dissolved in EtOH (0.4 mL) and then reacted with AG·HCl (7.5 mg, 0.068 mmol, 1.96 equiv) at reflux for 4 h. After the disappearance of the starting material, the reaction mixture was taken to dryness under reduced pressure obtaining a yellow solid residue (26 mg). The crude was triturated with Et_2_O (2 × 2.0 mL) to obtain pure target **GH10** (16 mg, 0.035 mmol, 96% yield) as a yellow solid.

^1^H NMR (400 MHz, DMSO-*d*_6_) δ 10.96 (s, 2H), 8.20 (d, *J* = 1.6 Hz, 2H), 7.94 (dd, *J* = 8.1, 1.6 Hz, 2H), 7.90 (d, *J* = 8.1 Hz, 2H), 7.50 (bs), 2.39 (s, 6H), 1.54 (s, 6H).

^13^C NMR (101 MHz, DMSO-*d*_6_) δ 155.8, 154.2, 152.2, 139.4, 136.3, 126.3, 121.2, 120.4, 46.9, 26.6, 14.7.

ESI positive mode: [M+H]^+^ 391.2381.

#### 3.1.23. Synthesis of 3,6-Diacetyl 9-H-Carbazole (**12**)

AlCl_3_ (640 mg, 4.78 mmol, 4 equiv) was suspended in dry DCM (6.3 mL) at 0 °C in a two-neck flask under a N_2_ atmosphere. AcCl (0.26 mL, 3.59 mmol, 3 equiv) and carbazole (200 mg, 1.19 mmol, 1 equiv) were sequentially added to the reaction mixture, which was taken under stirring at r.t. for 1 h, then refluxed for 3 h, monitoring by TLC (eluent mixture: 6:4 *n*-Hex/EtOAc). After reaction completion, the mixture was treated with few drops of a 4 M HCl aq. solution and the aqueous phase was extracted with DCM (3 × 25 mL). The combined organic phases were dried over anhydrous Na_2_SO_4_, filtered and concentrated under reduced pressure. The crude was purified via direct flash column chromatography (eluent mixture: 6:4 *n*-Hex/EtOAc) to afford 88 mg (0.350 mmol, 30% yield) of pure compound **12**.

^1^H NMR (400 MHz, DMSO-*d*_6_) δ 9.03 (s, 2H), 8.07 (d, *J* = 8.5 Hz, 2H), 7.61 (d, *J* = 8.6 Hz, 2H), 2.70 (s, 6H). The spectroscopic data are consistent with those reported in the literature [35].

#### 3.1.24. Synthesis of 2,2′-((9H-Carbazole-3,6-diyl)bis(ethan-1-yl-1-ylidene))bis(hydrazine-1-carboximidamide) Dihydrochloride (**GH11**)

The reaction was performed according to the general method A. Diketone **12** (19 mg, 0.084 mmol, 1 equiv) was dissolved in EtOH (0.9 mL) and then reacted with AG·HCl (18 mg, 0.165 mmol, 1.96 equiv) for 8 h. After reaction completion, the mixture was taken to dryness under reduced pressure giving a white solid. The residue was triturated with MeOH (3 × 2.5 mL) followed by filtration, to obtain pure target **GH11** (61 mg, 0.139 mmol, 70% yield) as a white solid.

^1^H NMR (400 MHz, DMSO-*d*_6_) δ 11.69 (s, 1H), 11.19–11.13 (m, 2H), 8.82 (s, 2H), 8.16 (dd, *J* = 8.7, 1.8 Hz, 2H), 7.80 (bs, 6H), 7.52 (d, *J* = 8.6 Hz, 2H), 2.49 (overlapped).

^13^C NMR (101 MHz, DMSO-*d*_6_) δ 156.4, 153.1, 141.4, 128.5, 125.4, 123.1, 120.2, 111.4, 15.3.

HRMS ESI (positive mode): [M+H]^+^ 364.2030.

#### 3.1.25. Synthesis of 2,8-Diformyl Dibenzofuran (**13**)

2,8-Dibromodibenzofuran (100 mg, 0.306 mmol, 1 equiv), dissolved in anhydrous Et_2_O (1.2 mL) under nitrogen atmosphere, was reacted with *n-*BuLi (0.57 mL, 0.920 mmol, 3 equiv), added at −78 °C, and the resulting reaction mixture was taken under stirring at r.t. After 1 h, DMF (0.10 mL, 1.22 mmol, 4 equiv) was added, and the resulting mixture was stirred at r.t. for 3 h monitoring by TLC (eluent mixture: 8:2 *n*-Hex/EtOAc, developed in 2,4-dinitrophenylhydrazine). After reaction completion, the mixture was quenched with water (3.0 mL). The aqueous phase was extracted with DCM (3 × 15 mL) and the combined organic phases were dried over anhydrous Na_2_SO_4_, filtered and concentrated under reduced pressure. The reaction crude was purified by direct flash column chromatography (eluent mixture: 8:2 *n*-Hex/EtOAc) to afford pure compound **13** (52 mg, 0.231 mmol, 76% yield) as a white solid.

^1^H NMR (400 MHz, CDCl_3_) δ 10.15 (s, 2H), 8.56 (d, *J* = 1.6 Hz, 2H), 8.09 (dd, *J* = 8.5, 1.7 Hz, 2H), 7.75 (d, *J* = 8.5 Hz, 2H).

^13^C NMR (101 MHz, CDCl_3_) δ 112.9, 123.6, 124.5, 130.1, 132.8, 160.5, 191.1.

ESI positive mode: [M+H]^+^ 225.0543.

#### 3.1.26. Synthesis of 2,2′-(Dibenzo[*b,d*]furan-2,8-diylbis(methaneylylidene))bis(hydrazine-1-carboximidamide) Dihydrochloride (**GH12**)

The reaction was performed according to the general method. Dialdehyde **13** (30 mg, 0.134 mmol, 1 equiv) was dissolved in EtOH (1.1 mL) and then reacted with AG·HCl (28 mg, 0.262 mmol, 1.96 equiv) at reflux for 8 h. After reaction completion, the reaction mixture was taken to dryness and the resulting residue was triturated with DCM (3 × 2.5 mL) and filtered, obtaining pure target **GH12** (39 mg, 0.095 mmol, 71% yield) as a white solid without further purification.

^1^H NMR (400 MHz, DMSO-*d*_6_) δ 12.14 (s, 2H), 8.67 (s, 2H), 8.37 (s, 2H), 8.13 (d, *J* = 8.6 Hz, 2H), 7.82 (d, *J* = 8.6 Hz, 2H).

^13^C NMR (101 MHz, DMSO-*d*_6_) δ 156.8, 155.2, 146.2, 129.1, 127.3, 123.5, 120.8, 112.1.

HRMS ESI (positive mode): [M+2H]^2+^ 169.0895; [M+H]^+^ 337.2002.

#### 3.1.27. Synthesis of 2,8-Diformyl Dibenzothiophene (**14**)

2,8-Dibromodibenzothiophene (50 mg, 0.146 mmol, 1 equiv), dissolved in dry Et_2_O (0.6 mL), was reacted with *n-*BuLi (0.26 mL, 0.438 mmol, 3 equiv) at −78 °C, and the reaction mixture was then left under stirring at r.t. After 1 h, dry DMF (0.043 mL, 0.584 mmol, 4 equiv) was added and the resulting mixture, stirred at r.t. for 3 h, was monitored by TLC (eluent mixture: 8:2 *n*-Hex/EtOAc, developed in 2,4-dinitrophenylhydrazine). After reaction completion, the reaction mixture was treated with water (3.0 mL) and the aqueous phase was extracted with DCM (3 × 15 mL). The combined organic phases were dried over anhydrous Na_2_SO_4_, filtered, and concentrated under reduced pressure. The reaction crude was purified via direct flash column chromatography (eluent mixture: 8:2 *n*-Hex/EtOAc) to afford pure compound **14** (25 mg, 0.104 mmol, 74% yield) as a white solid.

^1^H NMR (400 MHz, CDCl_3_) δ 10.18 (s, 2H), 8.71 (s, 2H), 8.07–7.99 (m, 4H). The spectroscopic data are consistent with those reported in the literature [36].

#### 3.1.28. Synthesis of 2,2′-(Dibenzo[*b,d*]thiophene-2,8-diylbis(methaneylylidene))bis(hydrazine-1-carboximidamide) Dihydrochloride (**GH13**)

The reaction was performed according to the general method. Dialdehyde **14** (25 mg, 0.104 mmol, 1 equiv) was dissolved in EtOH (1.1 mL) and then reacted with AG·HCl (22.5 mg, 0.204 mmol, 1.96 equiv) at reflux for 8 h. After reaction completion, the reaction mixture was taken to dryness obtaining a white solid. The resulting residue was triturated with DCM (2 × 3.0 mL) and then filtered, giving pure target **GH13** (43 mg, 0.113 mmol, 97% yield) as a white solid.

^1^H NMR (400 MHz, DMSO-*d*_6_) δ 12.21 (s, 2H), 9.02 (s, 2H), 8.36 (s, 2H), 8.14 (d, *J* = 8.4 Hz, 2H), 8.07 (d, *J* = 8.4 Hz, 2H), 7.87 (bs).

^13^C NMR (101 MHz, DMSO-*d*_6_) δ 155.9, 155.9, 146.9, 141.5, 135.7, 131.5, 131.1, 126.8, 123.9, 122.2.

HRMS ESI (positive mode): [M+2H]^2+^ 177.0810; [M+H]^+^ 353.1299.

#### 3.1.29. Synthesis of 2,8-Diacetyl 9-Methyl Carbazole (**15**)

3,6-Dibromo-9-methyl-9H-carbazole (100 mg, 0.294 mmol, 1 equiv), dissolved in dry THF (2.0 mL) and taken at −78 °C, was treated with *n*-BuLi (0.55 mL, 0.882 mmol, 3 equiv), added dropwise to the solution. The resulting mixture was allowed to reach r.t. within 1 h and then, after cooling again at −78 °C, was reacted with *N*-methoxy-*N*-methylacetamide (0.12 mL, 1.17 mmol, 4 equiv). The resulting mixture was allowed to reach r.t. within 3 h monitoring by TLC (eluent mixture: 6:4 *n*-Hex/EtOAc, developed in 2,4-dinitrophenylhydrazine). After reaction completion, the reaction mixture was treated with water (3.0 mL) and the aqueous phase was extracted with DCM (3 × 10 mL). The combined organic phases were dried over anhydrous Na_2_SO_4_, filtered, and concentrated under reduced pressure. The crude was purified by direct flash column chromatography (eluent mixture: 8:2 *n*-Hex/EtOAc) to afford pure compound **15** (38 mg, 0.143 mmol, 48% yield) as a white solid.

^1^H NMR (400 MHz, CDCl_3_) δ 8.76 (d, *J* = 1.8 Hz, 2H), 8.19 (d, *J* = 8.5 Hz, 2H), 7.44 (d, *J* = 8.6 Hz, 2H), 3.91 (s, 3H), 2.75 (s, 6H).

^13^C NMR (101 MHz, CDCl_3_) δ 197.5, 144.3, 129.7, 127.1, 122.8, 121.9, 108.7, 29.6, 26.7.

ESI positive mode: [M+H]^+^ 266.1172.

#### 3.1.30. Synthesis of 2,2′-((9-Methyl-9H-carbazole-3,6-diyl)bis(ethan-1-yl-1-ylidene))bis(hydrazine-1-carboximidamide) Dihydrochloride (**GH14**)

The reaction was performed according to the general method. Diketone **15** (30 mg, 0.113 mmol, 1 equiv) was dissolved in EtOH (1.3 mL) and then reacted with AG·HCl (24 mg, 0.221 mmol, 1.96 equiv) at reflux for 8 h. After reaction completion, the reaction mixture was taken to dryness, obtaining a white solid. The residue was triturated with DCM (3 × 2.0 mL) and then filtered to obtain pure target **GH14** (47 mg, 0.104 mmol, 92% yield) as a white solid.

^1^H NMR (400 MHz, DMSO-*d*_6_) δ 11.11 (s, 2H), 8.84 (s, 2H), 8.22 (d, *J* = 9.0 Hz, 2H), 7.77 (bs), 7.62 (d, *J* = 9.0 Hz, 2H), 3.94 (s, 3H), 2.50 (overlapped).

^13^C NMR (101 MHz, DMSO-*d*_6_) δ 156.4, 152.9, 142.4, 128.7, 125.5, 122.6, 120.2, 109.7, 29.9, 15.3.

HRMS ESI (positive mode): [M+2H]^2+^: 189.6216; [M+H]^+^: 378.2158.

#### 3.1.31. Synthesis of 2,8-Diacetyl Dibenzofuran (**16**)

2,8-Dibromodibenzofuran (100 mg, 0.306 mmol, 1 equiv), dissolved in dry THF (2.0 mL) at −78 °C under nitrogen atmosphere, was reacted with *n*-BuLi (0.57 mL, 0.920 mmol, 3 equiv), added dropwise to the solution. The resulting mixture was allowed to reach r.t. within 1 h and then cooled again at −78 °C for the subsequent addition of *N*-methoxy-*N*-methyl acetamide (0.13 mL, 1.22 mmol, 4 equiv). The resulting reaction mixture was allowed to reach r.t. within 3 h monitoring by TLC (eluent mixture: 7:3 *n*-Hex/EtOAc, developed in 2,4-dinitrophenylhydrazine). After reaction completion, the reaction mixture was treated with water (3.0 mL) and the aqueous phase was extracted with DCM (3 × 10 mL). The combined organic phases were dried over anhydrous Na_2_SO_4_, filtered and concentrated under reduced pressure. The crude was purified by direct flash column chromatography (eluent mixture: 8:2 *n*-Hex/EtOAc) to afford pure compound **16** (28 mg, 0.110 mmol, 37% yield) as a white solid.

^1^H NMR (400 MHz, CDCl_3_) δ 8.65 (s, 2H), 8.17 (d, *J* = 8.6 Hz, 2H), 7.65 (d, *J* = 8.6 Hz, 2H), 2.74 (s, 6H).

^13^C NMR (101 MHz, CDCl_3_) δ 197.4, 159.6, 133.6, 129.2, 124.6, 122.4, 112.5, 27.2.

ESI positive mode: [M+H]^+^ 253.0866.

#### 3.1.32. Synthesis of 2,2′-(Dibenzo[*b,d*]furan-2,8-diylbis(ethan-1-yl-1-ylidene))bis(hydrazine-1-carboximidamide) Dihydrochloride (**GH15**)

The reaction was performed according to the general method. Diketone **16** (15 mg, 0.059 mmol, 1 equiv), dissolved in EtOH (0.6 mL), was reacted with AG·HCl (12 mg, 0.116 mmol, 1.96 equiv) at reflux for 8 h. After reaction completion, the reaction mixture was taken to dryness obtaining a white solid. This residue was triturated with DCM (2 × 2.0 mL) and filtered, giving pure target **GH15** (24 mg, 0.054 mmol, 93% yield) as a white solid without further purification.

^1^H NMR (400 MHz, DMSO-*d*_6_) δ 11.34 (s, 2H), 8.88 (d, *J* = 2.0 Hz, 2H), 8.24 (dd, *J* = 8.8, 2.0 Hz, 2H), 7.75 (d, *J* = 8.8 Hz, 2H), 2.48 (overlapped).

^13^C NMR (101 MHz, DMSO-*d*_6_) δ 156.8, 156.1, 151.4, 132.5, 126.8, 123.7, 120.3, 111.5, 14.9.

HRMS ESI (positive mode): [M+2H]^2+^ 183.2585; [M+H]^+^ 365.1838.

#### 3.1.33. Synthesis of 2,8-Diacetyl Dibenzothiophene (**17**)

2,8-Dibromodibenzothiophene (150 mg, 0.438 mmol, 1 equiv), dissolved in dry THF (1.9 mL) at −78 °C under nitrogen atmosphere, was reacted with *n*-BuLi (0.82 mL, 1.31 mmol, 3 equiv), added dropwise to the solution. The resulting mixture was allowed to reach r.t. within 1 h and then cooled again to −78 °C for the subsequent addition of *N*-methoxy-*N*-methylacetamide (0.18 mL, 1.75 mmol, 4 equiv). The resulting mixture was allowed to reach r.t. within 3 h, monitoring by TLC (eluent mixture: 7:3 *n*-Hex/EtOAc, developed in 2,4-dinitrophenylhydrazine). After reaction completion, the mixture was treated with water (5.0 mL) and the aqueous phase was extracted with DCM (3 × 10 mL). The combined organic phases were dried over anhydrous Na_2_SO_4_, filtered, and concentrated under reduced pressure. The reaction crude was purified by direct flash column chromatography (eluent mixture: 8:2 *n*-Hex/EtOAc) to afford (49 mg, 0.183 mmol, 42% yield) of pure compound **17** as a white solid.

^1^H NMR (400 MHz, CDCl_3_) δ 8.77 (d, *J* = 1.5 Hz, 2H), 8.07 (dd, *J* = 8.4, 1.7 Hz, 2H), 7.90 (d, *J* = 8.4 Hz, 2H), 2.74 (s, 6H).

^13^C NMR (101 MHz, CDCl_3_) δ 197.4, 144.7, 135.3, 134.2, 126.9, 122.9, 122.0, 26.8.

ESI positive mode: [M+H]^+^ 269.0628.

#### 3.1.34. Synthesis of 2,2′-(Dibenzo[*b,d*]thiophene-2,8-diylbis(ethan-1-yl-1-ylidene))bis(hydrazine-1-carboximidamide) Dihydrochloride (**GH16**)

The reaction was performed according to the general method. Diketone **17** (14 mg, 0.052 mmol, 1 equiv) was dissolved in EtOH (0.5 mL) and then reacted with AG·HCl (11.3 mg, 0.102 mmol, 1.96 equiv) for 8 h. After reaction completion, the reaction mixture was taken to dryness, thus obtaining a white solid. This residue was triturated with DCM (2 × 2.0 mL) and filtered, to obtain pure target **GH16** (17 mg, 0.037 mmol, 72% yield) as a white solid without further purification.

^1^H NMR (400 MHz, DMSO-*d*_6_) δ 8.78 (d, *J* = 1.8 Hz, 2H), 8.06 (dd, *J* = 8.5, 1.7 Hz, 2H), 7.91 (d, *J* = 8.5 Hz, 2H), 2.40 (s, 6H).

^13^C NMR (101 MHz, DMSO-*d*_6_) δ 160.2, 147.8, 138.2, 137.7, 135.8, 125.2, 122.7, 119.4, 14.2.

HRMS ESI (positive mode): [M+2H]^2+^ 191.2115; [M+H]^+^ 381.1651.

### 3.2. G4-CPG Assay

Stock solutions (4 mM) of each guanyl hydrazone were obtained by dissolving a known amount of the sample in pure DMSO. From the stock solution a measured volume was taken, so to obtain a 60 μM ligand solution in 50 mM KCl, 10% DMSO, 10% EtOH aq. solution. For the G4-CPG assays, the following general procedure was adopted: weighed amounts of the nude CPG and G-quadruplex-/duplex-functionalized CPG supports (ca. 8 mg) [19] were left in contact with 300 μL of the ligand solution in a polypropylene column (4 mL volume, Alltech Nicholasville, KY, USA) equipped with a polytetrafluoroethylene frit (10 μm porosity), a stopcock and a cap. After incubation on a vibrating shaker for 4 min, each support was washed with defined volumes of the washing solution (50 mM KCl, 10% DMSO, 10% EtOH aq. solution), followed by the releasing solutions (2.5 M CaCl_2_, 15% DMSO aq. solution and pure DMSO), and all the eluted fractions were separately analyzed by UV measurements. After treatment with the releasing solution, inducing G-quadruplexes and duplex structures denaturation, the supports were suspended in the washing solution and then subjected to annealing by taking them at 75 °C for 5 min and then slowly cooling to r.t. This thermal treatment allowed the correct folding of the DNA structures and the G-quadruplex-/duplex-functionalized CPG supports to be used for multiple experiments.

The UV measurements were carried out on a JASCO V-550 UV-vis spectrophotometer (Tokyo, Japan), using a quartz cuvette with a path length of 1 cm. The UV quantification of the tested compounds was determined by measuring the absorbance relative to the λ_max_ characteristic of each compound and referring it to the corresponding calibration curves. The errors associated with the % determination of bound ligand are within ±2%.

### 3.3. Circular Dichroism

CD spectra were registered on a Jasco J-715 spectropolarimeter (Tokyo, Japan) equipped with a Peltier-type temperature control system (model PTC-348WI, Tokyo, Japan), using a quartz cuvette with a path length of 1 cm. The spectra were recorded at 20 °C in the range 230–600 nm, 200 nm/min scanning speed and 2.0 nm bandwidth and were corrected by subtraction of the background scan with buffer. All the spectra were averaged over three scans. The oligonucleotides d[(TTAGGG)_4_TT] (tel26), d(TGAGGGTGGGTAGGGTGGGTAA) (pu22), and d(CGCGAATTCGCG) (ds12) were purchased from Biomers (Ulm, Germany) as HPLC-purified compounds with a > 99% purity. The oligonucleotides tel26 and ds12 were dissolved in a 20 mM KCl, 5 mM potassium phosphate buffer (pH 7); in turn, pu22 was dissolved in a 10 mM Tris-HCl buffer (pH 7). In all cases, 2 μM oligonucleotide solutions were prepared, which were then annealed by heating at 95 °C for 5 min, followed by slow cooling to r.t. CD titrations were obtained by adding increasing amounts of each compound (up to 2 molar equiv, corresponding to a 4 μM compound solution) to the oligonucleotide solutions. For the CD melting experiments, ellipticity was recorded at 290 nm for tel26, 263 nm for pu22 and 253 nm for ds12, on increasing the temperature at a scan rate of 1 °C/min in the range of 10–95 °C.

### 3.4. Fluorescence Spectroscopy

Fluorescence spectra were registered on a HORIBA JobinYvon Inc. FluoroMax^®^-4 spectrofluorometer (Kyoto, Japan) equipped with Peltier F-3004 Sample Heater/Cooler Peltier Thermocouple Drive (Kyoto, Japan) at 20 °C, using a quartz cuvette with a 1 cm path length. Excitation wavelengths used for the fluorescence titrations were as follows: 305 nm for **GH1**, 273 nm for **GH2**, 383 nm for **GH3**, 369 nm for **GH4**, 287 nm for **GH5**, 285 nm for **GH6**, 294 nm for **GH7**, 292 nm for **GH8**, 291 nm for **GH9,** and 286 nm for **GH15**. The spectra were recorded in the range 325–550 nm for **GH1**, 300–500 nm for **GH2**, 400–600 nm **GH3** and **GH4**, 325–525 nm for **GH5**-**GH9** and **GH15**.

Titrations were carried out using a fixed concentration (2.0 μM) of each fluorescent ligand. Increasing amounts of tel26, pu22, or ds12 (up to 10 μM), taken from 120 μM annealed stock solutions of each DNA sample dissolved in a 20 mM KCl, 5 mM potassium phosphate buffer (pH 7), were then added to each ligand solution. The fraction of bound ligand was calculated from the fluorescence intensity at 359 nm for **GH2**, 426 nm for **GH4**, 361 nm for **GH5** and 414 nm for **GH7**. The fraction of bound ligand was determined using the equation:

$$\alpha \;=\frac{Y-Y_{0}}{Y_{b}\;-Y_{0}}$$

where Y, Y_0_, and Y_b_ are the values of fluorescence emission intensity at the maximum at each titrant concentration, at the initial and final state of the titration, respectively. These experimental points were fitted using the independent and equivalent-sites model as provided by the Origin 9.0 program [23].

The equation of the independent and equivalent-sites model is as follows:

$$\alpha =\left(\frac{1}{2{\left[L\right]}_{0}}\right)\left\{\left({\left[L\right]}_{0}+n\left[DNA\right]+\frac{1}{K_{b}}\right)-\sqrt{{\left({\left[L\right]}_{0}+n\left[DNA\right]+\frac{1}{K_{b}}\right)}^{2}-4{\left[L\right]}_{0}n\left[DNA\right]}\right\}$$

where α is the mole fraction of ligand in the bound form, [L]_0_ is the total ligand concentration, [DNA] is the added DNA concentration, n is the number of the equivalent and independent sites on the DNA structure, and K_b_ is the binding constant.

### 3.5. Docking Studies

The oligonucleotides tel26 and pu22 were prepared using as starting models the NMR deposited G-quadruplex structure of the complexes tel26/Auoxo6 (PDB 5MVB) [24] and pu22/Quindoline (PDB 2L7V) [25], respectively, from which the bound ligands were removed. The bimolecular duplex-forming oligonucleotide ds12 was prepared starting from the NMR deposited duplex structure of ds12 (PDB 1NAJ) [27], in which an intercalative binding site between C1:G24 and G2:C23 base pairs was created, using the crystallographic bimolecular duplex structure complexed with the intercalator ligand ellipticine as a template (PDB 1Z3F) [26].

Molecular docking calculations were carried out using AutoDock Vina v1.2.7 with the aid of its graphical user interface AutoDockTools (La Jolla, CA, USA. v1.5.7) [37,38]. The ligands and DNA targets were prepared using AutoDockTools and UCSF Chimera by assigning bond orders, adding hydrogen atoms and generating the appropriate protonation states. The ligands and targets were then converted to proper Autodock PDBQT file formats and the Gaisteiger charges were assigned. The 3D grid box dimensions were defined so to include the whole DNA macromolecules. The docking area was centered on the DNA center of mass and grid boxes of 110 Å × 100 Å × 80 Å, 100 Å × 90 Å × 60 Å and 60 Å × 120 Å × 60 Å for tel26, pu22, and ds12, respectively, with a 0.375 Å spacing. A total of 20 docking poses were obtained by using as docking parameters: seed = random, exhaustiveness = 24 for each DNA/ligand system. Docking poses were clustered on the basis of their root-mean square deviation and ranked based on their binding energy. Interactions were evaluated by LigPlot+. [39] Molecular modeling figures were drawn by UCSF Chimera (San Francisco, CA, USA) and LigPlot^+^ (Cambirdge, United Kingdom).

### 3.6. Biological Assays

#### 3.6.1. Cell Cultures and Cytotoxicity Assays

Human MCF7 breast cancer cells were obtained from the American Type Culture Collection (ATCC, Manassas, VA, USA) and cultured in high-glucose Dulbecco’s modified Eagle’s medium (DMEM) supplemented with 10% fetal bovine serum (FBS), 1% antibiotics (Pen/strep), and 1% L-glutamine at 37 °C in a humidified atmosphere containing 5% CO_2_ as previously described [40]. For the MTT assays, cells were seeded into 96-well plates at a density of 5 × 10^3^ cells/well. After 24 h, the cell supernatant was replaced with fresh medium containing increasing concentrations of the tested guanyl hydrazones (up to 50 µM). Cells were then cultured for different time intervals (up to 72 h). After incubation, MTT assays were performed as previously reported [40]. Briefly, cell culture supernatants were removed and cells were incubated with 0.5 mg/mL MTT reagent dissolved in DMEM medium without red phenol (100 μL/well). After an incubation of 4 h at 37 °C, the resulting insoluble formazan salts were solubilized upon addition of anhydrous isopropanol containing 0.01 M HCl and quantified by measuring their absorbance at 570 nm on an automated plate reader spectrophotometer (Benchmark Plus Microplate Spectrophotometer, Biorad, Hercules, CA, USA). Cell viability was expressed as the mean percentage compared to untreated control cells.

#### 3.6.2. Statistical Analyses

Statistical analyses were performed by using a Student’s *t*-Test. Significant differences were indicated as * *p* ≤ 0.05, ** *p* ≤ 0.01 and *** *p* ≤ 0.001 for treated vs. control samples.

## 4. Conclusions

Cancer treatments are typically associated with second-order effects due to poor selectivity of action of the common anticancer drugs. In this context, G-quadruplex structures and selective ligands thereof emerged as appealing targets and potential drugs, respectively, to solve this crucial issue in anticancer therapies. In this frame, we here designed, synthesized, and evaluated a novel library of guanyl hydrazone-derivatized tricyclic compounds as potential selective ligands of telomeric and oncogenic G-quadruplexes. A model duplex was used as selectivity control to evaluate potential off-targets effects.

In detail, the library included 9H-fluorene (**GH1** and **GH10**), 2,3,4,9-tetrahydro-1H-pyrido [3,4-*b*]indole (**GH2**), 9H-pyrido [3,4-*b*]indole (**GH3** and **GH4**), 9H-carbazole (**GH11** and **GH14**), dibenzo[*b*,*d*]furan (**GH5**, **GH6**, **GH12** and **GH15**), and dibenzo[*b*,*d*]thiophene (**GH7**, **GH8**, **GH9**, **GH13** and **GH16**) derivatives.

First, the G4-CPG assay was exploited to assess the capacity of the examined guanyl hydrazones to interact with G-quadruplexes, as well as their G-quadruplex vs. duplex selectivity. In contrast to the generally poor selectivity exhibited by ligands endowed with two guanyl hydrazone substituents, all the monosubstituted compounds (**GH1**–**GH9**) showed a remarkable ability to interact with the telomeric and oncogenic G-quadruplexes, as well as a good ability to discriminate between the G-quadruplex models and the control duplex. Among the monosubstituted compounds, higher G-quadruplex over duplex selectivity was found for the pyrido [3,4-*b*]indole and dibenzofuran derivatives (**GH2**, **GH5**, **GH6**) compared to the 9H-fluorene, 9H-carbazole, and dibenzothiophene-based compounds.

Considering the importance of developing novel generation anticancer agents endowed with a good affinity for the targets of choice, but also showing reduced off-targets effects compared to the currently used chemotherapeutics, the most selective ligands within the investigated series, i.e., all the monosubstituted derivatives, were considered as the privileged compounds to be advanced to more in-depth biophysical studies. Thus, these ligands, and in parallel **GH15** included as a reference for the disubstituted compounds, were tested for their ability to interact with and/or thermally stabilize the target G-quadruplex structures by CD analysis, always using a model duplex as control. All compounds produced significant conformational changes as well as generally strong stabilizing effects when mixed with both telomeric and oncogenic G-quadruplexes. In contrast, no detectable effect was observed on the control duplex for most of the investigated ligands, apart from slight conformational changes in the case of **GH7**, **GH9,** and particularly the control ligand **GH15**, with the strongest thermal stabilization produced by **GH15** followed by **GH1**, **GH4**, and **GH6**. Taken together, the CD data indicated **GH3**, **GH5**, **GH7**, and **GH9** as the most selective ligands in the series in stabilizing the G-quadruplexes compared to the duplex.

In fluorescence titration experiments, almost all ligands showed significant changes in their fluorescence intensities when treated with both tel26 and pu22 G-quadruplexes, whereas null or very limited effects were detected upon titration with ds12 duplex, confirming the higher affinity of the monosubstituted ligands for G-quadruplexes than duplex DNA. However, the highest selectivity regarding the differences in fluorescence observed upon titration with G-quadruplexes over duplex were produced in the case of **GH3**, **GH4**, **GH5**, **GH6**, **GH9**, and **GH15**. A quantitative analysis of the acquired fluorescence data could be performed for **GH2**, **GH4**, **GH5**, and **GH7**, proving their high affinity for G-quadruplexes.

Molecular docking studies carried out on **GH6** and **GH15** as representative examples of mono- and bis-substituted ligands provided useful structural hints to better intepret their binding. This analysis suggested that the first one targeted the hybrid telomeric G-quadruplex model with a coplanar arrangement of both its core and guanyl hydrazone moieties stacking on the G-quartets, while in the interaction with the parallel oncogenic G-quadruplex model only the ligand cores were located on top of the G-quartets, and the guanyl hydrazone moieties of both ligands pointed towards the grooves/loops. Interestingly, in the case of ds12 duplex, the bis-substituted ligand **GH15** targeted the minor groove of the duplex in close proximity to the duplex intercalation site, which would imply a stronger binding to the model duplex than observed with **GH6**. This was confirmed also by analysis of the obtained binding energies, which evidenced the higher affinity of the bis-substituted **GH15** compared to mono-substituted **GH6** towards G-quadruplexes, but also for the control duplex, thus well explaining the lower G-quadruplex vs. duplex selectivity of the bis-substituted ligand **GH15** with respect to the corresponding mono-substituted **GH6**.

Finally, preliminary biological assays indicated that the mono-guanyl hydrazone-derivatized tricyclic compounds **GH3**, **GH4**, **GH5**, **GH6**, **GH7**, and **GH9** showed the highest cytotoxicity (IC_50_ values in the low micromolar range) against MCF-7 cancer cells in the investigated series, with **GH4** and **GH9** showing also the highest selectivity with respect to normal cells. In turn, the bis-guanyl hydrazone-derivatized **GH15** showed the lowest anticancer activity among all the tested compounds along with reduced selectivity.

In conclusion, mono-guanyl hydrazone-derivatized tricyclic compounds emerged as promising G-quadruplex ligands, generally better performing than their bis-derivatized congeners. This study highlighted the potential of prioritizing synthetic modularity and focusing on essential structural components in order to develop structurally simple yet selective G-quadruplex-targeting agents.

## Figures and Tables

**Table 1:** Summary of the binding data obtained for the investigated compounds, as determined by the G4-CPG assay. The amounts of bound ligand are calculated as a difference between the initially loaded amount of ligand and the unbound ligand, recovered by the washing solution (50 mM KCl, 10% DMSO, 10% EtOH), and are expressed as percentage of the quantity initially loaded on each support. The errors associated with the reported percentages are typically within ±2%. The selectivity index is the ratio between the percentages of ligand bound to the indicated supports.

Compound	Bound Ligand				Selectivity Index		
	Nude-CPG	CPG-tel26	CPG-c-myc	CPG-ds27	CPG-tel26/ CPG-ds27	CPG-c-myc/ CPG-ds27	CPG-tel26/ CPG-c-myc
**GH1**	0%	86%	71%	38%	2.3	1.9	1.2
**GH2**	0%	88%	72%	28%	3.1	2.6	1.2
**GH3**	0%	87%	82%	37%	2.3	2.2	1.1
**GH4**	0%	73%	70%	30%	2.4	2.3	1.0
**GH5**	0%	65%	86%	32%	2.0	2.7	0.75
**GH6**	0%	70%	68%	21%	3.3	3.2	1.0
**GH7**	0%	92%	88%	63%	1.5	1.4	1.1
**GH8**	0%	70%	94%	52%	1.3	1.8	0.74
**GH9**	0%	60%	77%	56%	1.1	1.4	0.78
**GH10**	0%	71%	88%	65%	1.1	1.3	0.81
**GH11**	0%	72%	85%	75%	0.96	1.1	0.85
**GH12**	0%	76%	100%	91%	0.83	1.1	0.76
**GH13**	0%	85%	81%	83%	1.0	0.97	1.0
**GH14**	0%	74%	85%	97%	0.76	0.88	0.87
**GH15**	0%	74%	88%	81%	0.91	1.1	0.84
**GH16**	0%	82%	83%	77%	1.1	1.1	0.99

**Table 2:** Melting temperature (T_m_) values of tel26, pu22, and ds12 in the presence of the investigated compounds (2 molar equiv) as measured by CD melting experiments in 20 mM KCl, 5 mM potassium phosphate buffer (pH 7) for tel26 and ds12, or 10 mM Tris-HCl buffer (pH 7) for pu22. The error associated with T_m_ values is ±1 °C. ΔT_m_ = T_m_ (DNA/ligand, 1:2) − T_m_ (free DNA).

Compound	tel26		pu22		ds12	
	T_m_ (°C)	ΔT_m_ (°C)	T_m_ (°C)	ΔT_m_ (°C)	T_m_ (°C)	ΔT_m_ (°C)
**GH1**	47	+4	35	+5	67	+2
**GH2**	44	+1	32	+2	66	+1
**GH3**	49	+6	46	+16	67	+2
**GH4**	46	+3	40	+10	66	+1
**GH5**	48	+5	39	+9	66	+1
**GH6**	48	+5	39	+9	67	+2
**GH7**	51	+8	43	+13	66	+1
**GH8**	47	+4	35	+5	66	+1
**GH9**	51	+8	40	+10	66	+1
**GH15**	58	+15	72	+42	71	+6

**Table 3:** Binding constant (K_b_) and stoichiometry (n) values for tel26, pu22, and ds12 in the presence of **GH2**, **GH4**, **GH5**, and **GH7** measured by fluorescence experiments in 20 mM KCl, 5 mM potassium phosphate buffer (pH 7).

Compound	tel26		pu22		ds12	
	n	K_b_	n	K_b_	n	K_b_
**GH2**	2	(7.6 ± 1.0) × 10^5^	2	(2.5 ± 1.0) × 10^6^	2	(6.3 ± 0.7) × 10^5^
**GH4**	2	(2.3 ± 0.4) × 10^6^	2	(1.4 ± 0.3) × 10^6^	ND	ND
**GH5**	2	(1.5 ± 0.2) × 10^6^	ND	ND	2	(2.0 ± 0.5) × 10^6^
**GH7**	3	(2.4 ± 0.7) × 10^6^	ND	ND	2	(3.7 ± 0.7) × 10^6^

ND = not defined.

**Table 4:** Binding energies calculated by AutoDock Vina for **GH1**, **GH4**, **GH6**, **GH8,** and **GH15** docked to tel26 G-quadruplex (PDB 5MVB), pu22 G-quadruplex (PDB 2L7V), and ds12 duplex (PDB 1NAJ).

Compound	Binding Energy (kcal/mol)		
	tel26	pu22	ds12
**GH1**	−8.2	−7.3	−7.7
**GH4**	−8.1	−7.1	−7.3
**GH6**	−8.2	−6.9	−7.3
**GH8**	−8.3	−7.3	−8.0
**GH15**	−10.2	−8.1	−8.7

**Table 5:** IC_50_ values were established by testing increasing concentrations of each compound in the 0–50 µM range for 72 h on MCF7 cancer cells, as well as HaCaT normal cells by MTT assays.

Compound	IC_50_ (µM)		Cancer vs. Normal Cells Selectivity
	MCF7	HaCaT	HaCaT/MCF7
**GH1**	1.8	1.5	0.83
**GH2**	35	33	0.94
**GH3**	4.0	5.0	1.2
**GH4**	4.0	7.2	1.8
**GH5**	4.0	3.6	0.90
**GH6**	4.0	3.5	0.88
**GH7**	1.8	2.4	1.3
**GH8**	5.9	7.8	1.3
**GH9**	3.2	4.5	1.4
**GH15**	6.0	6.7	1.1

## Data Availability

Original data can be reasonably requested from the corresponding authors.

## Supplementary Materials

The following supporting information can be downloaded at: https://www.mdpi.com/article/10.3390/ijms27125282/s1.

## Abbreviations

AG·HCl	aminoguanidine hydrochloride
AcCl	acetyl chloride
AcOH	acetic acid
ACN	acetonitrile
CD	circular dichroism
DCM	dichloromethane
DMF	dimethylformamide
DMP	Dess–Martin periodinane
DMSO	dimethyl sulfoxide
Et_2_O	diethyl ether
EtOAc	ethyl acetate
EtOH	ethanol
G4-CPG	G-quadruplex on Controlled Pore Glass affinity chromatography assay
IC_50_	half-maximal inhibitory concentration
K_b_	binding constant
MeOH	methanol
MTT	3-(4,5-dimethylthiazol-2-yl)-2,5-diphenyltetrazolium bromide
*n*-BuLi	*n*-butyllithium
TFA	trifluoroacetic acid
THF	tetrahydrofuran
TMS	tetramethylsilane
T_m_	melting temperature
Tris-HCl	tris(hydroxymethyl)aminomethane hydrochloride
