# Supplementary material for: Mono- vs. Bis-Guanyl Hydrazone-Decorated Tricyclic Scaffolds: Effects on G-Quadruplex Binding and Selectivity
Source: Int J Mol Sci. 2026 Jun 10;27(12):5282. doi: 10.3390/ijms27125282 (PMC13299455; doi:10.3390/ijms27125282)

SUPPLEMENTARY MATERIAL

to the manuscript:

Mono- vs. Bis-Guanyl Hydrazone-Decorated Tricyclic Scaffolds: Effects on G-Quadruplex Binding and Selectivity

Chiara Platella [1,†], Alice Maiocchi [2,†], Giovanni Cipolla [2], Rosa Gaglione [1], Angela Arciello [1], Pierfausto Seneci [2], Domenica Musumeci [1,3], Alessandra Silvani [2], Clelia Giannini [2,*] and Daniela Montesarchio [1,*]

[1] Department of Chemical Sciences, University of Naples Federico II, Via Cintia 21, 80126 Naples, Italy; chiara.platella@unina.it (C.P.); rosa.gaglione@unina.it (R.G.); angela.arciello@unina.it (A.A.); domenica.musumeci@unina.it (D.M.)
[2] Department of Chemistry, University of Milan, Via Golgi 19, 20133 Milan, Italy; alice.maiocchi@unimi.it (A.M.); giovanni.cipolla@studenti.unimi.it (G.C.); pierfausto.seneci@unimi.it (P.S.); alessandra.silvani@unimi.it (A.S.)
[3] Institute of Biostructure and Bioimaging (IBB), National Research Council (CNR), 80145 Naples, Italy
* Correspondence: clelia.giannini@unimi.it (C.G.); daniela.montesarchio@unina.it (D.M.)
† These authors contributed equally to this work.

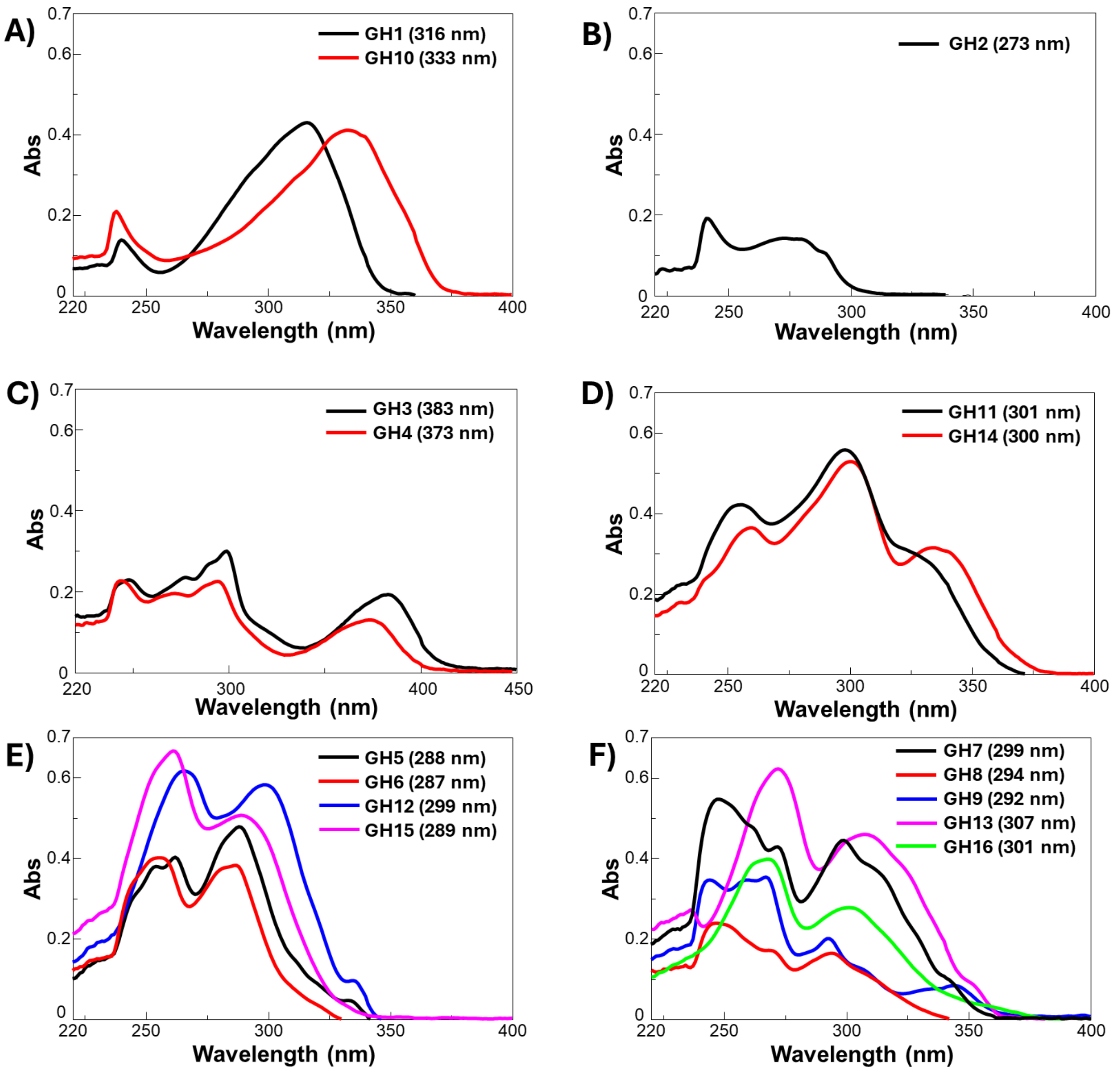

Figure S1. UV spectra of 15 μM solutions of A) 9H-fluorene (**GH1**, **GH10**), B) 2,3,4,9-tetrahydro-1H-pyrido[3,4-*b*]indole (**GH2**), C) 9H-pyrido[3,4-*b*]indole (**GH3**, **GH4**), D) 9H-carbazole (**GH11**, **GH14**), E) dibenzo[*b,d*]furan (**GH5**, **GH6**, **GH12**, **GH15**) and F) dibenzo[*b,d*]thiophene derivatives (**GH7**, **GH8**, **GH9**, **GH13**, **GH16**) in 50 mM KCl, 10% DMSO, 10% CH_3CH_2OH. The λ_{max} used to calculate the percentage of bound ligand are indicated in brackets for each guanyl hydrazone.

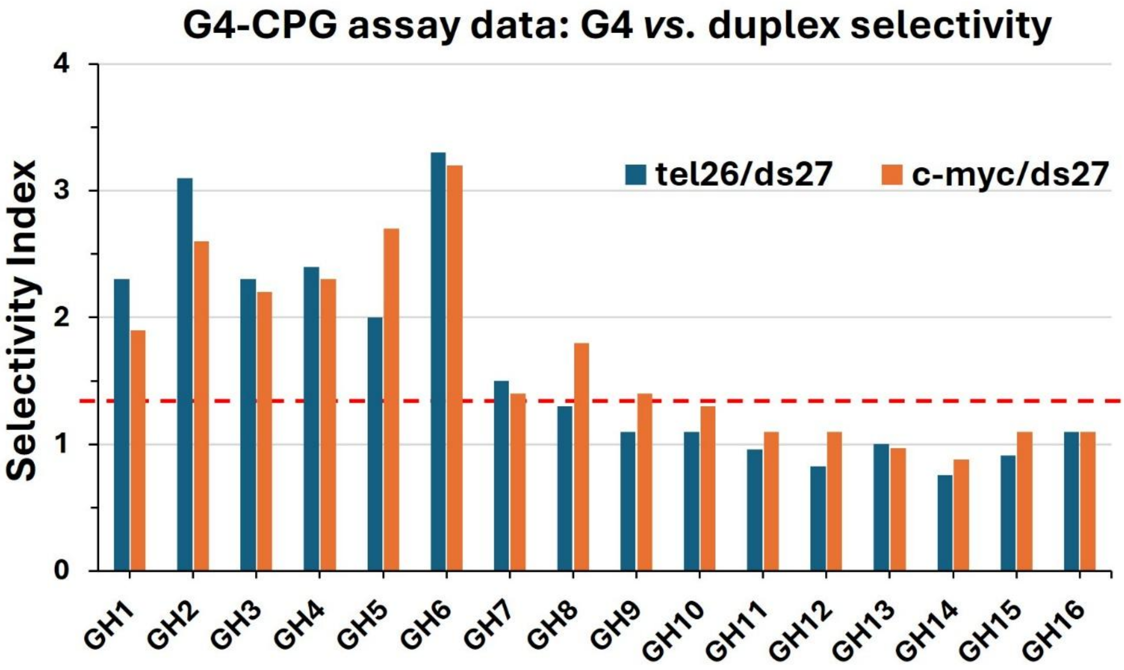

Figure S2. Selectivity index values (G4 *vs.* duplex) of the compounds **GH1-GH16** as determined by the G4-CPG assay and reported in Table 1 of the main text, section: **Screening of the guanyl hydrazones library by the G4-CPG assay**.

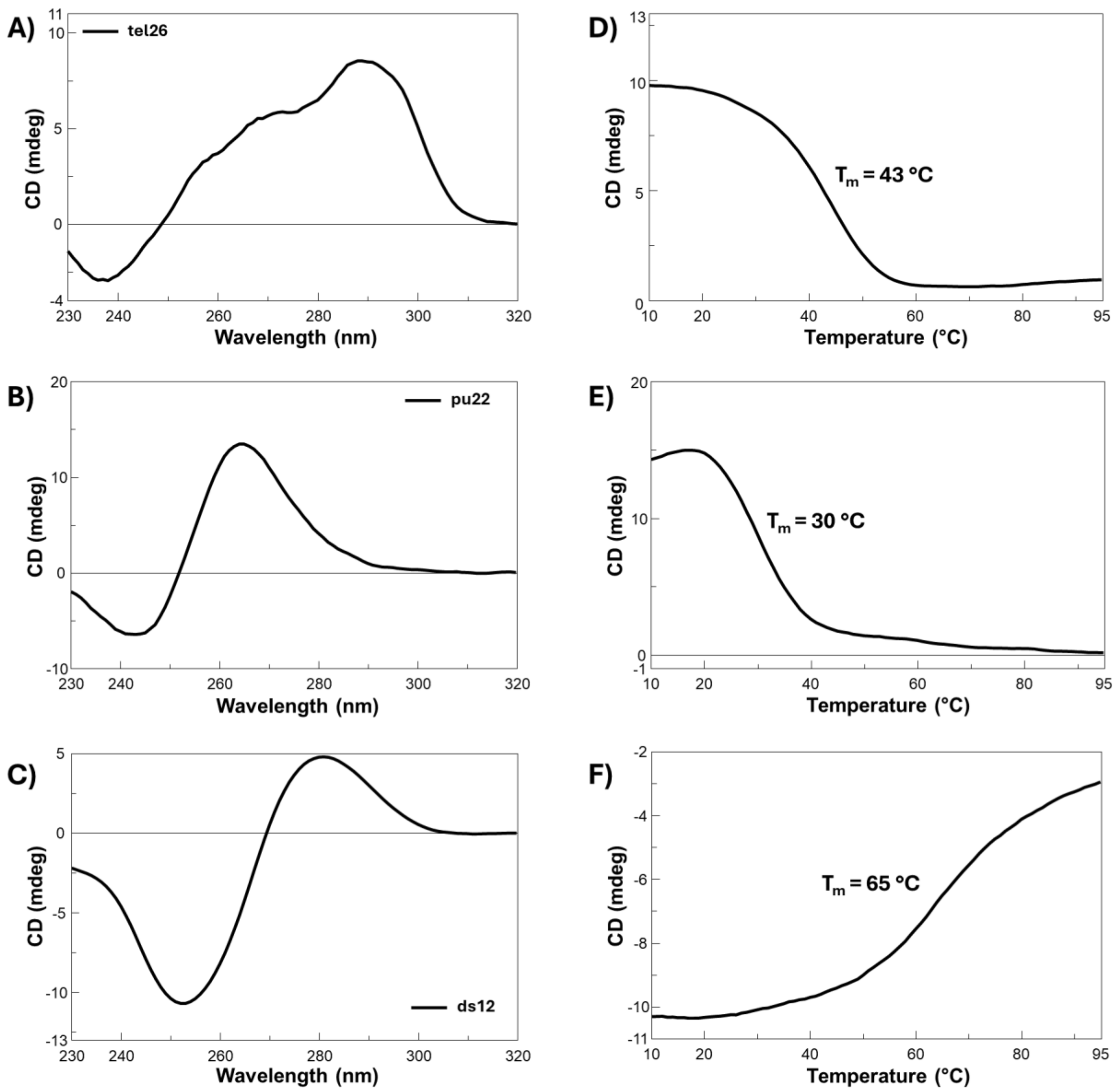

Figure S3. Left panels: CD spectra of 2 μM solutions of A) tel26, B) pu22 and C) ds12 in 20 mM KCl, 5 mM potassium phosphate buffer (pH 7) for tel26 and ds12 or in 10 mM Tris-HCl buffer (pH 7) for pu22. Right panels: CD melting curves for D) tel26, E) pu22 and F) ds12 in 20 mM KCl, 5 mM potassium phosphate buffer (pH 7) for tel26 and ds12, recorded at 290 and 253 nm respectively, and in 10 mM Tris-HCl buffer (pH 7) for pu22, recorded at 263 nm.

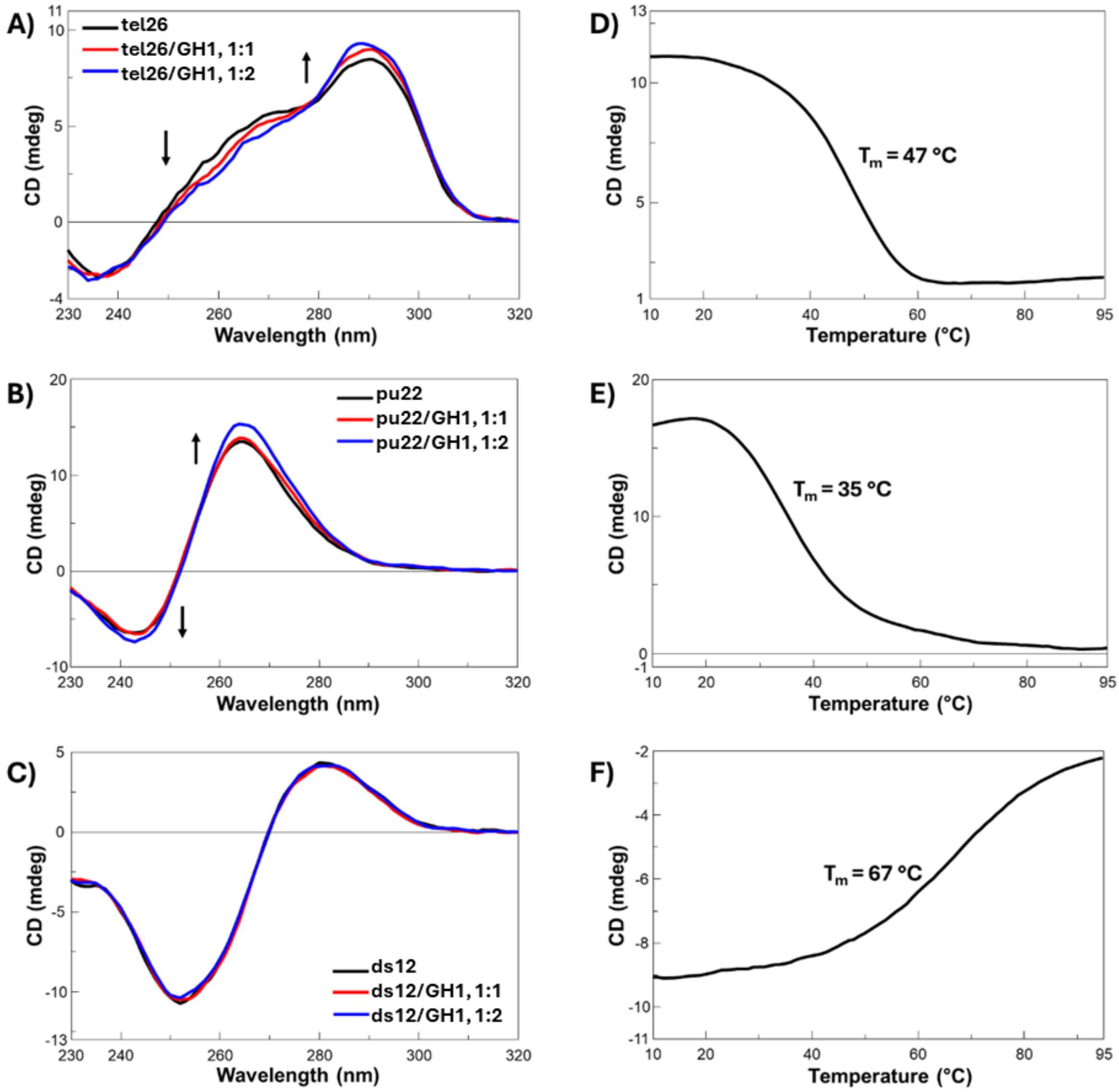

Figure S4. Left panels: CD spectra of 2 μM solutions of A) tel26, B) pu22 and C) ds12 in 20 mM KCl, 5 mM potassium phosphate buffer (pH 7) for tel26 and ds12 or in 10 mM Tris-HCl buffer (pH 7) for pu22 in the presence of increasing amounts (up to 2 equivalents) of **GH1**. Arrows indicate the direction of the CD band variation on increasing ligand concentration. Right panels: CD melting curves for D) tel26, E) pu22 and F) ds12 in the presence of **GH1** (2 equivalents) in 20 mM KCl, 5 mM potassium phosphate buffer (pH 7) for tel26 and ds12, recorded at 290 and 253 nm respectively, and in 10 mM Tris-HCl buffer (pH 7) for pu22, recorded at 263 nm.

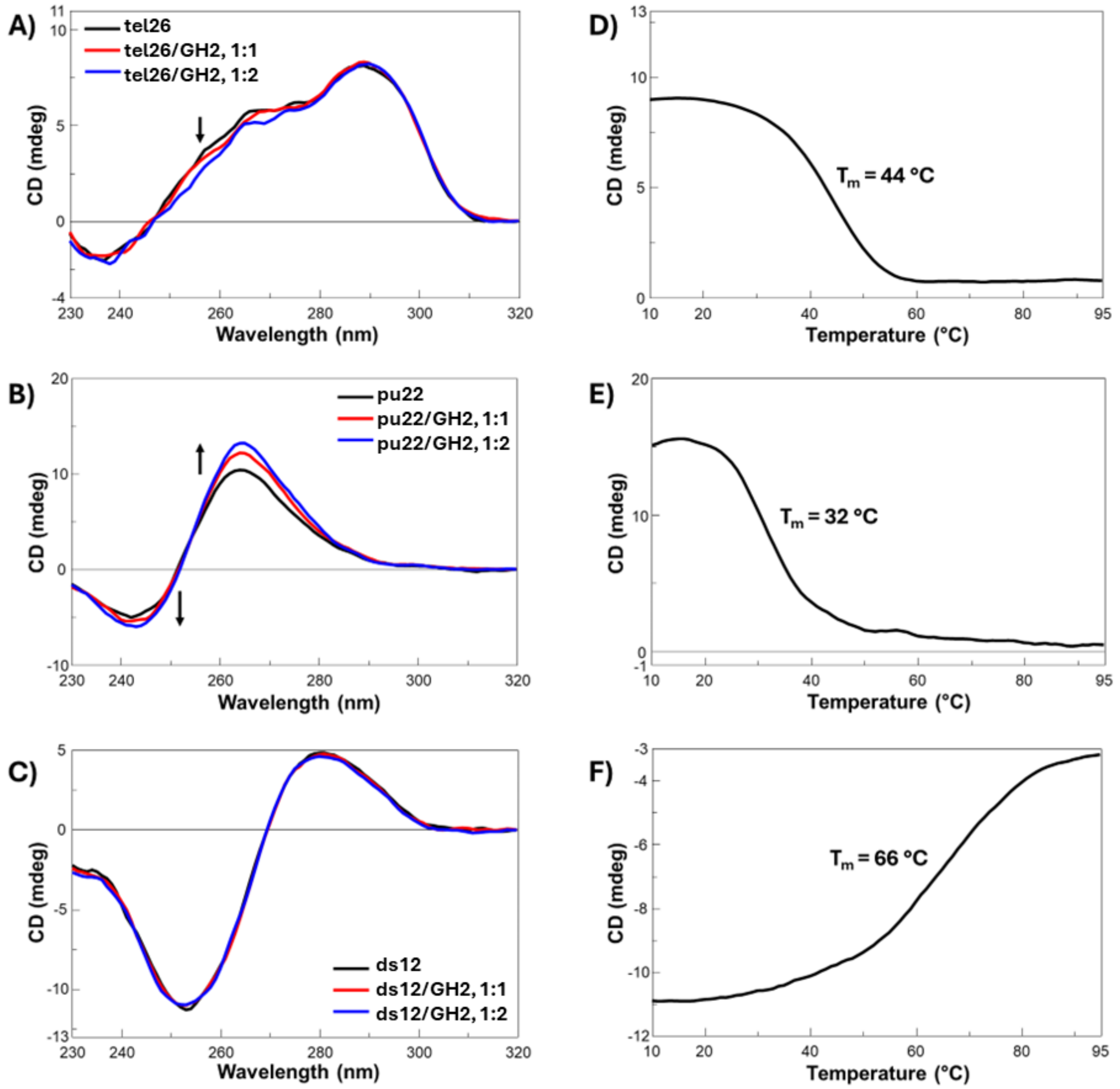

Figure S5. Left panels: CD spectra of 2 μM solutions of A) tel26, B) pu22 and C) ds12 in 20 mM KCl, 5 mM potassium phosphate buffer (pH 7) for tel26 and ds12 or in 10 mM Tris-HCl buffer (pH 7) for pu22 in the presence of increasing amounts (up to 2 equivalents) of **GH2**. Arrows indicate the direction of the CD band variation on increasing ligand concentration. Right panels: CD melting curves for D) tel26, E) pu22 and F) ds12 in the presence of **GH2** (2 equivalents) in 20 mM KCl, 5 mM potassium phosphate buffer (pH 7) for tel26 and ds12, recorded at 290 and 253 nm respectively, and in 10 mM Tris-HCl buffer (pH 7) for pu22, recorded at 263 nm.

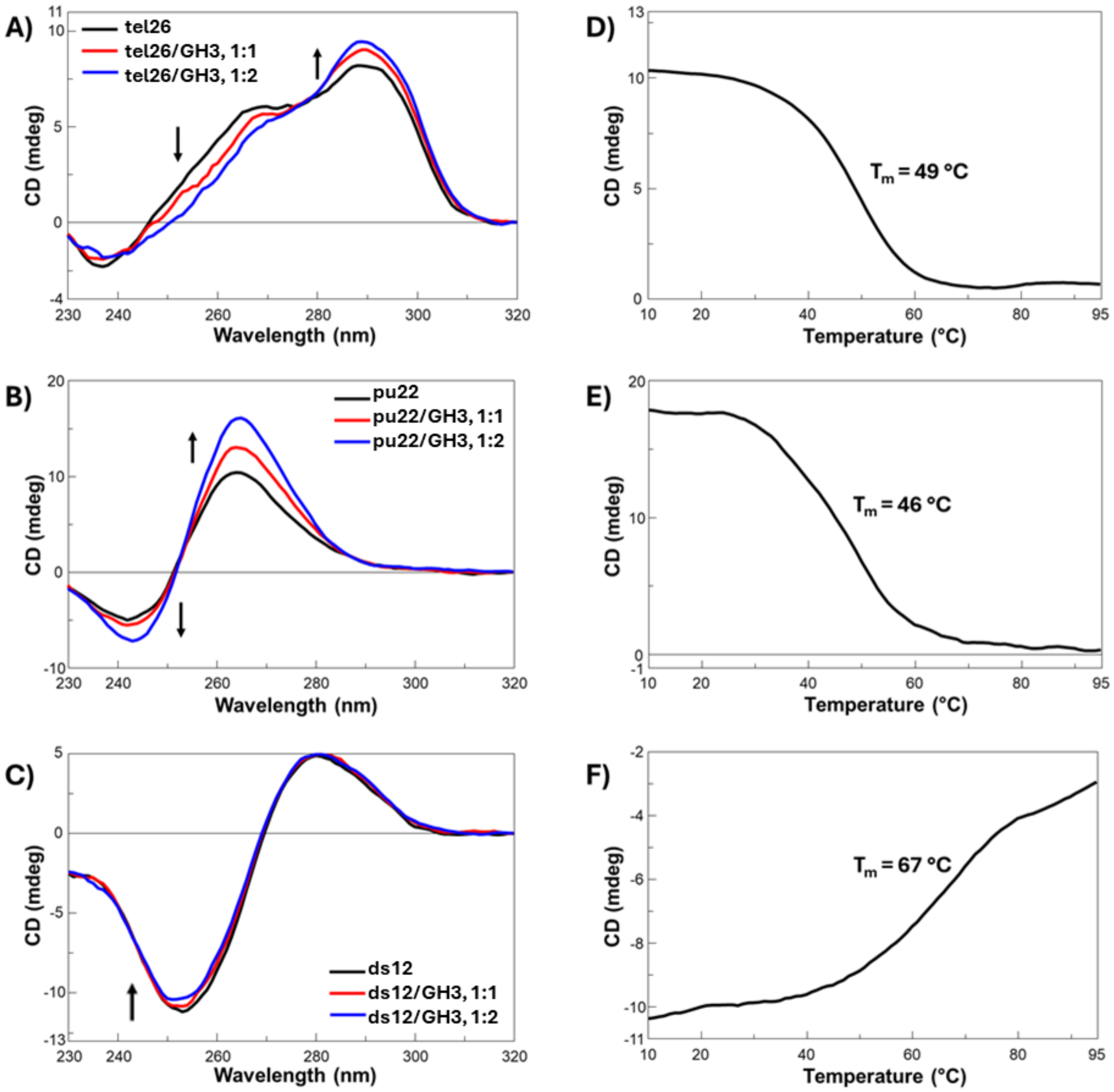

Figure S6. Left panels: CD spectra of 2 μM solutions of A) tel26, B) pu22 and C) ds12 in 20 mM KCl, 5 mM potassium phosphate buffer (pH 7) for tel26 and ds12 or in 10 mM Tris-HCl buffer (pH 7) for pu22 in the presence of increasing amounts (up to 2 equivalents) of **GH3**. Arrows indicate the direction of the CD band variation on increasing ligand concentration. Right panels: CD melting curves for D) tel26, E) pu22 and F) ds12 in the presence of **GH3** (2 equivalents) in 20 mM KCl, 5 mM potassium phosphate buffer (pH 7) for tel26 and ds12, recorded at 290 and 253 nm respectively, and in 10 mM Tris-HCl buffer (pH 7) for pu22, recorded at 263 nm.

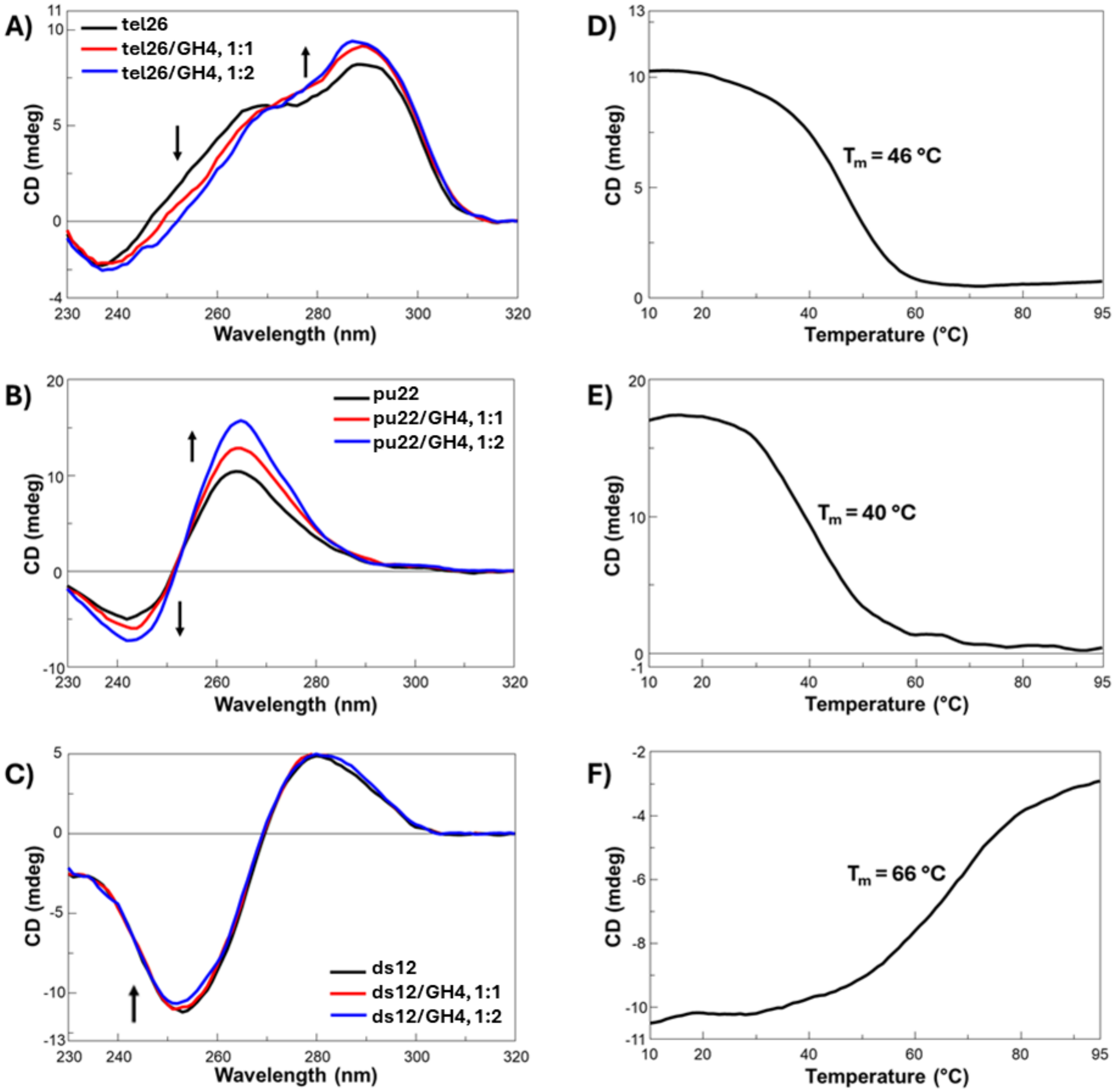

Figure S7. Left panels: CD spectra of 2 μM solutions of A) tel26, B) pu22 and C) ds12 in 20 mM KCl, 5 mM potassium phosphate buffer (pH 7) for tel26 and ds12 or in 10 mM Tris-HCl buffer (pH 7) for pu22 in the presence of increasing amounts (up to 2 equivalents) of **GH4**. Arrows indicate the direction of the CD band variation on increasing ligand concentration. Right panels: CD melting curves for D) tel26, E) pu22 and F) ds12 in the presence of **GH4** (2 equivalents) in 20 mM KCl, 5 mM potassium phosphate buffer (pH 7) for tel26 and ds12, recorded at 290 and 253 nm respectively, and in 10 mM Tris-HCl buffer (pH 7) for pu22, recorded at 263 nm.

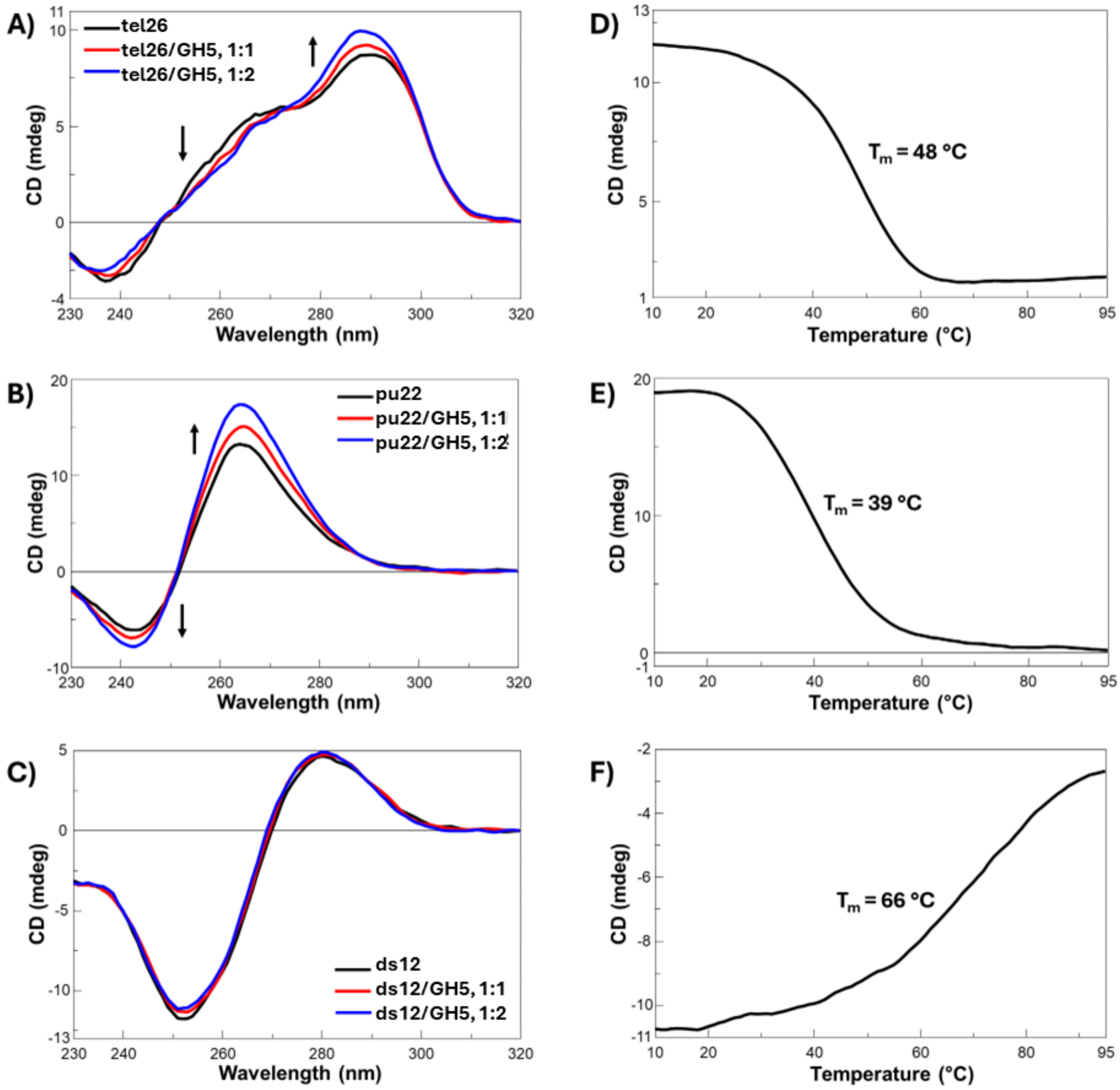

Figure S8. Left panels: CD spectra of 2 μM solutions of A) tel26, B) pu22 and C) ds12 in 20 mM KCl, 5 mM potassium phosphate buffer (pH 7) for tel26 and ds12 or in 10 mM Tris-HCl buffer (pH 7) for pu22 in the presence of increasing amounts (up to 2 equivalents) of **GH5**. Arrows indicate the direction of the CD band variation on increasing ligand concentration. Right panels: CD melting curves for D) tel26, E) pu22 and F) ds12 in the presence of **GH5** (2 equivalents) in 20 mM KCl, 5 mM potassium phosphate buffer (pH 7) for tel26 and ds12, recorded at 290 and 253 nm respectively, and in 10 mM Tris-HCl buffer (pH 7) for pu22, recorded at 263 nm.

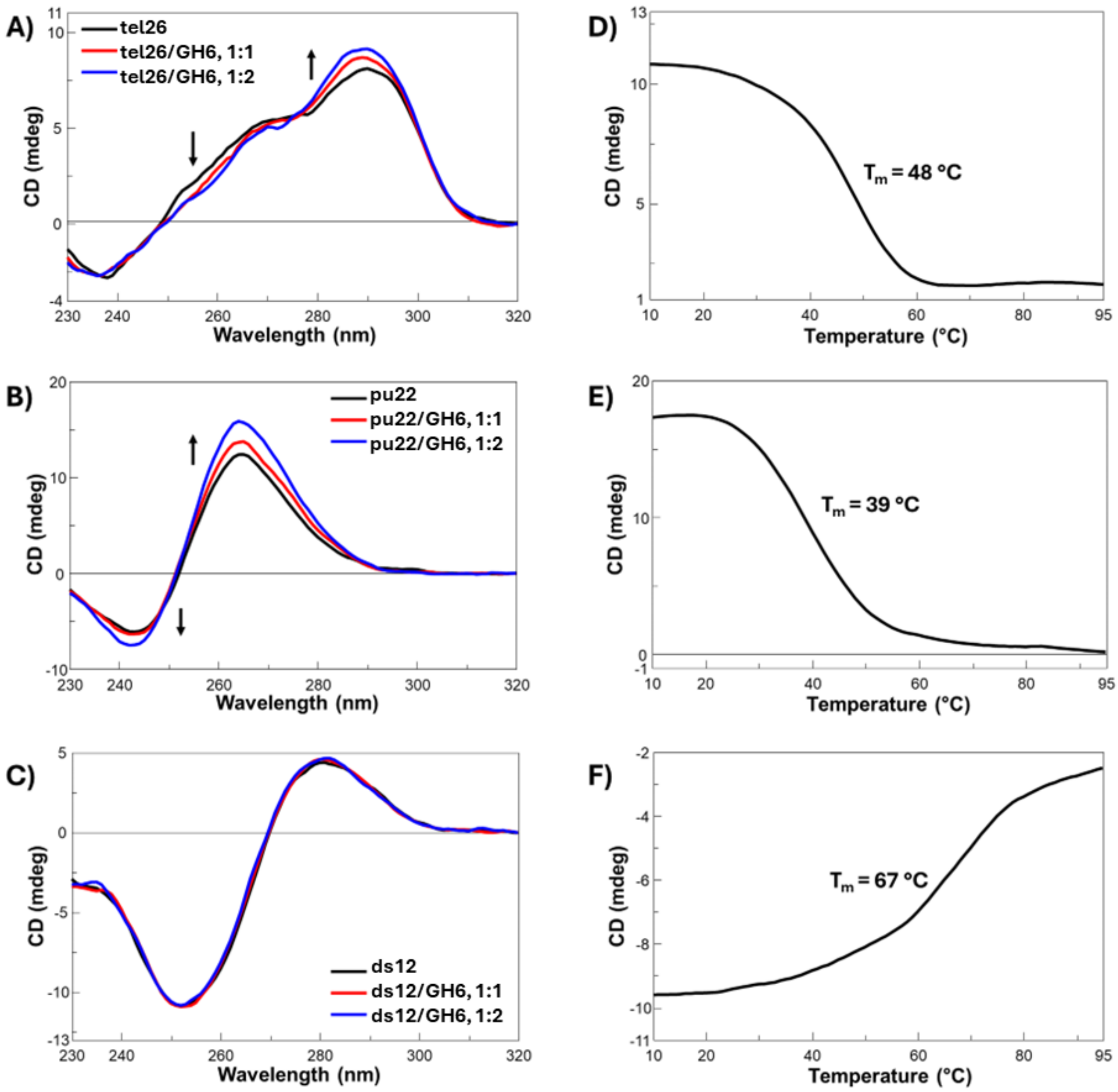

Figure S9. Left panels: CD spectra of 2 μM solutions of A) tel26, B) pu22 and C) ds12 in 20 mM KCl, 5 mM potassium phosphate buffer (pH 7) for tel26 and ds12 or in 10 mM Tris-HCl buffer (pH 7) for pu22 in the presence of increasing amounts (up to 2 equivalents) of **GH6**. Arrows indicate the direction of the CD band variation on increasing ligand concentration. Right panels: CD melting curves for D) tel26, E) pu22 and F) ds12 in the presence of **GH6** (2 equivalents) in 20 mM KCl, 5 mM potassium phosphate buffer (pH 7) for tel26 and ds12, recorded at 290 and 253 nm respectively, and in 10 mM Tris-HCl buffer (pH 7) for pu22, recorded at 263 nm.

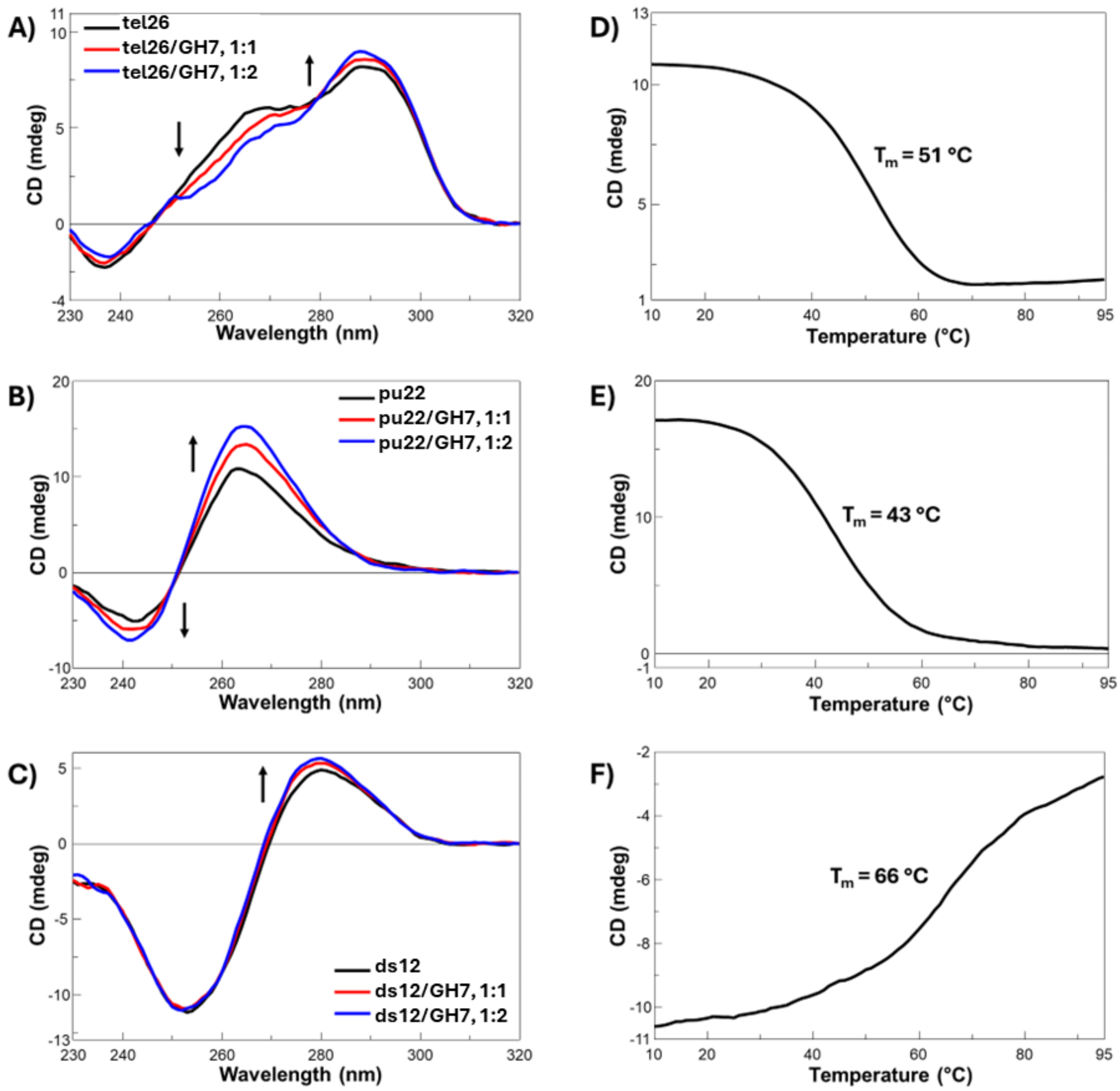

Figure S10. Left panels: CD spectra of 2 µM solutions of A) tel26, B) pu22 and C) ds12 in 20 mM KCl, 5 mM potassium phosphate buffer (pH 7) for tel26 and ds12 or in 10 mM Tris-HCl buffer (pH 7) for pu22 in the presence of increasing amounts (up to 2 equivalents) of **GH7**. Arrows indicate the direction of the CD band variation on increasing ligand concentration. Right panels: CD melting curves for D) tel26, E) pu22 and F) ds12 in the presence of **GH7** (2 equivalents) in 20 mM KCl, 5 mM potassium phosphate buffer (pH 7) for tel26 and ds12, recorded at 290 and 253 nm respectively, and in 10 mM Tris-HCl buffer (pH 7) for pu22, recorded at 263 nm.

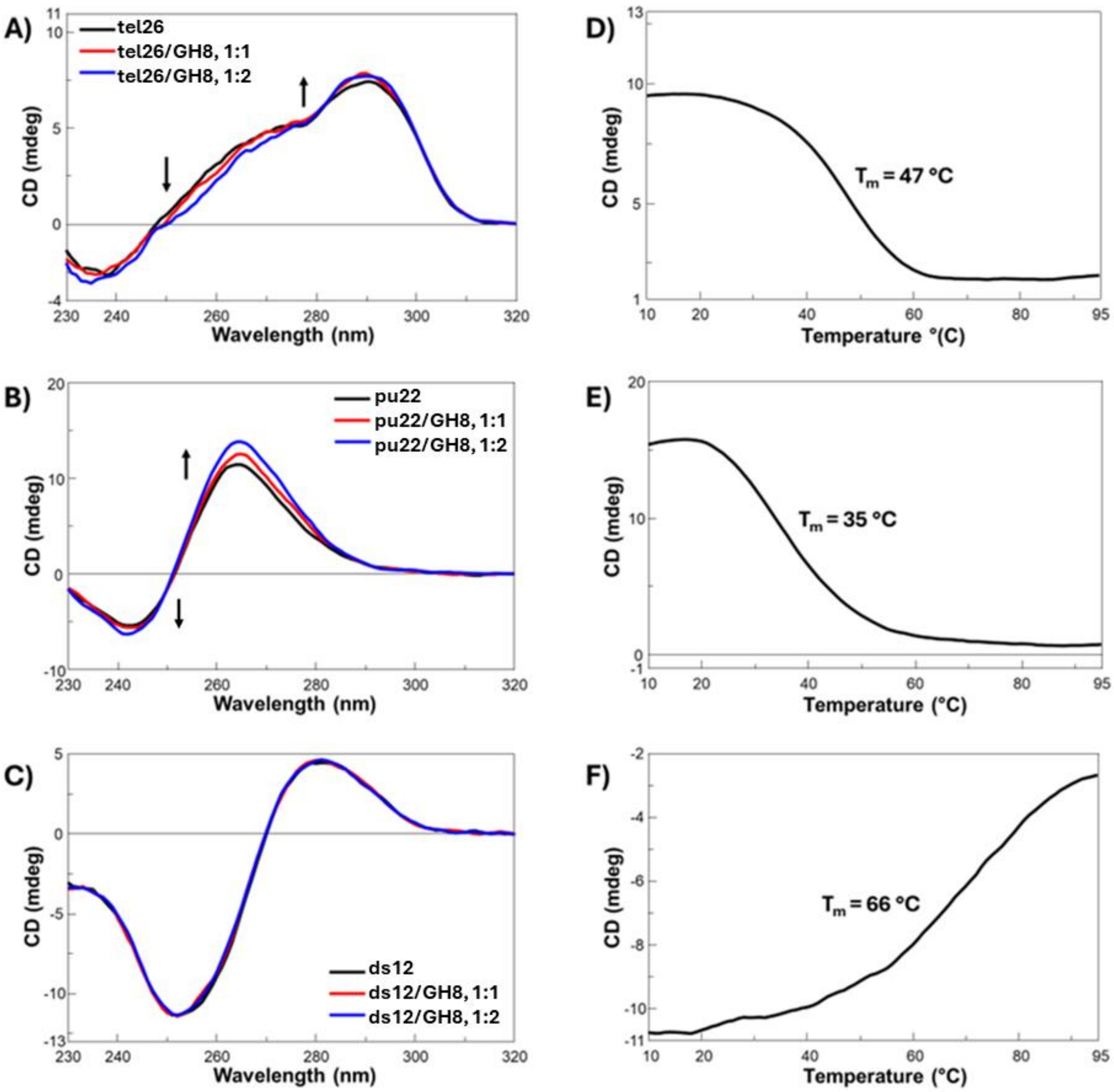

Figure S11. Left panels: CD spectra of 2 μM solutions of A) tel26, B) pu22 and C) ds12 in 20 mM KCl, 5 mM potassium phosphate buffer (pH 7) for tel26 and ds12 or in 10 mM Tris-HCl buffer (pH 7) for pu22 in the presence of increasing amounts (up to 2 equivalents) of **GH8**. Arrows indicate the direction of the CD band variation on increasing ligand concentration. Right panels: CD melting curves for D) tel26, E) pu22 and F) ds12 in the presence of **GH8** (2 equivalents) in 20 mM KCl, 5 mM potassium phosphate buffer (pH 7) for tel26 and ds12, recorded at 290 and 253 nm respectively, and in 10 mM Tris-HCl buffer (pH 7) for pu22, recorded at 263 nm.

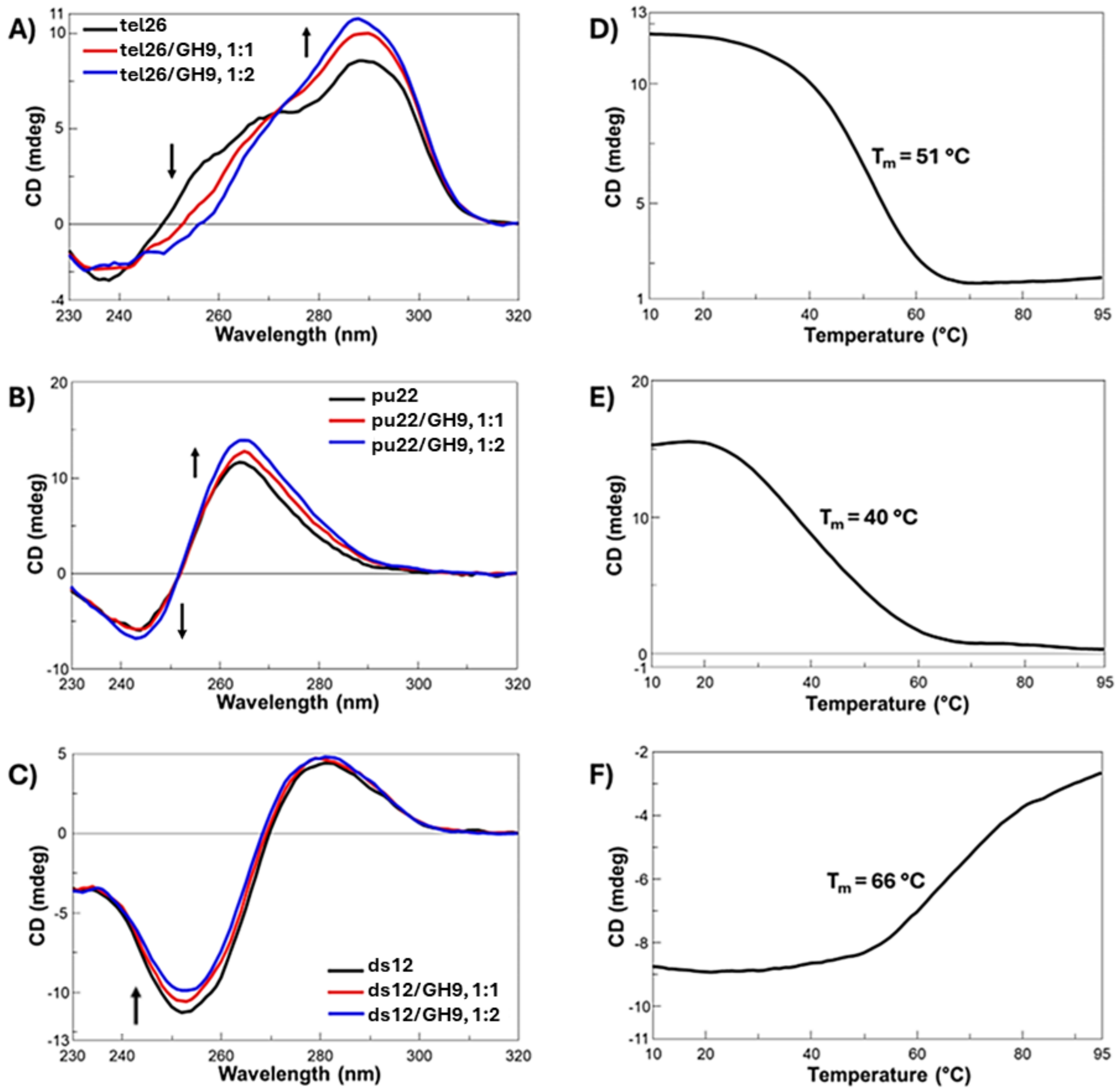

Figure S12. Left panels: CD spectra of 2 μM solutions of A) tel26, B) pu22 and C) ds12 in 20 mM KCl, 5 mM potassium phosphate buffer (pH 7) for tel26 and ds12 or in 10 mM Tris-HCl buffer (pH 7) for pu22 in the presence of increasing amounts (up to 2 equivalents) of **GH9**. Arrows indicate the direction of the CD band variation on increasing ligand concentration. Right panels: CD melting curves for D) tel26, E) pu22 and F) ds12 in the presence of **GH9** (2 equivalents) in 20 mM KCl, 5 mM potassium phosphate buffer (pH 7) for tel26 and ds12, recorded at 290 and 253 nm respectively, and in 10 mM Tris-HCl buffer (pH 7) for pu22, recorded at 263 nm.

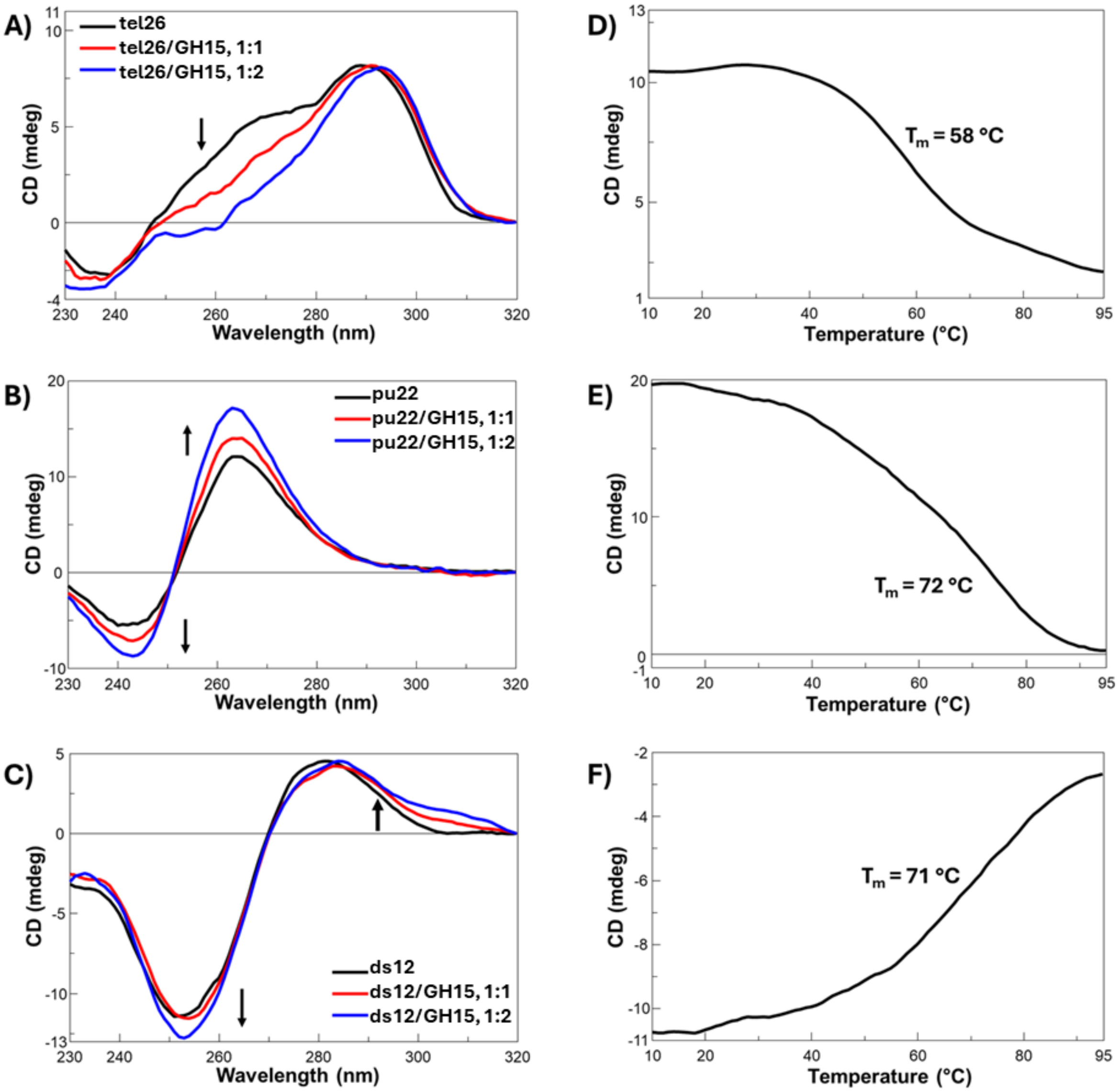

Figure S13. Left panels: CD spectra of 2 µM solutions of A) tel26, B) pu22 and C) ds12 in 20 mM KCl, 5 mM potassium phosphate buffer (pH 7) for tel26 and ds12 or in 10 mM Tris-HCl buffer (pH 7) for pu22 in the presence of increasing amounts (up to 2 equivalents) of **GH15**. Arrows indicate the direction of the CD band variation on increasing ligand concentration. Right panels: CD melting curves for D) tel26, E) pu22 and F) ds12 in the presence of **GH15** (2 equivalents) in 20 mM KCl, 5 mM potassium phosphate buffer (pH 7) for tel26 and ds12, recorded at 290 and 253 nm respectively, and in 10 mM Tris-HCl buffer (pH 7) for pu22, recorded at 263 nm.

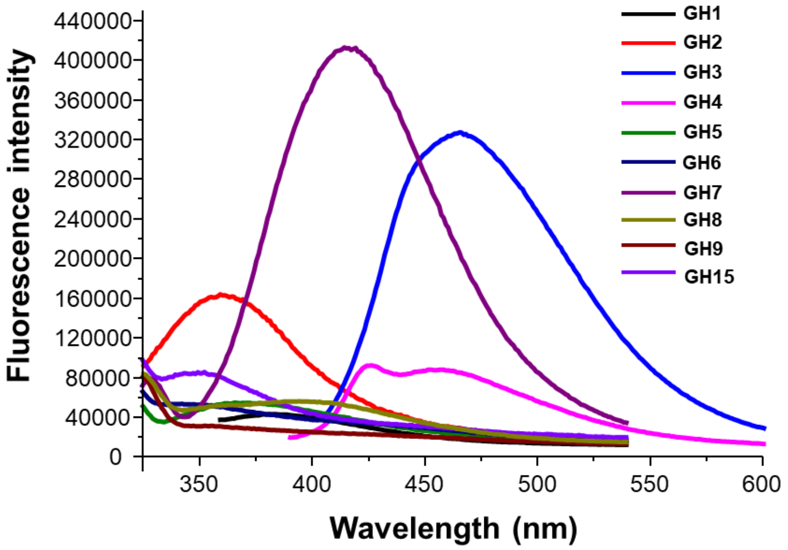

Figure S14. Fluorescence spectra of the investigated guanyl hydrazones at 2 μM concentration in 20 mM KCl, 5 mM potassium phosphate buffer (pH 7).

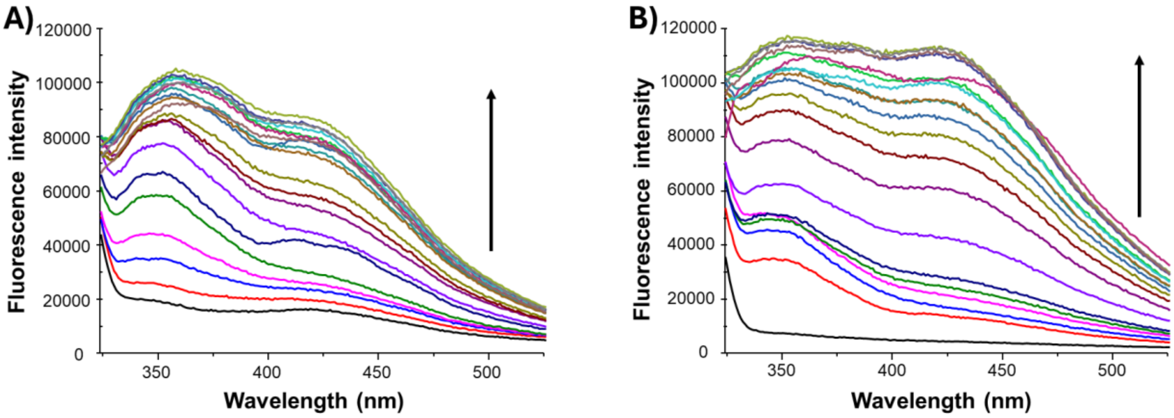

Figure S15. Fluorescence spectra of free A) tel26 and B) pu22 in 20 mM KCl, 5 mM potassium phosphate buffer (pH 7). Arrows indicate the direction of the changes in fluorescence intensity on increasing DNA concentration (from 0.1 μM to 10 μM).

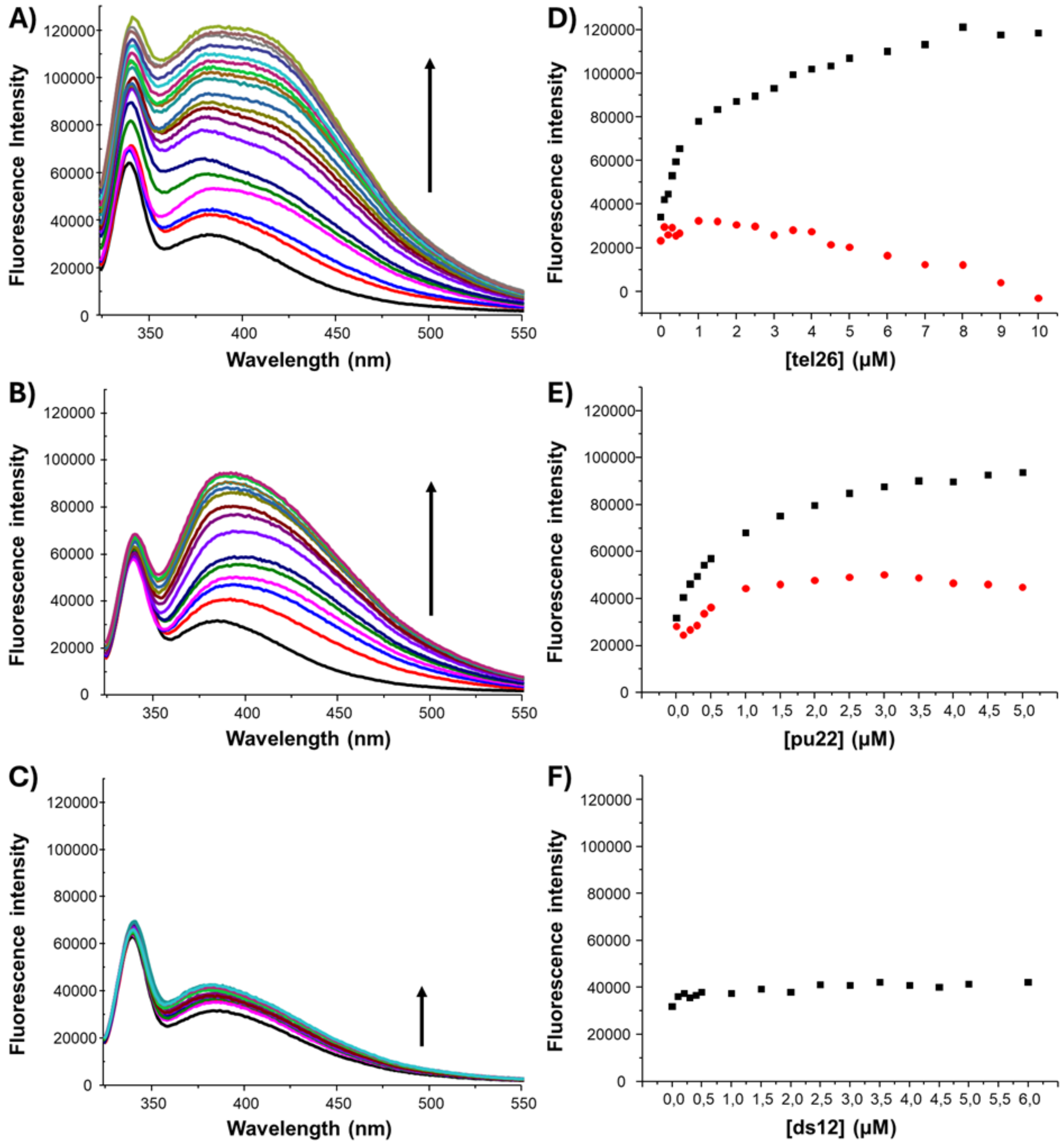

Figure S16. Left panels: Fluorescence spectra obtained by adding increasing amounts of A) tel26, B) pu22 and C) ds12 to solutions at a fixed concentration of **GH1** (i.e. 2 μM). Arrows indicate the variation of fluorescence intensity on increasing DNA concentration. Right panels: Fluorescence intensity at the ligand emission maximum *vs.* concentration of D) tel26, E) pu22 and F) ds12 without (black squares) or after (red circles) subtraction of the oligonucleotide contribution.

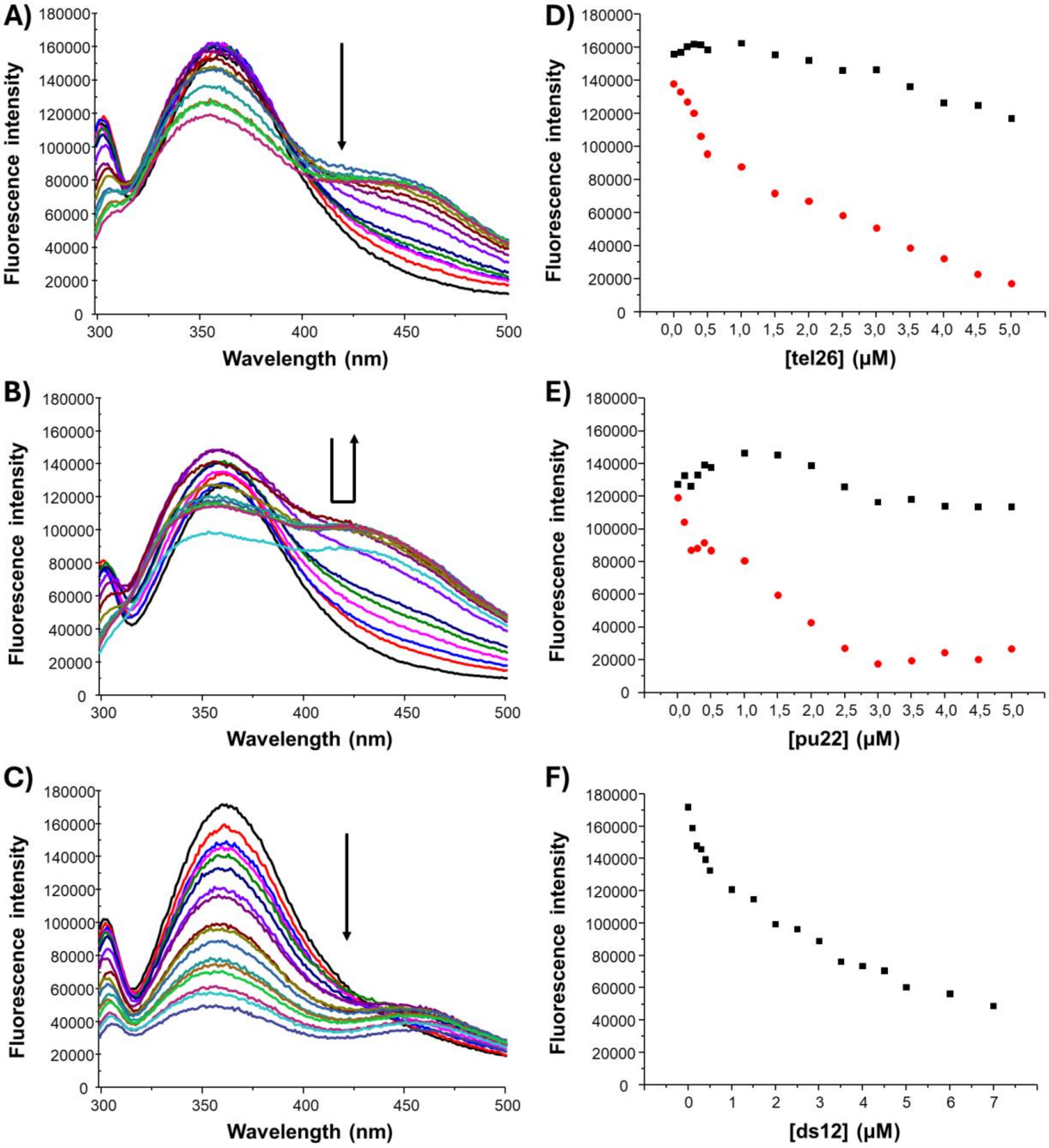

Figure S17. Left panels: Fluorescence spectra obtained by adding increasing amounts of A) tel26, B) pu22 and C) ds12 to solutions at a fixed concentration of **GH2** (i.e. 2 μM). Arrows indicate the variation of fluorescence intensity on increasing DNA concentration. Right panels: Fluorescence intensity at the ligand emission maximum *vs.* concentration of D) tel26, E) pu22 and F) ds12 without (black squares) or after (red circles) subtraction of the oligonucleotide contribution.

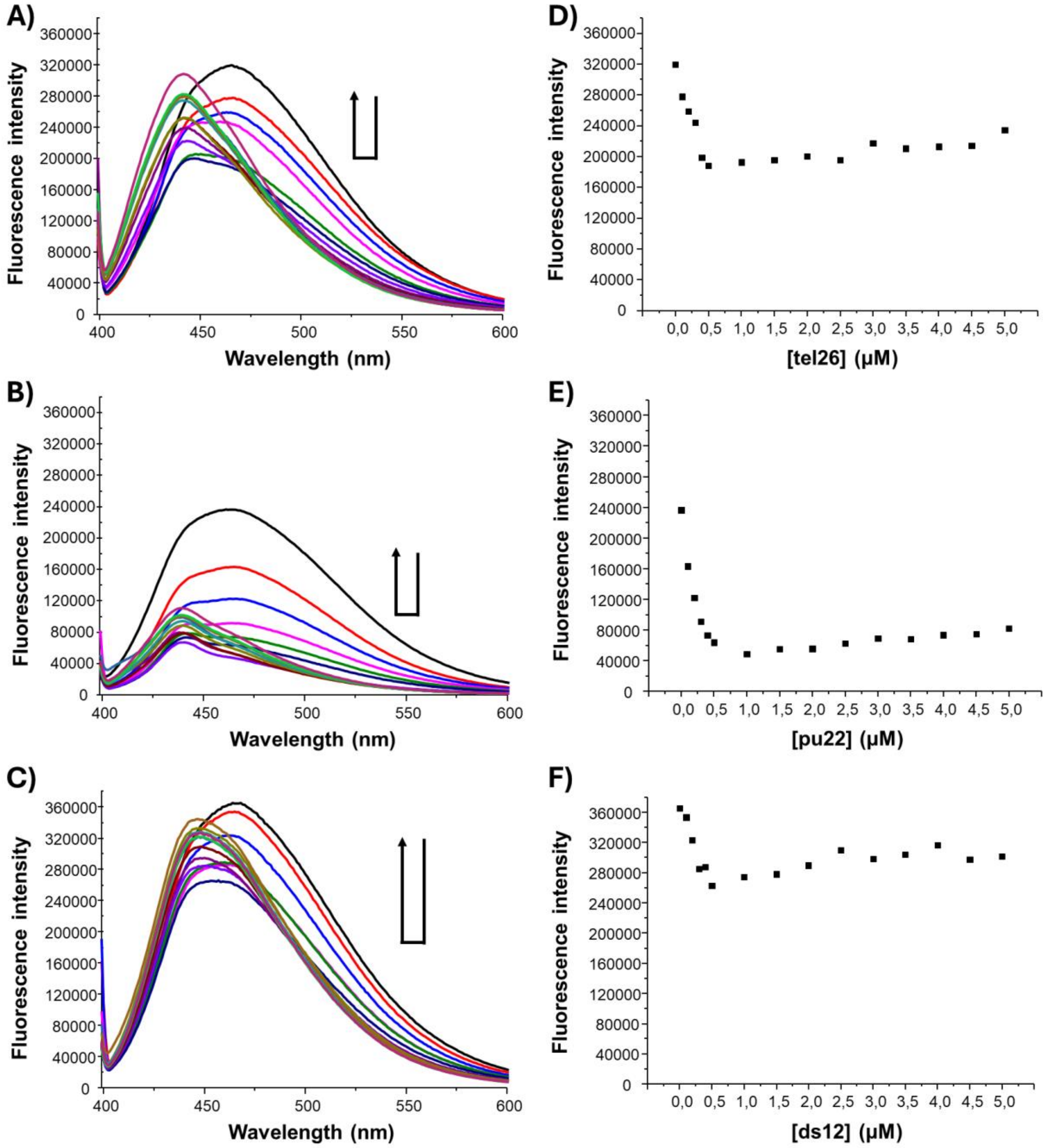

Figure S18. Left panels: Fluorescence spectra obtained by adding increasing amounts of A) tel26, B) pu22 and C) ds12 to solutions at a fixed concentration of **GH3** (i.e. 2 μM). Arrows indicate the direction of the variation of fluorescence intensity on increasing DNA concentration. Right panels: Fluorescence intensity at the ligand emission maximum *vs.* concentration of D) tel26, E) pu22 and F) ds12. No subtraction of the oligonucleotide contribution was here necessary.

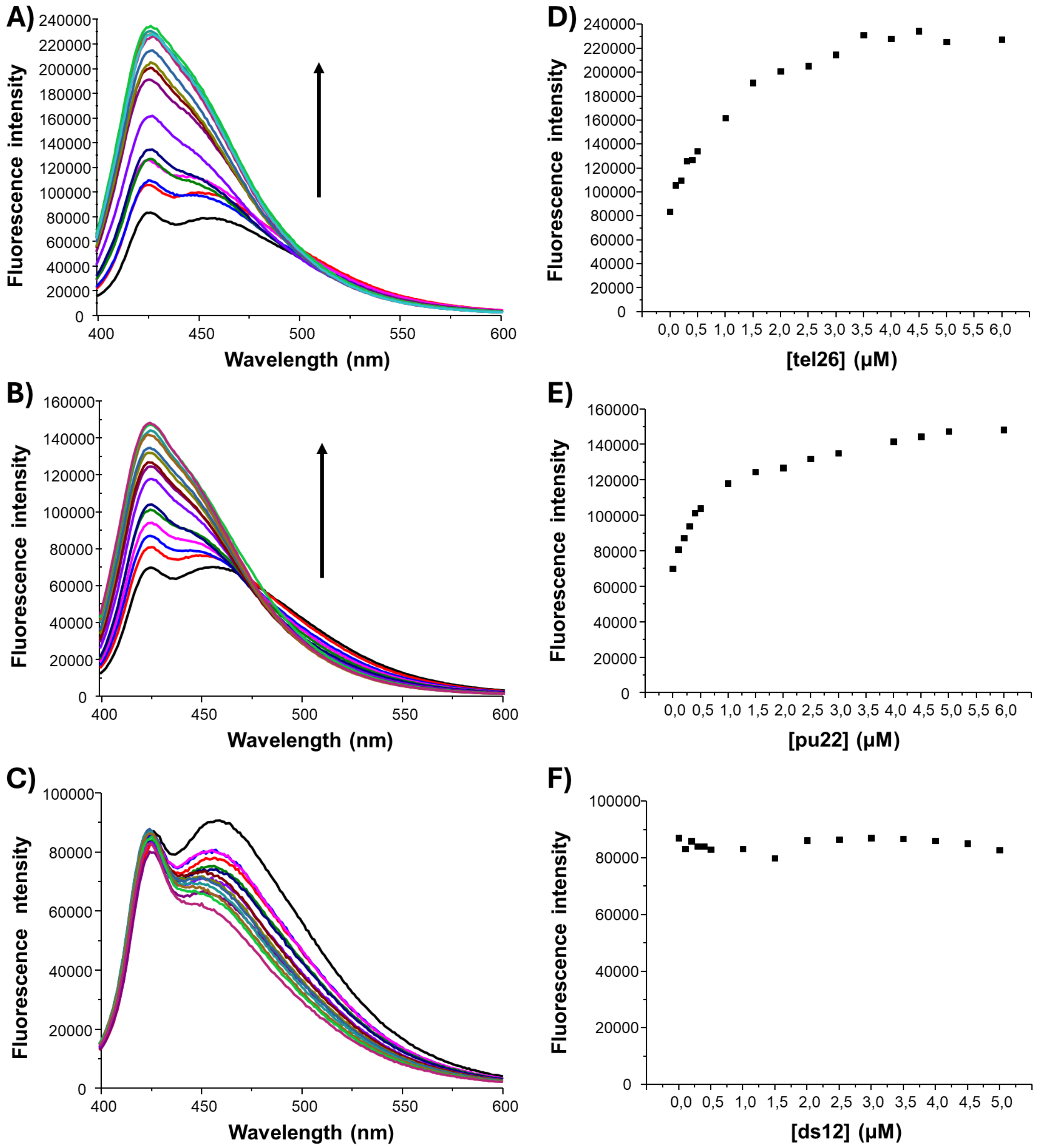

Figure S19. Left panels: Fluorescence spectra obtained by adding increasing amounts of A) tel26, B) pu22 and C) ds12 to solutions at a fixed concentration of **GH4** (i.e. 2 μM). Arrows indicate the direction of the variation of fluorescence intensity on increasing DNA concentration. Right panels: Fluorescence intensity at the ligand emission maximum *vs.* concentration of D) tel26, E) pu22 and F) ds12. No subtraction of the oligonucleotide contribution was here necessary.

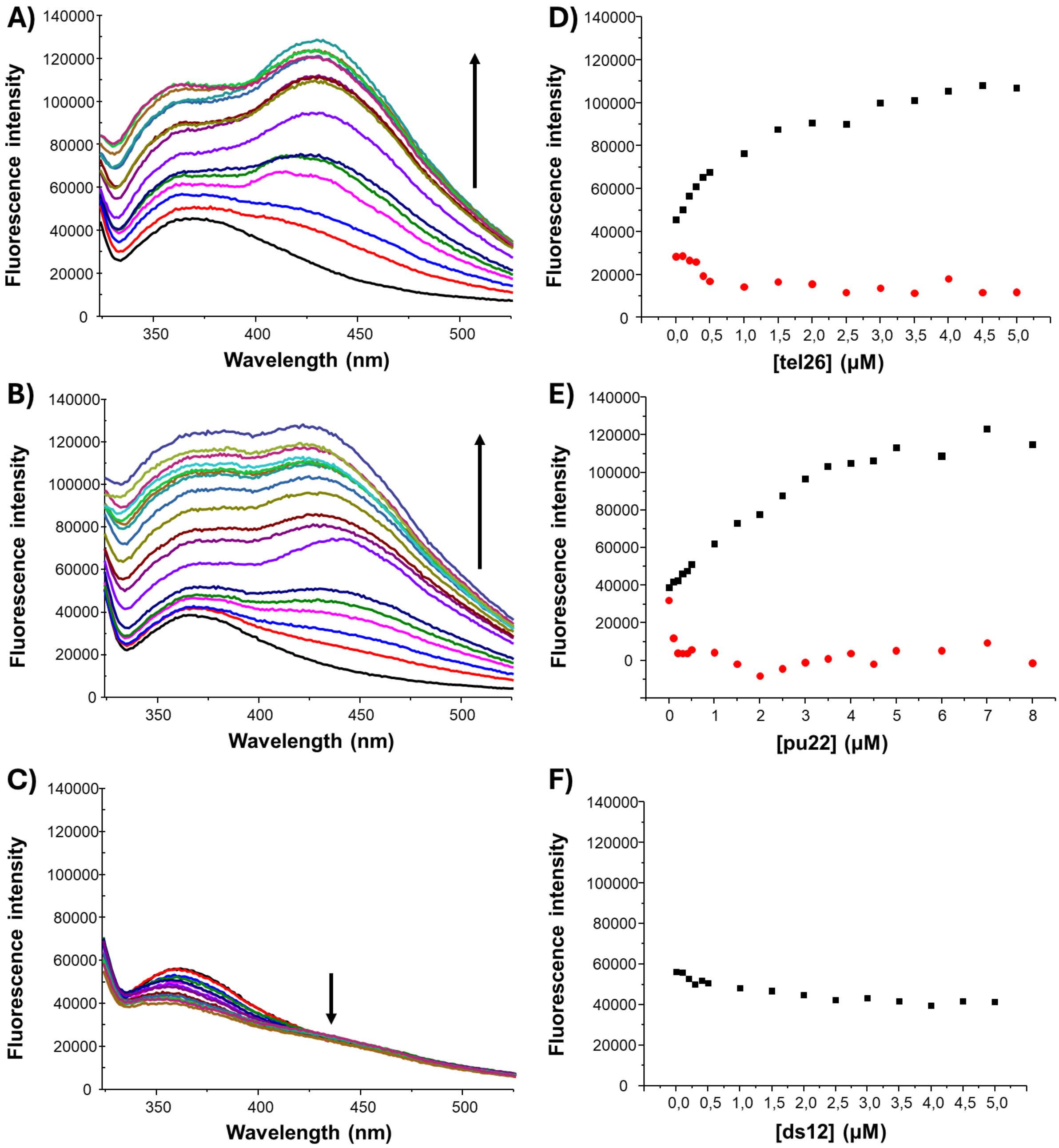

Figure S20. Left panels: Fluorescence spectra obtained by adding increasing amounts of A) tel26, B) pu22 and C) ds12 to solutions at a fixed concentration of **GH5** (i.e. 2 μM). Arrows indicate the direction of the variation of fluorescence intensity on increasing DNA concentration. Right panels: Fluorescence intensity at the ligand emission maximum *vs.* concentration of D) tel26, E) pu22 and F) ds12 without (black squares) or after (red circles) subtraction of the oligonucleotide contribution.

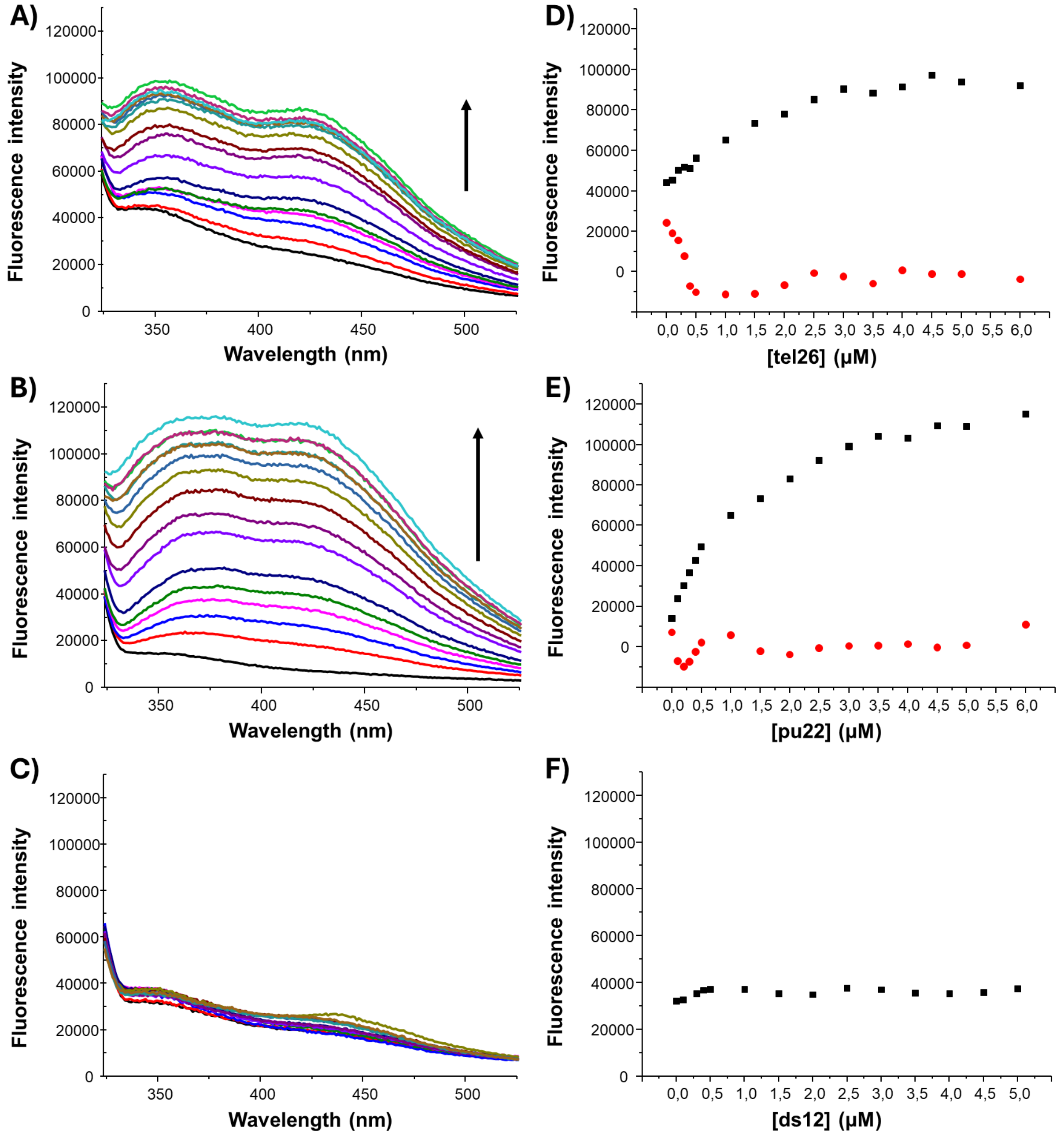

Figure S21. Left panels: Fluorescence spectra obtained by adding increasing amounts of A) tel26, B) pu22 and C) ds12 to solutions at a fixed concentration of **GH6** (i.e. 2 μM). Arrows indicate the direction of the variation of fluorescence intensity on increasing DNA concentration. Right panels: Fluorescence intensity at the ligand emission maximum *vs.* concentration of D) tel26, E) pu22 and F) ds12 without (black squares) or after (red circles) subtraction of the oligonucleotide contribution.

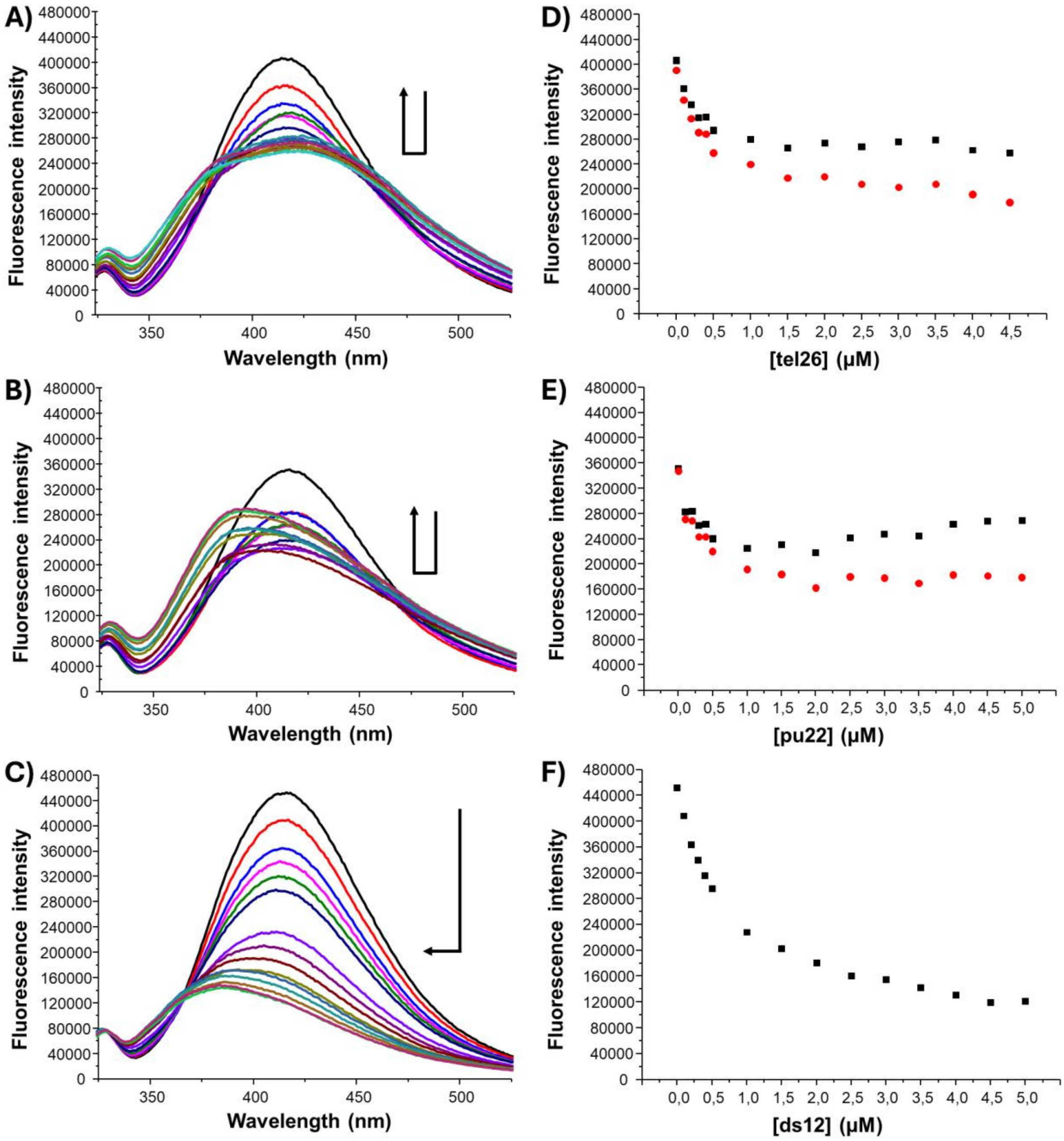

Figure S22. Left panels: Fluorescence spectra obtained by adding increasing amounts of A) tel26, B) pu22 and C) ds12 to solutions at a fixed concentration of **GH7** (i.e. 2 μM). Arrows indicate the direction of the variation of fluorescence intensity on increasing DNA concentration. Right panels: Fluorescence intensity at the ligand emission maximum *vs.* concentration of D) tel26, E) pu22 and F) ds12 without (black squares) or after (red circles) subtraction of the oligonucleotide contribution.

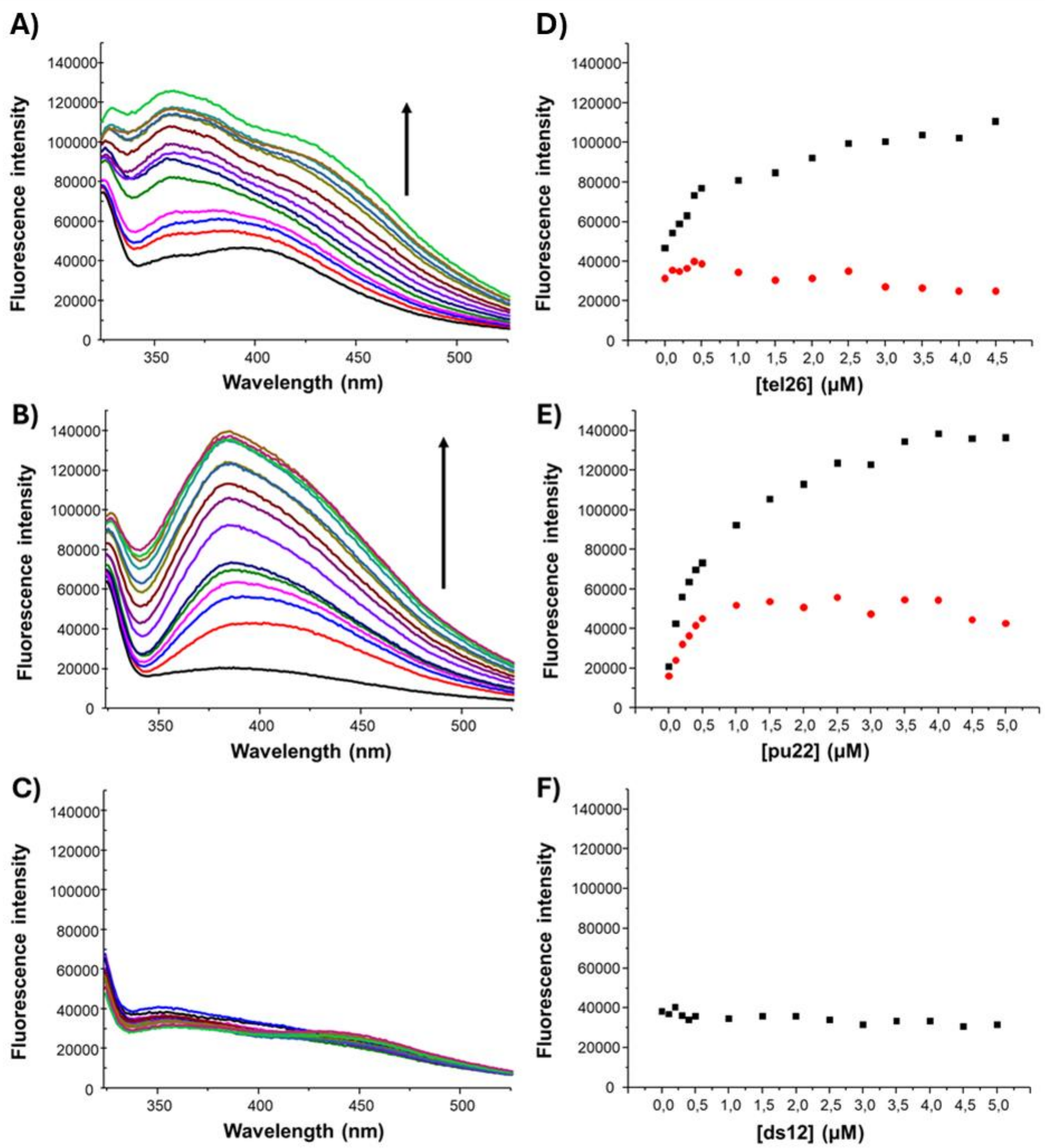

Figure S23. Left panels: Fluorescence spectra obtained by adding increasing amounts of A) tel26, B) pu22 and C) ds12 to solutions at a fixed concentration of **GH8** (i.e. 2 μM). Arrows indicate the direction of the variation of fluorescence intensity on increasing DNA concentration. Right panels: Fluorescence intensity at the ligand emission maximum *vs.* concentration of D) tel26, E) pu22 and F) ds12 without (black squares) or after (red circles) subtraction of the oligonucleotide contribution.

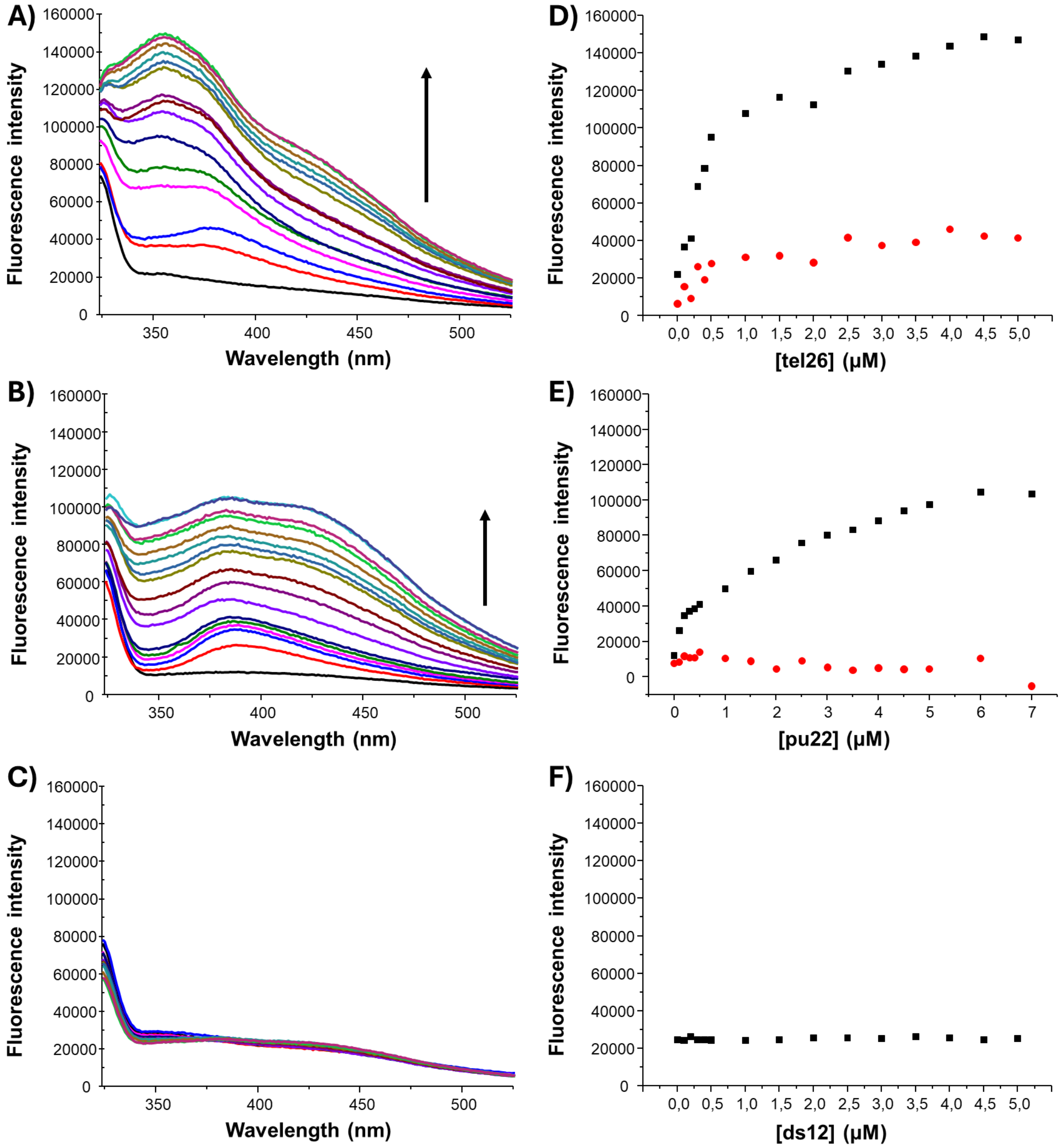

Figure S24. Left panels: Fluorescence spectra obtained by adding increasing amounts of A) tel26, B) pu22 and C) ds12 to solutions at a fixed concentration of **GH9** (i.e. 2 μM). Arrows indicate the direction of the variation of fluorescence intensity on increasing DNA concentration. Right panels: Fluorescence intensity at the ligand emission maximum *vs.* concentration of D) tel26, E) pu22 and F) ds12 without (black squares) or after (red circles) subtraction of the oligonucleotide contribution.

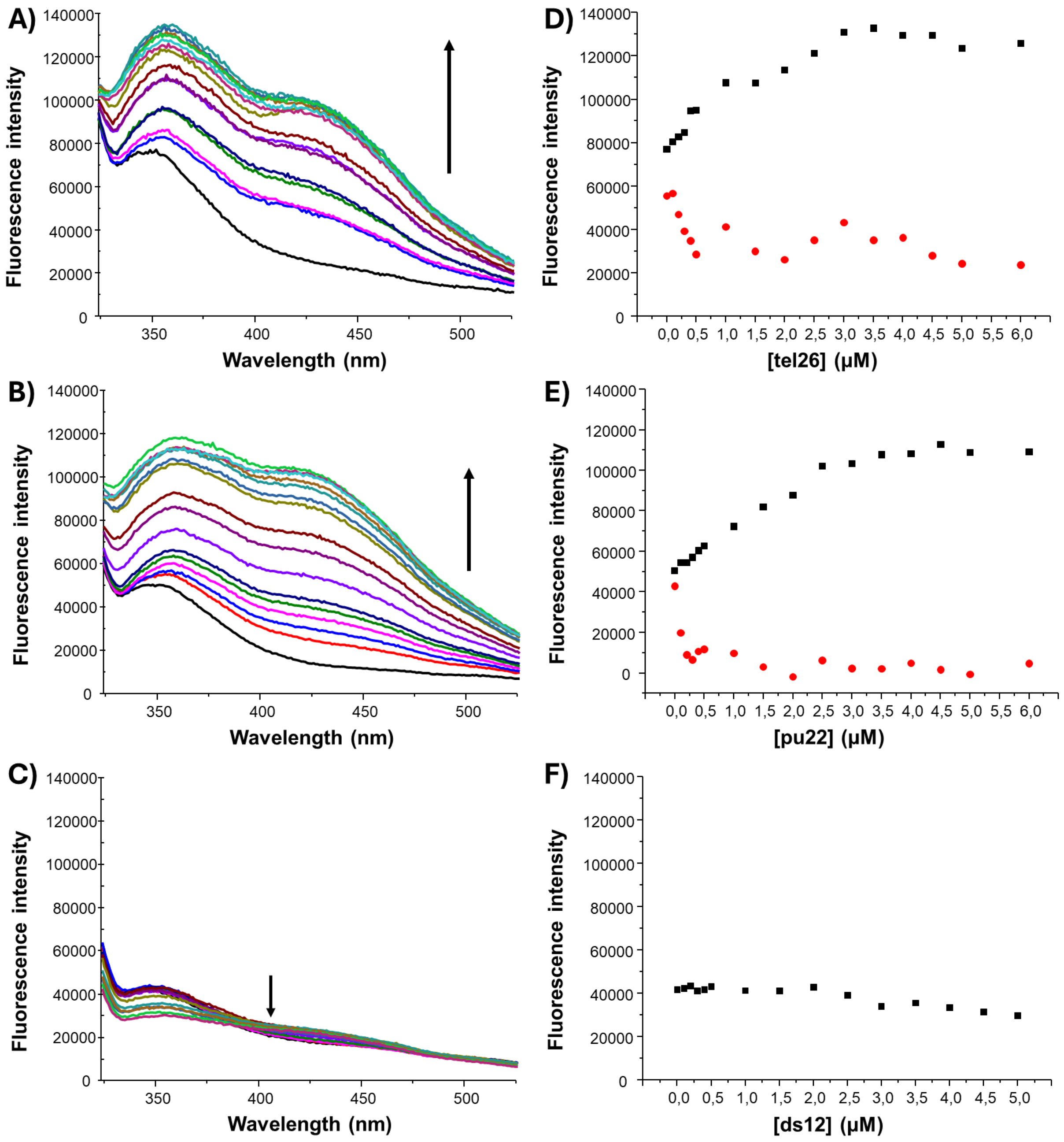

Figure S25. Left panels: Fluorescence spectra obtained by adding increasing amounts of A) tel26, B) pu22 and C) ds12 to solutions at a fixed concentration of **GH15** (i.e. 2 μM). Arrows indicate the direction of the variation of fluorescence intensity on increasing DNA concentration. Right panels: Fluorescence intensity at the ligand emission maximum *vs.* concentration of D) tel26, E) pu22 and F) ds12 without (black squares) or after (red circles) subtraction of the oligonucleotide contribution.

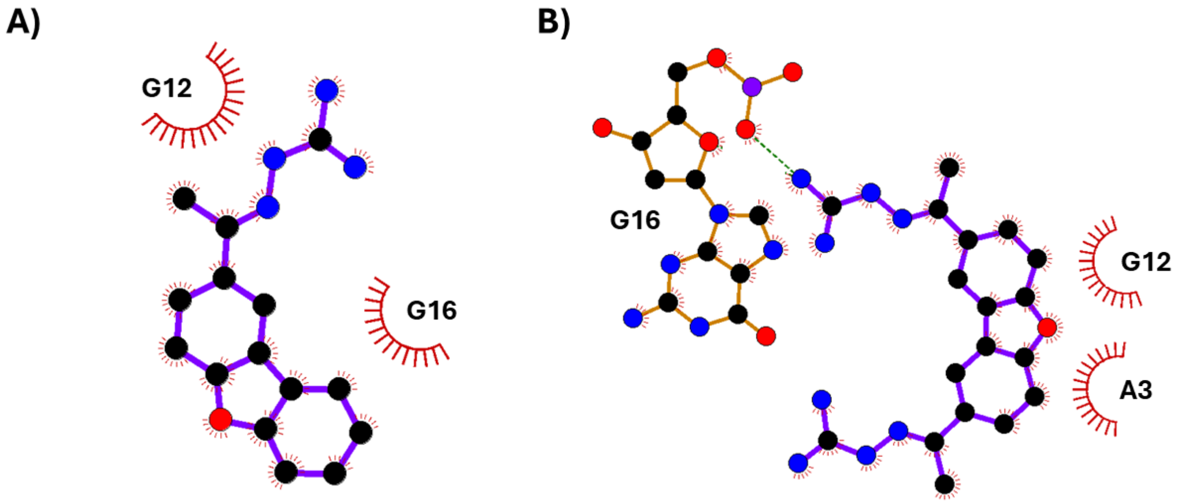

Figure S26. 2D map of the interactions for A) **GH6** and B) **GH15** when docked to tel26 G-quadruplex (PDB 5MVB). C, N, O and P atoms are reported in black, blue, red and purple, respectively. Hydrogen atoms are not depicted for ease of illustration. Electrostatic interactions and hydrophobic contacts are depicted as green dashed lines and red arcs with radiating lines, respectively. Nucleotides involved in the interactions are labeled.

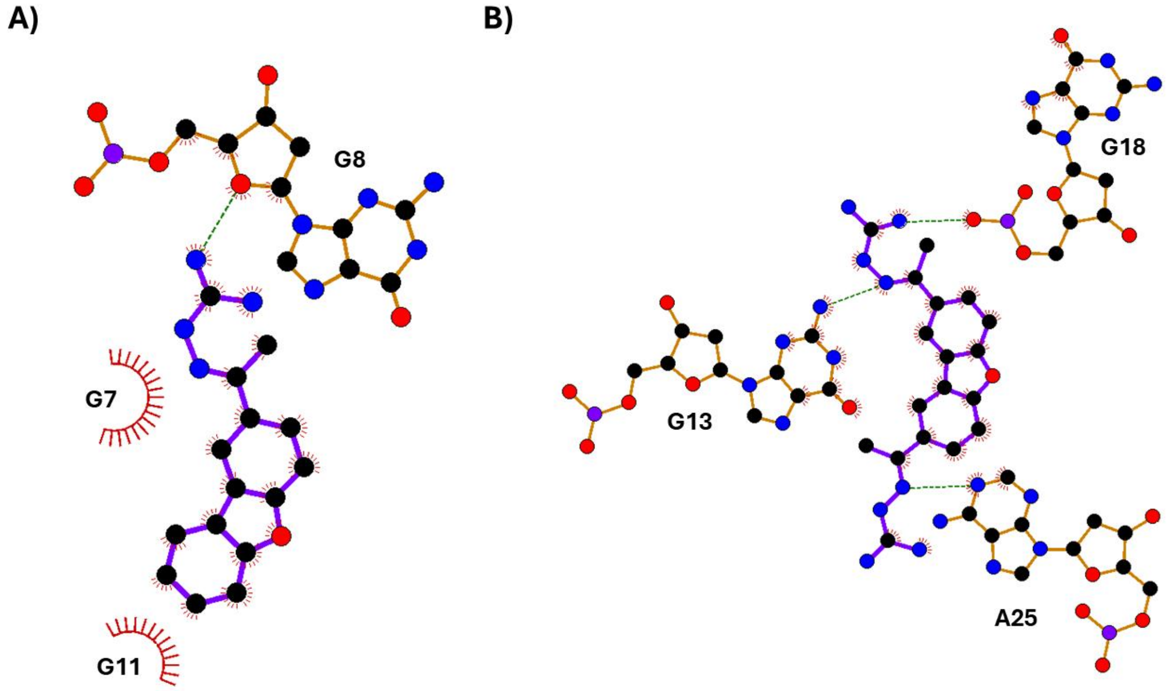

Figure S27. 2D map of the interactions for A) **GH6** and B) **GH15** when docked to pu22 G-quadruplex (PDB 2L7V). C, N, O and P atoms are reported in black, blue, red and purple, respectively. Hydrogen atoms are not depicted for ease of illustration. Electrostatic interactions/H-bond and hydrophobic contacts are depicted as green dashed lines and red arcs with radiating lines, respectively. Nucleotides involved in the interactions are labeled.

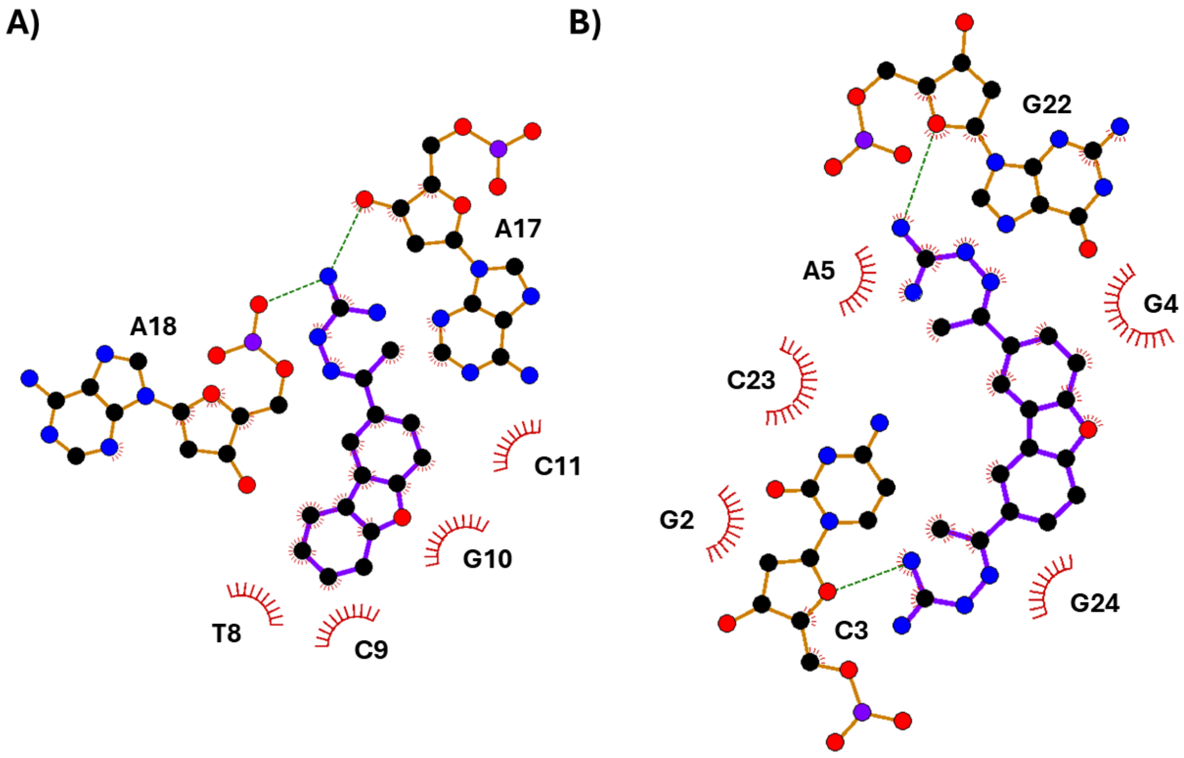

Figure S28. 2D map of the interactions for A) **GH6** and B) **GH15** when docked to ds12 duplex (from PDB 1NAJ). C, N, O and P atoms are reported in black, blue, red and purple, respectively. Hydrogen atoms are not depicted for ease of illustration. Electrostatic interactions/H-bond and hydrophobic contacts are depicted as green dashed lines and red arcs with radiating lines, respectively. Nucleotides involved in the interactions are labeled.

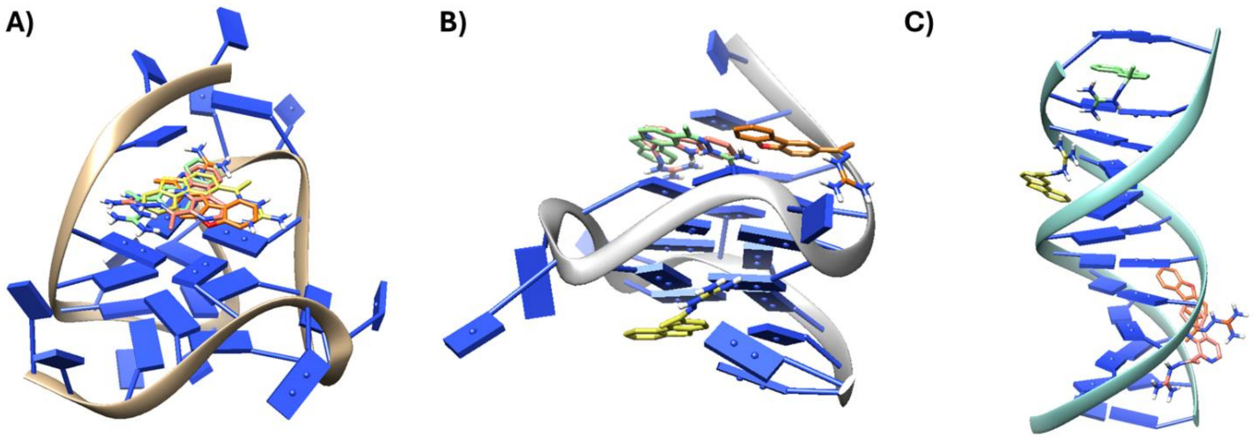

Figure S29. Binding modes of **GH1**, **GH4**, **GH6** and **GH8** when docked to A) tel26 G-quadruplex (PDB 5MVB), B) pu22 G-quadruplex (PDB 2L7V) and C) ds12 duplex (from PDB 1NAJ). Ligands and G-quadruplexes/duplex are represented as sticks and ribbon, respectively. **GH1**, **GH4**, **GH6** and **GH8** are coloured green, pink, orange and yellow, respectively. 5’- and 3’-end of the G-quadruplexes are at the top and bottom, respectively.

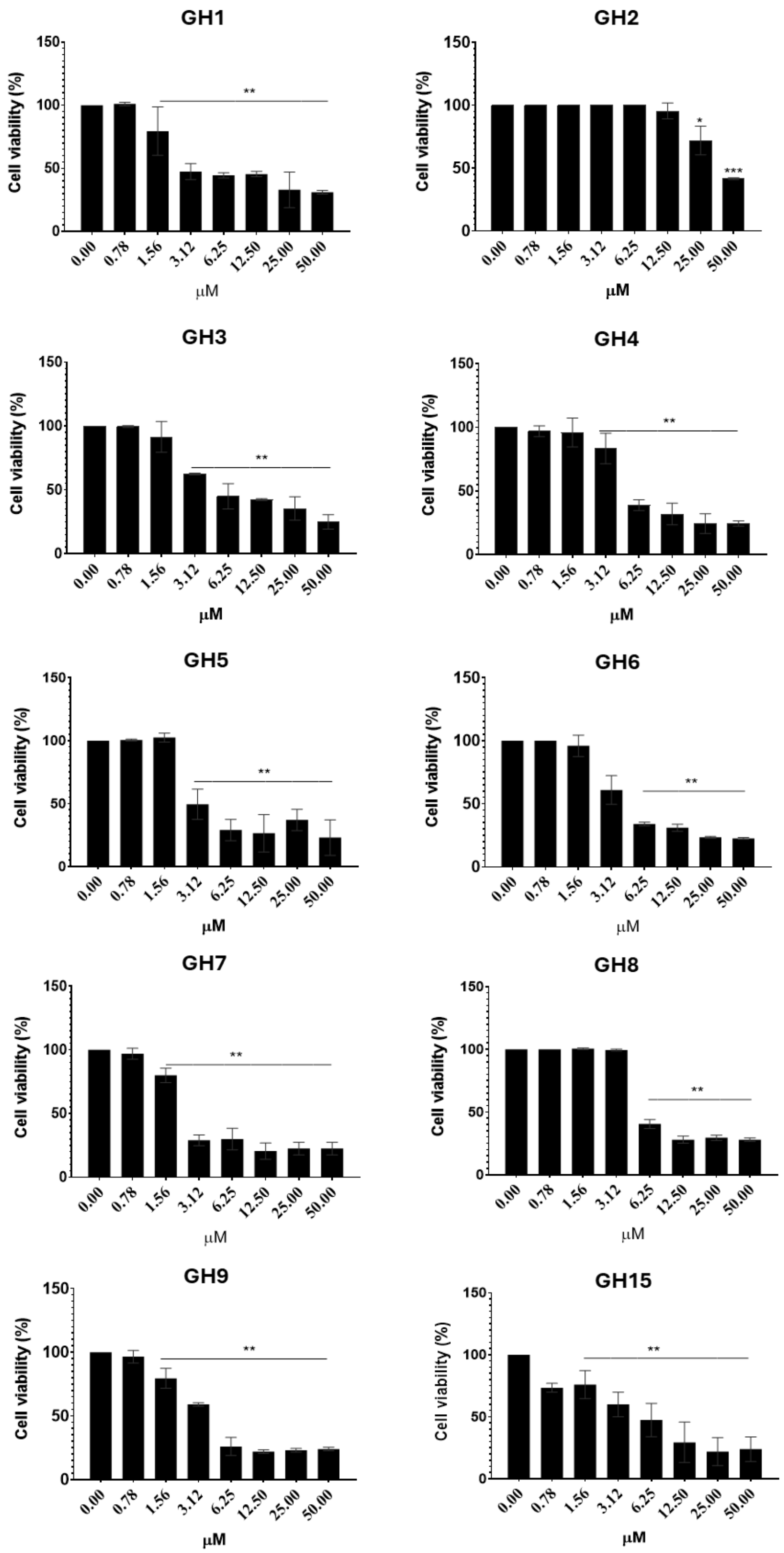

Figure S30. Effects of increasing concentrations (0-50 μM) of each investigated guanyl hydrazone derivative on the viability of MCF7 human cancer cells after 72 h of incubation. Cell viability values are reported as the percentage of viable treated cells with respect to control cells grown in the absence of the tested compounds. Three independent experiments were performed with triplicated determinations. * $P \leq 0.05$, ** $P \leq 0.01$ and *** $P \leq 0.001$ were obtained for treated *vs.* control samples.

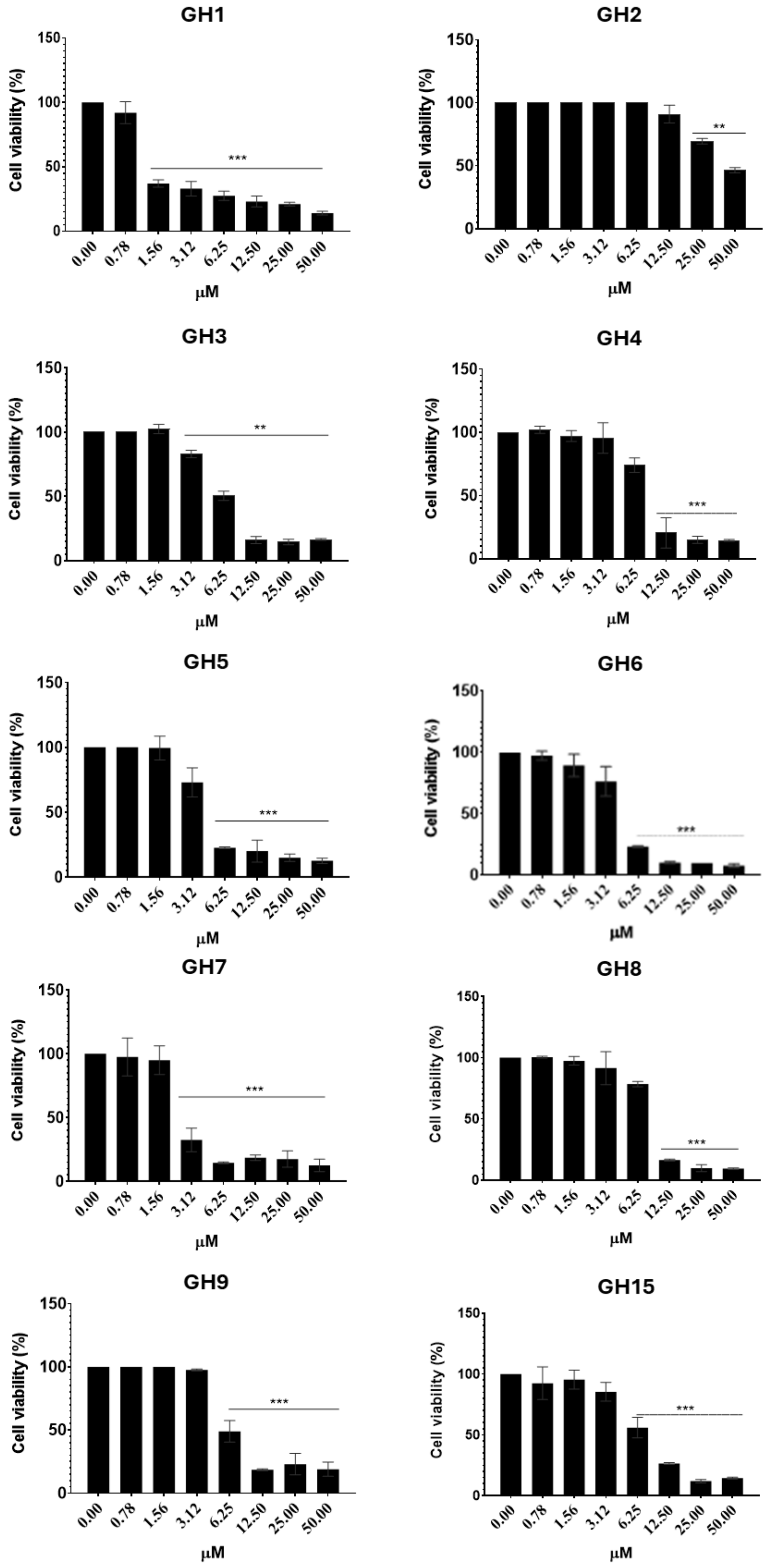

Figure S31. Effects of increasing concentrations (0-50 μM) of each investigated guanyl hydrazone derivative on the viability of HaCaT human normal cells after 72 h of incubation. Cell viability values are reported as the percentage of viable treated cells with respect to control cells grown in the absence of the tested compounds. Three independent experiments were performed with triplicated determinations. ** $P \leq 0.01$ and *** $P \leq 0.001$ were obtained for treated *vs.* control samples.

^{1}H-NMR (400 MHz, CDCl₃) of 1

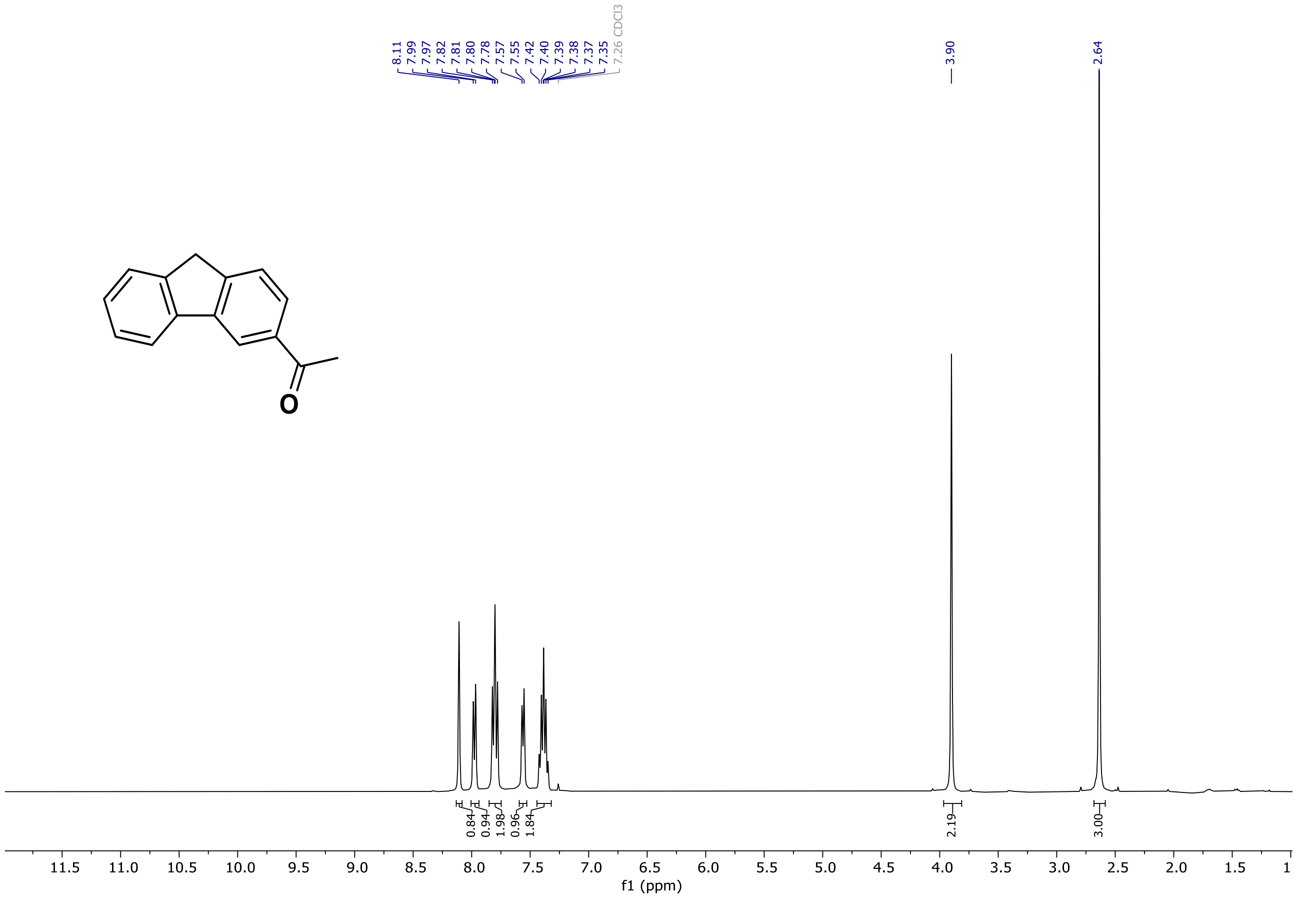

^{1}H-NMR (400 MHz, DMSO-d_6) of GH1

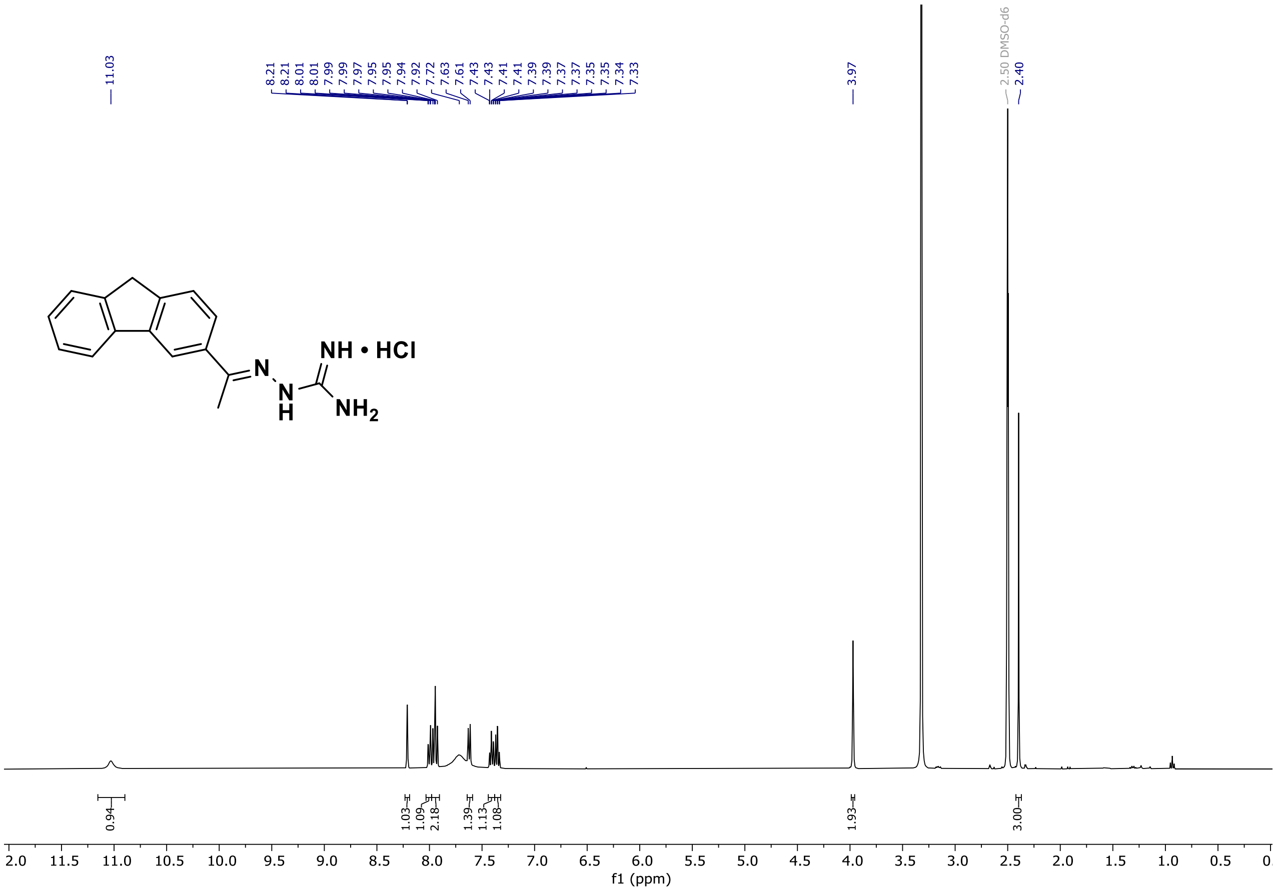

^{13}C-NMR (101 MHz, DMSO-d_6) of GH1

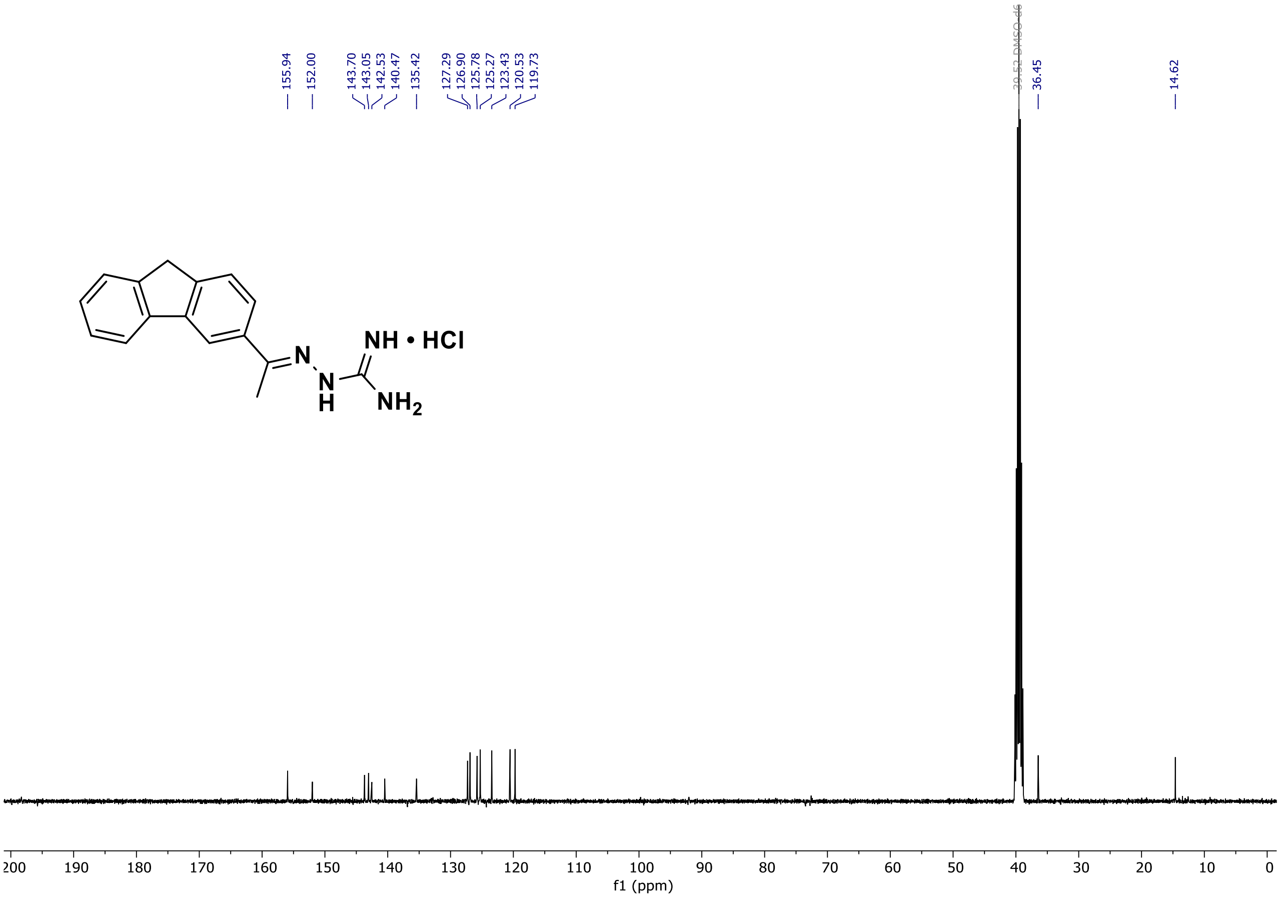

^{1}H-NMR (400 MHz, CDCl3) of 2

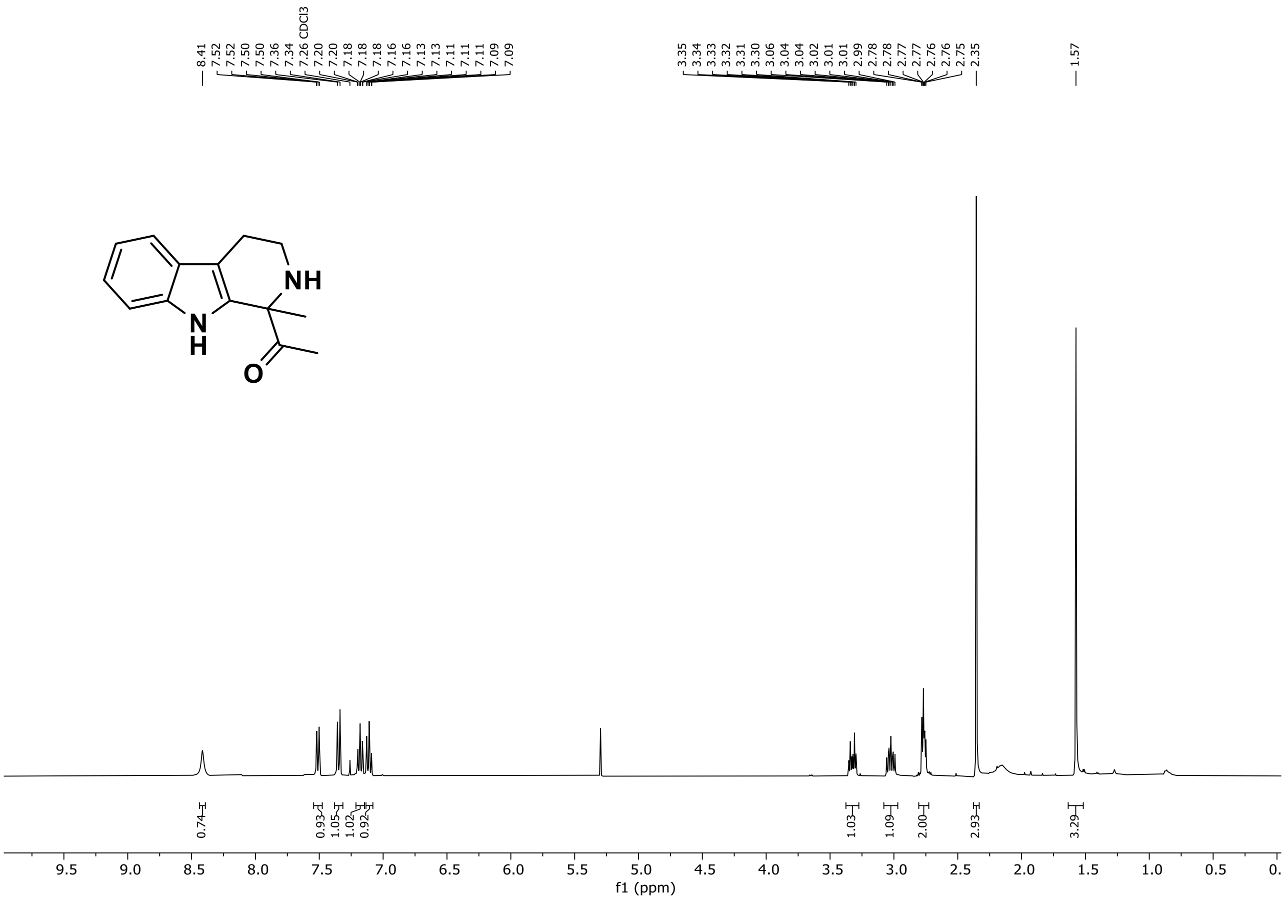

^{1}H-NMR (400 MHz, DMSO-d_6) of GH2

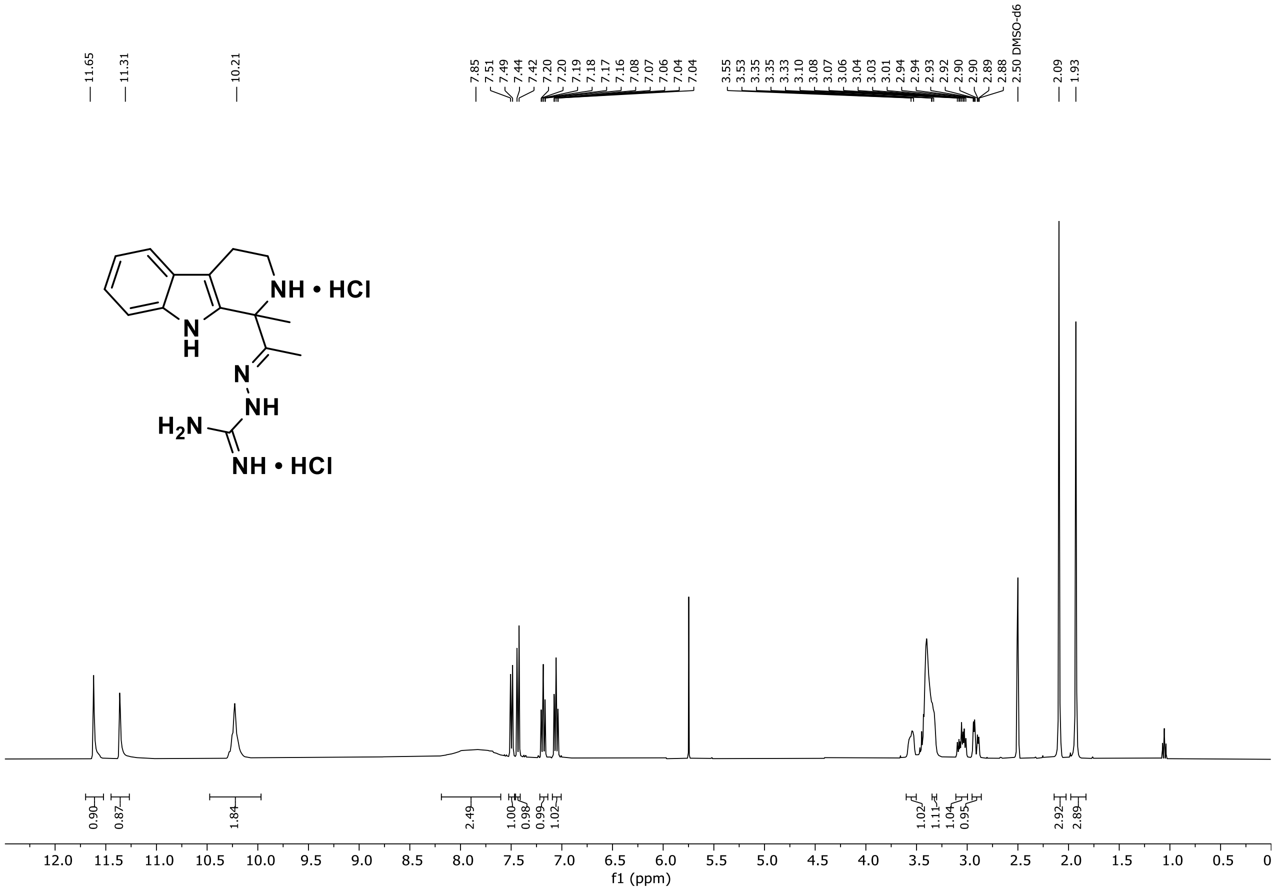

^{13}C-NMR (101 MHz, DMSO-d_6) of GH2

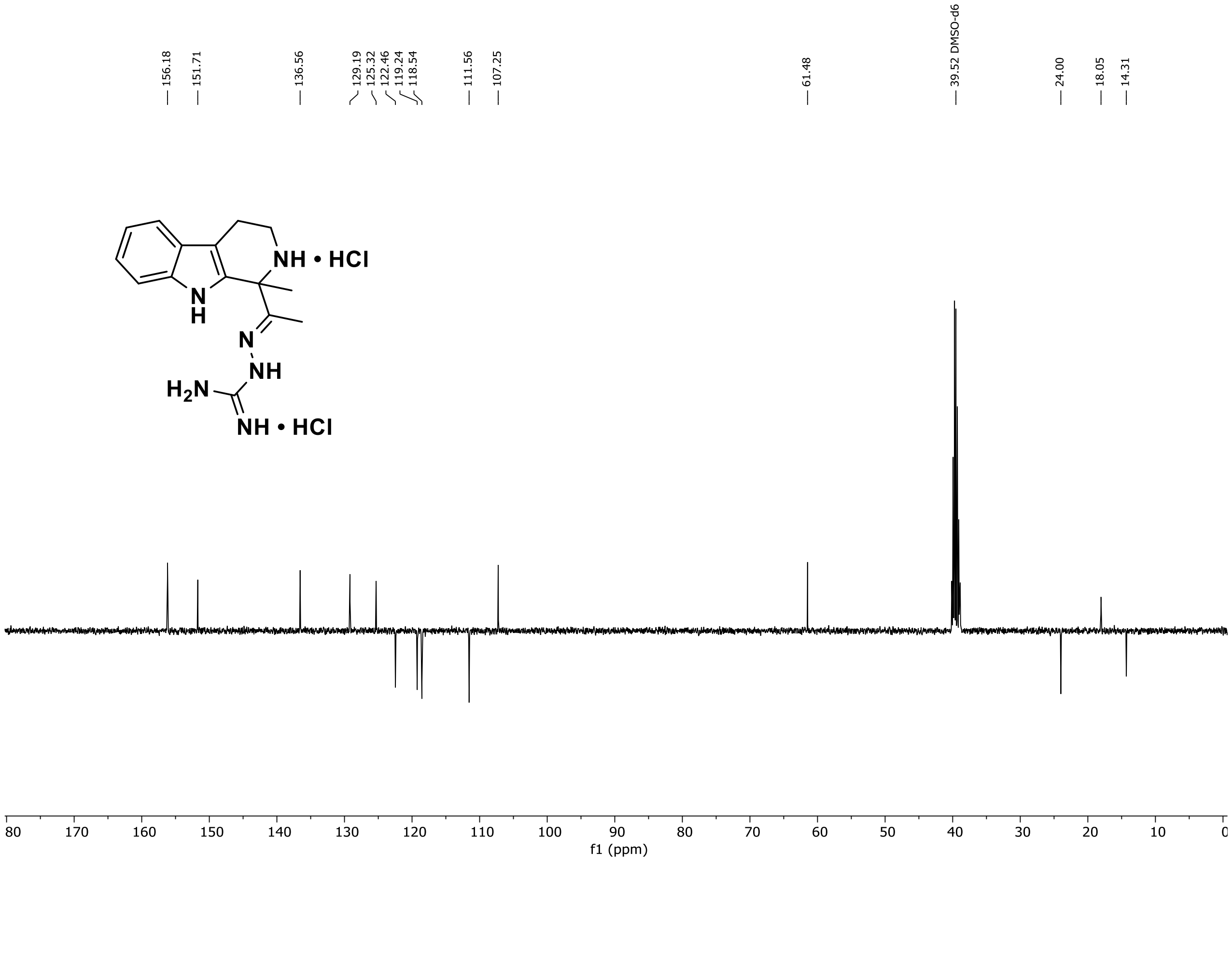

^{1}H-NMR (400 MHz, CDCl₃) of 3

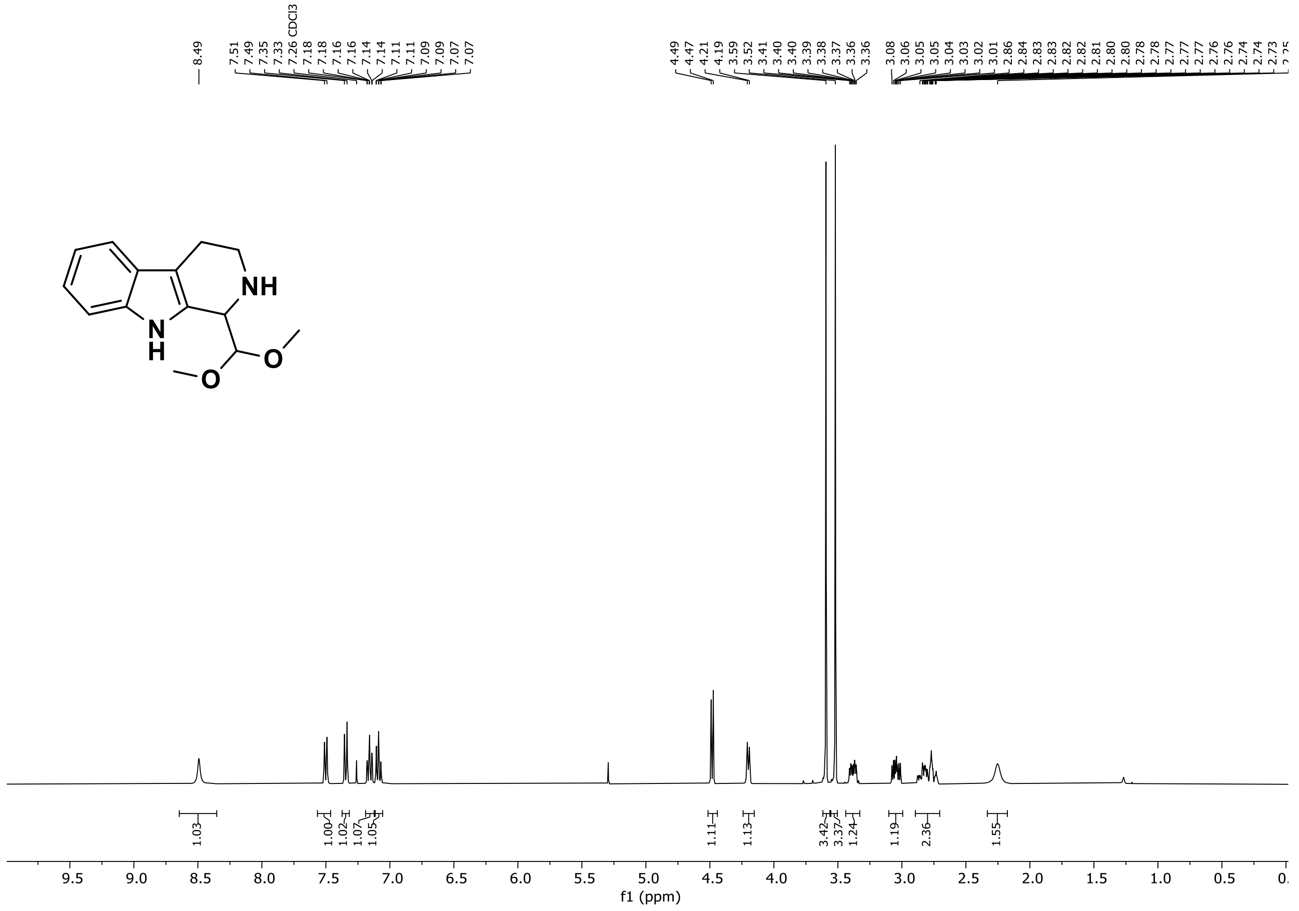

^{1}H-NMR (400 MHz, $CDCl_3$) of 4

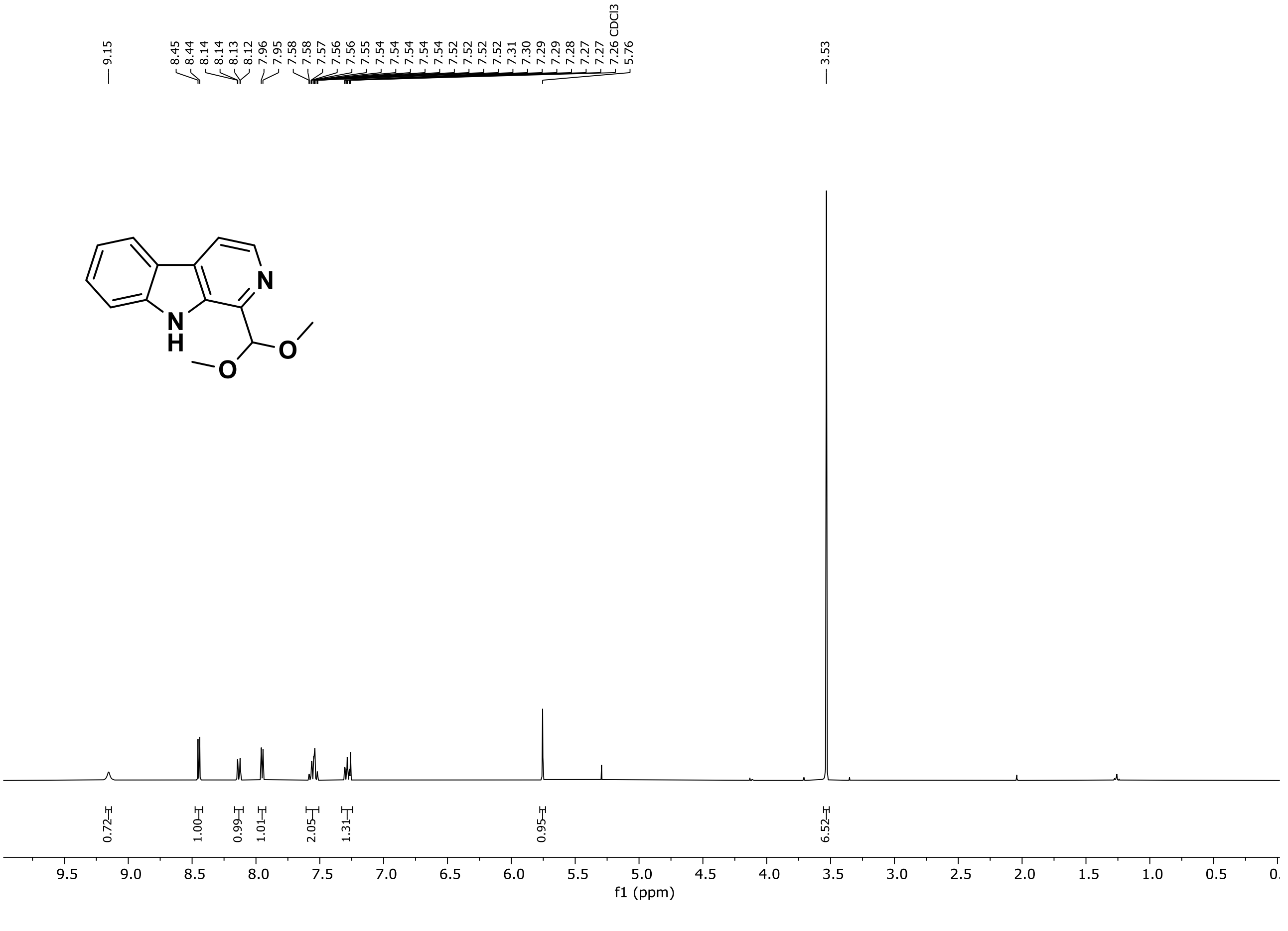

^{1}H-NMR (400 MHz, $CDCl_3$) of 5

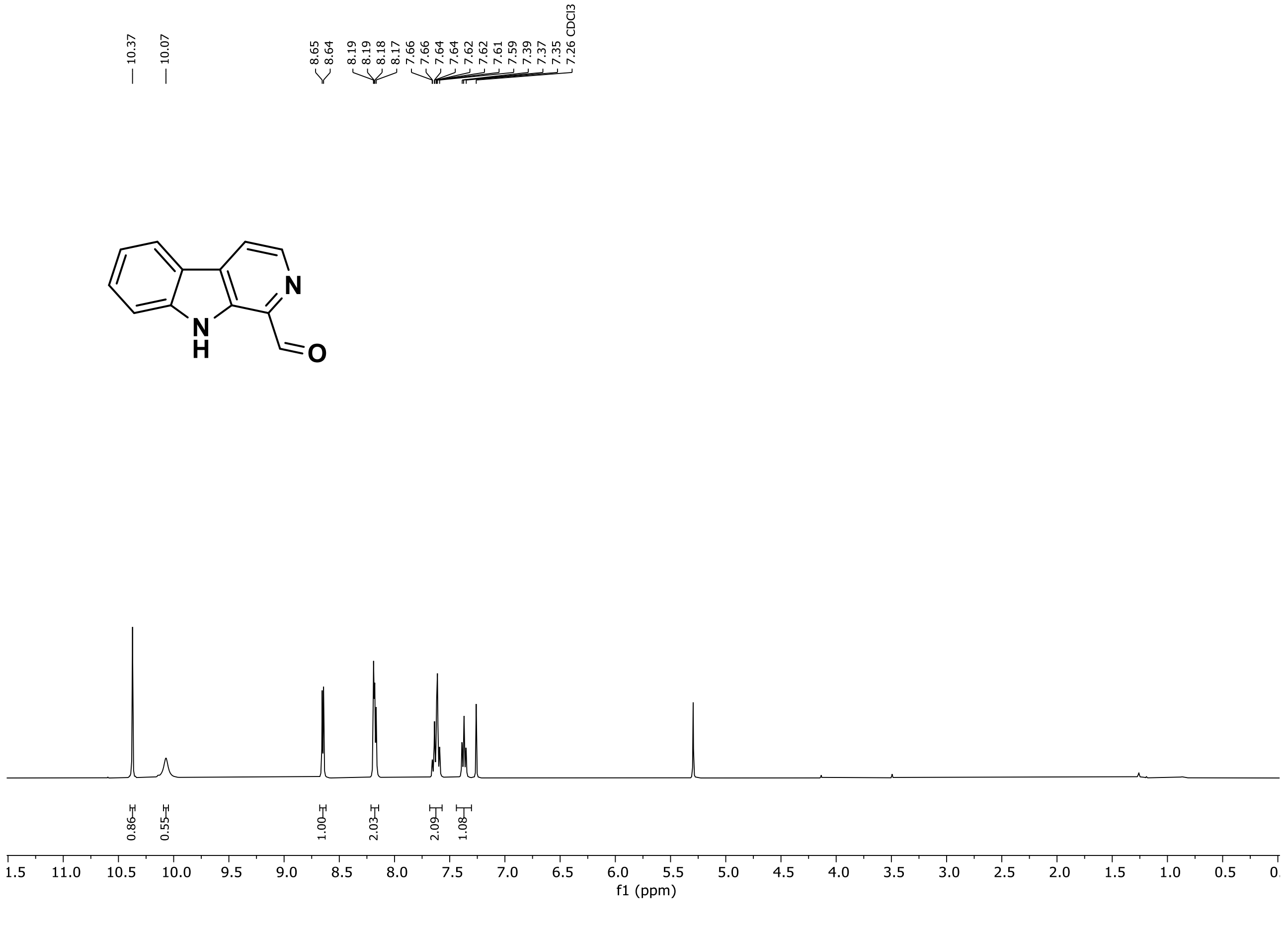

^{1}H-NMR (400 MHz, DMSO-d_6) of GH3

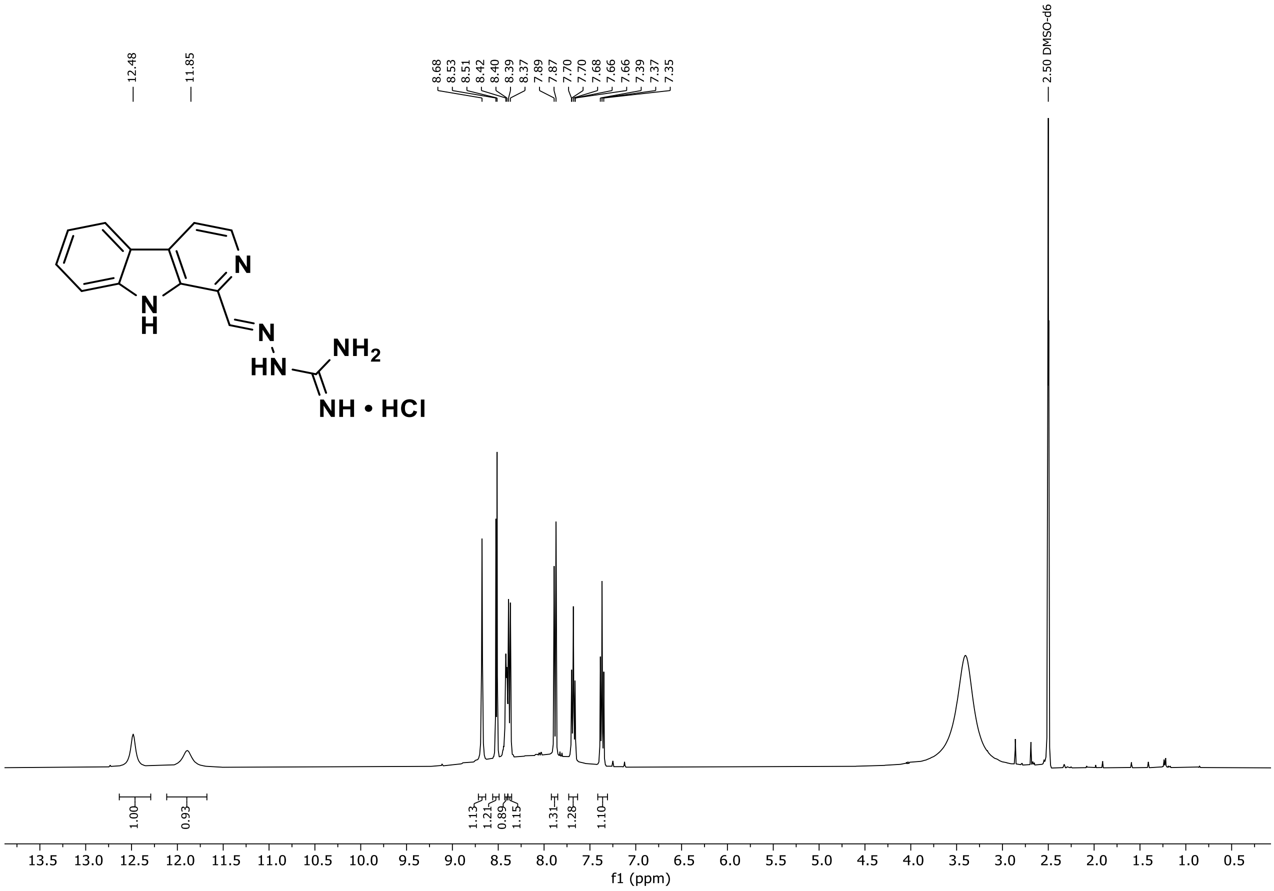

^{13}C-NMR (101 MHz, DMSO-d_6) of GH3

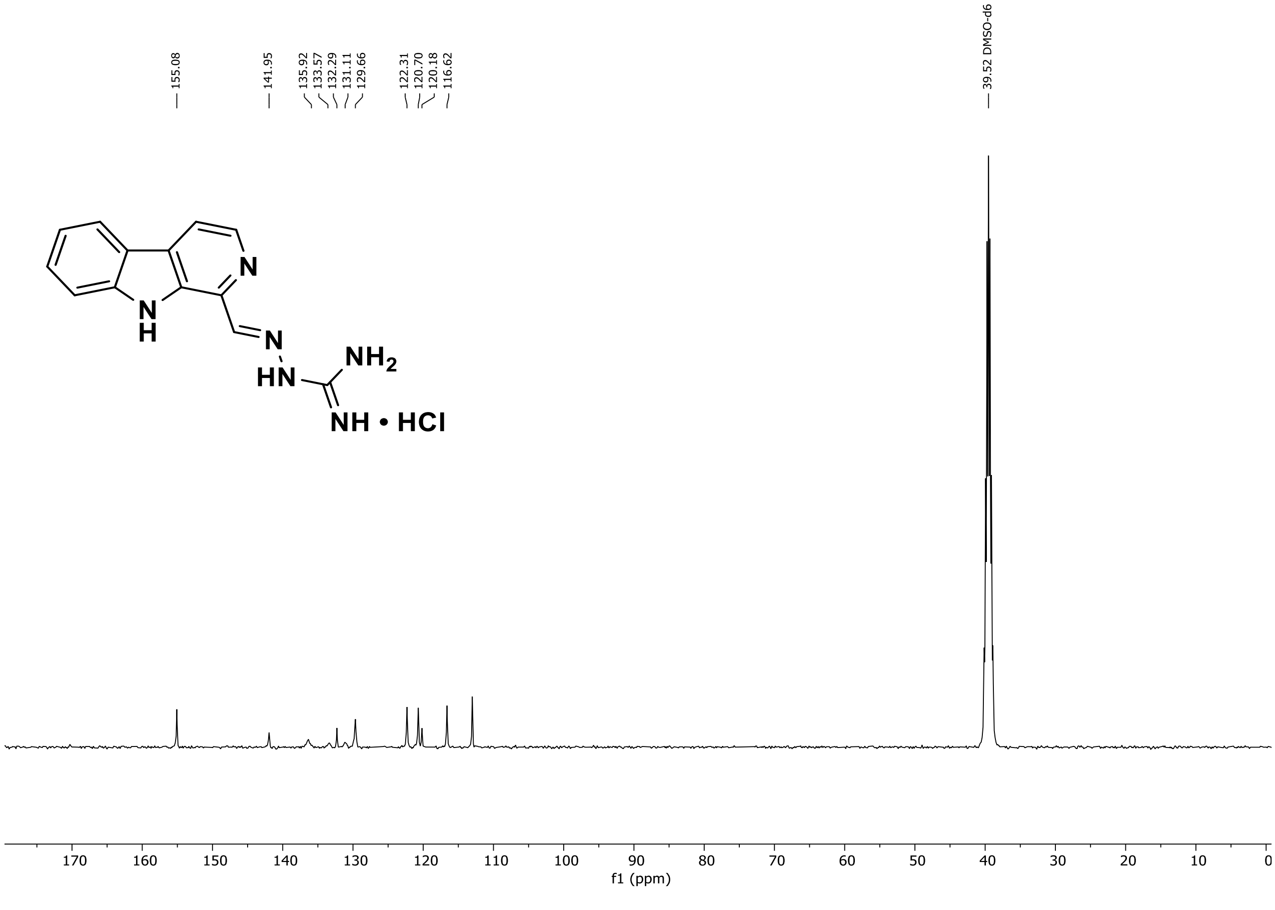

^{1}H-NMR (400 MHz, $CDCl_3$) of 6

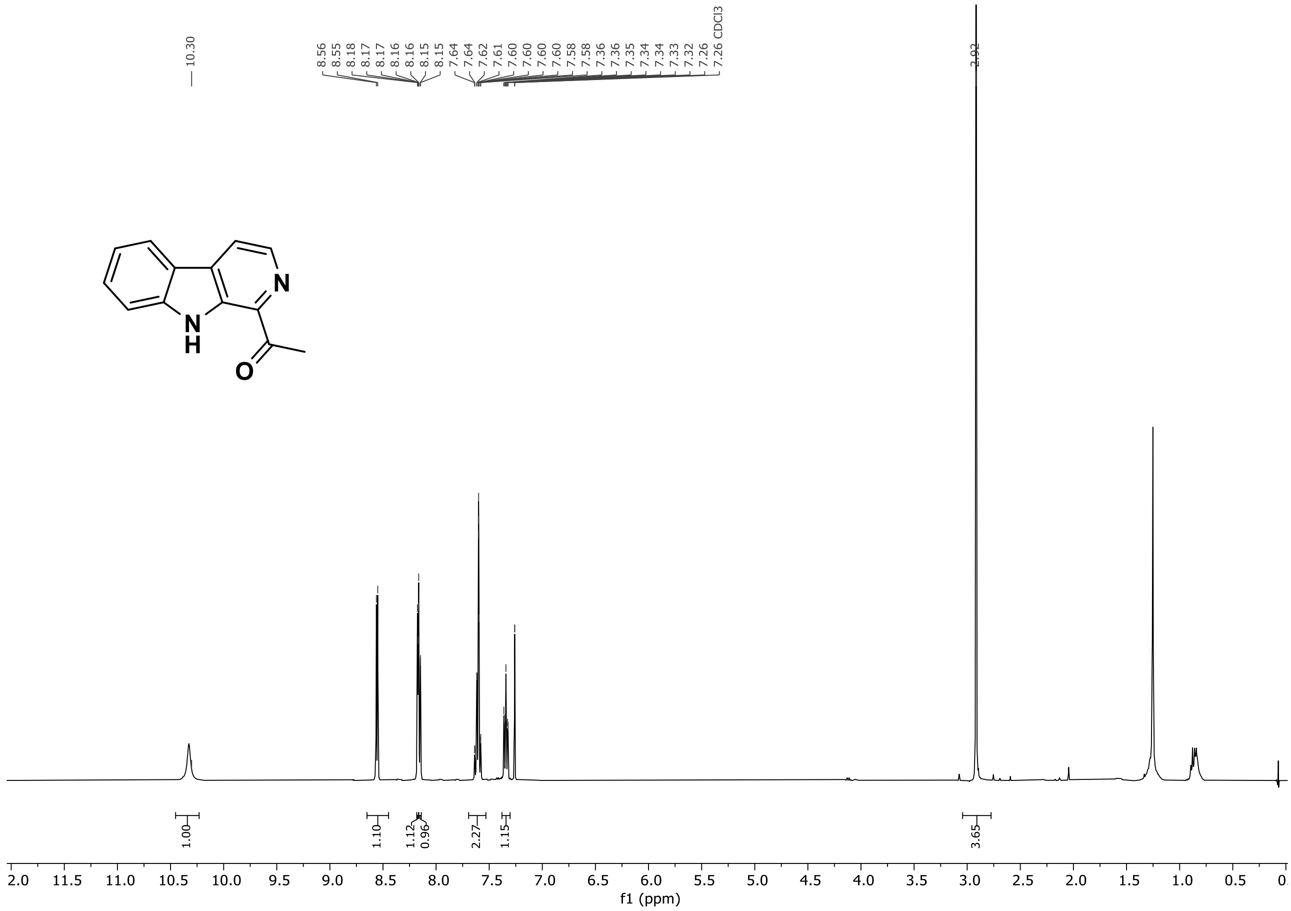

^{1}H-NMR (400 MHz, DMSO-d_6) of GH4

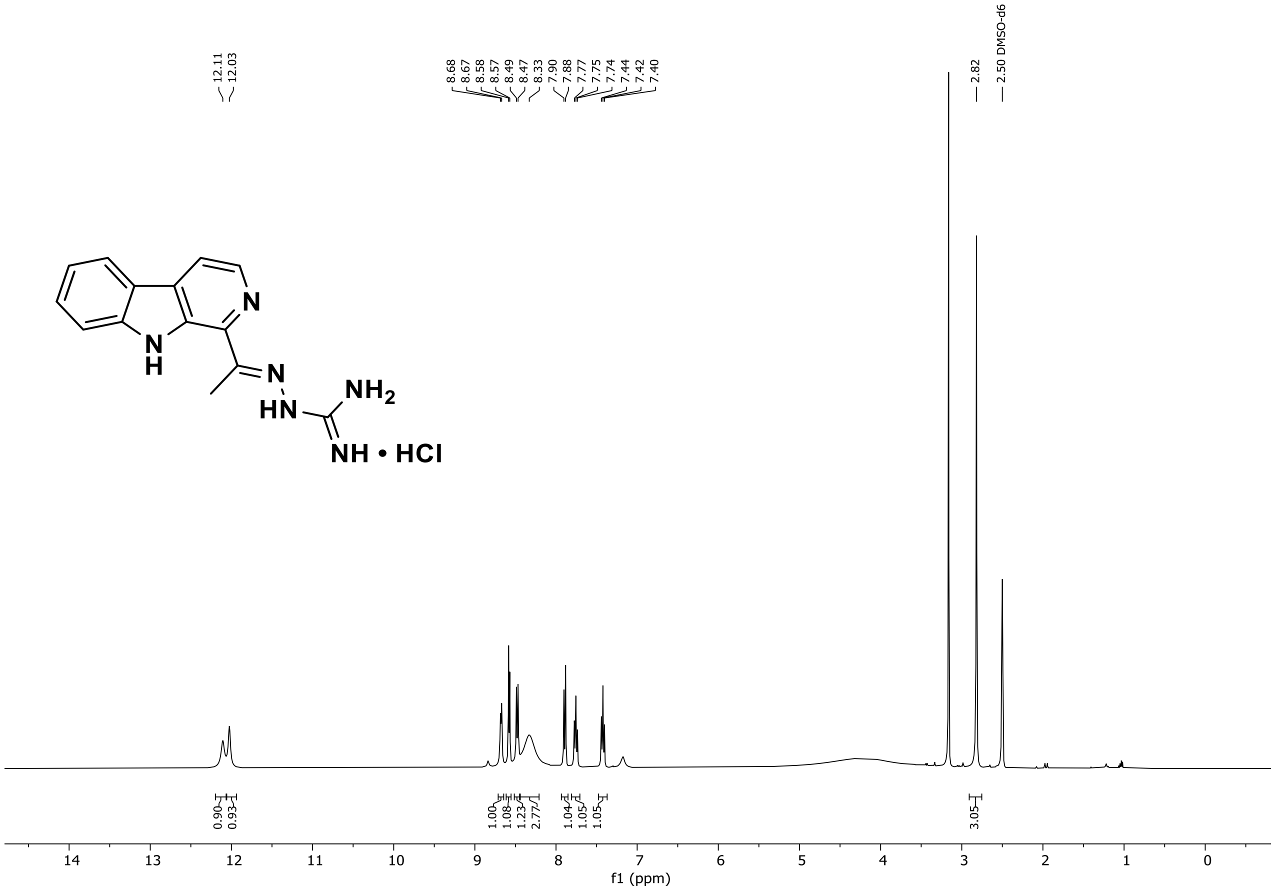

^{13}C-NMR (101 MHz, DMSO-d_6) of GH4

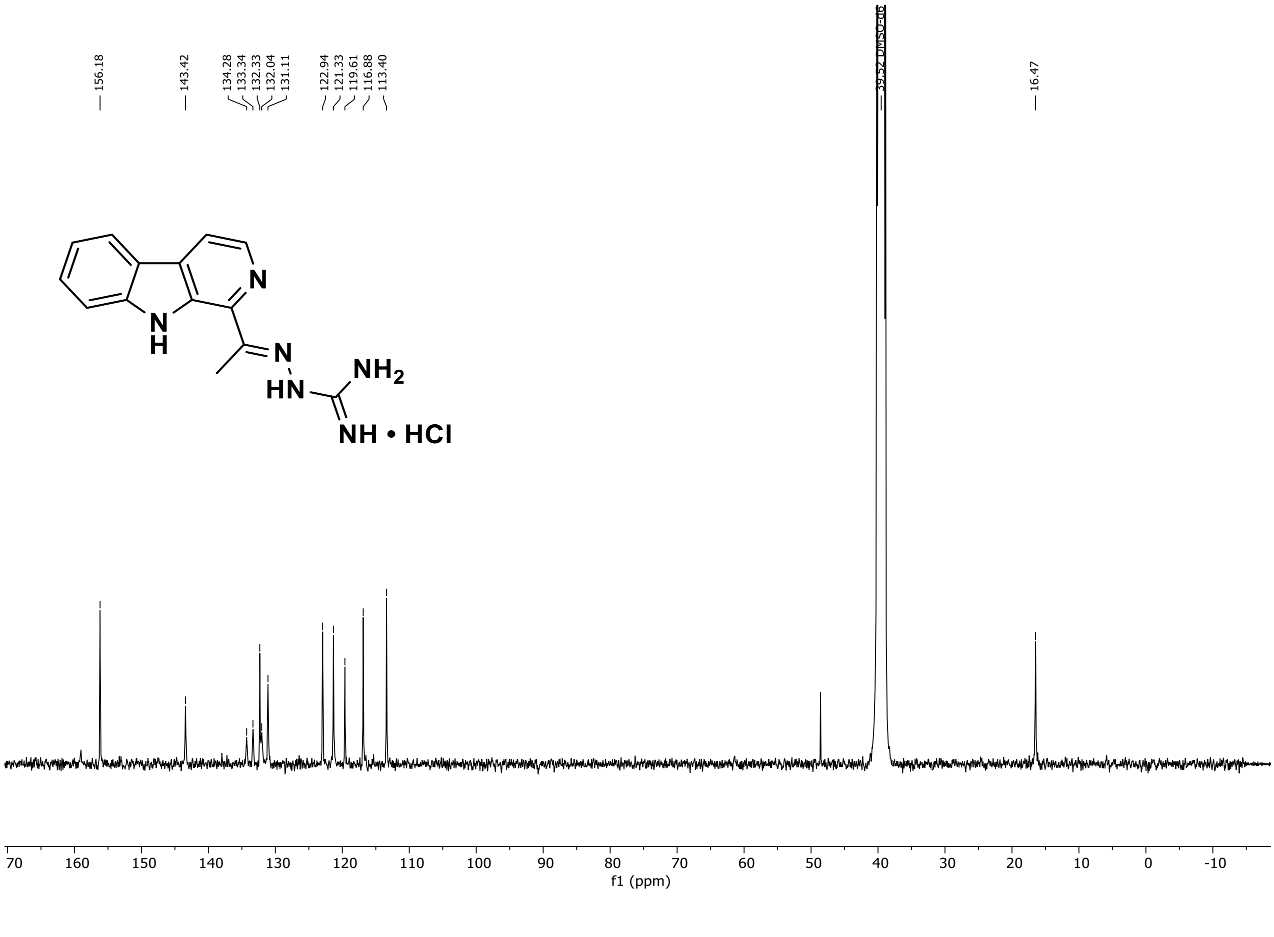

^{1}H-NMR (400 MHz, DMSO-*d₆*) of GH5

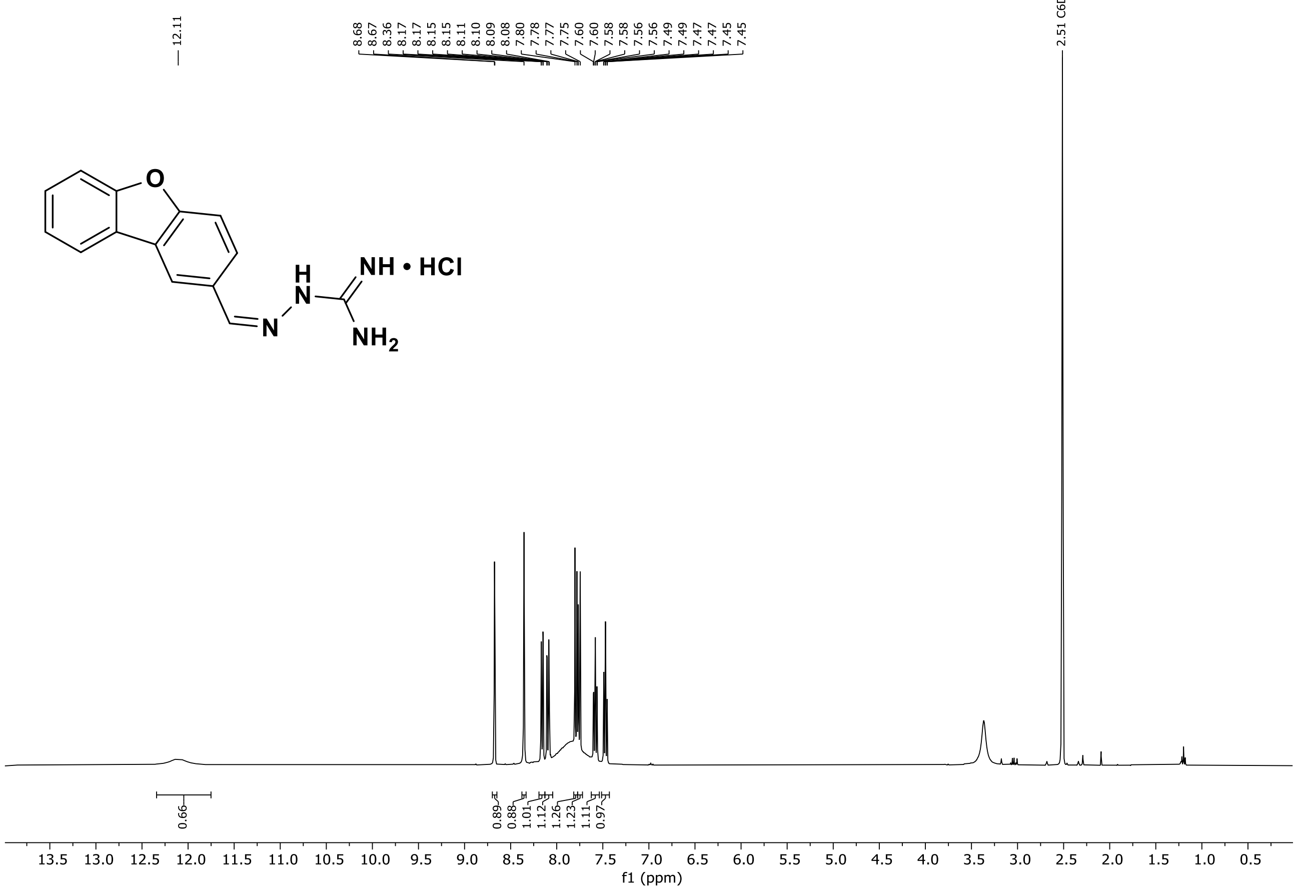

^{13}C-NMR (101 MHz, DMSO-*d₆*) of GH5

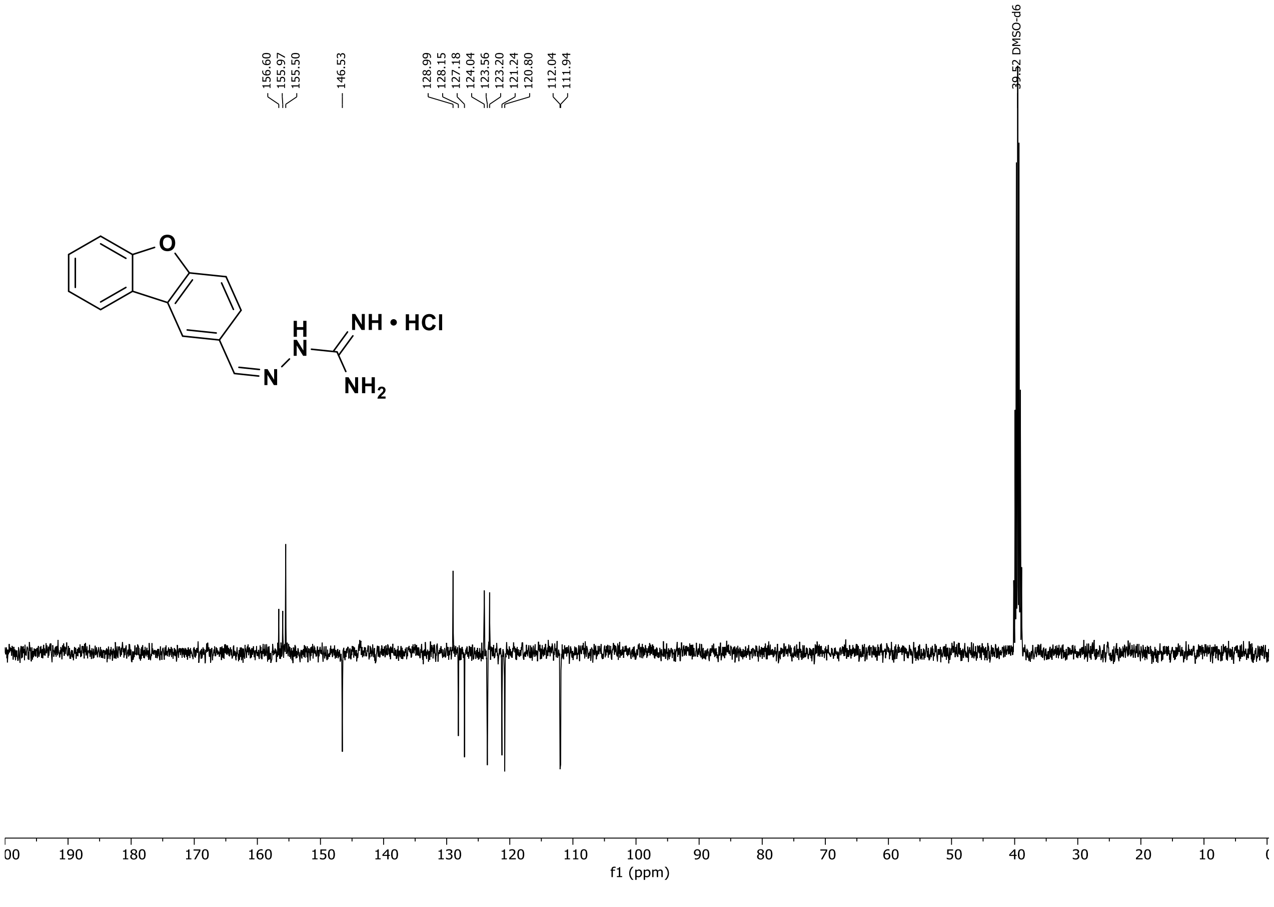

^{1}H-NMR (400 MHz, $CDCl_3$) of 7

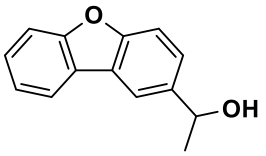

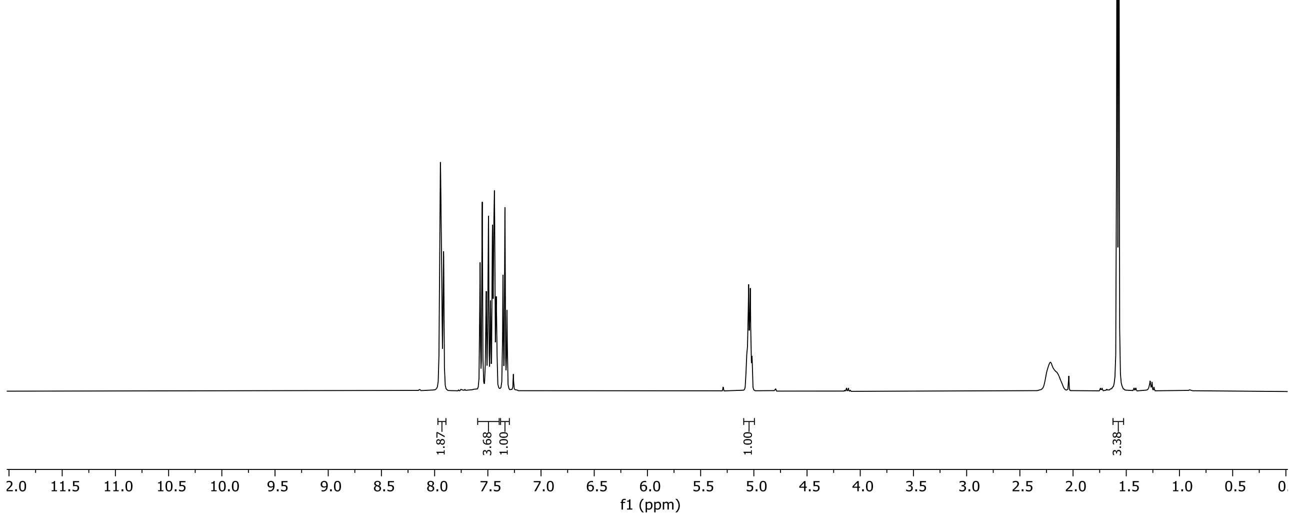

^{1}H-NMR (400 MHz, $CDCl_3$) of 8

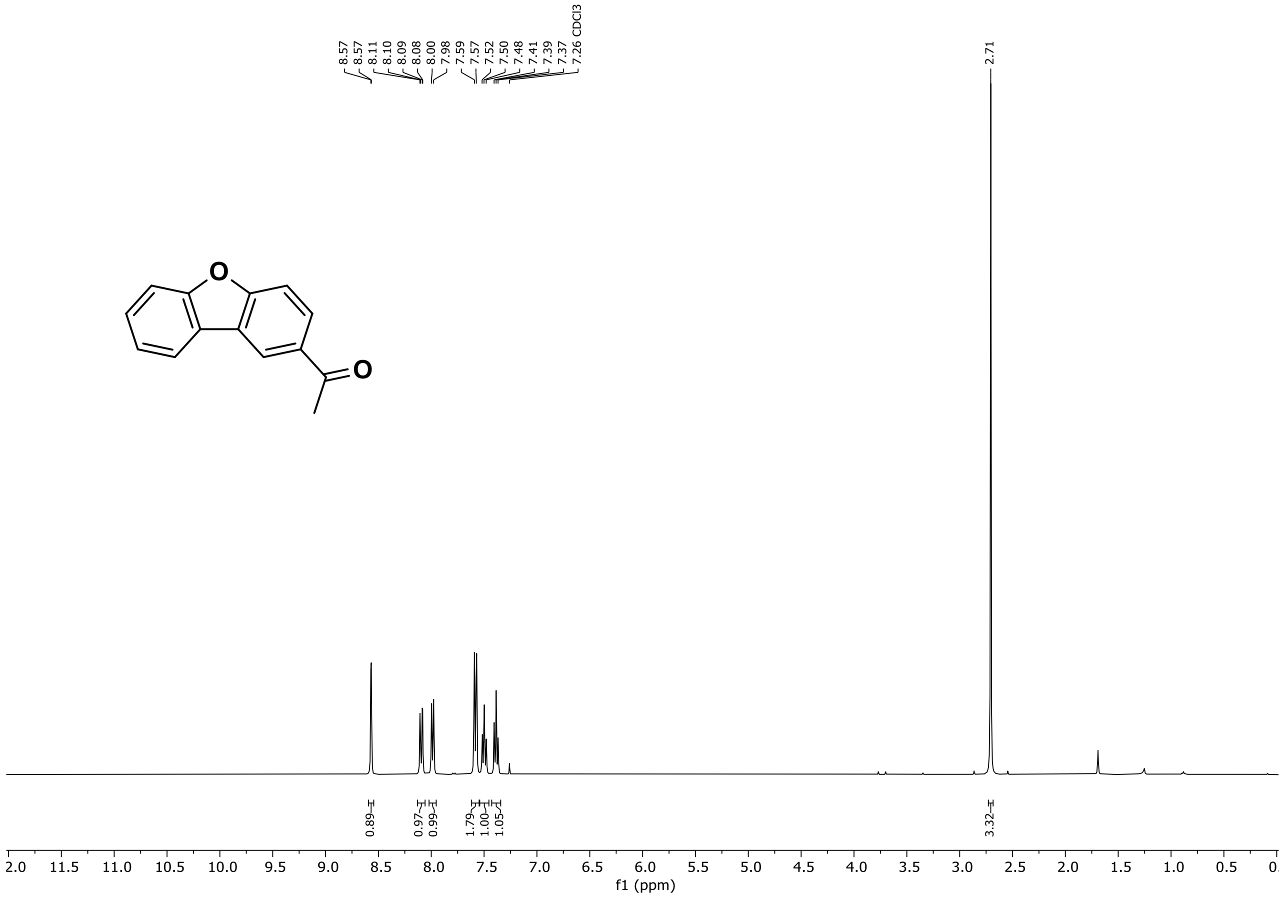

^{1}H-NMR (400 MHz, DMSO-d_6) of GH6

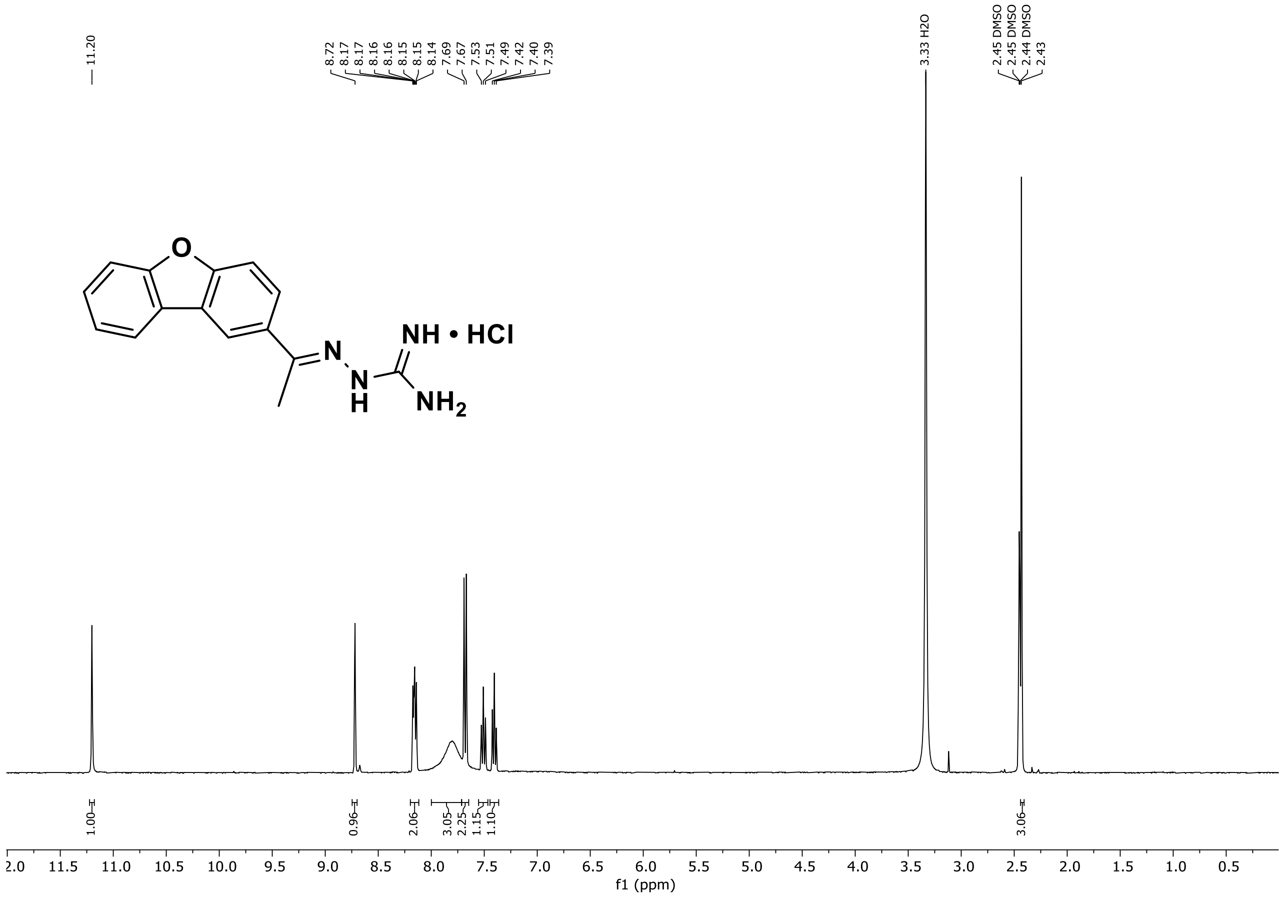

^{13}C-NMR (101 MHz, DMSO-d_6) of GH6

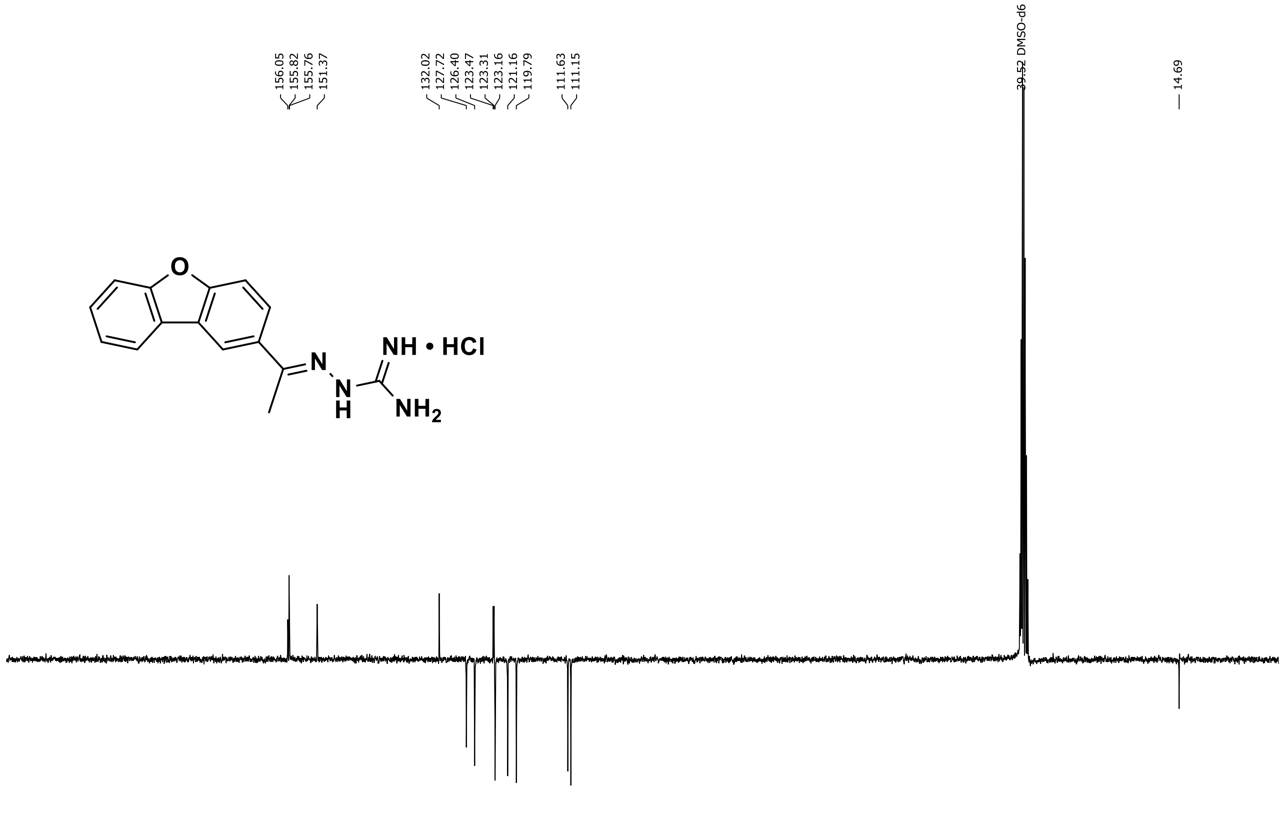

^{1}H-NMR (400 MHz, DMSO-d_6) of GH7

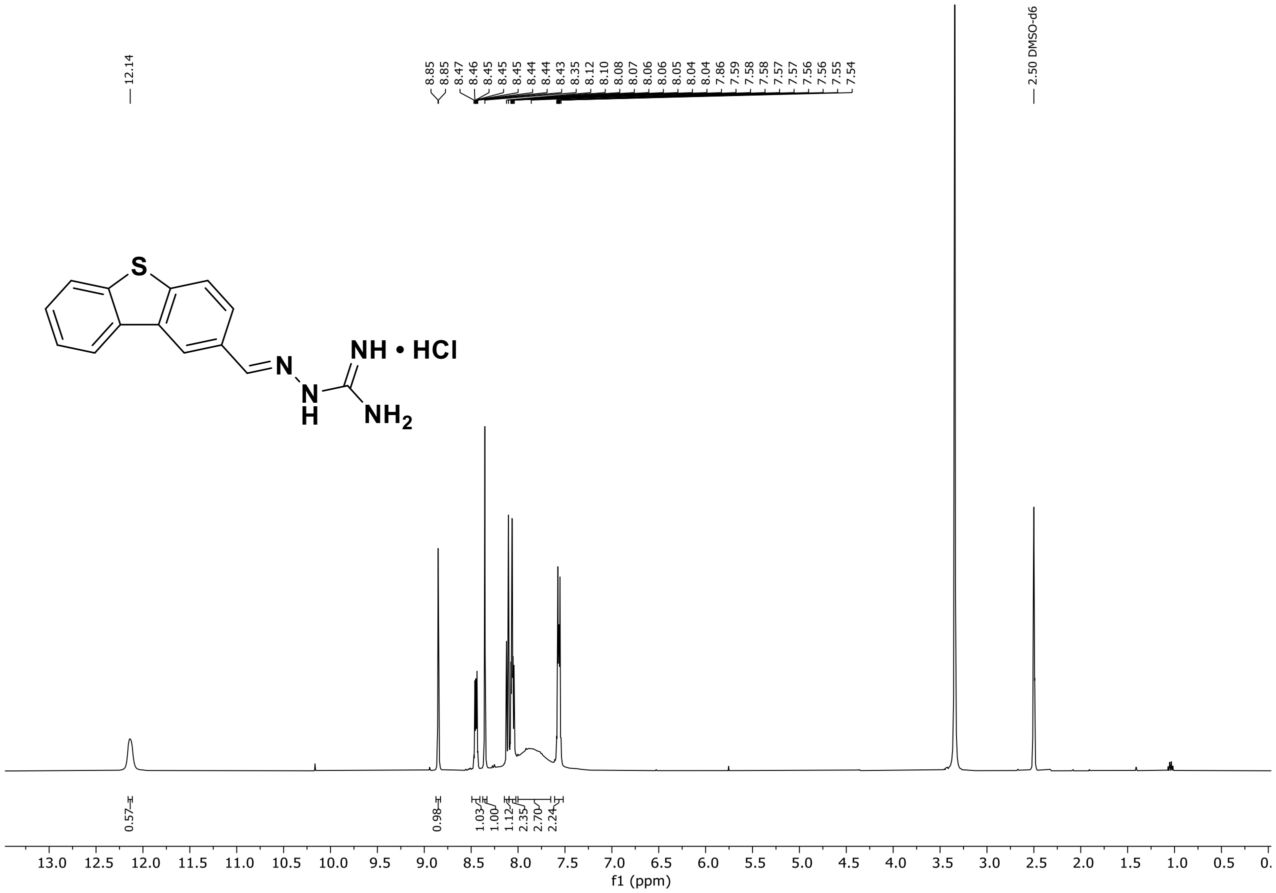

^{13}C-NMR (101 MHz, DMSO-d_6) of GH7

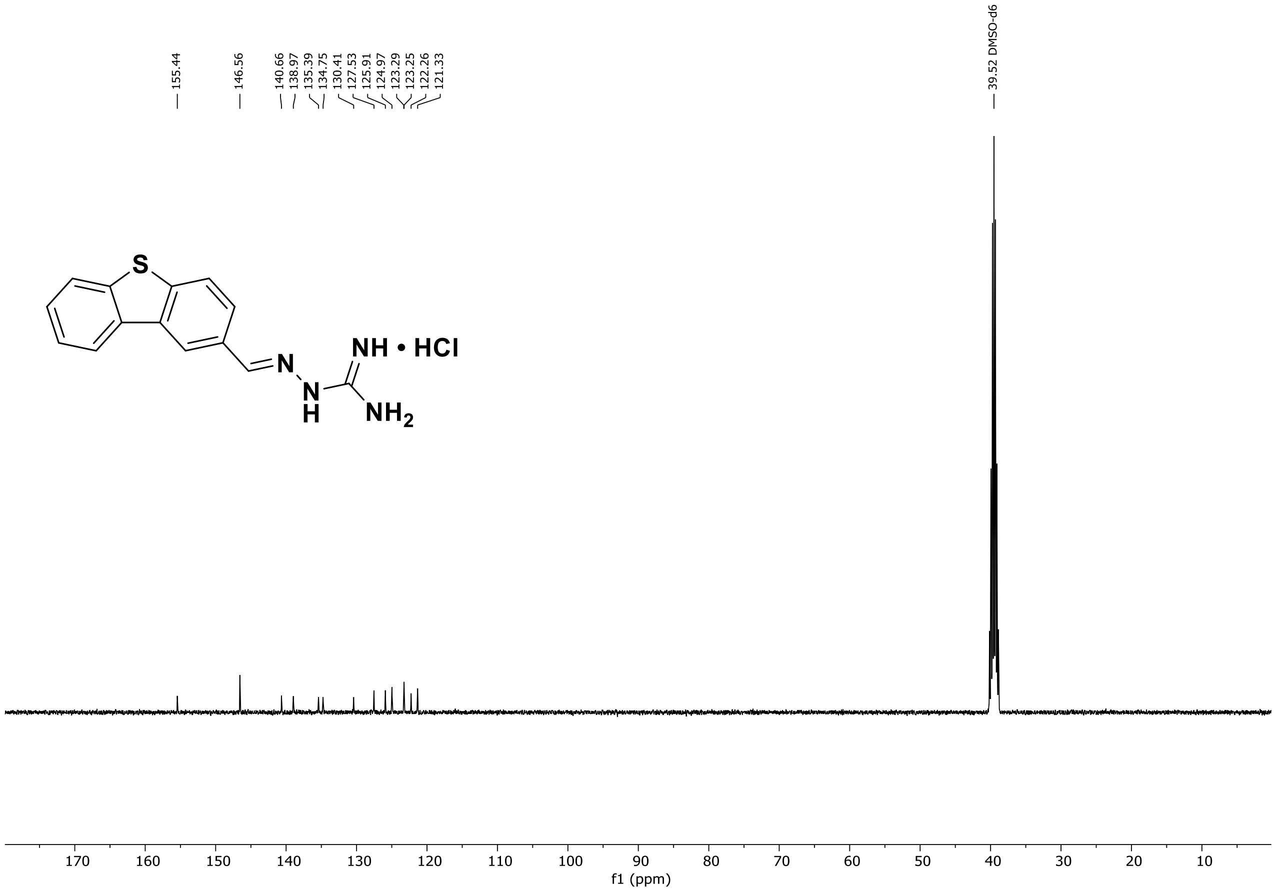

^{1}H-NMR (400 MHz, $CDCl_3$) of 9

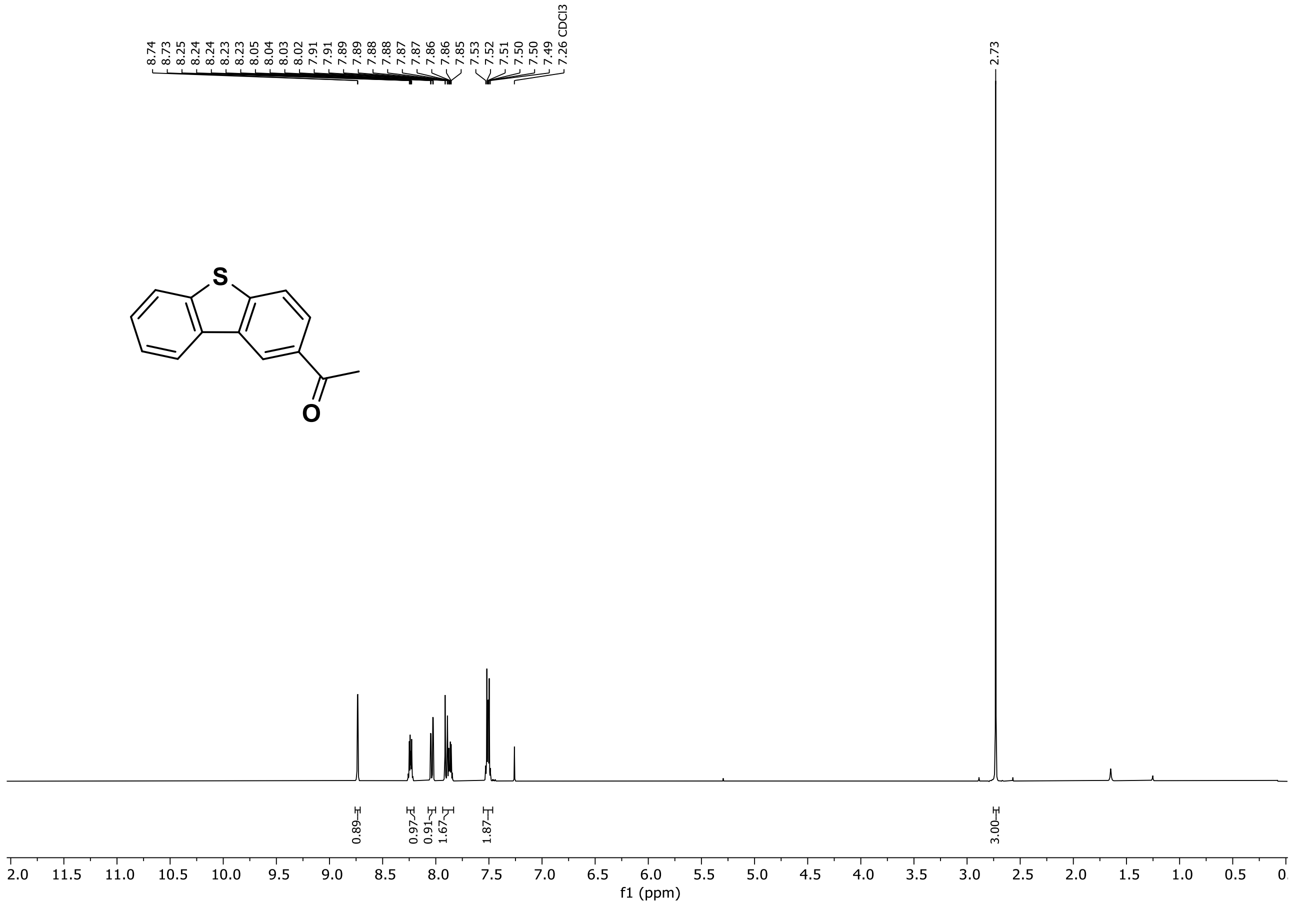

^{1}H-NMR (400 MHz, CDCl3) of 10

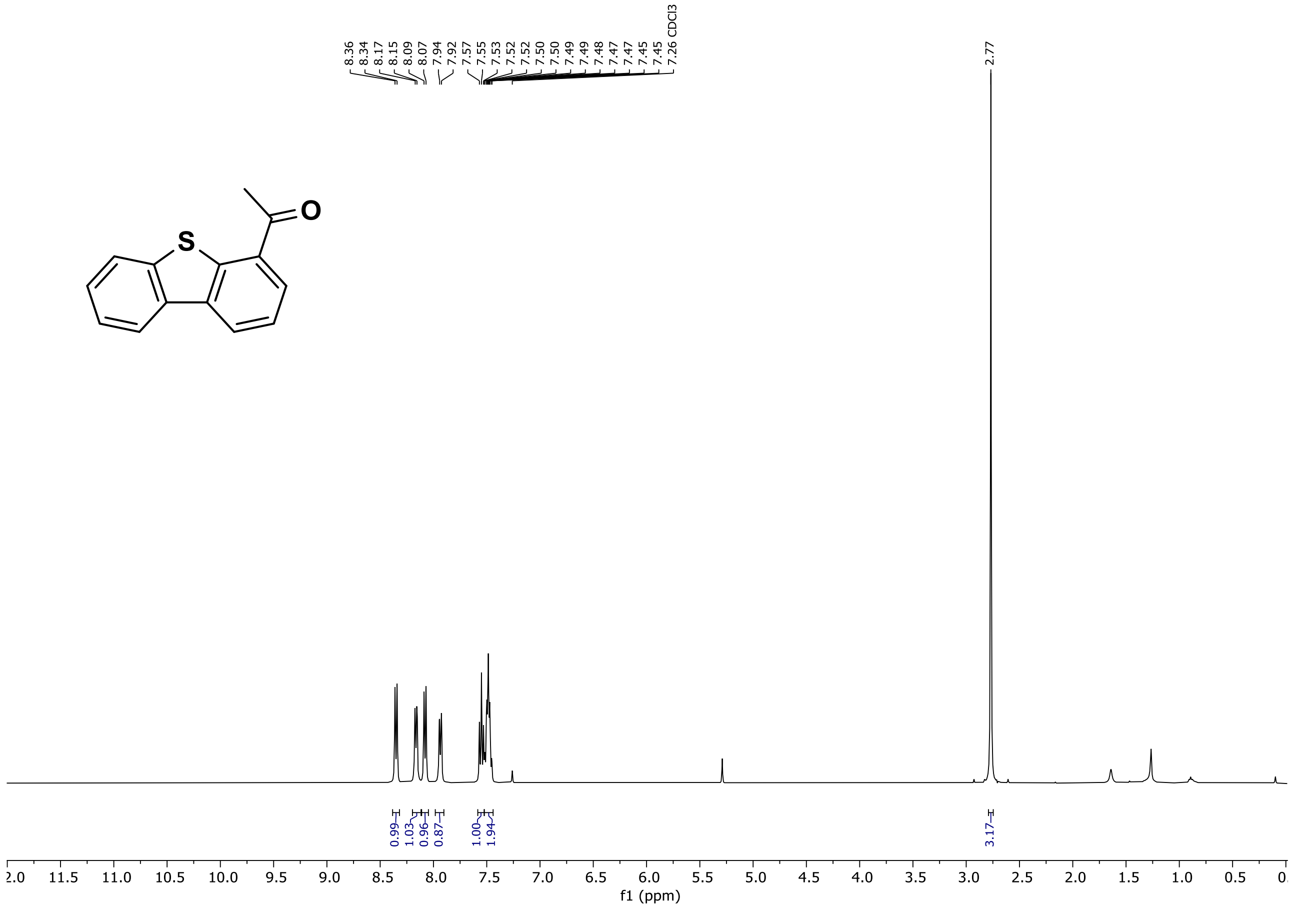

^{1}H-NMR (400 MHz, DMSO-d_6) of GH8

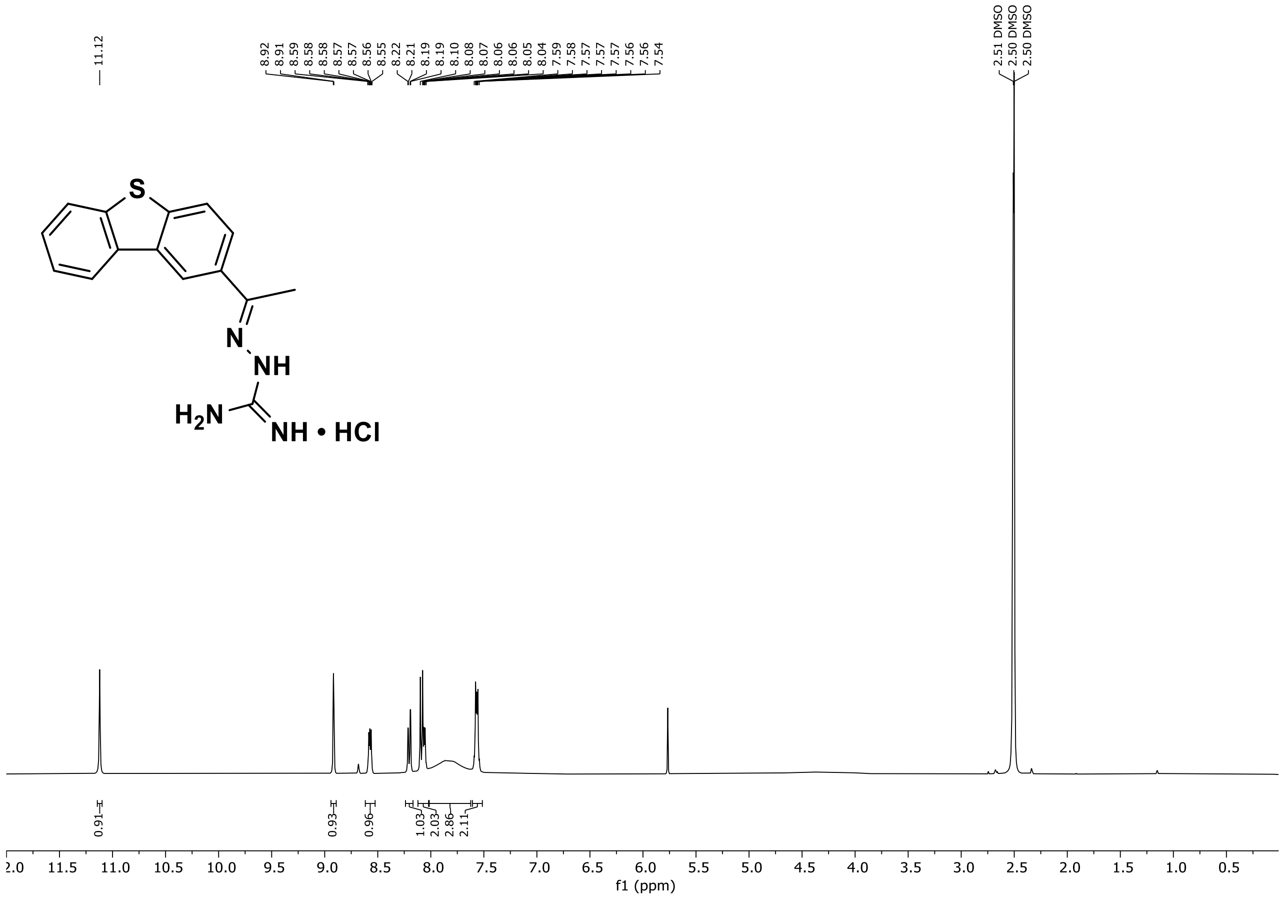

^{13}C-NMR (101 MHz, DMSO-d_6) of GH8

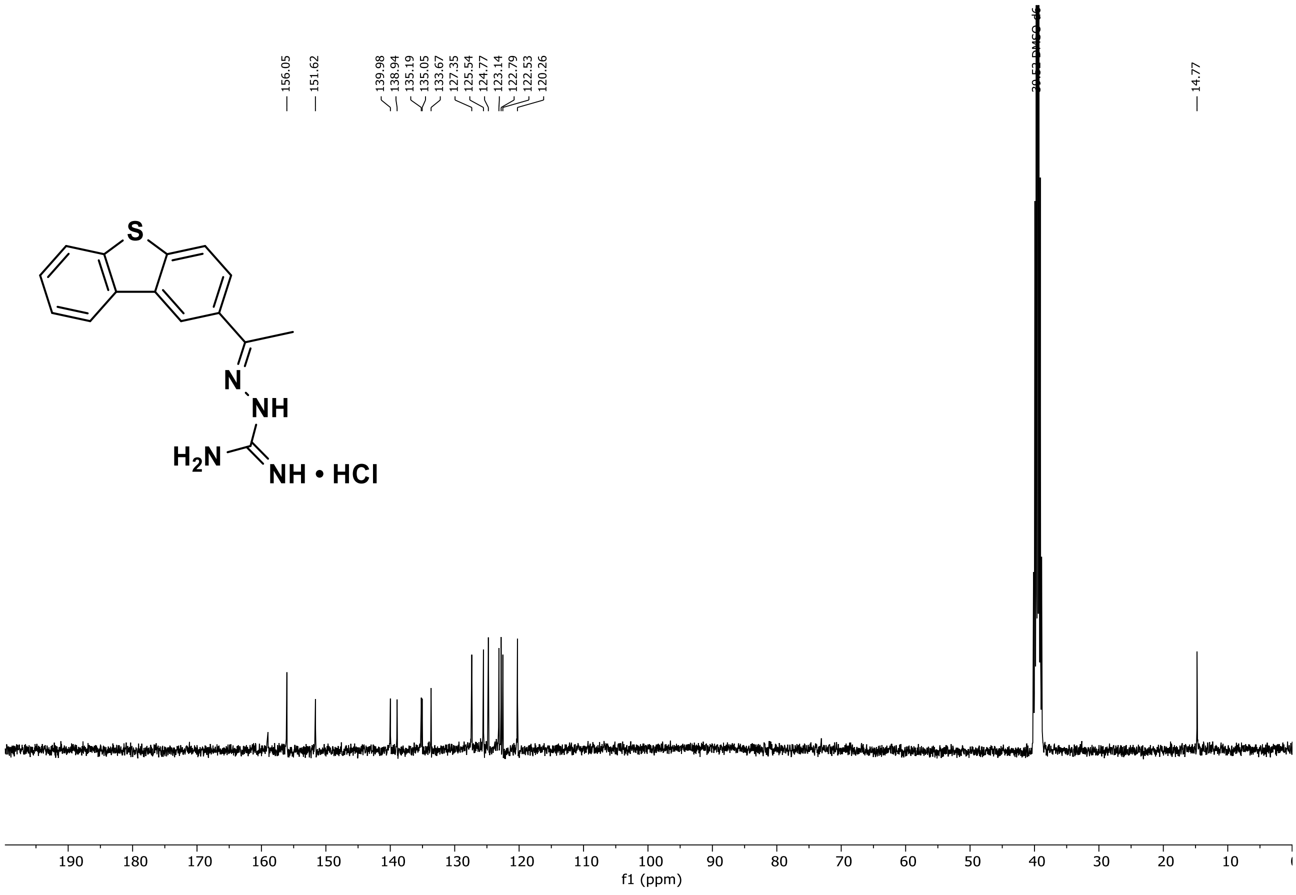

^{1}H-NMR (400 MHz, DMSO-d_6) of GH9

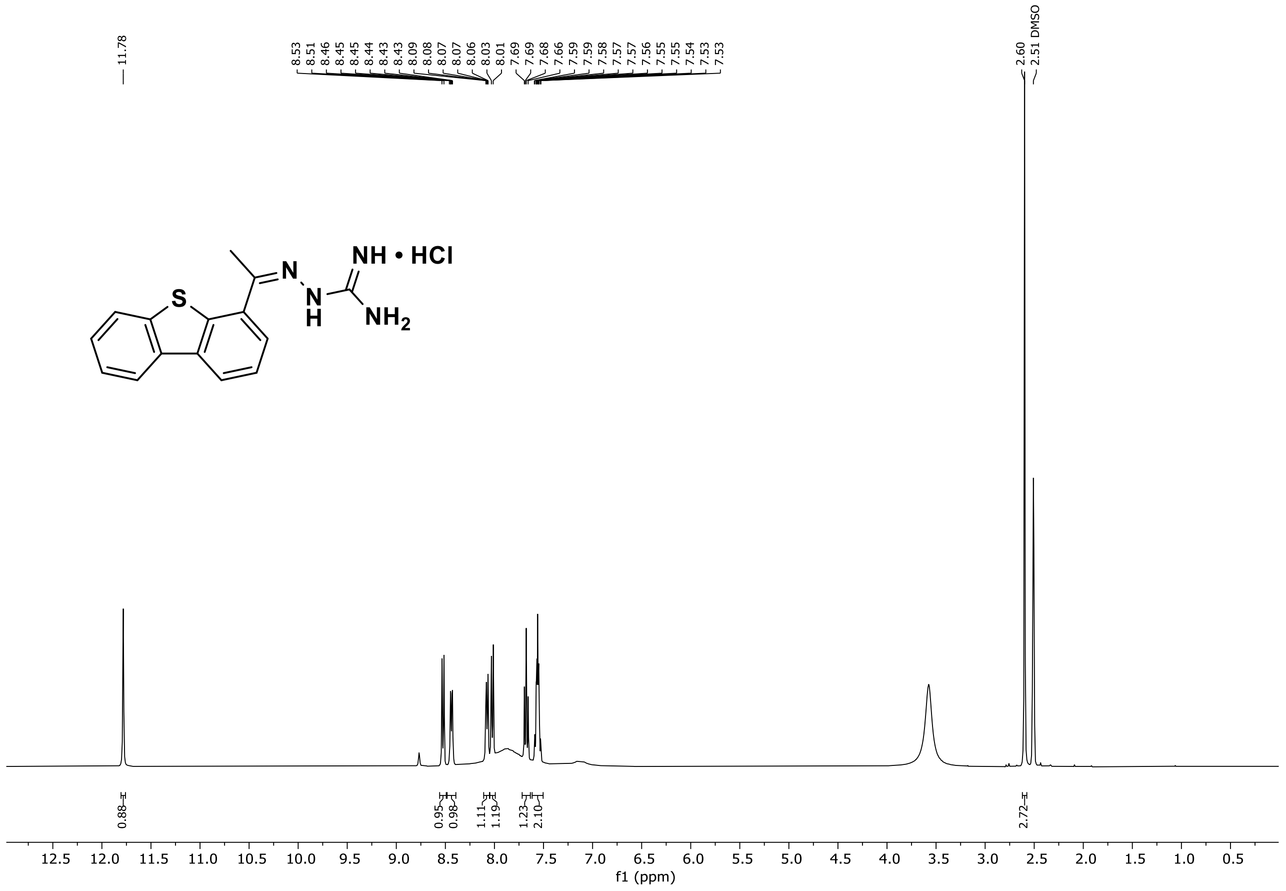

^{13}C-NMR (101 MHz, DMSO-d_6) of GH9

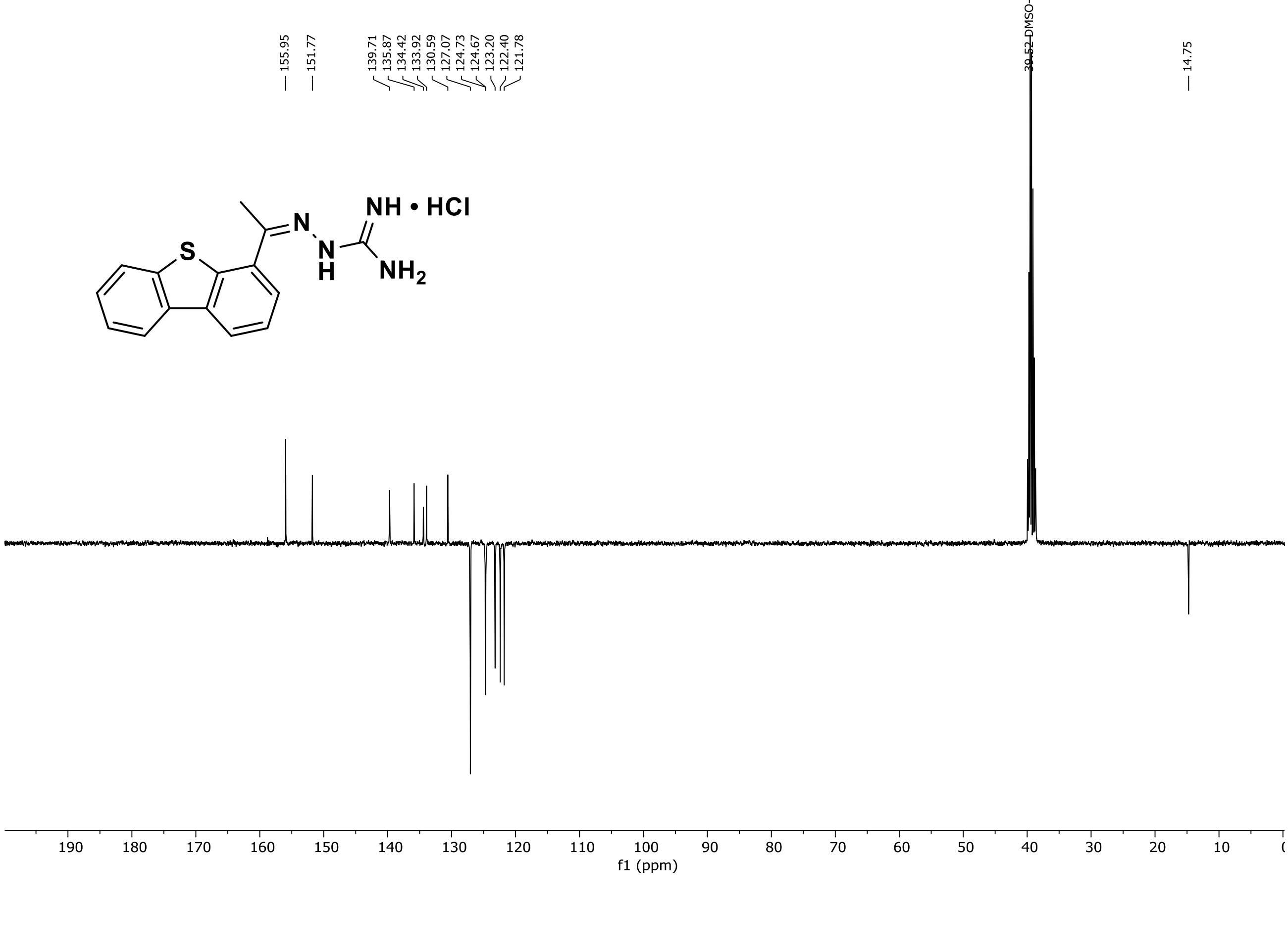

^{1}H-NMR (400 MHz, CDCl3) of 11

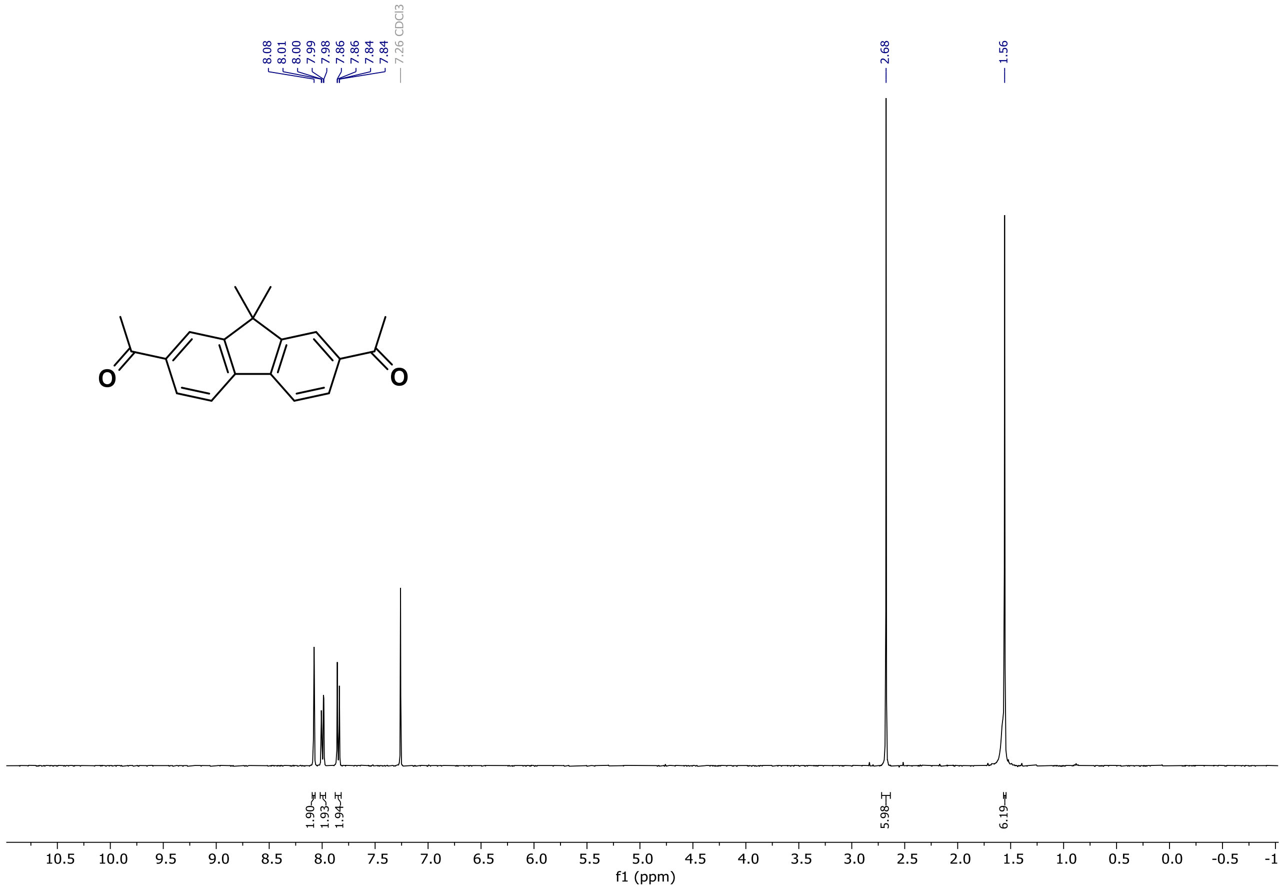

^{1}H-NMR (400 MHz, DMSO-d_6) of GH10

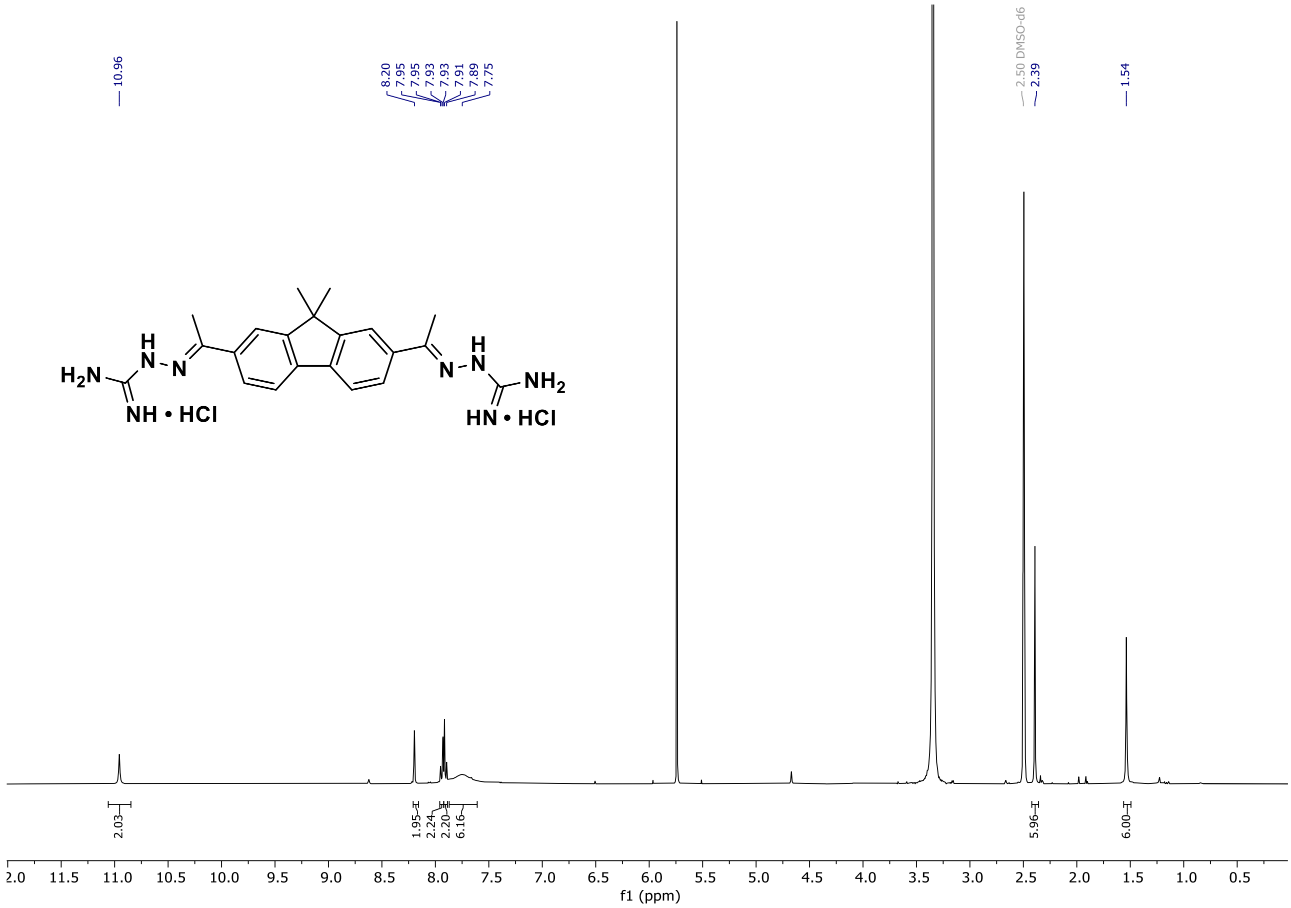

^{13}C-NMR (101 MHz, DMSO-d_6) of GH10

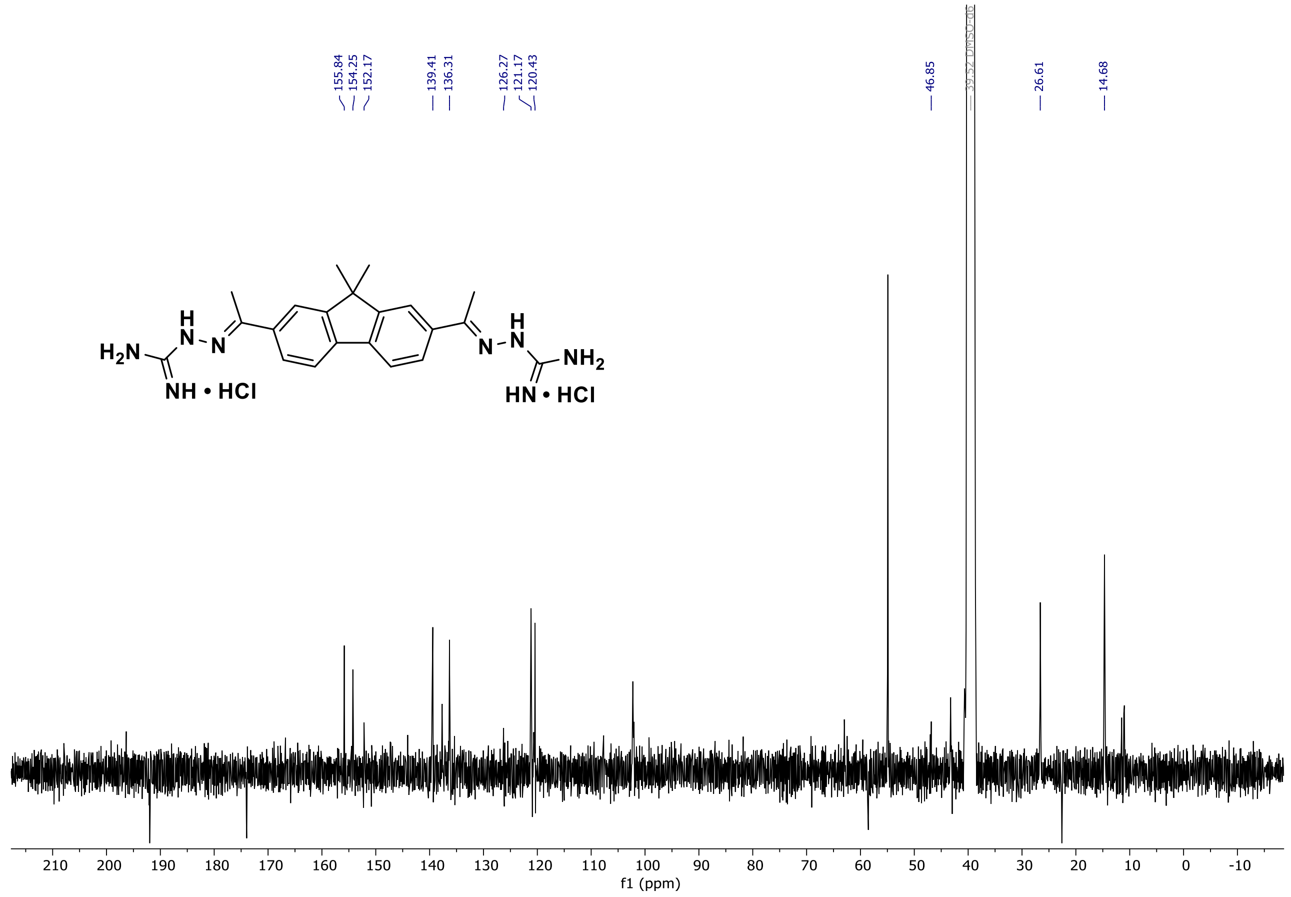

^{1}H-NMR (400 MHz, DMSO-d_6) of 12

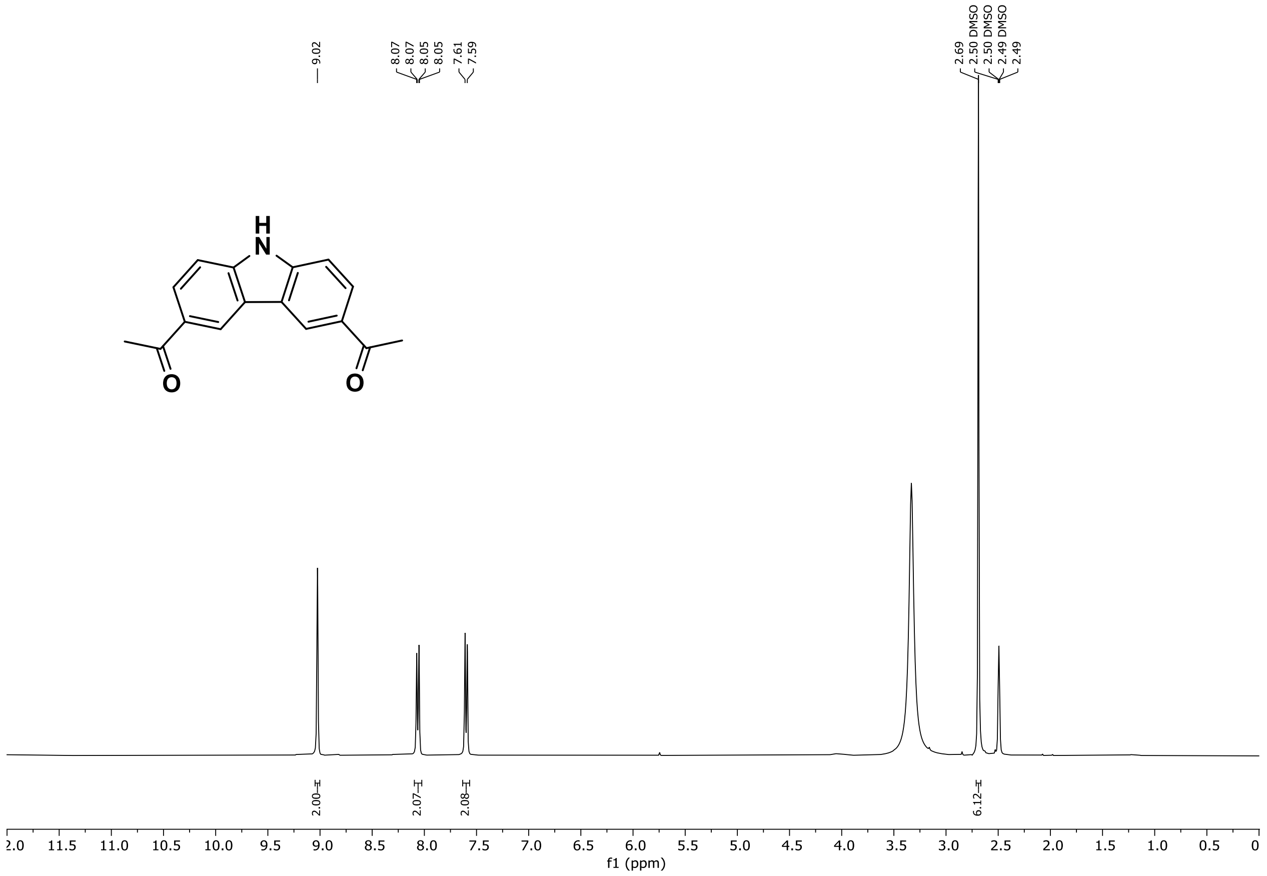

^{1}H-NMR (400 MHz, DMSO-d_6) of GH11

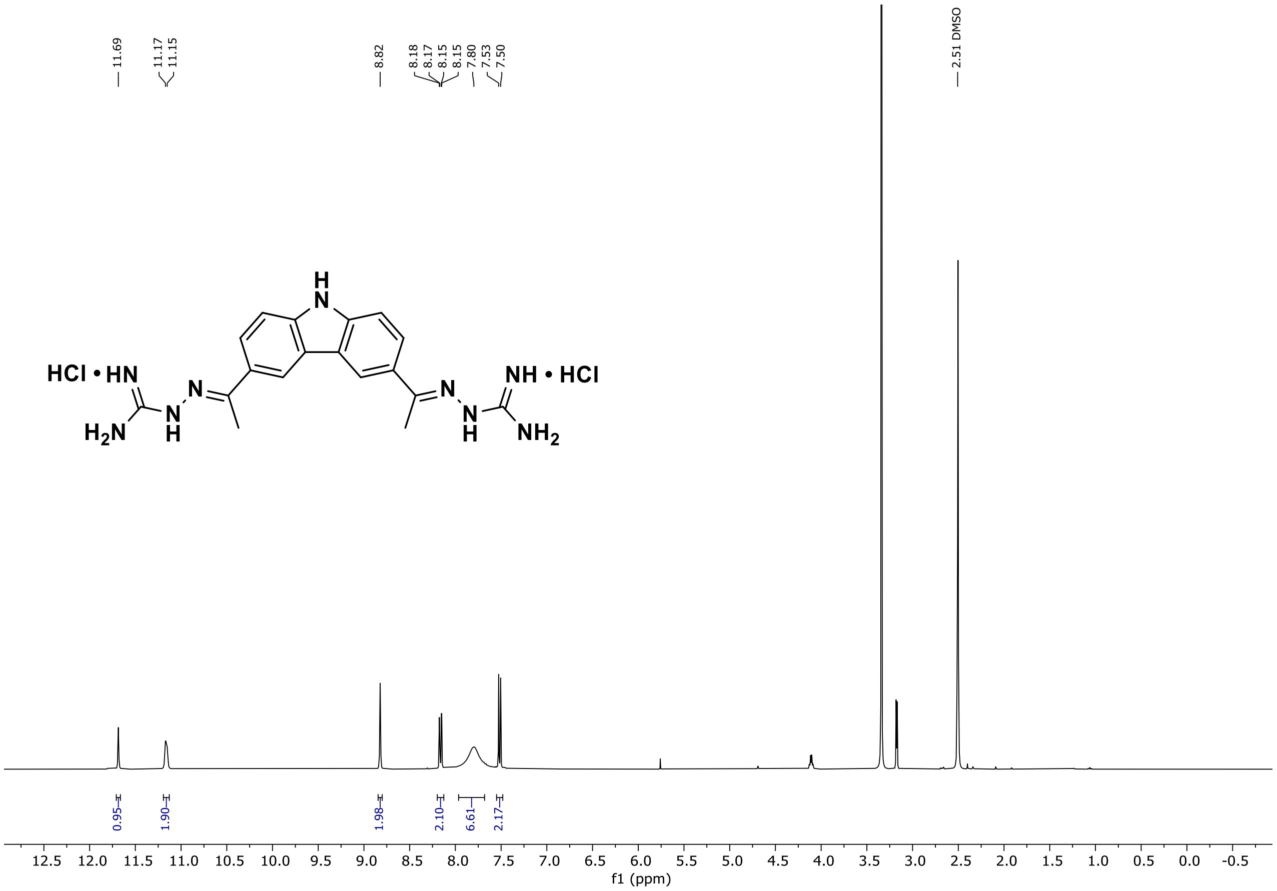

^{13}C-NMR (101 MHz, DMSO-d_6) of GH11

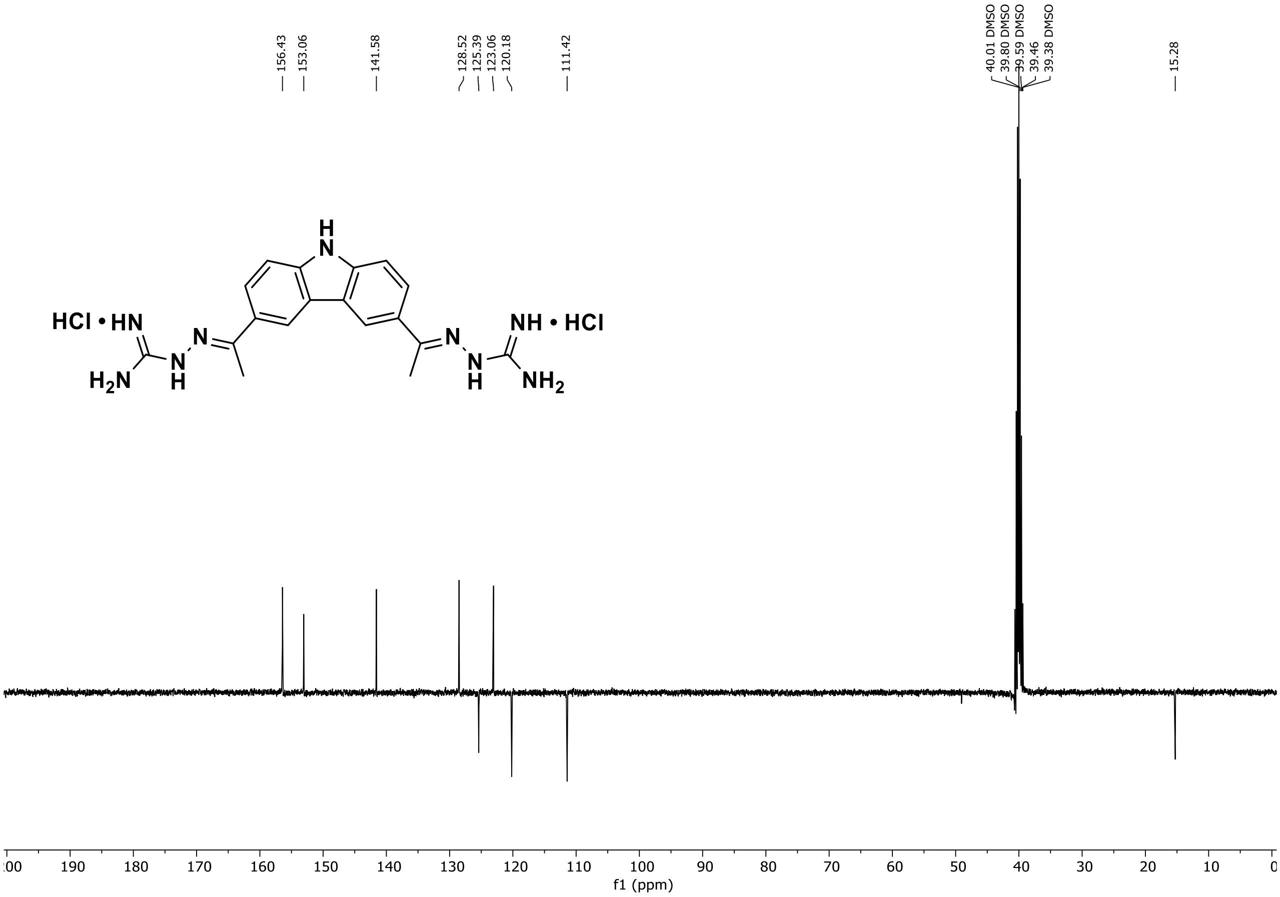

^{1}H-NMR (400 MHz, CDCl₃) of 13

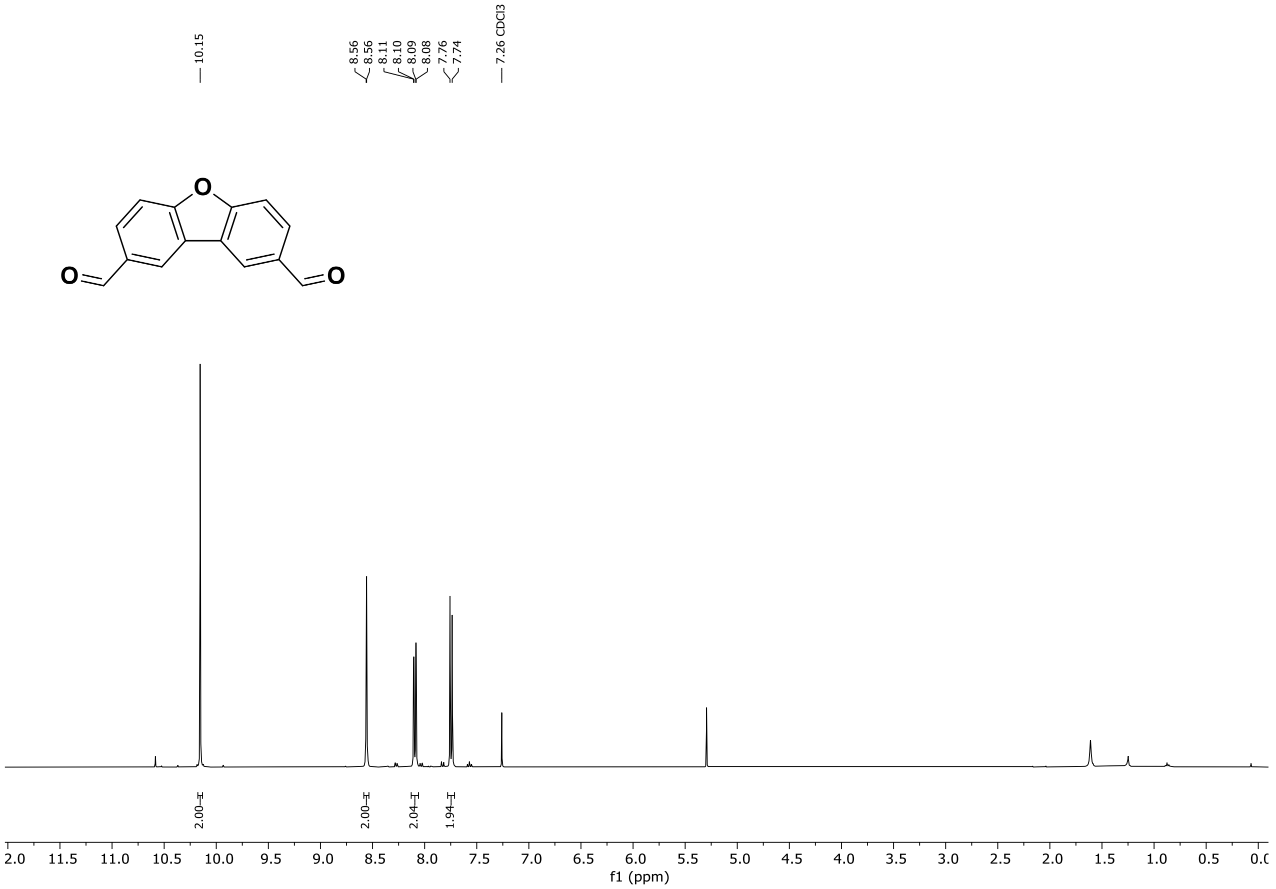

^{13}C-NMR (101 MHz, CDCl₃) of 13

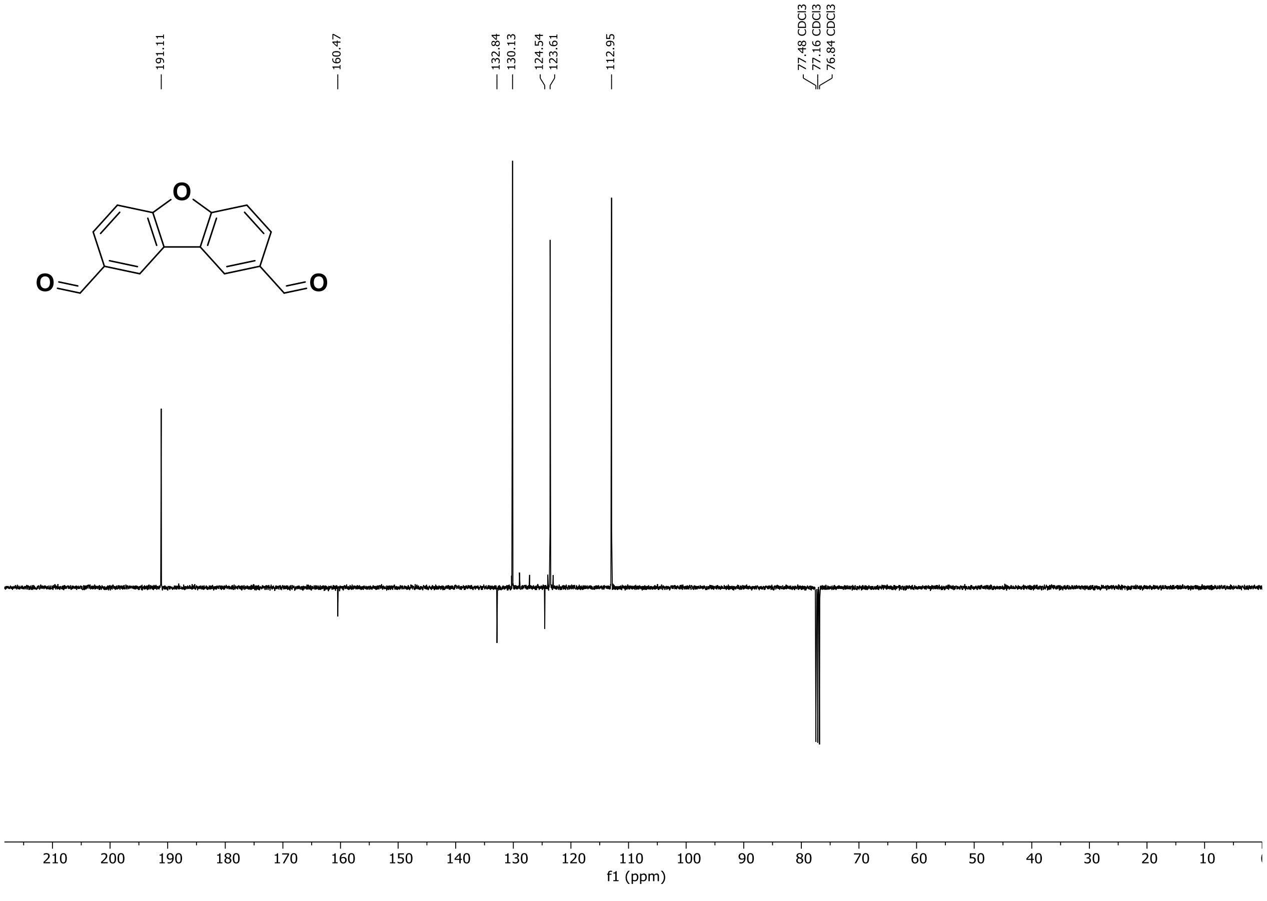

^{1}H-NMR (400 MHz, DMSO-d_6) of GH12

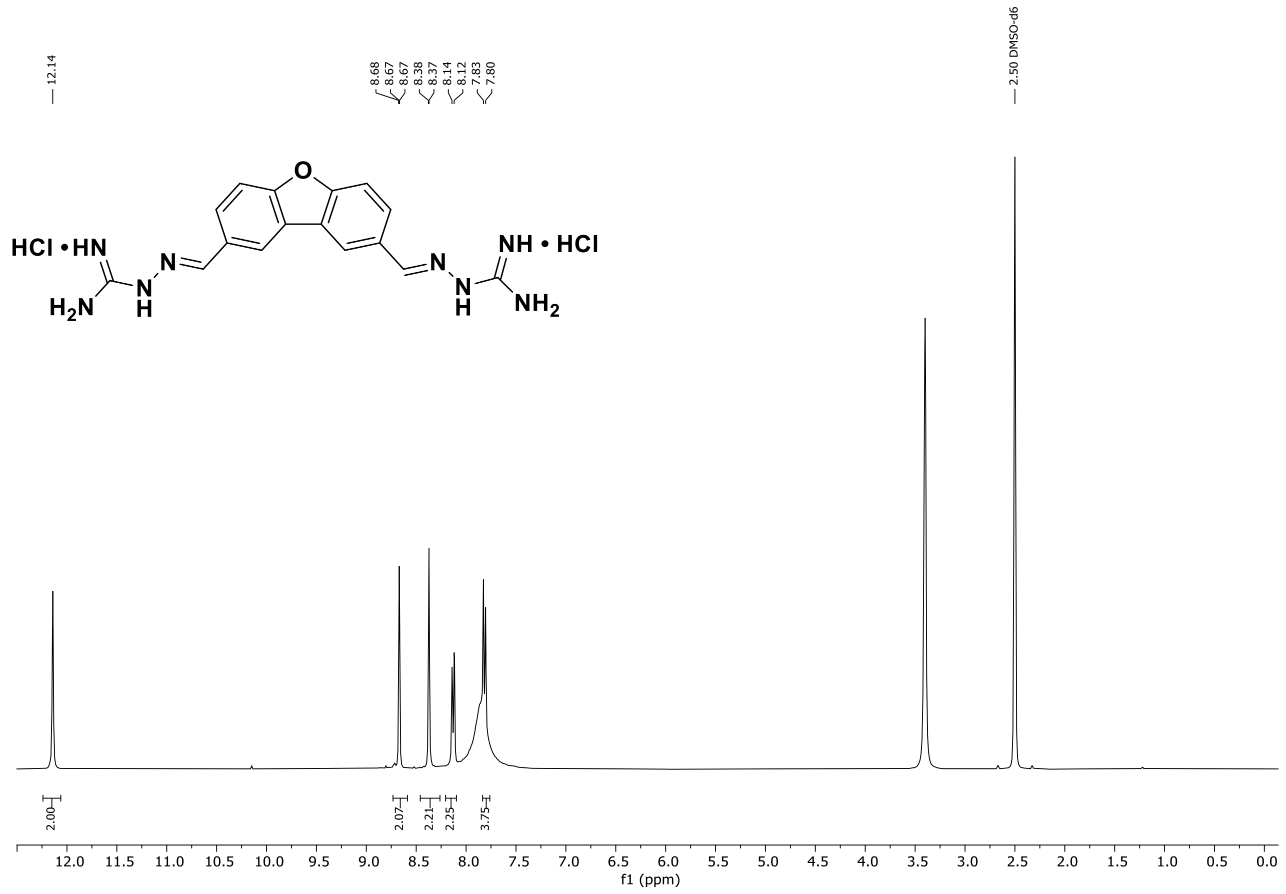

^{13}C-NMR (101 MHz, DMSO-d_6) of GH12

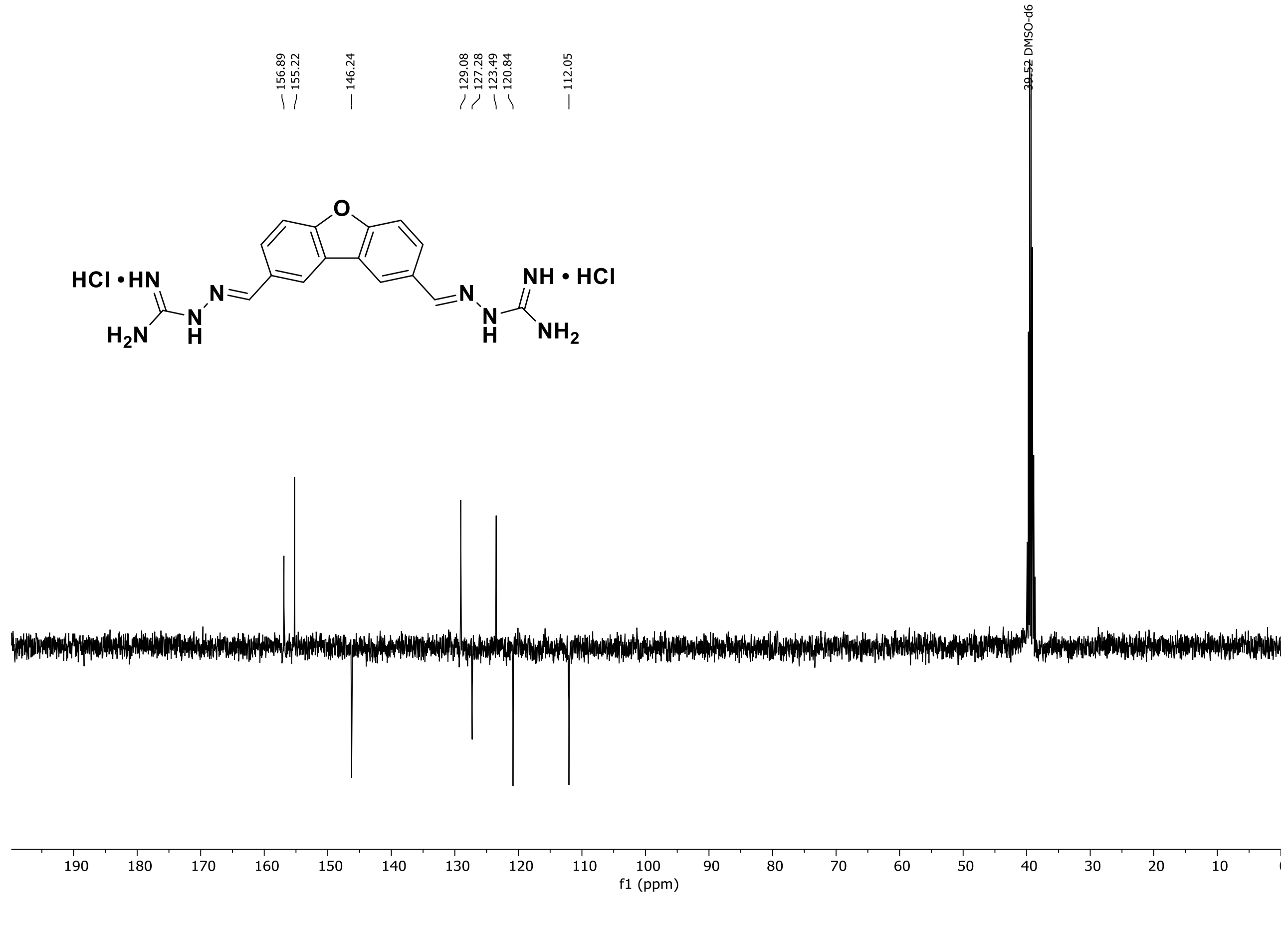

^{1}H-NMR (400 MHz, CDCl3) of 14

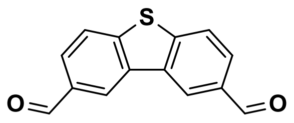

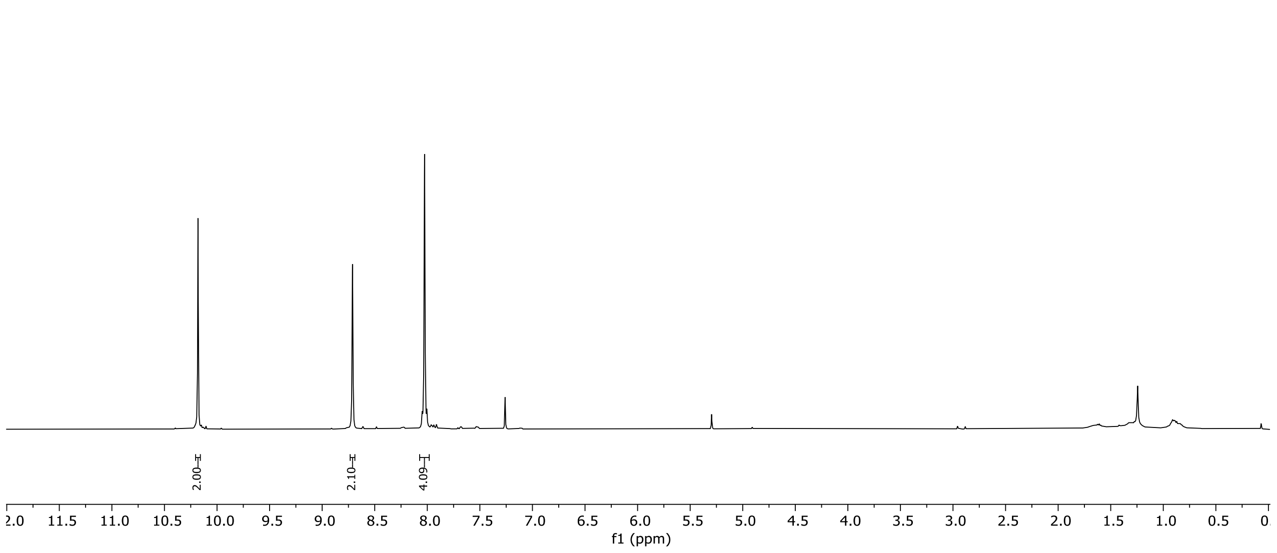

^{1}H-NMR (400 MHz, DMSO-d_6) of GH13

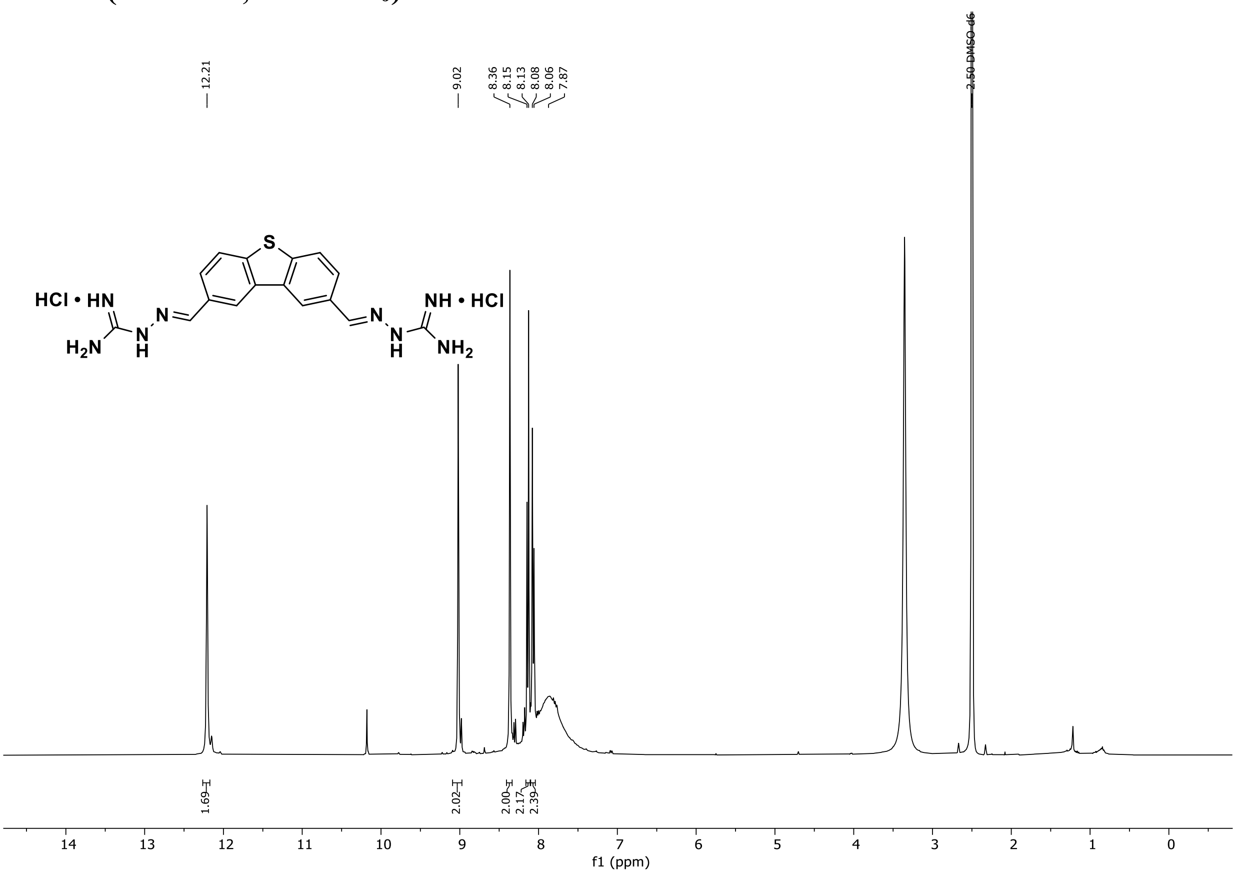

^{13}C-NMR (101 MHz, DMSO-d_6) of GH13

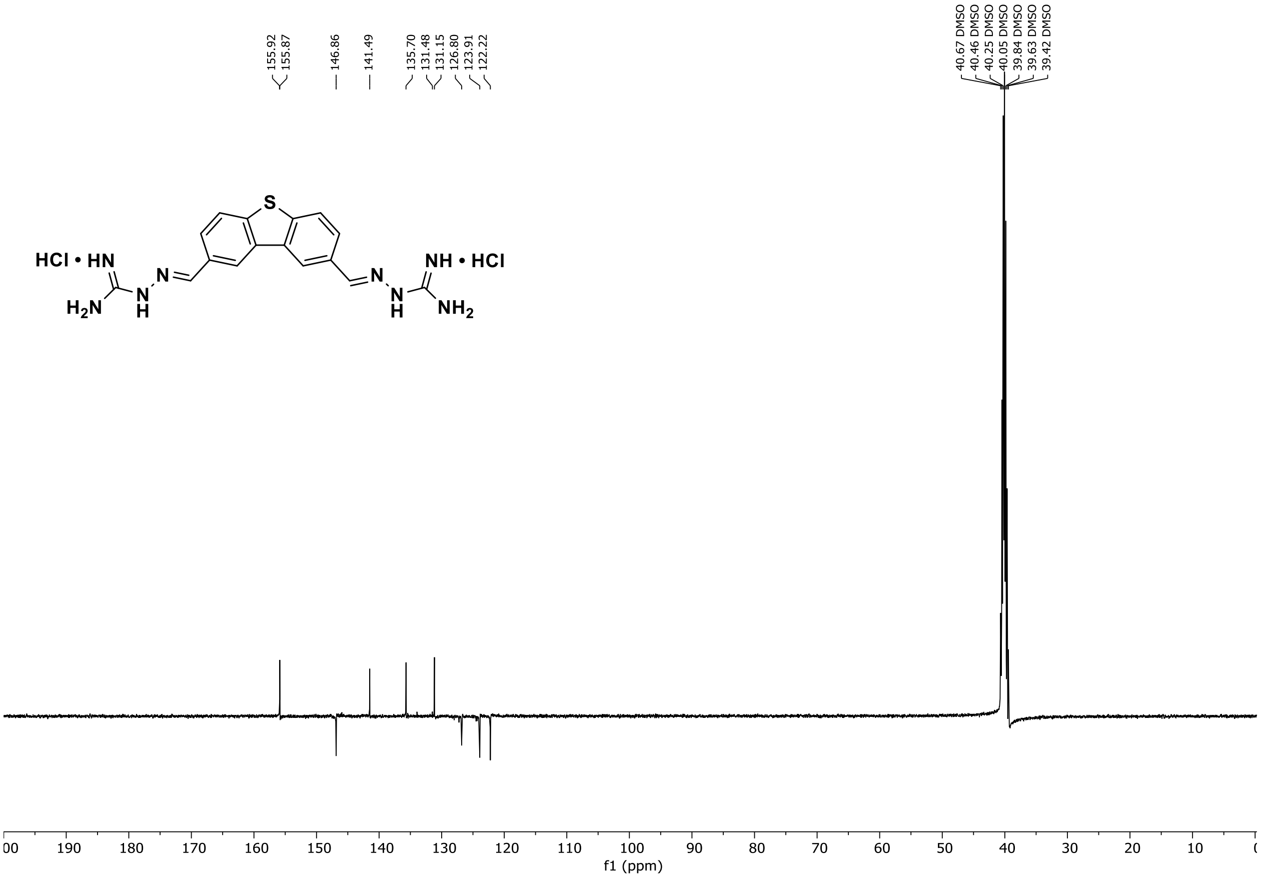

^{1}H-NMR (400 MHz, $CDCl_3$) of 15

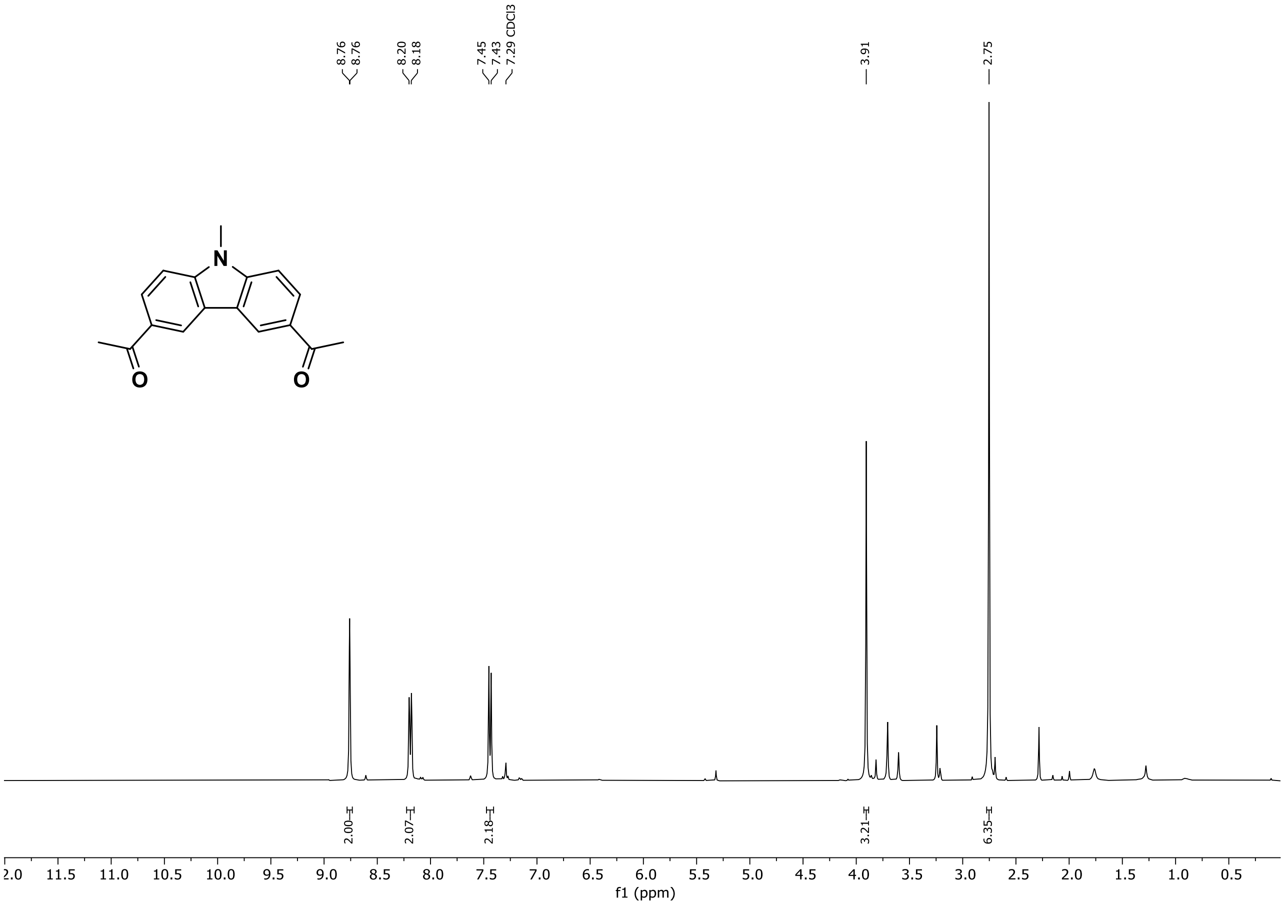

^{13}C-NMR (101 MHz, $CDCl_3$) of 15

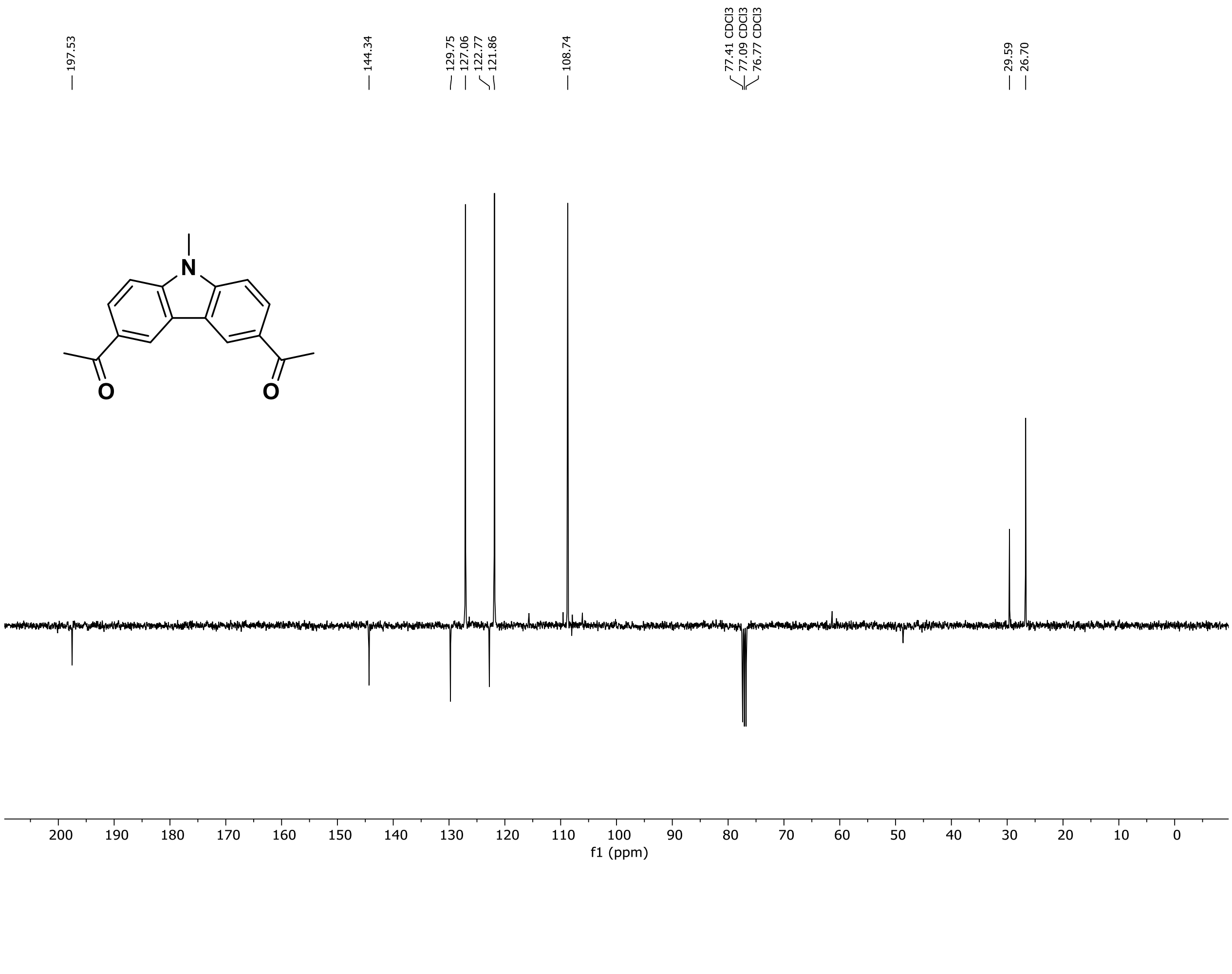

^{1}H-NMR (400 MHz, DMSO-d_6) of GH14

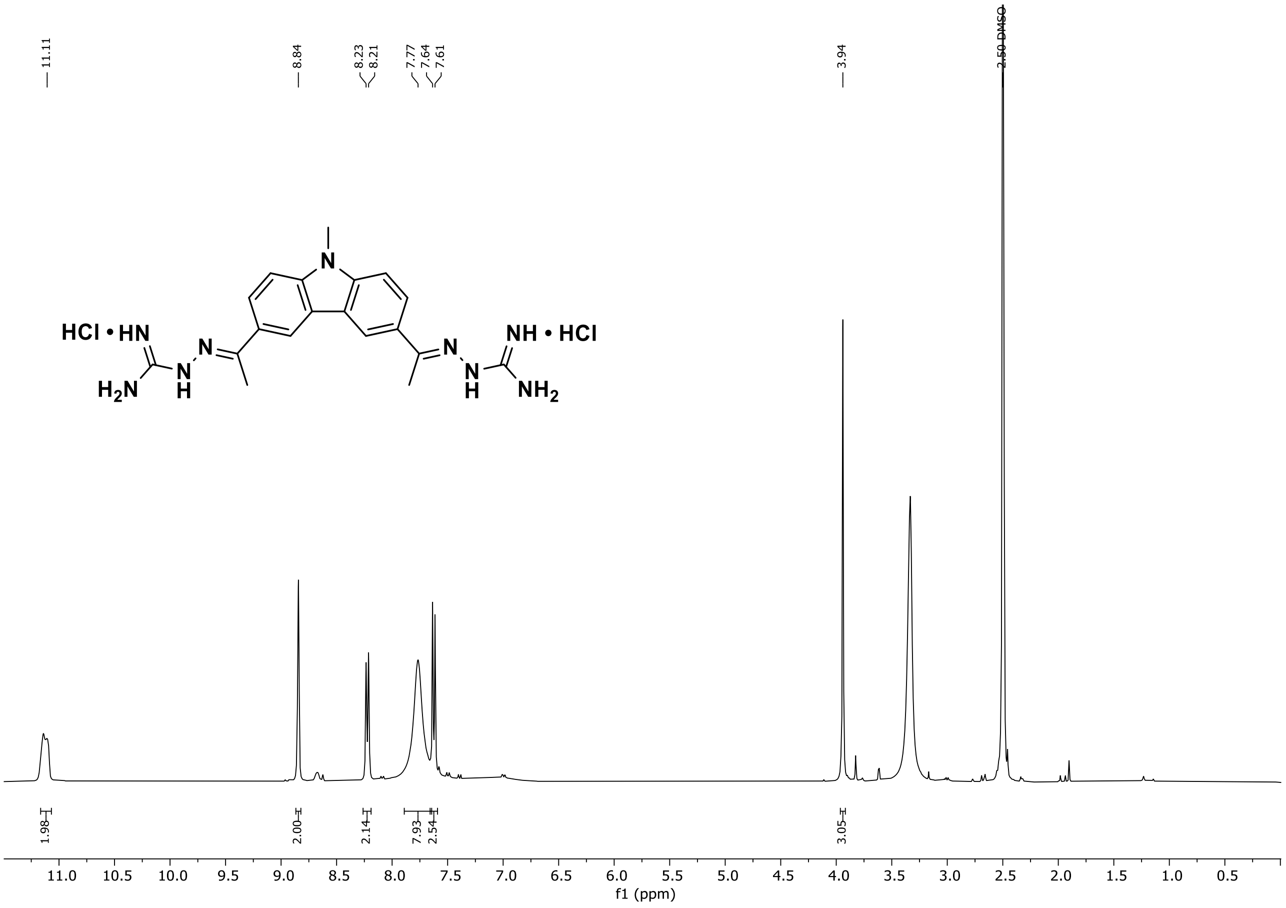

^{13}C-NMR (101 MHz, DMSO-d_6) of GH14

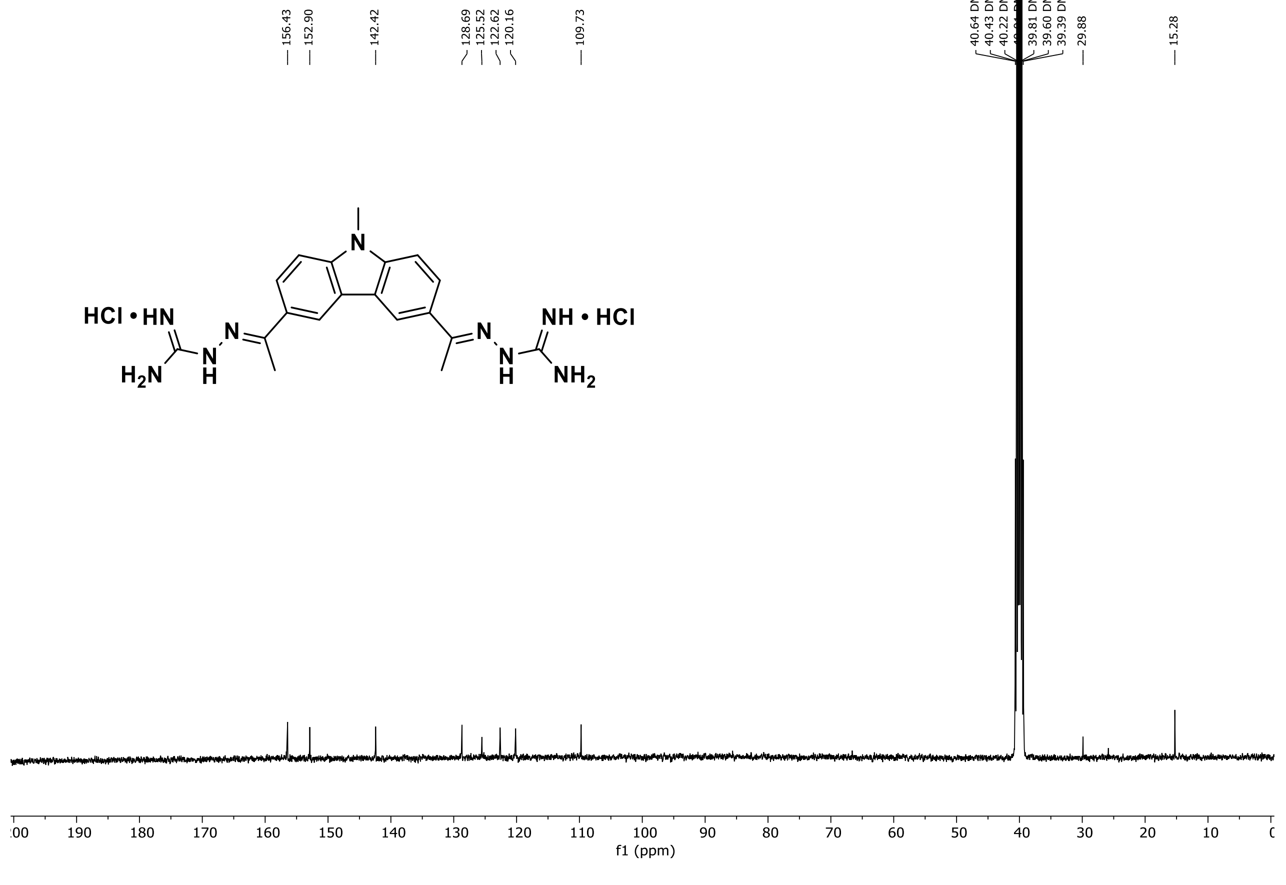

^{1}H-NMR (400 MHz, CDCl3) of 16

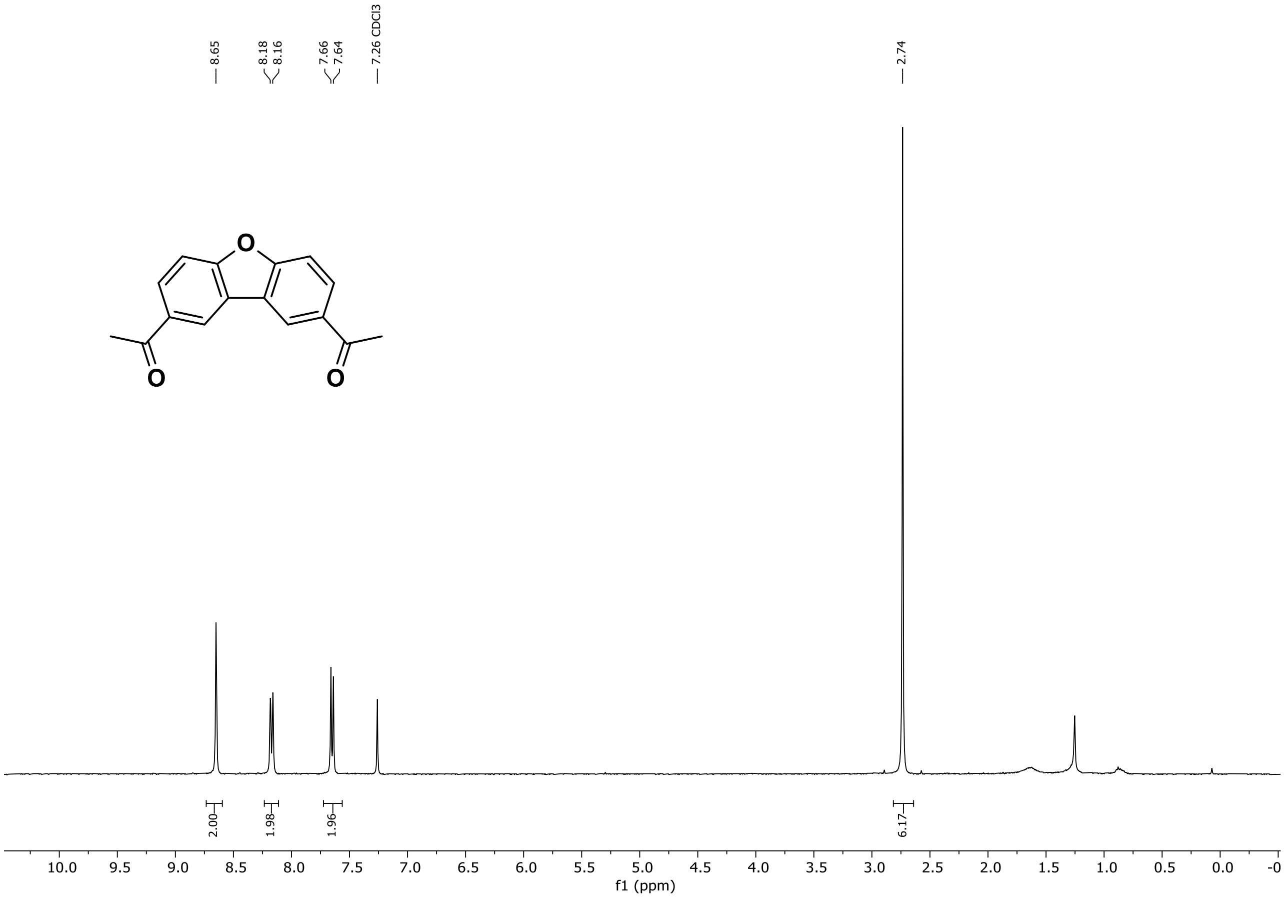

^{13}C-NMR (101 MHz, CDCl3) of 16

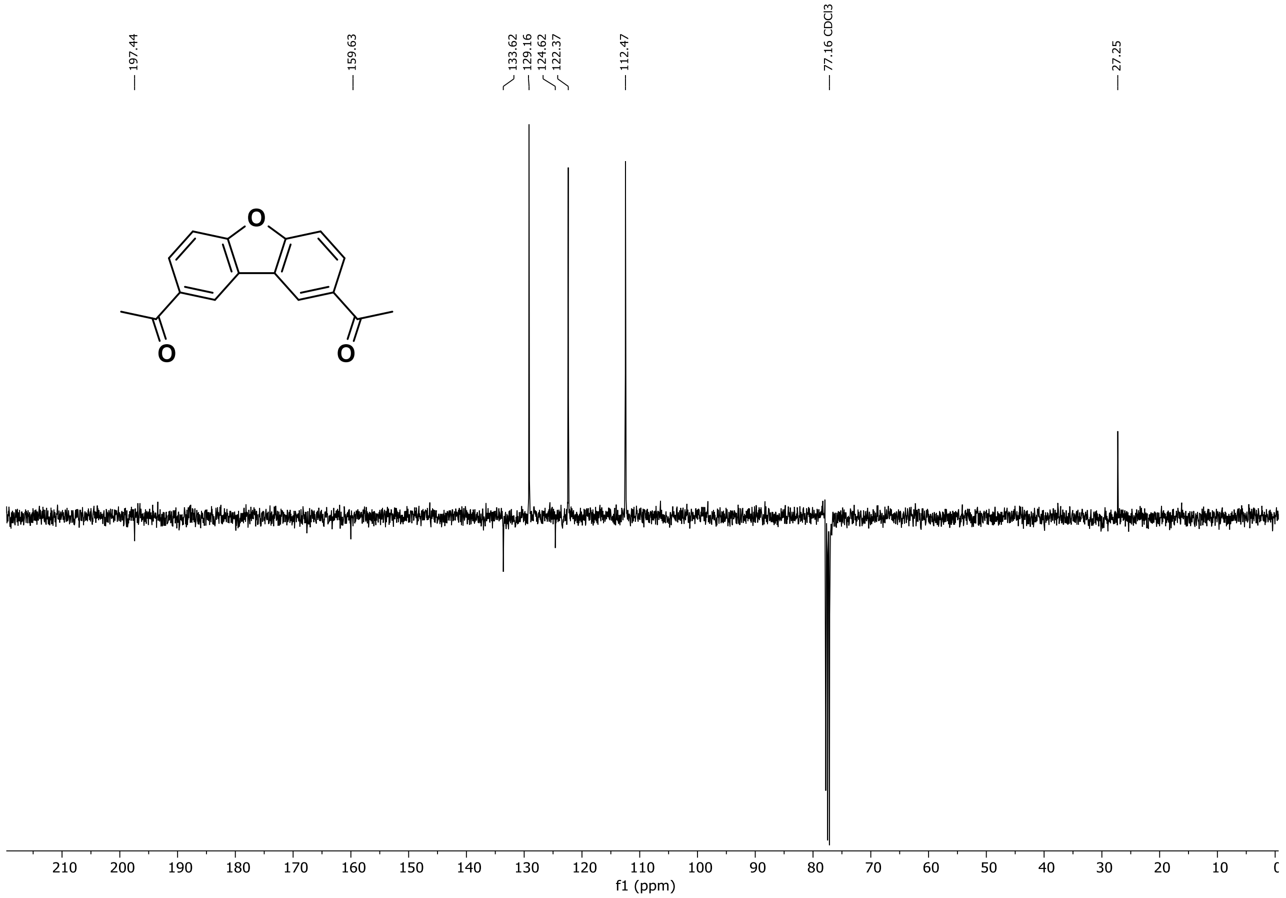

^{1}H-NMR (400 MHz, DMSO-d_6) of GH15

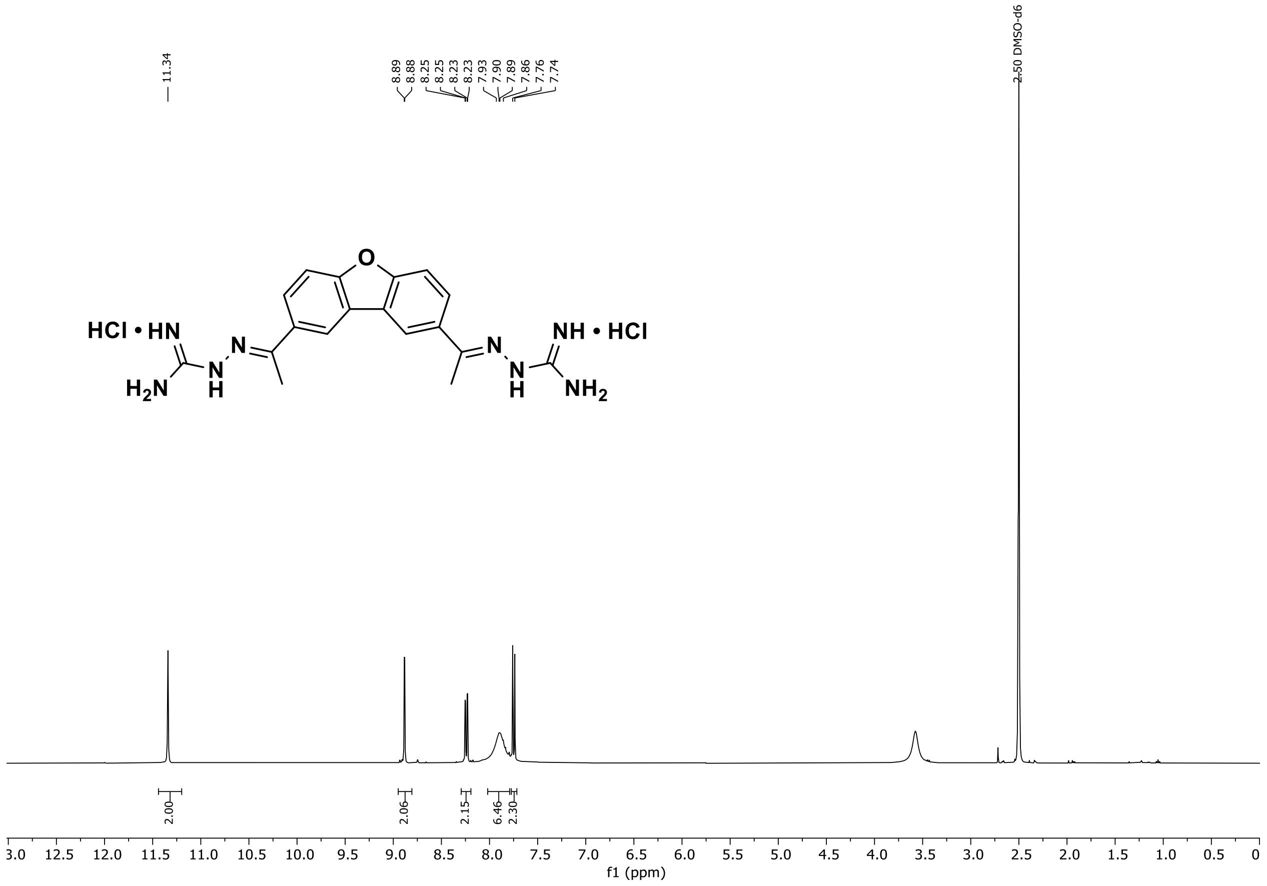

^{13}C-NMR (101 MHz, DMSO-d_6) of GH15

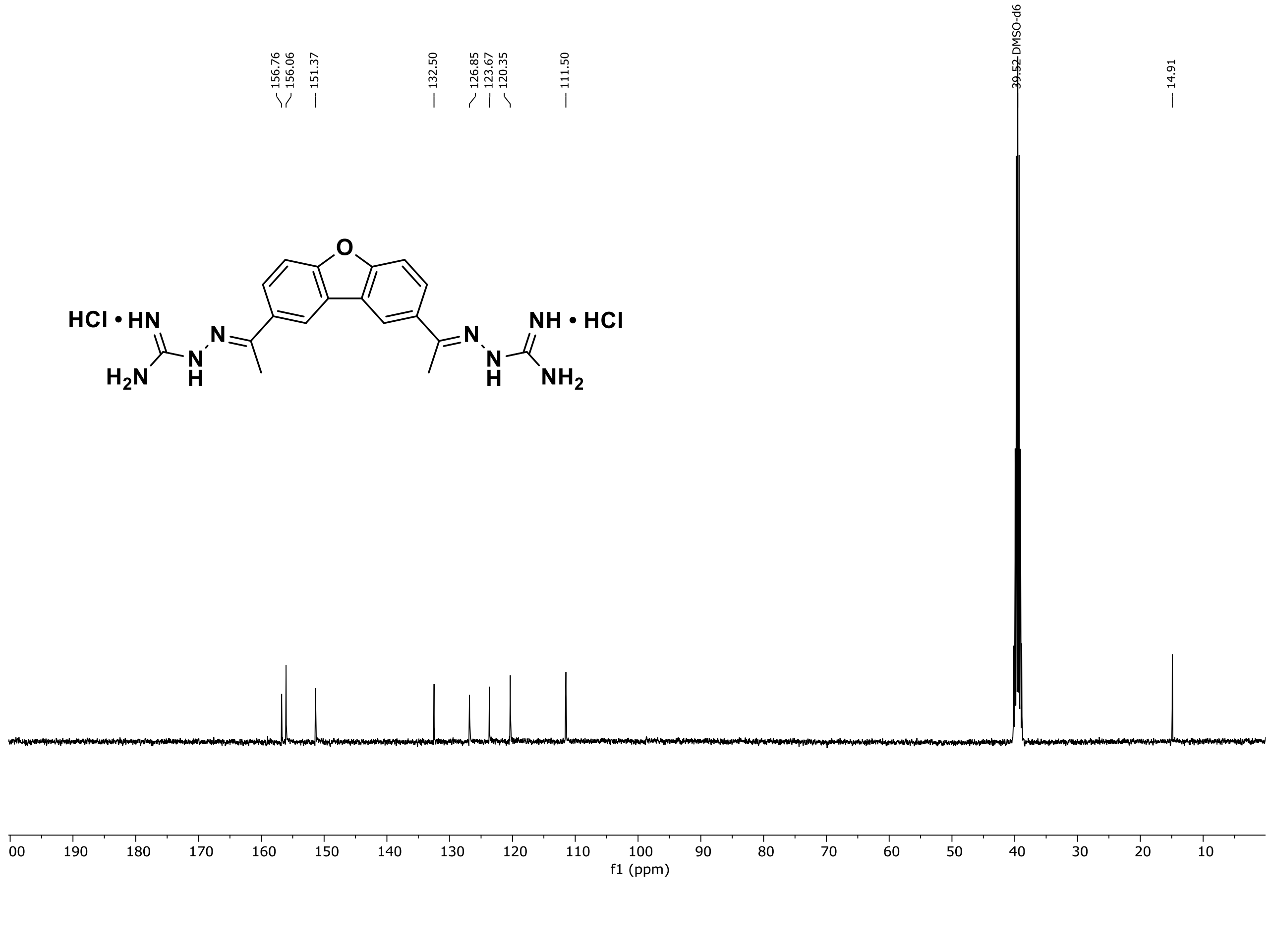

^{1}H-NMR (400 MHz, $CDCl_3$) of 17

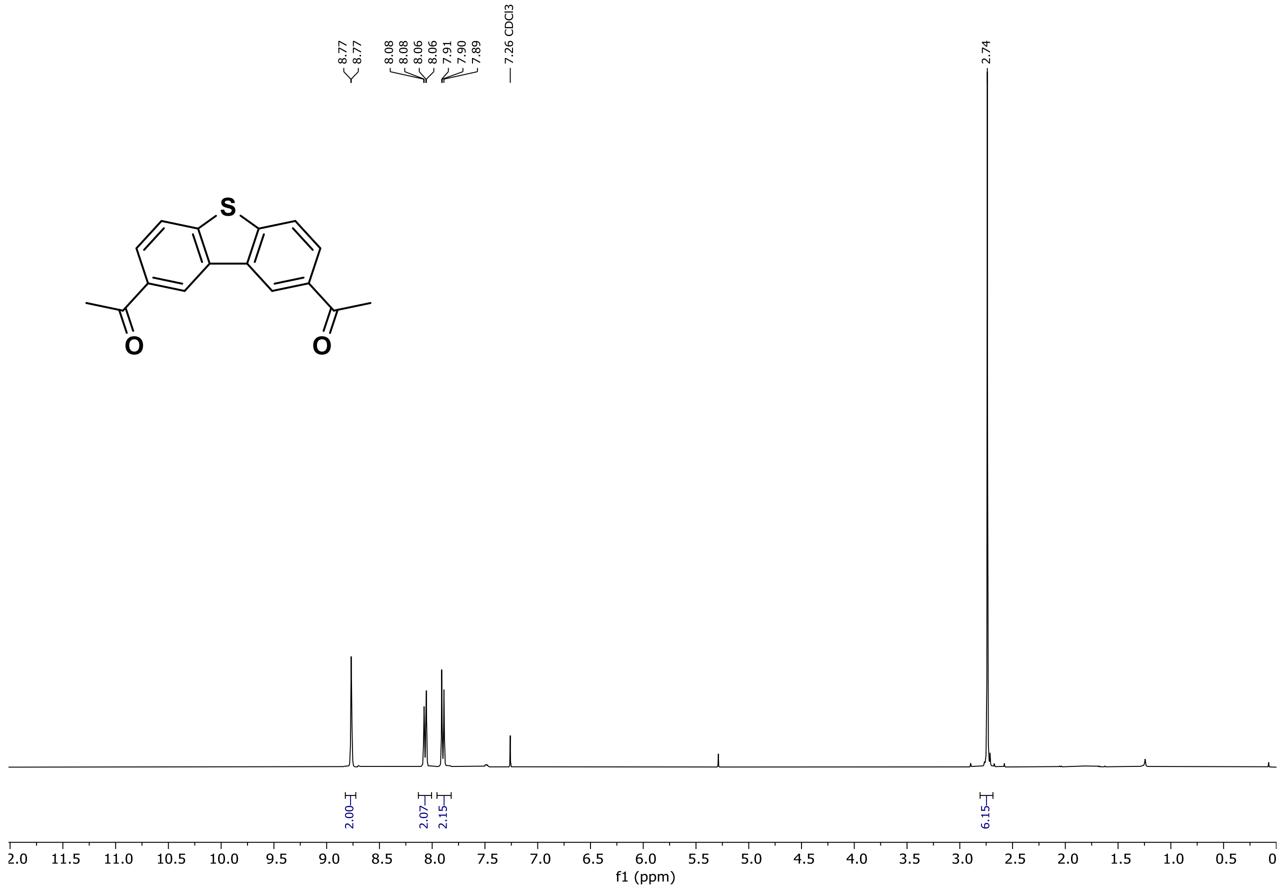

^{13}C-NMR (101 MHz, $CDCl_3$) of 17

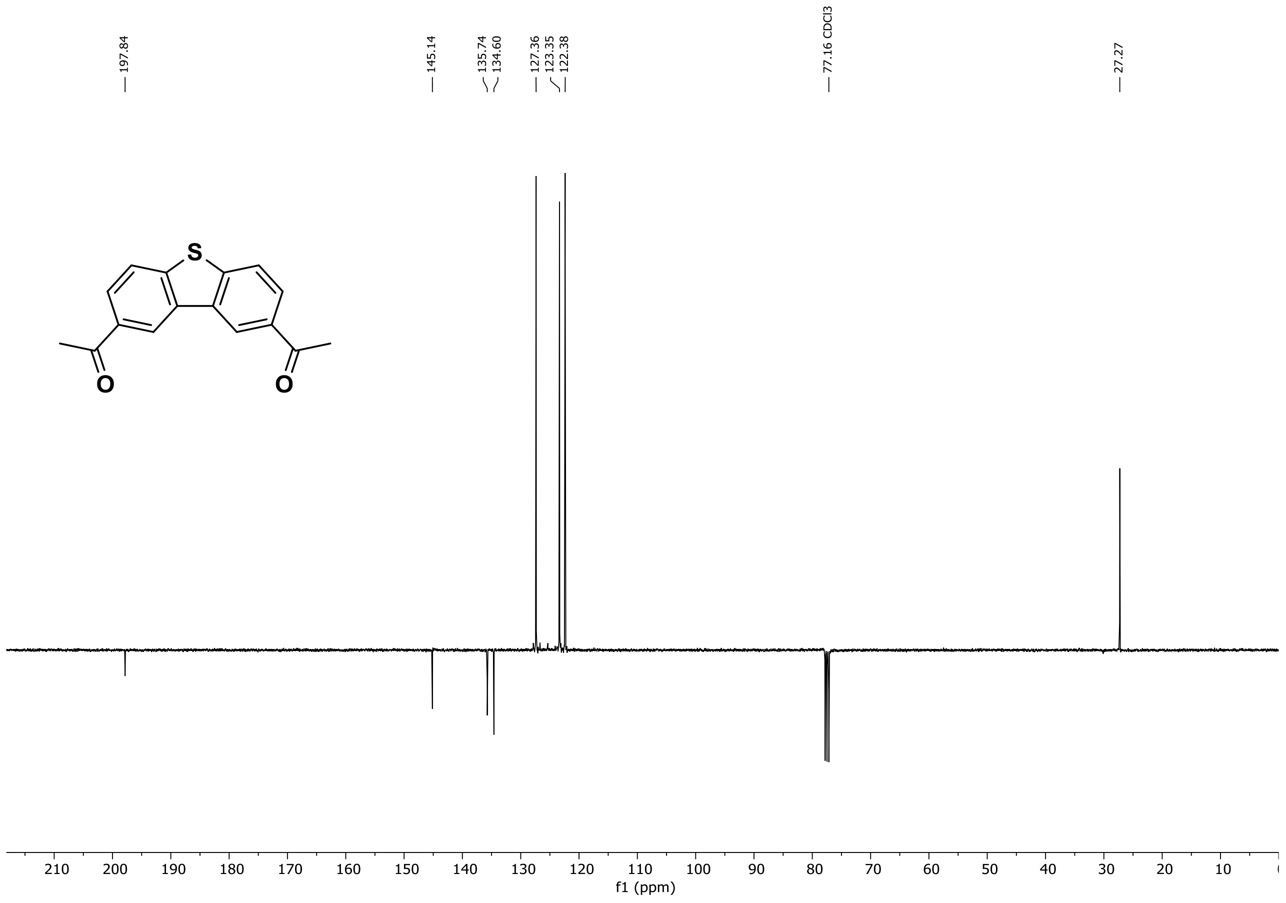

^{1}H-NMR (400 MHz, DMSO-d_6) of GH16

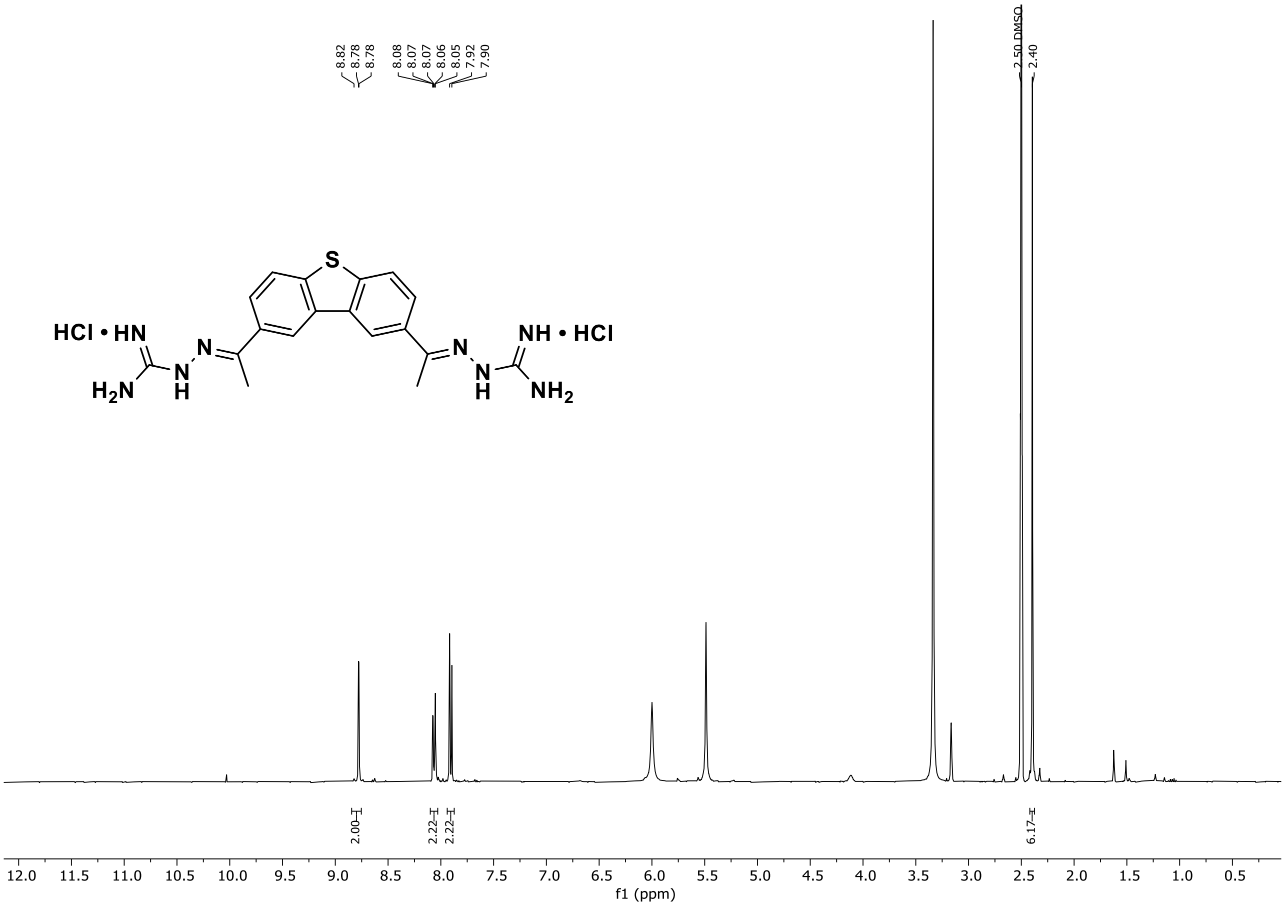

^{13}C-NMR (101 MHz, DMSO-d_6) of GH16

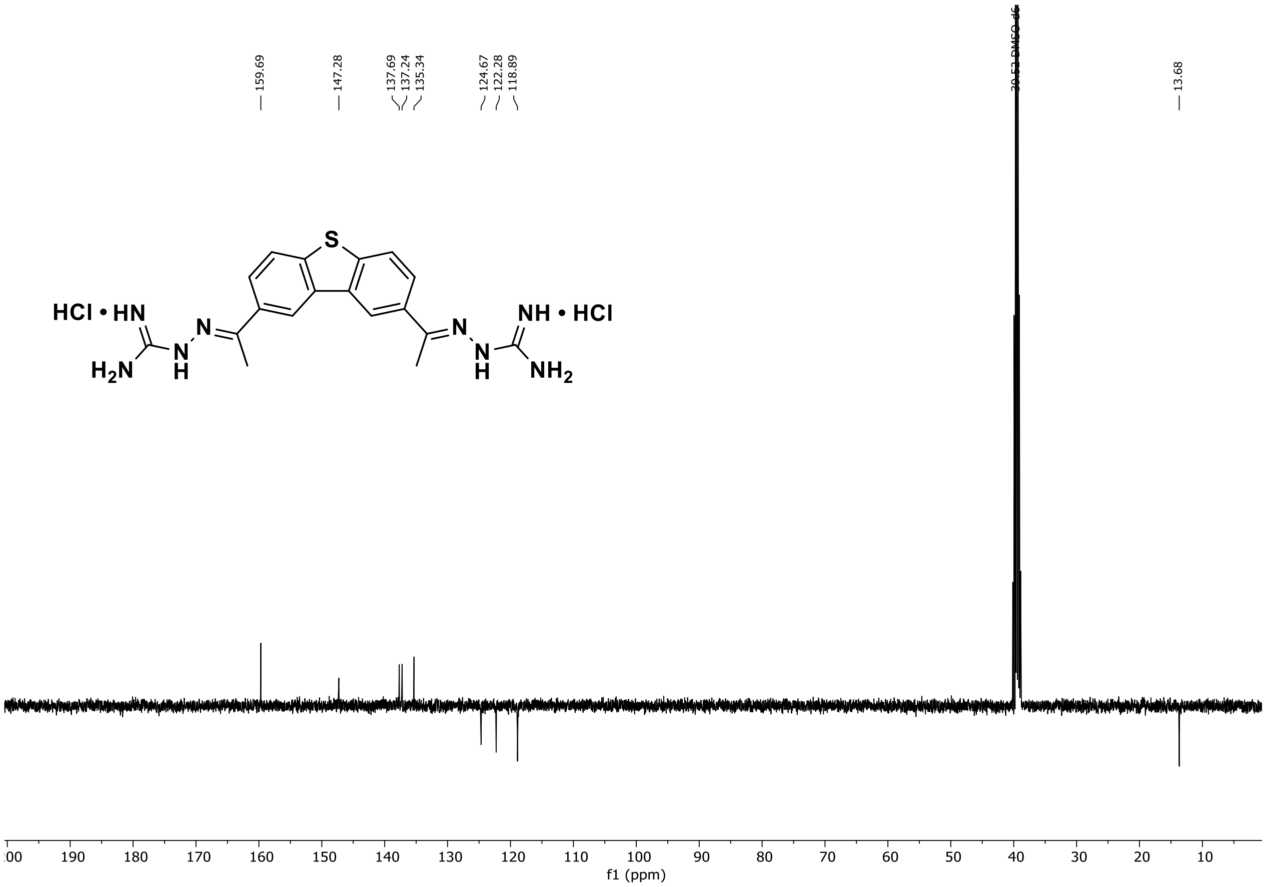

LC-MS of GH1

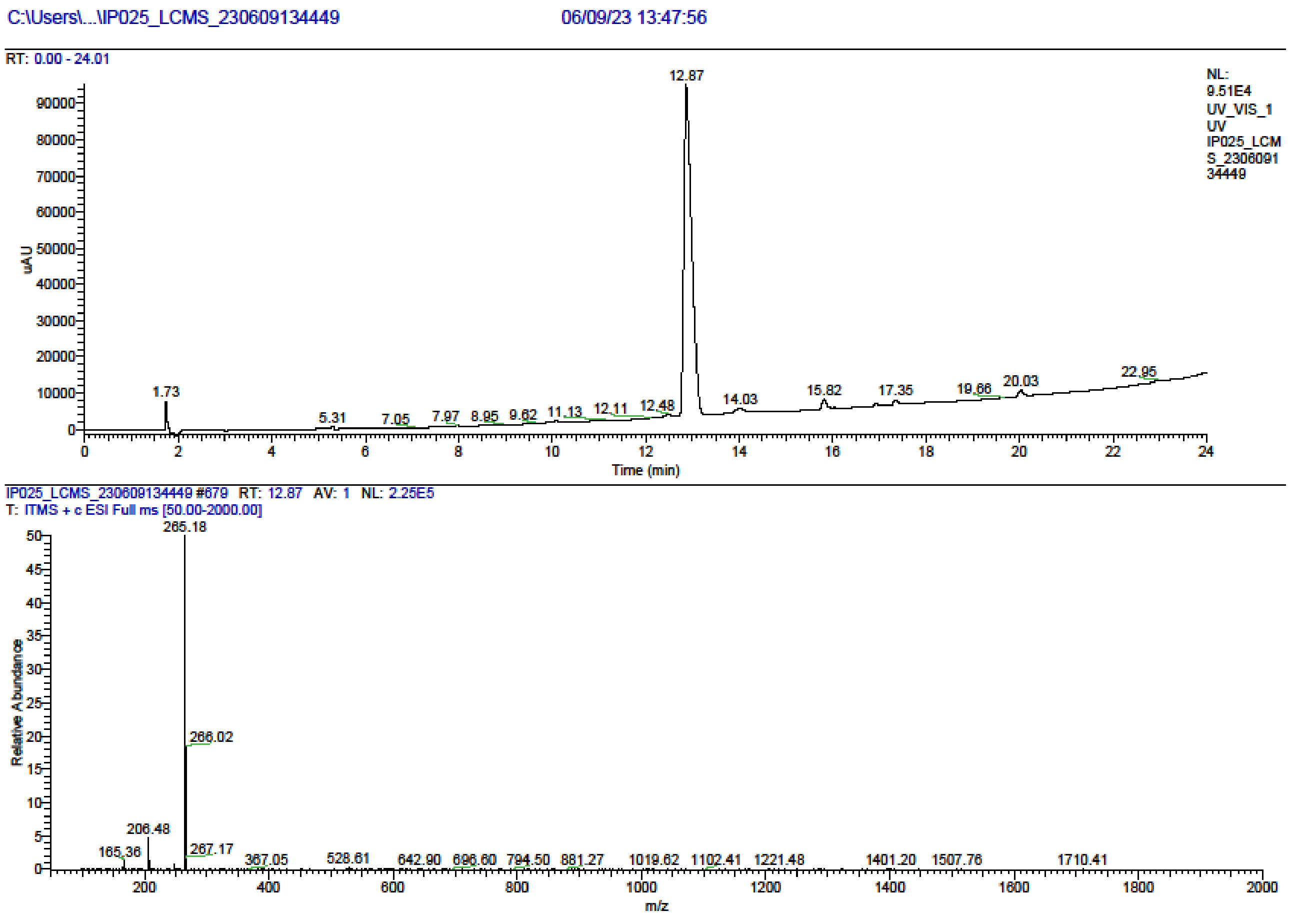

LC-MS of GH2

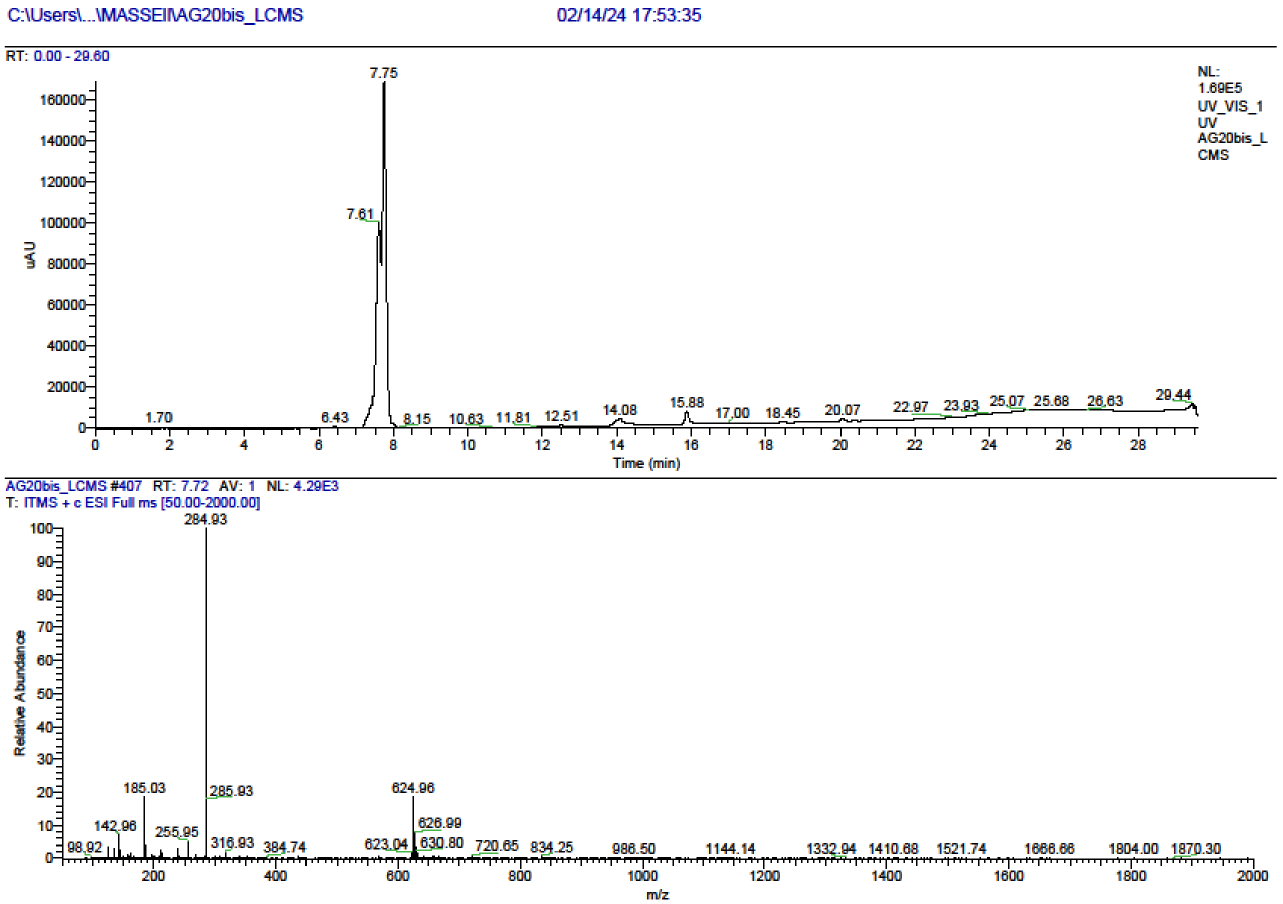

LC-MS of GH3

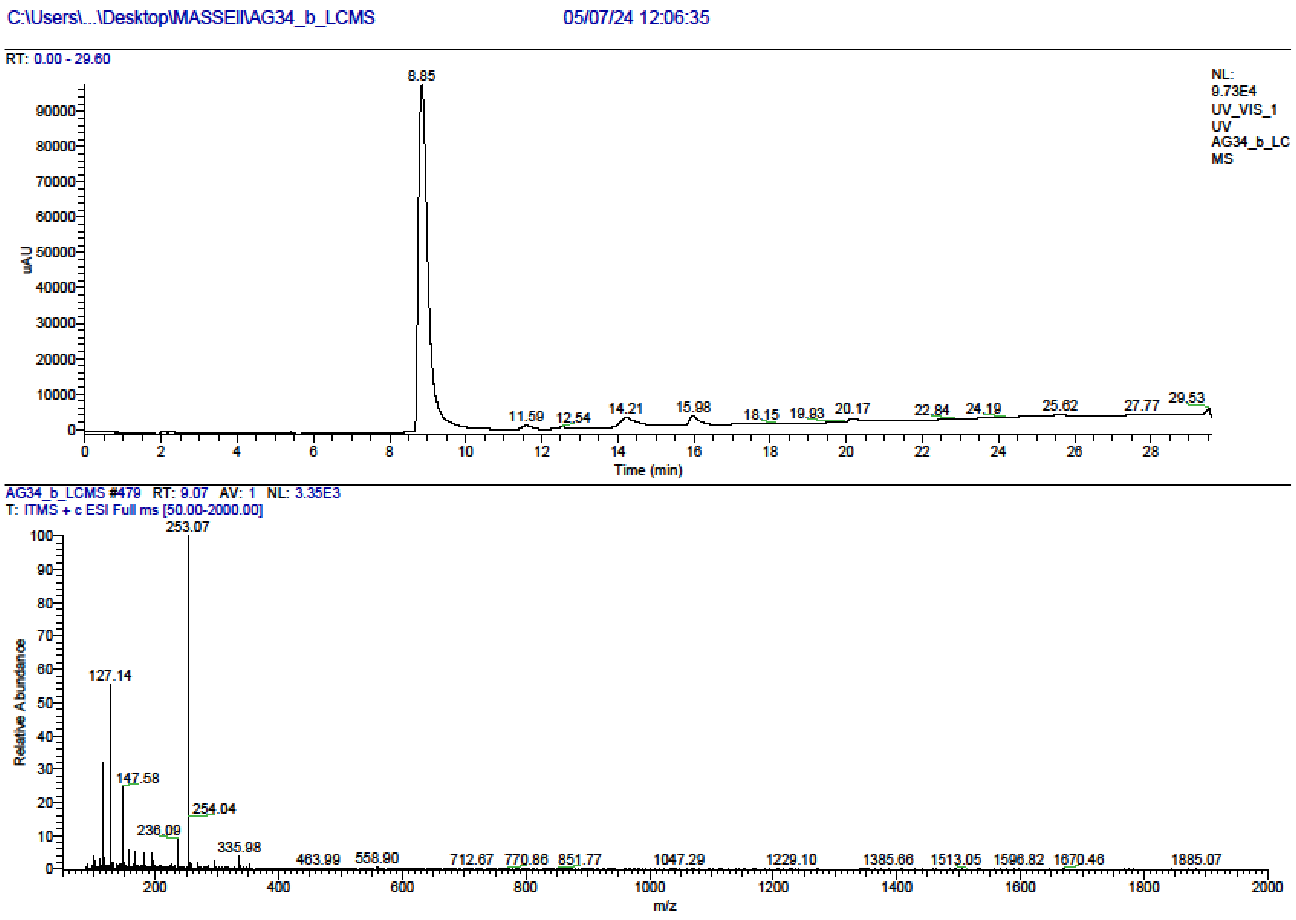

LC-MS of GH4

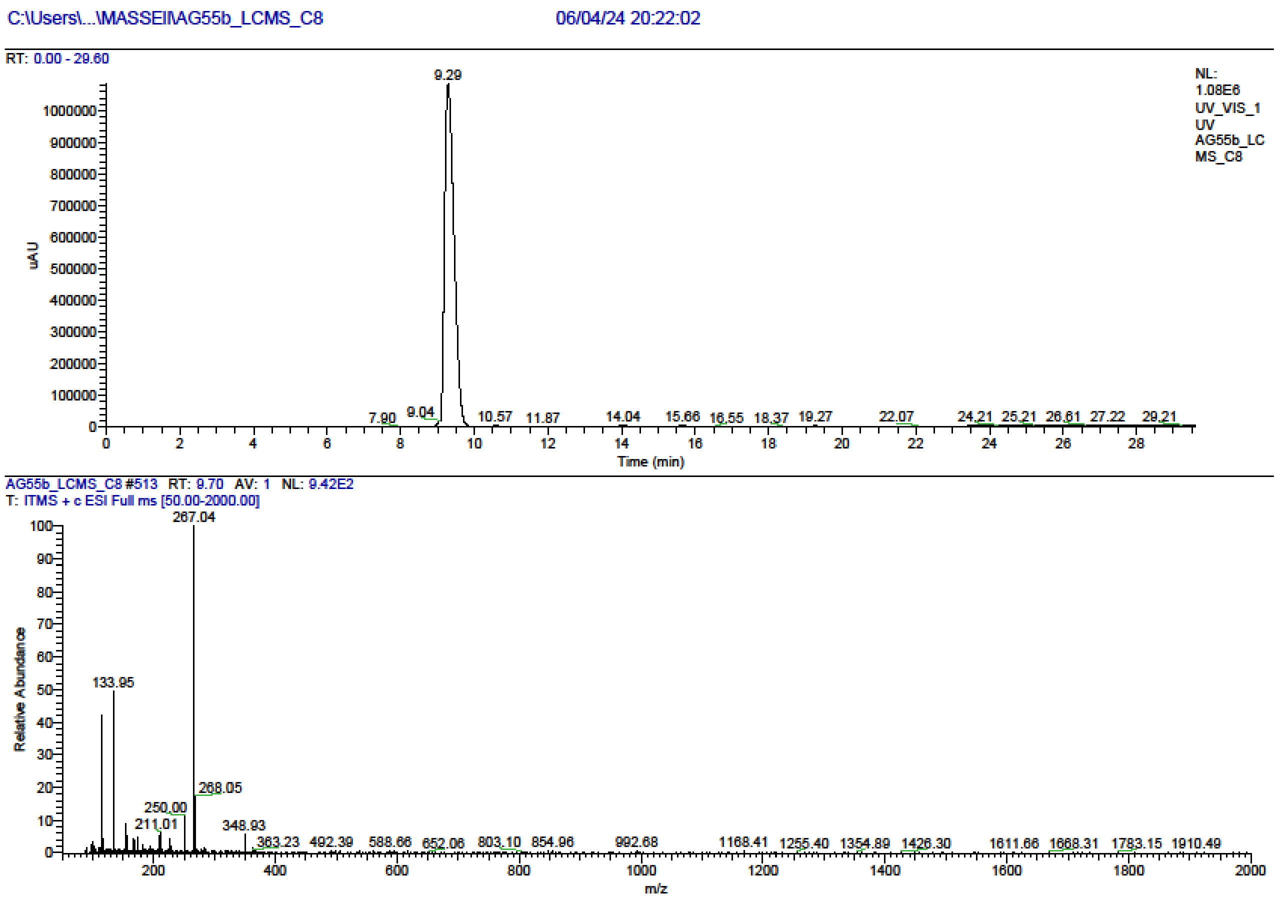

LC-MS of GH5

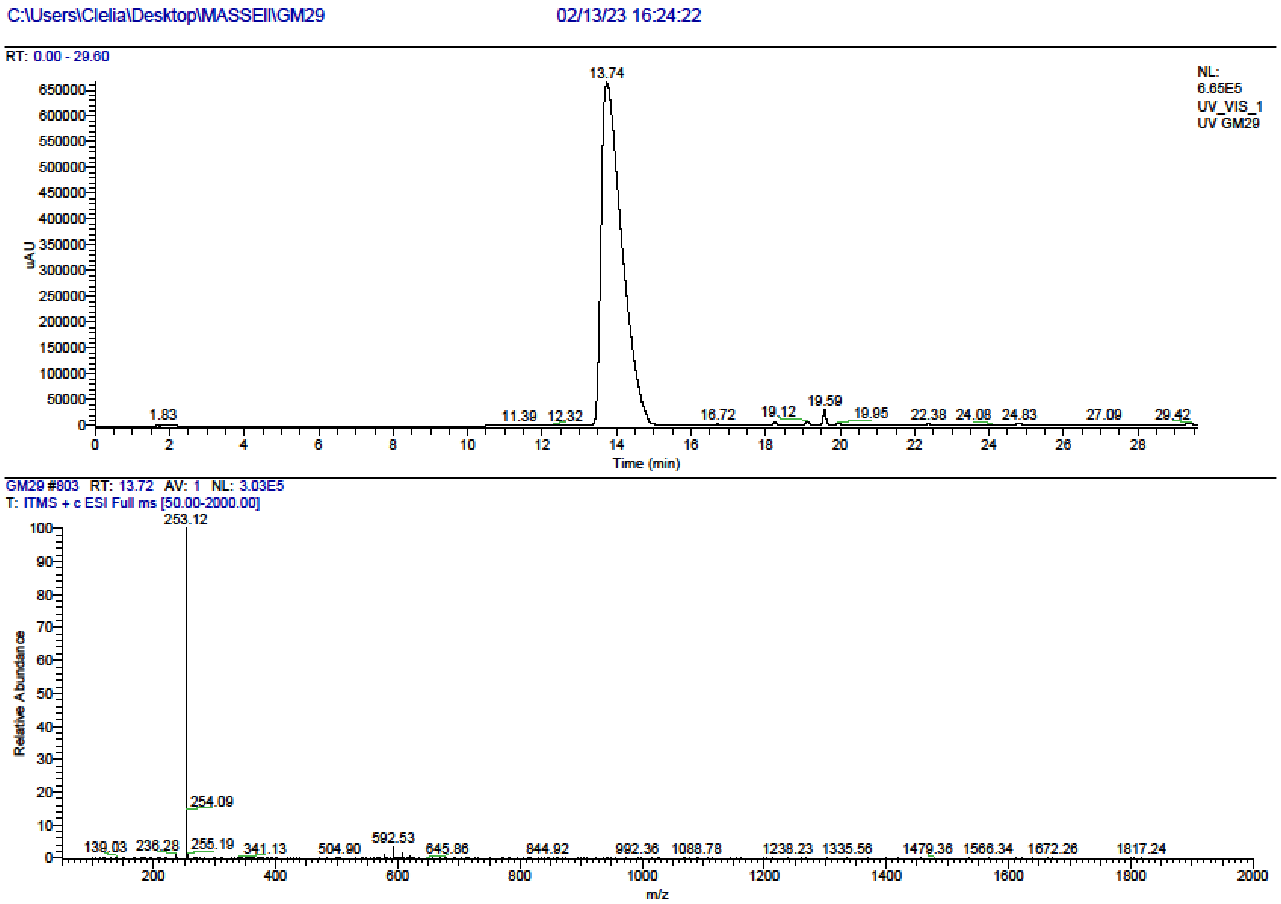

LC-MS of GH6

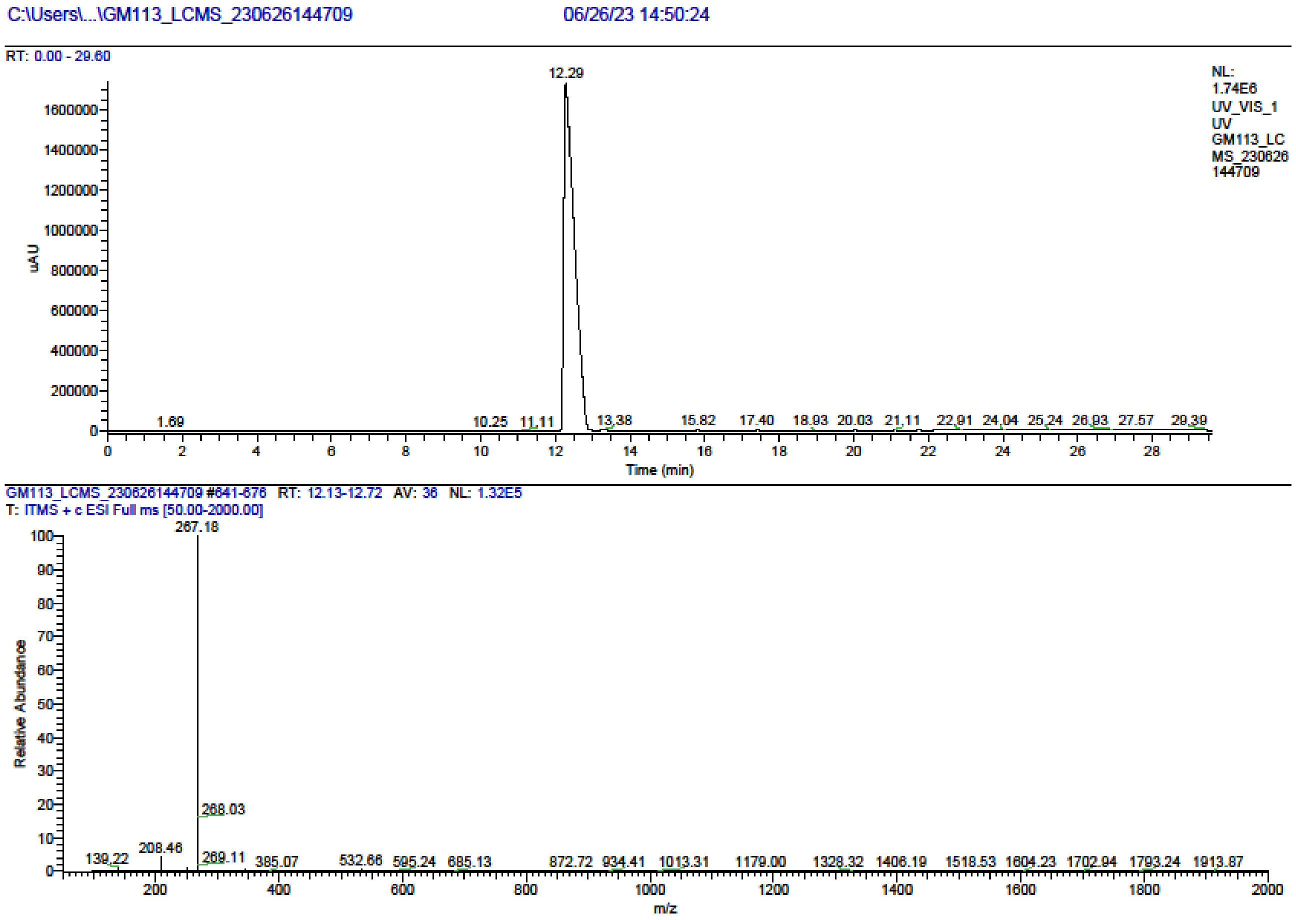

LC-MS of GH7

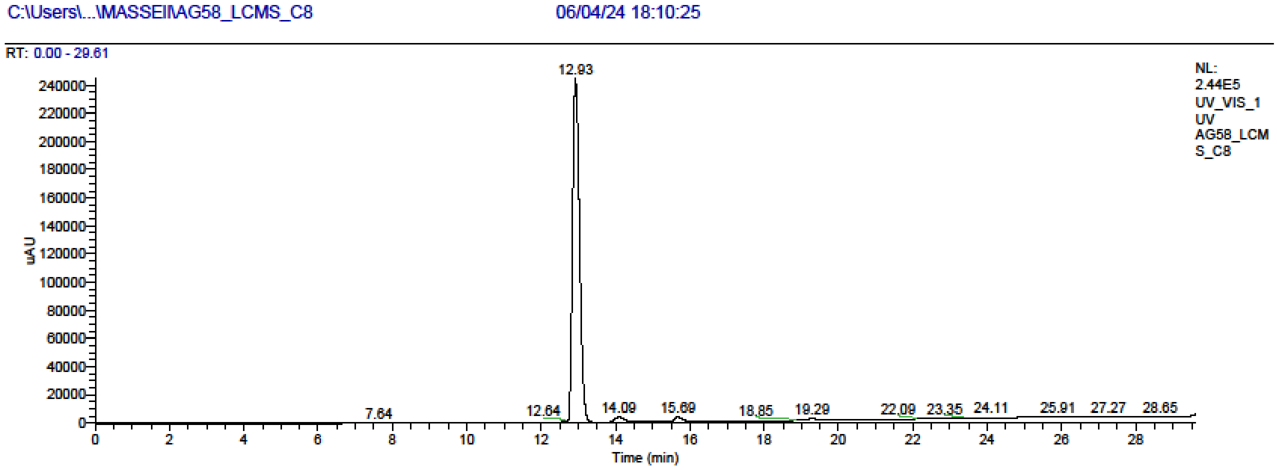

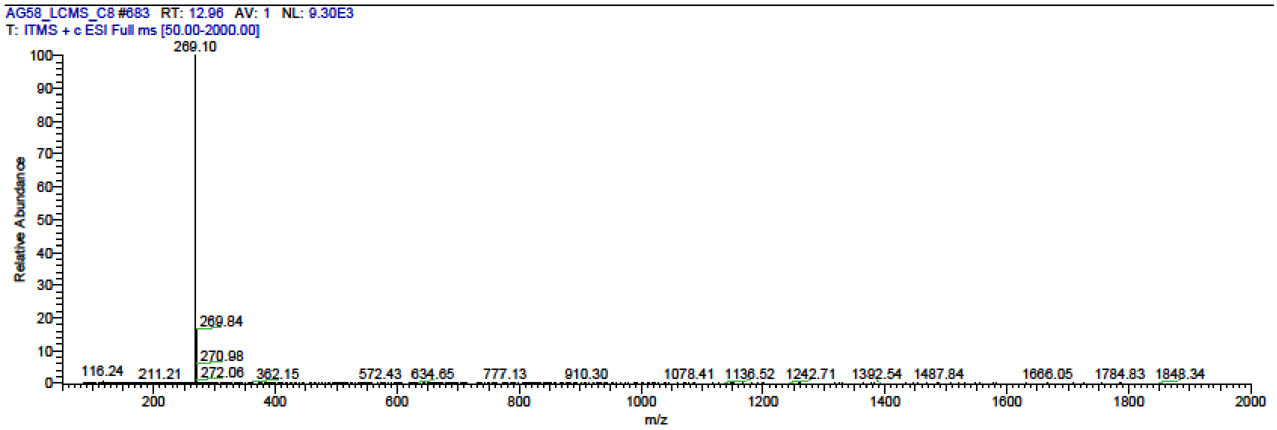

LC-MS of GH8

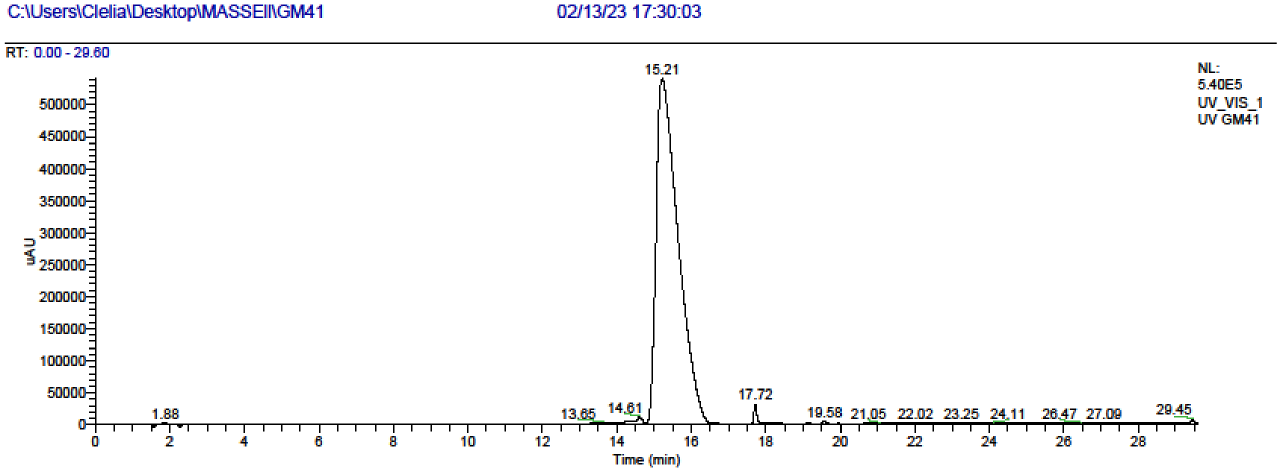

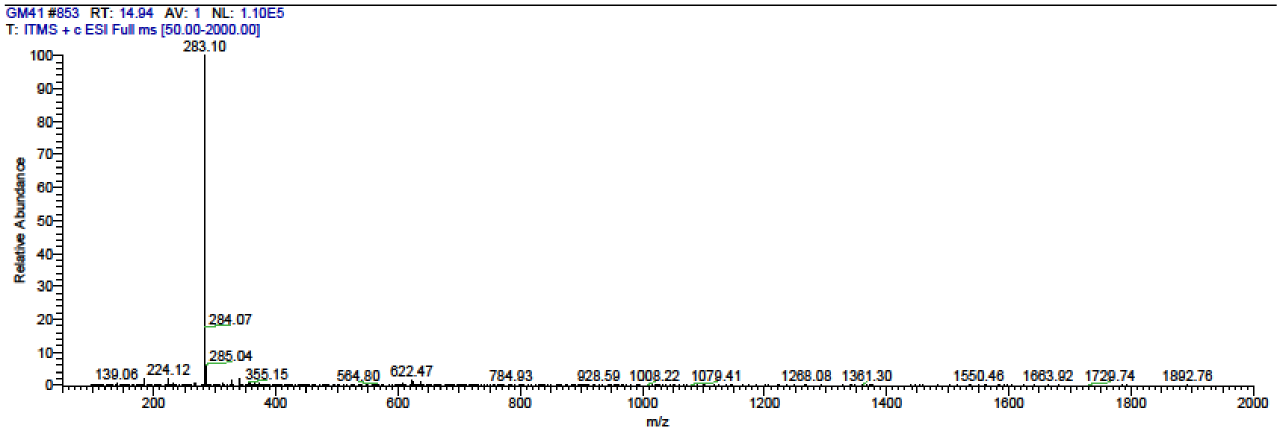

LC-MS of GH9

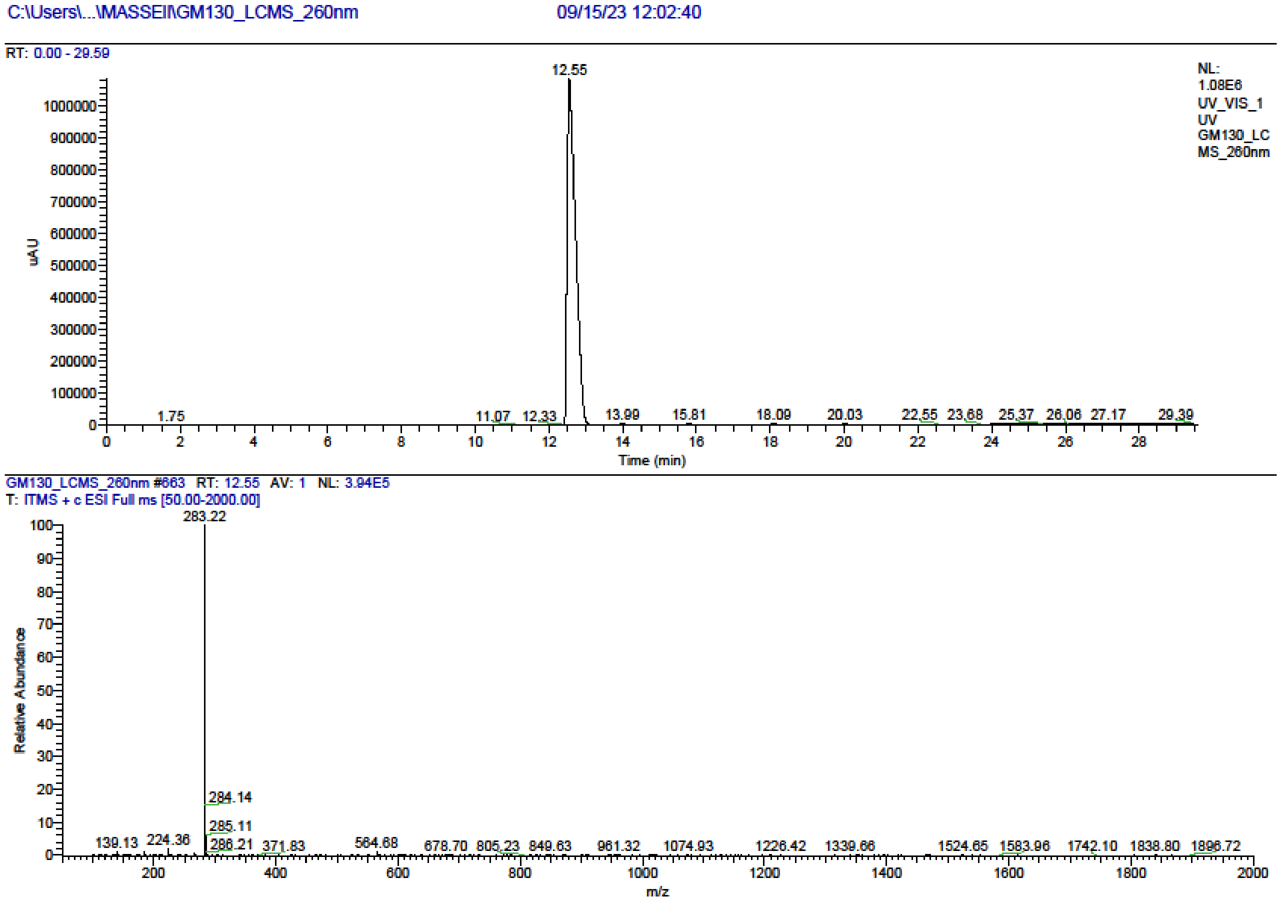

LC-MS of GH10

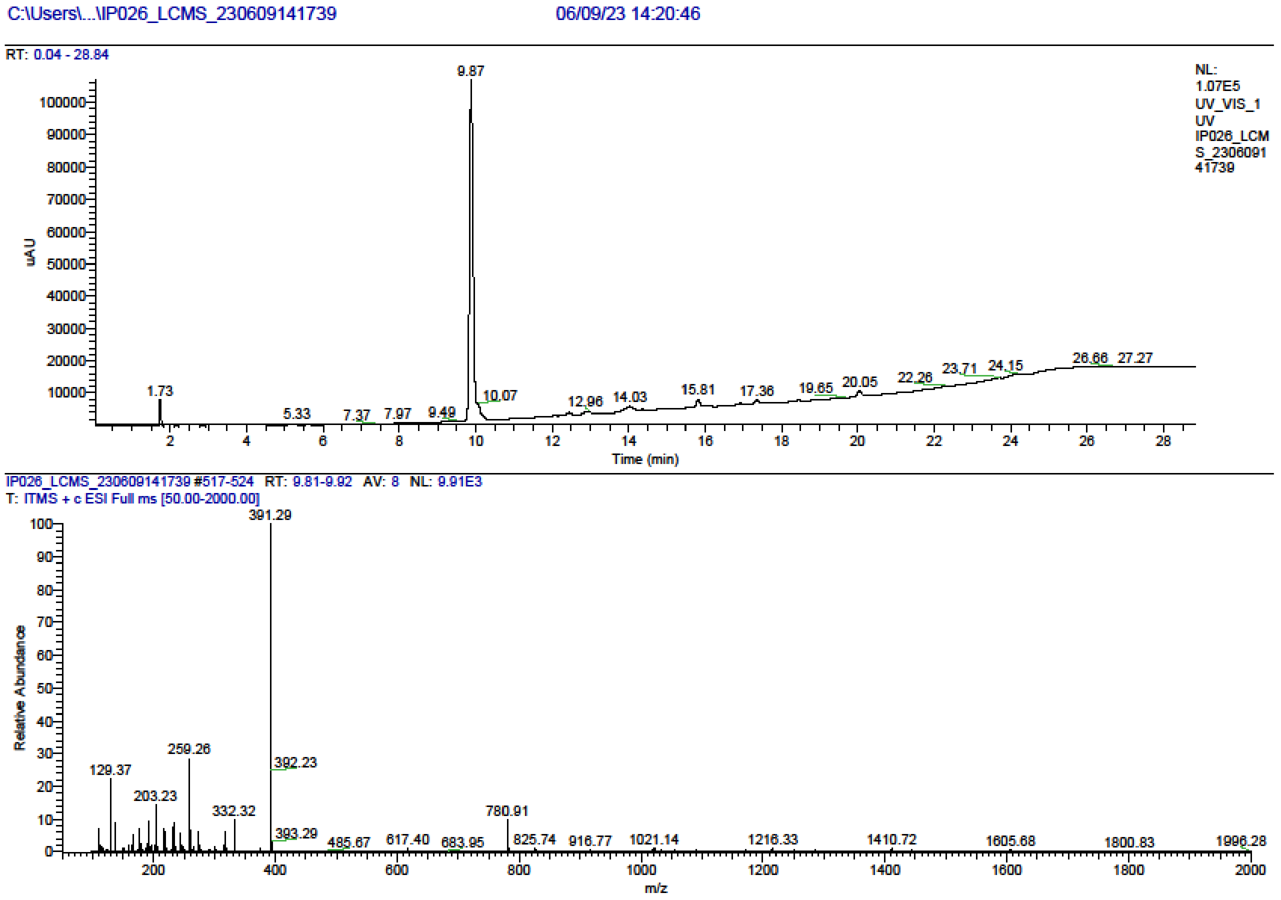

LC-MS of GH11

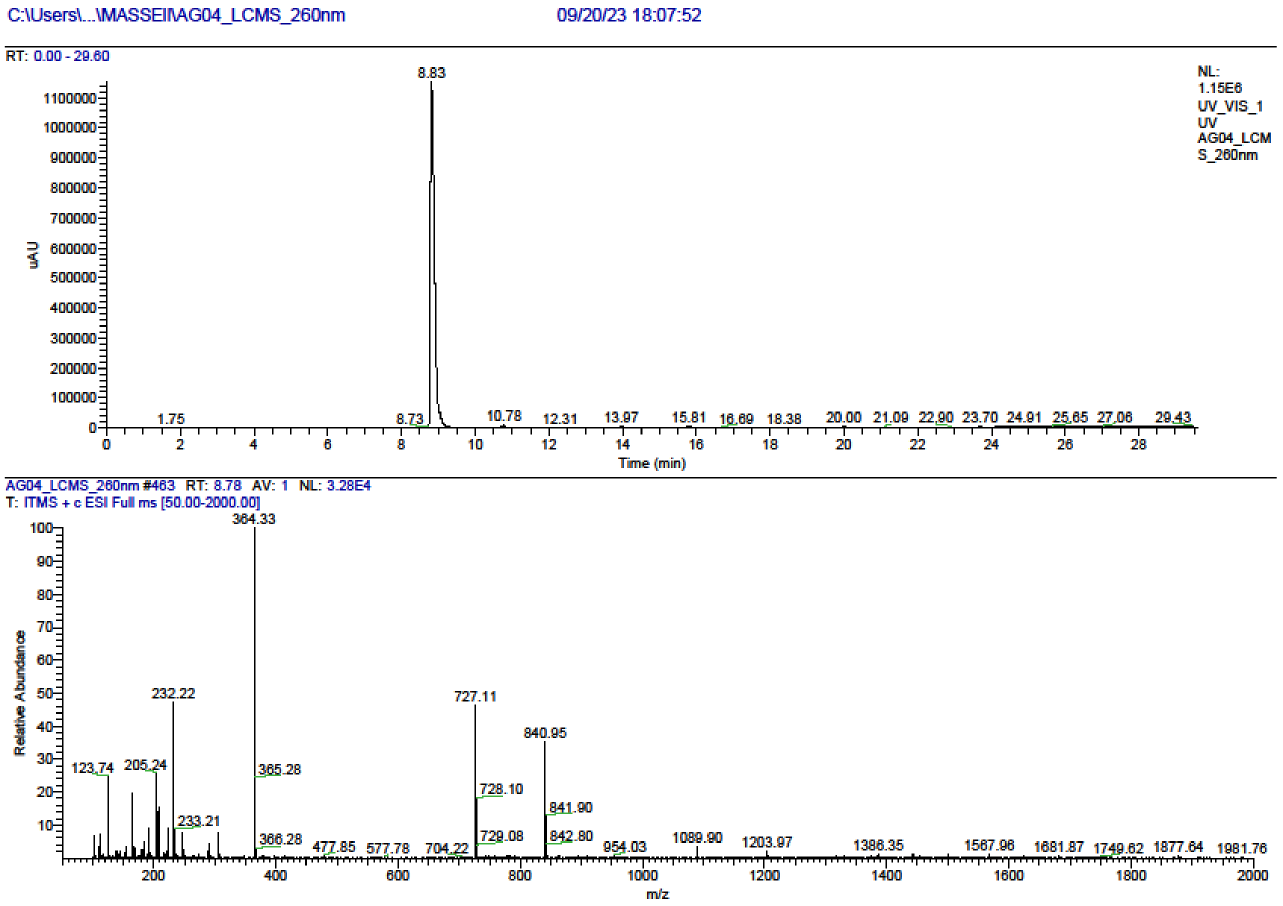

LC-MS of GH12

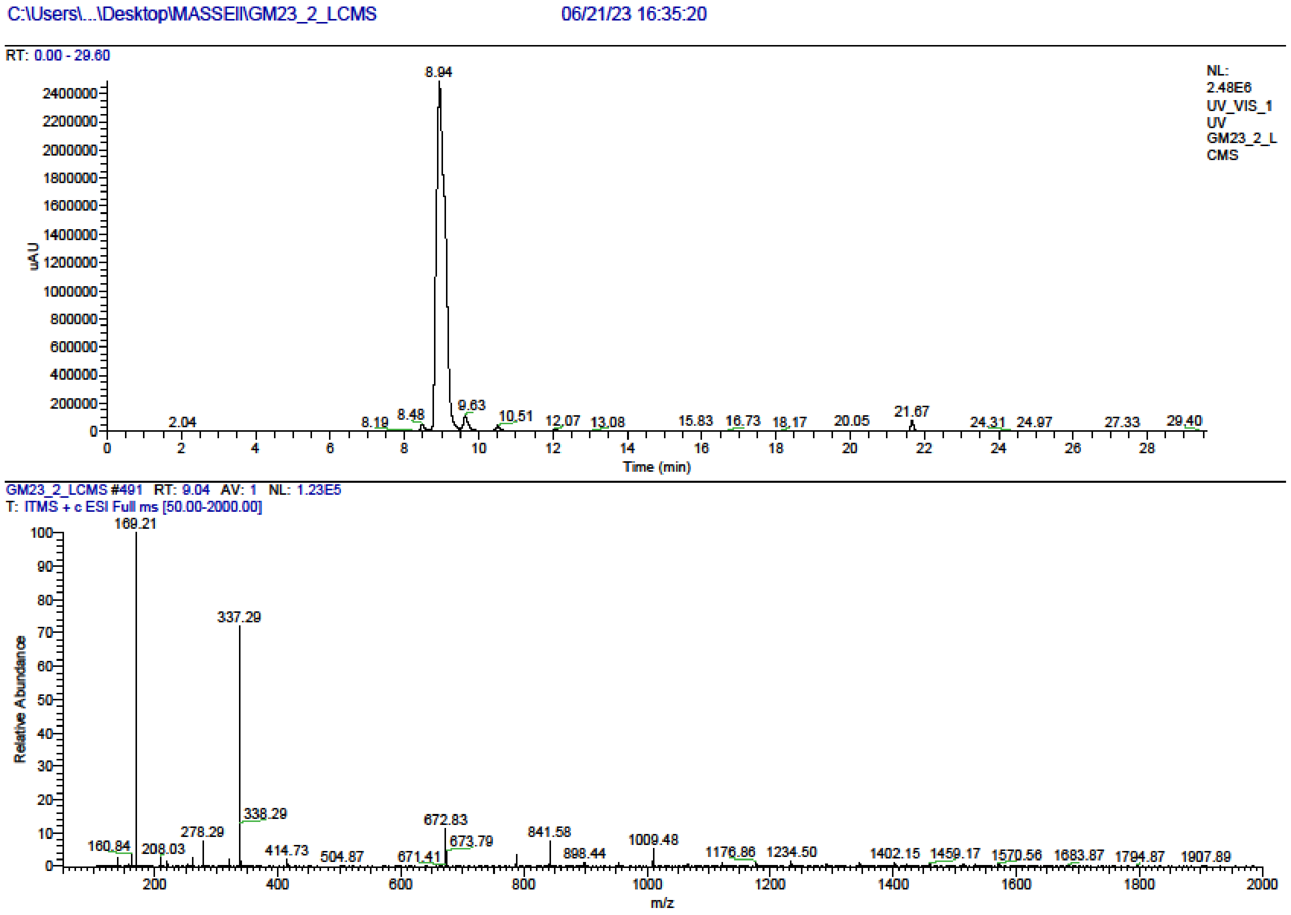

LC-MS of GH13

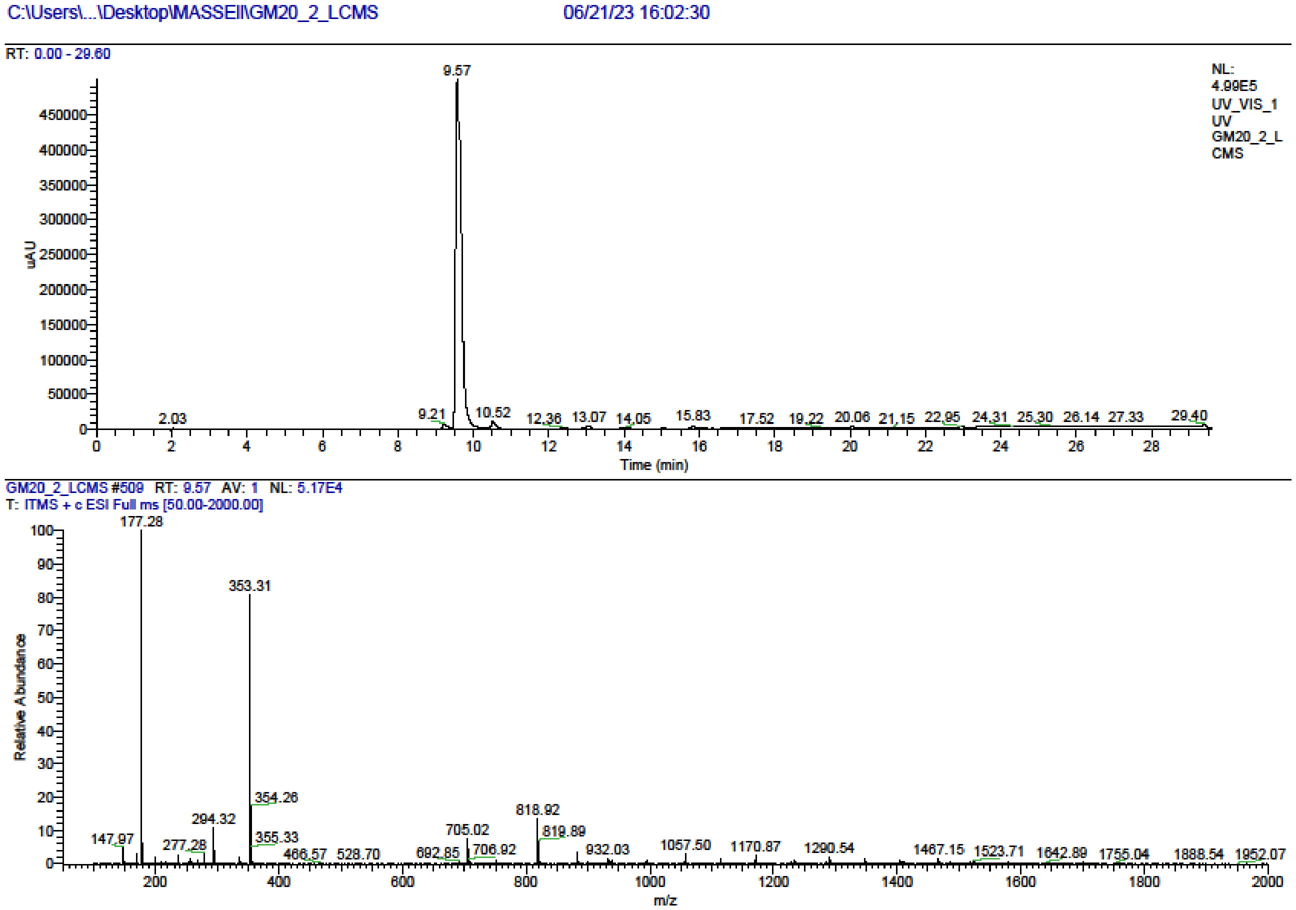

LC-MS of GH14

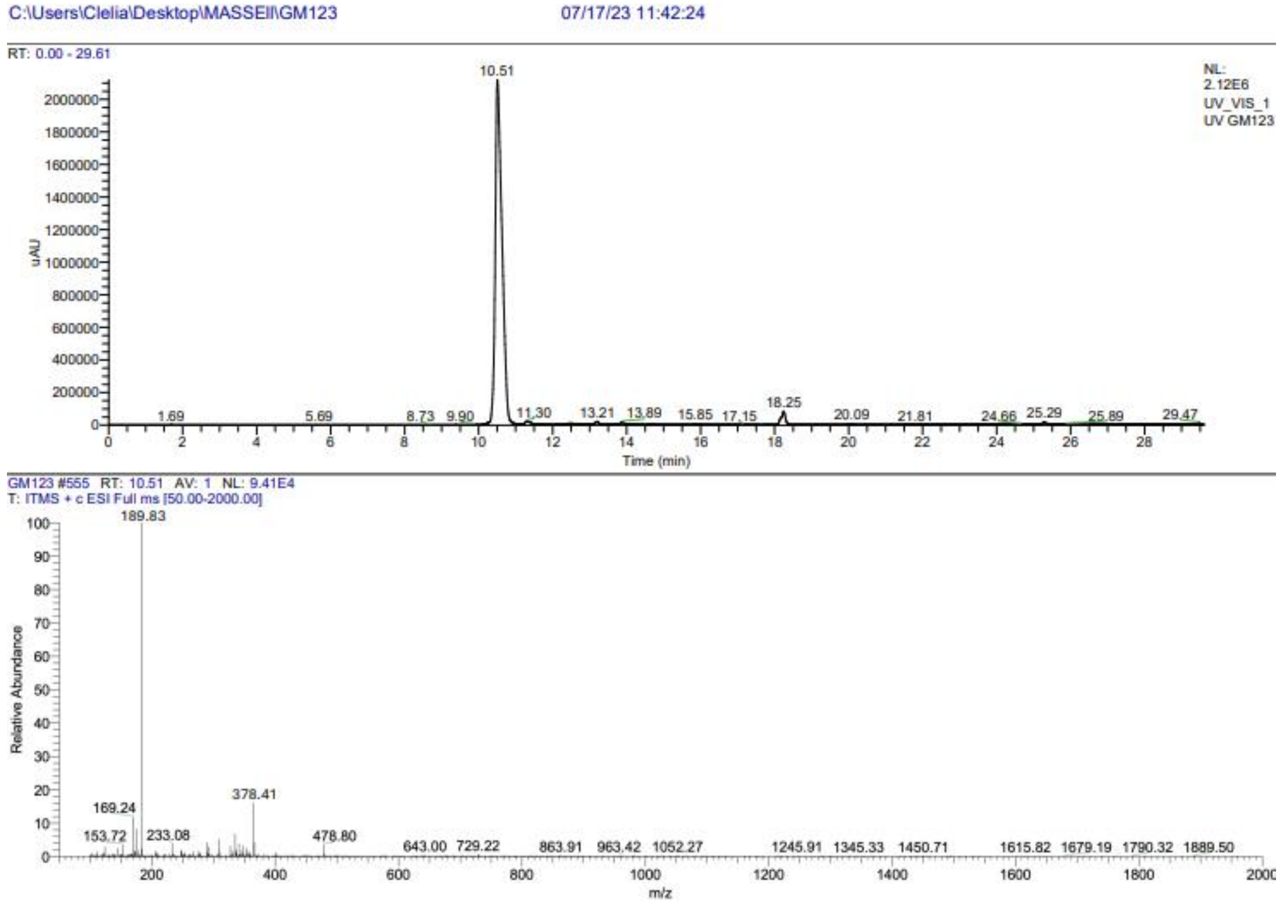

LC-MS of GH15

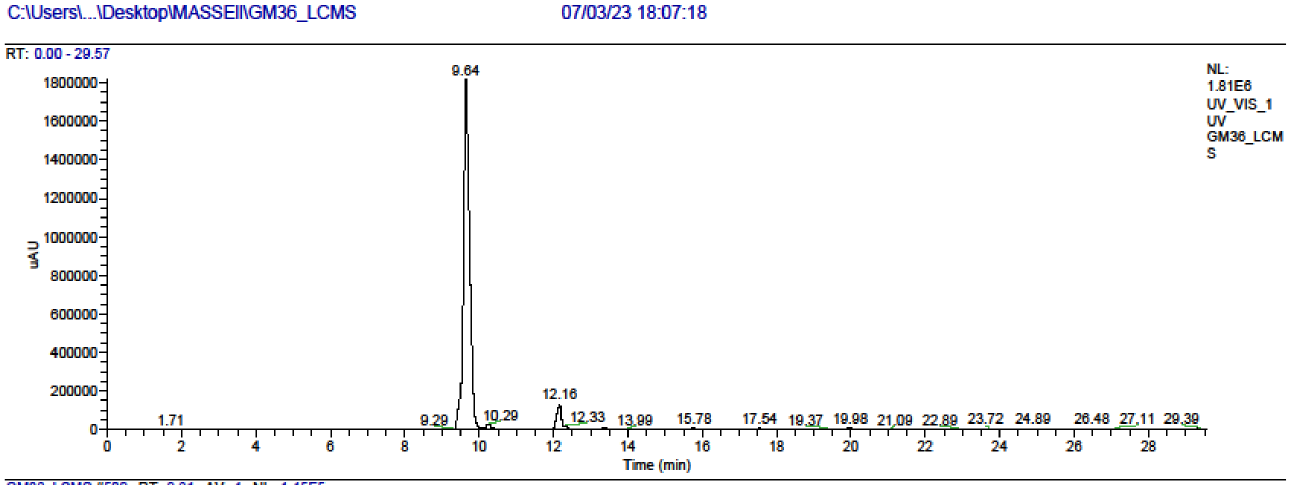

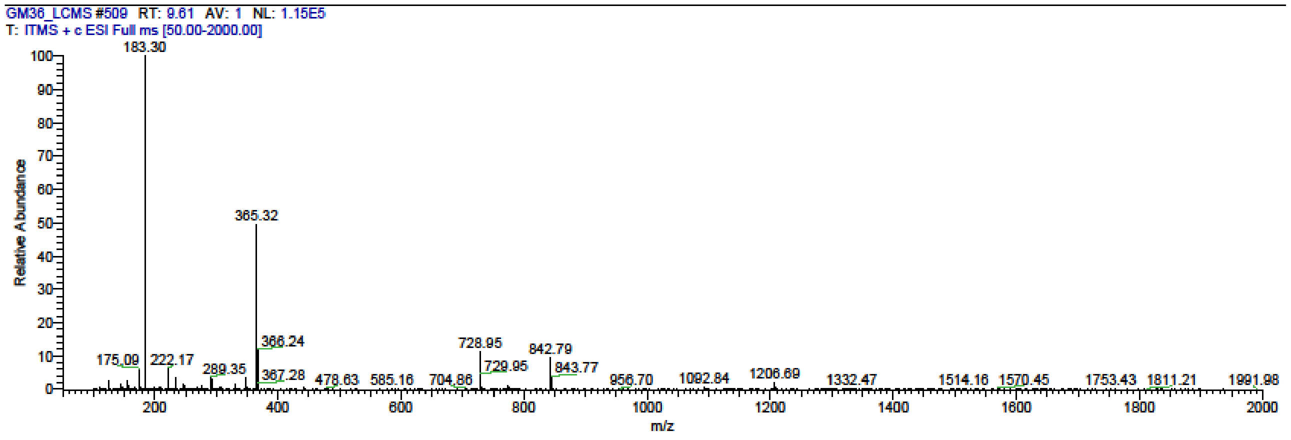

LC-MS of GH16

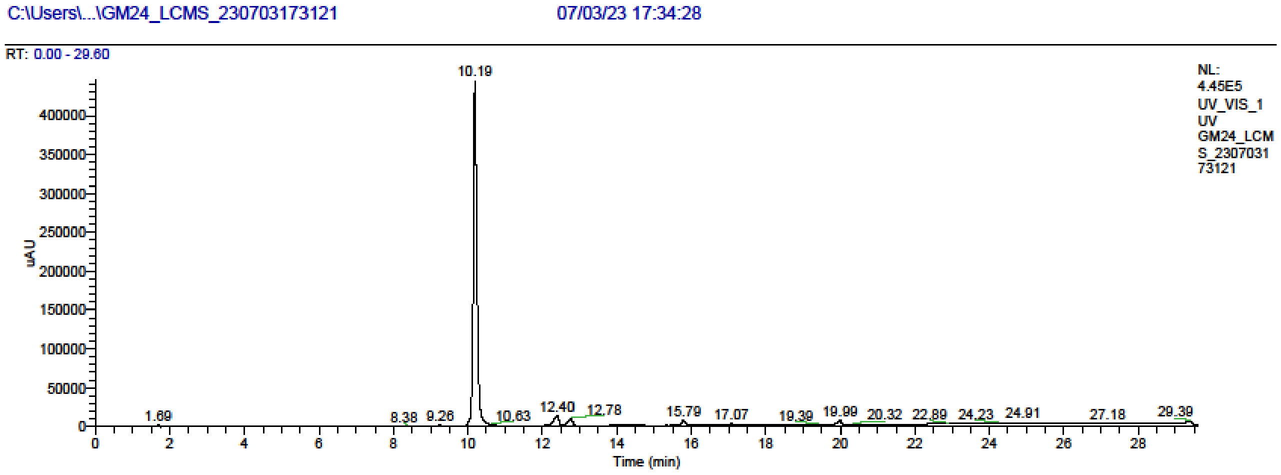

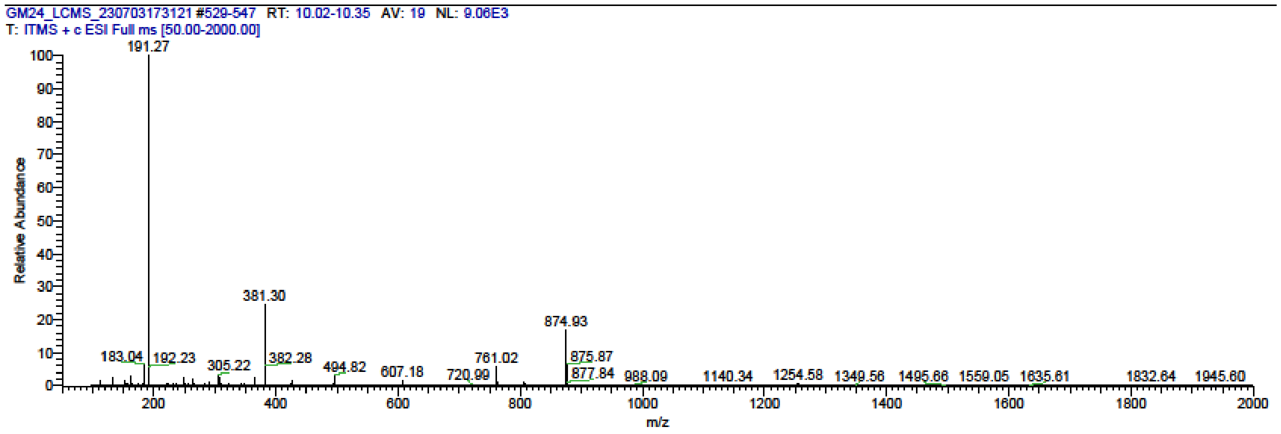

Supplement: Supplementary file 1 [file ijms-27-05282-s001.zip › ijms-4332446-supplementary.pdf]
